# Programming of cardiac metabolism by miR-15b-5p, a miRNA released in cardiac extracellular vesicles following ischemia-reperfusion injury

**DOI:** 10.1016/j.molmet.2024.101875

**Published:** 2024-01-11

**Authors:** Lucas C. Pantaleão, Elena Loche, Denise S. Fernandez-Twinn, Laura Dearden, Adriana Córdova-Casanova, Clive Osmond, Minna K. Salonen, Eero Kajantie, Youguo Niu, Juliana de Almeida-Faria, Benjamin D. Thackray, Tuija M. Mikkola, Dino A. Giussani, Andrew J. Murray, Martin Bushell, Johan G. Eriksson, Susan E. Ozanne

**Affiliations:** 1 Wellcome-MRC Institute of Metabolic Science and Medical Research Council Metabolic Diseases Unit, University of Cambridge, Cambridge, UK; 2 MRC Lifecourse Epidemiology Unit, University of Southampton, UK; 3 Finnish Institute for Health and Welfare, Public Health Unit, Finland; 4 Clinical Medicine Research Unit, MRC Oulu, Oulu University Hospital and University of Oulu, Oulu, Finland; 5 Department of Clinical and Molecular Medicine, Norwegian University of Science and Technology, Trondheim, Norway; 6 Department of Physiology, Development, and Neuroscience, University of Cambridge, Cambridge, UK; 7 Folkhalsan Research Center, Helsinki, Finland; 8 Faculty of Medicine, University of Helsinki, Finland; 9 CRUK Beatson Institute, Garscube Estate, Switchback Road, Bearsden, Glasgow, G61 1BD, UK; 10 Department of General Practice and Primary Health Care, University of Helsinki and Helsinki University Hospital, Finland; 11 Singapore Institute for Clinical Sciences, Agency for Science Technology and Research, Singapore, Singapore; 12 Department of Obstetrics and Gynaecology, Yong Loo Lin School of Medicine, National University of Singapore, Singapore, Singapore

**Keywords:** Maternal obesity, Cardiac metabolism, miR-15b, CVD biomarker, Sex differences, Developmental programming

## Abstract

**Objective:**

We investigated the potential involvement of miRNAs in the developmental programming of cardiovascular diseases (CVD) by maternal obesity.

**Methods:**

Serum miRNAs were measured in individuals from the Helsinki Birth Cohort (with known maternal body mass index), and a mouse model was used to determine causative effects of maternal obesity during pregnancy and ischemia-reperfusion on offspring cardiac miRNA expression and release.

**Results:**

miR-15b-5p levels were increased in the sera of males born to mothers with higher BMI and in the hearts of adult mice born to obese dams. In an *ex-vivo* model of perfused mouse hearts, we demonstrated that cardiac tissue releases miR-15b-5p, and that some of the released miR-15b-5p was contained within small extracellular vesicles (EVs). We also demonstrated that release was higher from hearts exposed to maternal obesity following ischaemia/reperfusion. Over-expression of miR-15b-5p *in vitro* led to loss of outer mitochondrial membrane stability and to repressed fatty acid oxidation in cardiomyocytes.

**Conclusions:**

These findings suggest that miR-15-b could play a mechanistic role in the dysregulation of cardiac metabolism following exposure to an *in utero* obesogenic environment and that its release in cardiac EVs following ischaemic damage may be a novel factor contributing to inter-organ communication between the programmed heart and peripheral tissues.

## Highlights

• Hearts from offspring of obese dams are more vulnerable to ischemia-reperfusion damage.
• Increased maternal BMI is associated with increased offspring serum miR-15b in humans.
• Maternal obesity leads to increased cardiac miRNA-15b expression in mice offspring.
• miR-15b-5p regulates cardiomyocyte metabolism *in vitro*.
• miR-15b-5p is released from hearts basally and increased in response to ischemia.

## Abbreviations

ACTB: Beta Actin
ADP: Adenosine Diphosphate
AWERB: Animal Welfare and Ethical Review Body
BCA: Bicinchoninic Acid
BMI: Body Mass Index
CBAL: Core Biochemical Analysis Laboratory
CD31: Cluster of Differentiation 31
CK: creatine kinase
CVD: Cardiovascular Diseases
DMEM: Dulbecco's Modified Eagle Medium
DMSO: Dymethil sulfoxide
EGTA: Ethylene glycol-bis(β-aminoethyl ether)-N,N,N′,N'-tetraacetic acid
EVs: Extracellular Vesicles
GEO: Gene Expression Omnibus
GSE: Gene Expression Series
HBCS: Helsinki Birth Cohort Study
HEPES: N-2-hydroxyethylpiperazine-N-2-ethane sulfonic acid
Higd1a: HIG1 Domain Family Member 1A
IPA: Ingenuity® Pathway Analysis
LC-MS: Liquid Chromatography Mass Spectrometry
miRNA: MicroRNA
MTT: 3-(4,5-Dimethylthiazol-2-yl)-2,5-Diphenyltetrazolium Bromide
NRF1: Nuclear Respiratory Factor 1
NTA: Nanoparticle Tracking Analysis
PCR: Polymerase Chain Reaction
PPARA: Peroxisome Proliferator-Activated Receptor Alpha
PPARGC1A: Peroxisome Proliferator-Activated Receptor Gamma Coactivator 1-Alpha
ROC: Receiver-Operating Characteristic
T2D: Type 2 Diabetes
TSG101: Tumor Susceptibility Gene 101
UPLC: Ultra Performance Liquid Chromatography

## 1 Introduction

Environmental and nutritional imbalances during fetal and early post-natal life increase the risk of cardiometabolic diseases such as obesity [1], metabolic syndrome [2] and cardiovascular diseases (CVD) in the offspring [3,4]. This has been termed developmental programming and is based on findings from epidemiological studies and analysis of animal models. These observations include evidence for a relationship between maternal obesity during pregnancy and offspring CVD risk in humans and animal models [[5], [6], [7], [8]]. The Aberdeen Cohort study (that included 37,709 people with birth records from 1950 to 1976), revealed that individuals born to obese mothers had an increased risk of hospital admission and premature death from cardiovascular complications compared to those born to lean mothers [5]. Similar observations were made in the Helsinki Birth Cohort Study, with positive association between maternal BMI and offspring type 2 diabetes (T2D) as well as CVD [9].

The associations between maternal BMI and offspring CVD are in part independent of inherited genetic susceptibility, as evidenced by the comparison between siblings born before and after maternal gastrointestinal bypass surgery. These studies showed significant improvement in several markers of metabolic and cardiac health including a reduction in adiposity, plasma insulin and systolic blood pressure in children born to mothers after they had lost weight compared to sibling born before the maternal bariatric surgery, when the mother had obesity [10]. Studies in animal models have also demonstrated a causative relationship between maternal overnutrition during pregnancy and offspring cardiac dysfunction. Our own studies showed that young male mice exposed to maternal obesity during fetal life and suckling, but fed a control diet from weaning, develop cardiac hypertrophy [8] and dysfunction [6,7] in young adult life.

There is growing evidence that there are sexually dimorphic responses of male and female progeny to a suboptimal *in utero* environment [11]. In general, male fetuses are more vulnerable to the detrimental effects of a suboptimal *in utero* environment [[11], [12], [13], [14]]. This is true in the context of maternal obesity with limited data showing that male, but not female, mouse offspring from obese dams demonstrate cardiomyocyte hypertrophy in young adulthood [15].

The mechanisms linking fetal exposure to maternal overnutrition and long-term poor cardiometabolic health are poorly understood but are likely to involve epigenetic processes [16]. MicroRNAs (miRNAs) are small endogenous single-stranded RNAs of ∼21 nucleotides in length, which post-transcriptionally regulate gene expression. An increasing number of studies have identified miRNAs as pivotal mediators of programmed phenotypes [[17], [18], [19], [20]]. Specific miRNA signatures are associated with defined pathological conditions, and the role of miRNAs in cardiovascular pathophysiology is well-established [21]. MiRNAs have also been extensively proposed as valuable circulating biomarkers for the prognosis of a variety of human pathologies [22,23]. We, and others, have identified microRNAs as tissue-specific mediators of nutritional programming, and established that poor maternal nutrition alters adipose and hepatic miRNA profiles in the offspring [[17], [18], [19], [20]], which may contribute to disease risk later in life.

The aim of this study was therefore to test the following inter-related hypotheses: 1) miRNAs could contribute mechanistically to programmed cardiac dysfunction in offspring exposed to gestational maternal obesity and 2) miRNAs act as biomarkers of suboptimal *in utero* exposures. We tested the hypotheses using a combination of studies in an established human cohort and a mouse model of maternal obesity. First, we identified a panel of miRNAs that were indicators of heart disease and profiled their expression in middle-aged humans from the Helsinki Birth Cohort Study and then established which were also differentially expressed in heart tissue of young mice born to obese and lean dams. Using a Langendorff preparation, we determined *ex vivo* that miRNAs were released from isolated hearts in response to ischemia-reperfusion in mice born to obese and lean dams, and adopting *in vitro* approaches, we determined the functional consequence of changes in miRNA expression in an immortalized cardiac cell line.

## 2 Material and methods

### 2.1 Lead contact

Further information and requests for resources and reagents should be directed to and will be fulfilled by the Lead Contact, Susan E. Ozanne (seo10@cam.ac.uk).

### 2.2 Data and code availability

The proteomics dataset generated during this study is available as supplemental material (Table S1).

### 2.3 Publicly available data collection and analysis

Metadata and miRNA expression data were obtained from two independent datasets: GEO accession GSE 148153 [24] and GEO accession GSE49823 [25]. Normalized matrix files were downloaded and pre-processed using GEOquery v.2.66.0 and analysed using linear models from limma v.3.54.0 for R v.4.1.2. For GSE49823, miRNAs with missing values (CT = 40) in more than 25 % of samples were filtered out. For GSE148153, probes with undetectable signal in samples from one or both groups were filtered out and data from individual arrays were normalized using quantile Cyclic Loess method prior to statistical analysis. For both datasets, variables with the lowest expression levels (lowest quartile) were removed and miRNAs were deemed significantly regulated when adj. p-value <0.01.

### 2.4 miRNA target prediction and pathway enrichment analysis

To identify genes confidently annotated as potential targets of miR-15b-5p, and potential enriched pathways in the Kyoto Encyclopedia of Genes and Genomes (KEGG), we used mirPath v.3 [26] to query the TargetScan database, applying a context score threshold of −0.3.

### 2.5 Human serum samples characteristics

The Helsinki Birth Cohort Study (HBCS) is a longitudinal study following up individuals born in Helsinki between 1934 and 1944 [27,28], with pregnancy and newborn data collected from records of the birth hospital. These records include maternal height and weight before delivery. In this cohort, maternal BMI was based on measurements immediately prior to delivery in hospital because there is no pre-pregnancy BMI data available. Maternal and offspring anthropometric characterization is described in Table S2. Human sera (n = 95) from a subset of individuals recruited to the HBCS were randomly selected for use in the current study. Serum was obtained after overnight fasting from individuals whose mothers were characterized either as “obese” if their BMI >30 kg/m^2^ (mean 32.5, SD 2.6 kg/m^2^) or “control” if their average BMI was <30 kg/m^2^ (mean 26.2, SD 0.4 kg/m^2^) at time of delivery (Table S2).

### 2.6 Hemolysis assessment

Serum hemolysis was assessed by spectrophotometric measurement of oxyhemoglobin absorbance at ʎ = 414 nM on a Tecan Infinite® M1000 Pro instrument. Serum samples were classified as being hemolyzed if the reading exceeded a value of 0.25. Based on this criteria, 12 samples out of 95 were considered hemolyzed and excluded from the experiment prior to any miRNA analysis.

### 2.7 Animal handling

The mouse model used has been described in detail previously [20,29]. Briefly, female C57BL/6J mice, were fed *ad libitum* either a standard control chow diet (RM1) or a highly palatable energy-rich obesogenic diet and sweetened condensed milk (Nestle, York, United Kingdom) fortified with mineral and vitamin mix AIN93G. Both diets were purchased from Special Dietary Services, Witham UK. Dams were maintained on their respective experimental diets prior to pregnancy as well as throughout pregnancy and lactation. Offspring born to control and obese mothers were weaned onto standard chow (RM1) fed *ad libitum*. At 8 weeks of age, offspring were killed by a rising CO2 concentration either before or after an overnight fast. Two independent cohorts of animals euthanized at different metabolic states (fasted or fed) were used in this study and male mice from 10 control and 12 obese dams and female mice from 16 control and 12 obese dams were used for molecular analyses. Heart was weighed, snap frozen and stored at −80 °C until use. At 12 weeks of age, male mice were killed by cervical dislocation in a fed state and heart was rapidly excised for *ex vivo* experiments.

### 2.8 Culture of H9c2 cells

H9c2 cells, derived from embryonic rat ventricular tissue, were acquired from ATCC (Manassas, VI, USA) and cultured in Dulbecco's Modified Eagle Medium high glucose [DMEM (Thermo Fisher Scientific, Waltham, MA, United States)] containing 10 % fetal bovine serum [FBS (Thermo Fisher Scientific)], 4 mM l-Glutamine and 1 % penicillin and streptavidin under controlled conditions (37 °C in 5 % CO_2_). H9c2 cell differentiation was induced by restricting the FBS supply to 1 % and by adding 1 μM retinoic acid (Merck, Darmstadt, Germany) to the culture medium for 7 days. Medium was changed every other day, but fresh retinoic acid was added daily. This protocol has been shown to induce differentiation of H9c2 cells towards a cardiac myotube phenotype [30], and we observed that differentiated cells expressed TNNT2, a cardiomyocyte marker (Fig. S1). Using a MycoAlert® Mycoplasma Detection Assay (Lonza, Basel, Switzerland), media from H9c2 cultures were tested and deemed free of mycoplasma contamination.

### 2.9 Culture of primary cardiomyocytes and fibroblasts

Fetal cardiac cells were isolated as described previously with some minor modifications [31]. Briefly, fetal hearts were excised, minced and digested. Cells were dispersed into culture medium [DMEM for Primary Cell Isolation (Thermo Fisher Scientific) supplemented with 10 % heat inactivated FBS and 1 % penicilin/streptomycin] and strained using a 70 μm strainer. Cultures were incubated in 12-well plates for 2 h to allow for fibroblasts and endothelial cells to attach to the bottom of the plate. Medium containing the remaining cells (cardiomyocytes) was transferred to fresh wells. Fibroblasts were incubated in fibroblast growth medium (High glucose DMEM/F12 supplemented with 10 % FBS, 4 mM l-Glutamine and 1 % penicillin and streptavidin) under controlled conditions (37 °C in 5 % CO_2_). Cells were visualized daily for morphological characteristics.

### 2.10 Langendorff heart perfusion

Langendorff heart perfusion was conducted as described previously [6]. Briefly, hearts from 12-week-old mice were rapidly excised and immediately immersed into ice-cold Krebs-Henseleit bicarbonate buffer. Previous pilot experiments showed that 12-week of age represents the youngest age that it was possible to successfully maintain the beating heart ex-vivo for the duration of the Langendorff experiment. Hearts were then mounted onto a Langendorff preparation and perfused through coronary arteries after canulation via the aorta. Following the recording of basal cardiac function, the hearts were subjected to an ischemia-reperfusion challenge involving 15 min of global ischemia by stopping the perfusion, followed by 30 min of reperfusion. The coronary effluent (2 mL) was collected before ischemia (−15 min) and at the onset of reperfusion (0 min), and at 5, 15, and 30 min of reperfusion. Samples were immediately frozen in liquid nitrogen and kept at −80 °C until analysis.

### 2.11 Creatine kinase in the heart perfusate

Creatine kinase levels in perfusates were measured by the Core Biochemical Analysis Laboratory (CBAL) of the Addenbrookes Hospital in Cambridge using a Siemens Dimension® EXL analyzer (Siemens Healthcare Diagnostics, Surrey, UK).

### 2.12 Perfusate fractioning and extracellular vesicle isolation

For extracellular vesicles (EV) purification from Langendorff perfusates, we adapted a previously described protocol for *in vitro* EV isolation [32]. Briefly, 400 μl perfusate samples were centrifuged at 300 g for 10 min at 4 °C and the pellet retrieved for analysis of miRNA content in circulating whole cells. The supernatant was then concentrated using Amicon Ultra-4 filter columns [UFC801024 (Merck)] following the manufacturer protocol and centrifuged again for another 5 min at 3000 g at 4 °C for dead cells/apoptotic bodies and at 10,000 g for 30 min for large micro precipitation. The supernatant was then transferred to ultracentrifuge tubes (Beckman Coulter, Brea, CA, United States) and centrifuged at 100,000 g for 90 min at 4 °C. The supernatant was discarded, and the remaining pellet containing the purified EVs was stored in a −80 °C freezer.

### 2.13 Nanoparticle Tracking Analysis (NTA)

Purified EV fractions were loaded into a NanoSight NS300 (Malvern Panalytical, Malvern, UK) equipment using an infusion pump. Nano particles were recorded and analyzed for size and concentration using NanoSight NTA software v3.30.

### 2.14 Transmission electron microscopy

Extracellular vesicles were fixed and embedded by the Electron Microscopy Core Facility of the Wellcome-MRC Cambridge Stem Cell Institute following standard protocols. Images were acquired using a Hitachi HT7800 transmission electron microscope with magnification between 40,000 and 150,000.

### 2.15 EV uptake assay

Acceptor cells were cultured following standard culturing conditions. Cytoplasm was stained with ViaFluor®488 proliferation dye (Biotium, Fremont, CA, USA) 24h prior to the uptake experiment and seeded at 3 x 103 cells per well in a 384-well plate (PerkinElmer, Waltham, MA, United States). The following day, cells were stained with NucBlue™ Live ReadyProbes™ Reagent (Hoechst 33342) (Thermo Fisher Scientific), washed and maintained in regular growth medium. Langendorff perfusate-derived EVs were stained with DiOC18(7) (DiR) (Thermo Fisher Scientific). Stained EVs were added to cell medium at a final concentration of 13 ng protein/μl. Cells were imaged for 16 h using an Opera Phenix™ system (PerkinElmer). Analysis of internalization of EVs was performed using Harmony v.4.9 (Perkin Elmer).

### 2.16 Western blotting

EV containing pellets and cells were homogenized using RIPA buffer supplemented with 1 mM PMSF, 2 mM Na3VO4 and protease inhibitors cocktails [PhosStop and complete Mini EDTA proteinase inhibitor (Roche, Basel, Switzerland)] and stored in −80 °C freezer. The protein content of the extracts was quantified using the Micro BCA Protein Assay Kit (Thermo Fisher Scientific). Samples were loaded into a Novex™ 4–12 %, Tris-Glycine Gel (Thermo Fisher Scientific), and proteins were subjected to electrophoresis and transferred to a nitrocellulose membrane. Immunoblotting was conducted using specific primary antibodies anti-TSG101 (Abcam, Cambridge, UK Cat# ab125011) diluted 1:1000, anti-CD31 (R&D, Minneapolis, MN, United States Cat# AF3628) diluted 1:500, anti-ACTB (Merck, Cat# A2228) diluted 1:1000, ani-TNNT2 (Thermo Fisher Scientific, cat#MA5-12960) diluted 1:1000, and anti-TUBB5 (Abcam, cat#ab6046), and blots were developed using a chemiluminescence Immobilon Forte solution (Merck) in a BioRad Chemidoc equipment. After imaging, antibodies were stripped from the membrane using a commercial stripping solution [Restore™ Western Blot Stripping Buffer (Thermo Fisher Scientific)] for 30 min before re-blotting.

### 2.17 Indirect immunofluorescence

Cells were cultured over glass coverslips, fixed for 15 min in 4 % paraformaldehyde, and permeabilized in 0.1 % Triton X-100 in PBS. Fixed cells were blocked for 60 min in 1 % BSA in PBS, incubated overnight at 4 °C with anti-cardiac troponin T antibody (Thermo Fisher Scientific, Cat# MA5-12960) diluted 1:50 and incubated with phalloidin (Thermo Fisher Scientific, Cat# A12381) and NucBlue™ Live ReadyProbes™ Reagent (Hoechst 33342) for 10 min. Cells were then washed in PBS and incubated for 1 h at room temperature with a secondary antibody Alexa-Fluor-488 donkey anti-mouse IgG (H+L) (Thermo Fisher Scientific, Cat# A21202). Images were acquired on a Leica DMi8 inverted microscope, equipped with a Leica DFC365 FX camera (Leica, Wetzlar, Germany).

### 2.18 RNA purification and cDNA synthesis

Sera and Langendorff perfusates were thawed and centrifuged at 3000 g for 5 min at 4 °C to remove any remaining cell debris. Total RNA was isolated using a miRCURY RNA Isolation kit Biofluids (Exiqon, Vedbaek, Denmark). Total murine cardiac RNA was isolated using either a mirVana™ miRNA Isolation Kit (Thermo Fisher Scientific) or a Qiagen (Hilden, Germany) miRNeasy mini kit protocol according to manufacturers' instructions. Total RNA from cell cultures was isolated following washing with PBS, using a Qiagen miRNeasy micro kit, following the manufacturer's protocol. Total RNA was isolated from EVs following the Qiagen miRNeasy micro kit protocol with the addition of a spike-in cocktail (UniSp2/4/5). Freshly isolated RNA was quantified (Nanodrop, Thermo Fisher Scientific) and, when applicable, the integrity assessed by determination of the integrity of the 28S and 18S ribosomal RNA bands following electrophoresis through a 1 % agarose gel. RNA from cardiac samples were considered of high quality and therefore included in the analysis when no clear smear could be identified, and the intensity of the 28S band was approximately twice that of the 18S band. Assessing small RNA integrity from perfusates, sera, and EV was not feasible as these extracts (as expected) lack integral ribosomal RNA in sufficient amounts. However, miRNAs, due to their small size, chemical structure (usually double-stranded), association with proteins and encapsulation in EVs, are generally considered more stable than mRNA. Although we could not determine potential nuclease activity on these samples, we took measures to ensure miRNA preservation, such as quickly freezing samples upon collection and using sterile, nuclease-free plastics and pipettes. Samples were stored at −80 °C until use. Complementary DNA (cDNA) was generated using TaqMan® MicroRNA Reverse Transcription Kit (Thermo Fisher Scientific) or miRCURY LNA RT kit (Qiagen), with the addition of a synthetic spike-in control (UniSp6) to each reaction, following manufacturer's instructions.

### 2.19 qPCR

For each qPCR reaction in this study, cDNA was diluted between 1:15 and 1:60 following optimization. PCR was performed using either an Applied Biosystems 7900HT or an Applied Biosystems QuantStudio 7 Real-Time PCR Systems and TaqMan® Universal PCR Master Mix TaqMan MicroRNA Assays (Cat # 4427975) for hsa-miR-15b-5p (assay 000390), hsa-miR-199a-3p (assay 00234) and snoRNA202 (assay 001232) detection or miRCURY LNA miRNA PCR Assays (Cat # 339306) for hsa-miR-15b-5p (GeneGlobe ID # YP00204243), hsa-miR-199a-3p (GeneGlobe ID # YP00204536), hsa-miR-144 (GeneGlobe ID # YP00204754), hsa-miR-451a (GeneGlobe ID # YP02119305), UniSp2 (GeneGlobe ID # YP00203950), UniSp4 (GeneGlobe ID # YP00203953), UniSp6 (GeneGlobe ID # YP00203954) and SNORD68 (GeneGlobe ID # YP00203911) detection. Quantification was performed using the ΔΔCt method [33] with snoRNA202, SNORD68, UniSp2 or UniSp4 amplification as normalizing factors.

### 2.20 Stable isotope labelling by amino acids in cell culture (SILAC)

We used a pulsed-SILAC methodology as described previously [19]. In brief, H9c2 cells were transfected with 50 nM miR-15b-5p mimic or 50 nM negative control mimic (Qiagen) using Lipofectamine® RNAiMAX (Thermo Fisher Scientific). After preincubation, cells were cultured with a medium containing stable isotope labeled lysine for 24 h. Protein was extracted and digested, and the resulting peptides were eluted. Extracts were subjected to LC-MSMS on a Q Exactive with an EASY spray source coupled to an RSLC3000 nano UPLC (Thermo Fisher Scientific). MSMS data were processed in Maxquant 1.5.2.8 using a Uniprot *Rattus Norvegicus* dataset. Detected peptides were initially annotated to 2,379 proteins. Proteins containing one of the stable isotopes with no mapped peptides (intensity of ions detected equals to zero) in one or more samples were trimmed out of the final analysis, resulting in a final set of 1,294 proteins.

### 2.21 Outer mitochondrial membrane stability assessment

A fixed number of cells were resuspended in MiR05 respiratory medium [20 mM HEPES (Thermo Fisher Scientific), 0.5 mM EGTA (Merck), 3 mM MgCl_2_⋅6H_2_O (Merck), 10 mM KH_2_PO_4_ (Merck), 20 mM taurine (Merck), 1 mg/ml bovine serum albumin (Merck), 60 mM potassium lactobionate (Merck), and 110 mM sucrose (Thermo Fisher Scientific), pH7.1] at a density of 1.5 x 105 cells⋅mL-1, and the suspension transferred to Oxygraph-2K (Oroboros Instruments, Innsbruck, Austria) chambers. Cells were first permeabilized by injection of 1 μL of 10 mg⋅mL-1 digitonin (Sigma) in DMSO (final concentration of 5 μg⋅mL-1). A substrate-inhibitor titration was then performed, comprising sequential injection of 2 mM malate (Merck), 0.2 mM octanoyl l-carnitine (Tocris Bioscience, Bristol, UK), 10 mM ADP (Merck), 10 μM cytochrome c (Merck), 25 mM pyruvate (Merck), 10 mM glutamate (Merck), 10 mM succinate (Merck), 0.5 μM rotenone (Merck), and 2.5 μM antimycin A (Merck). Datlab v. 6.0 (Oroboros Instruments) was used for data acquisition and analysis.

### 2.22 mtDNA:nDNA quantification

Relative mtDNA copy number was quantified following a previously described protocol [34]. In brief, total DNA was extracted from frozen transfected, differentiated H9c2 cells using the DNeasy® Blood & Tissue Kit (Qiagen) according to manufacturer's instructions and quantified using the Quant-iT™ PicoGreen™ dsDNA assay (Thermo Fisher Scientific). 6 ng dsDNA was used for real-time qPCR using SYBR® Green JumpStart™ Taq ReadyMix™ reagents (Sigma Aldrich) and nDNA and mtDNA specific primers (Table S3) on a StepOnePlus™ Real-Time PCR System (Applied Biosystems). Results were analyzed using the ΔΔCt method.

### 2.23 Fatty acid oxidation assay

The fatty acid oxidation assay followed the protocol described in [31], adapted from [35]. Briefly, a filter paper saturated with 1 M NaOH was placed inside sealed wells containing transfected, differentiated H9c2 cells in fatty acid oxidation medium [12.5 mM HEPES, 0.3 % fatty acid-free BSA, 1 mM l-carnitine, 100 μM oleic acid medium containing 0.4 μCi/ml [1–^14^C]-oleate (PerkinElmer)] for 3 h at 37 °C. Disintegrations per minute from [1–^14^C]-CO_2_ derived salts were measured in the filter paper to determine oleate oxidation using a TRI-CARB 5110 TR Liquid Scintillation Counter system (PerkinElmer). Results were normalized to total protein extracted from cells using RIPA buffer and standard protein extraction protocol.

### 2.24 MTT metabolic viability assay

7.2 × 10^5^ H9c2 cells were evenly seeded in a 96 well plate and transfected with miR-15b-5p mimics or a negative control as described above. After 48 h, media was changed for complete DMEM containing 0.5 mg/ml 3-(4,5-dimethylthiazol-2-yl)-2,5-diphenyltetrazolium bromide. Cells were incubated for further 2.5 h; medium was aspirated and 100 μL DMSO (Merck) added to solubilise the formazan precipitate. After 15 min, absorbance was read using a Spark® multimode microplate reader (Tecan, Männedorf, Switzerland) at 570 nm.

### 2.25 Statistics

Details of statistical analysis (statistical tests used, number of individuals, animals or experimental iterations, identification of outliers and precision measures) can be found in the figure legends. For animal studies, the dam is the statistical unit; data obtained from same sex siblings are represented as an average and counted as one. Unpaired two-tailed Student t-test were employed to identify statistically significant differences in univariate pairwise analyses and factorial analyses of variance (ANOVA) were employed to determine statistically significant group and sex differences using Prism 9 (GraphPad, La Jolla, California, USA). Mixed linear models with passage number as random effect were used to identify statistical differences between treatments in cell culture experiments with more than one iteration using R v.4.1.2. Linear mixed-effects linear models with wells as random effect were used to identify statistical differences between cell lines across time in the EV uptake experiment using R v.4.1.2. Receiver operating characteristic was performed using pROC v.1.18.0 for R. Human serum data multiple regression analyses were performed using SPSS and Pearson's r was calculated for data normally distributed; otherwise, Spearman's correlation was used. SILAC data was analysed through multiple linear models with Bayesian correction using limma package v.2.16.0 for R, and data interpretation and pathway enrichment analysis was performed in Ingenuity® Pathway Analysis (IPA – Qiagen). A p-value cut-off of 0.05 was used to determine genes to be mapped to IPA networks. Cumulative distributions of the fold changes were calculated in R v. 4.3.2. Two-sided Kolmogorov-Smirnov tests determined if differences between distributions were statistically significant.

### 2.26 Study approval

Human studies were approved by the Coordinating Ethics Committee at Helsinki and Uusimaa Hospital District. All participants signed a written informed consent. The animal research was regulated under the Animals (Scientific Procedures) Act 1986 Amendment Regulations 2012 following ethical review by the University of Cambridge Animal Welfare and Ethical Review Body (AWERB, project number PP8498895).

## 3 Results

### 3.1 Maternal obesity elevates serum miRNA-15b levels in humans and cardiac miRNA-15b expression in mice offspring

To identify biomarkers of both acute and chronic coronary disease, we used GEOquery (v.2.40.0) [36] for R (v.4.2.1) to scrutinise publicly available human datasets in the GEO database [37], and investigated independent human datasets of serum markers of coronary heart disease available in the literature. We observed miR-15b-5p and miR-199a-3p as being consistently increased in myocardial infarction (GEO accession GSE 148153 [24]), unstable coronary artery disease (GEO accession GSE49823 [25]) and coronary heart disease in two independent sets of patients [38] (Table 1).

**Table 1:** Overlap of significantly regulated miRNAs (adj. p-value <0.01) from publicly available datasets (see methods for details) and in two independent datasets by Su et al., 2020.

GSE49823 x GSE148153	GSE49823 x GSE148153 x Su et al.
hsa-let-7d-5p	hsa-miR-15b-5p
hsa-let-7g-5p	hsa-miR-199a-3p
hsa-miR-15b-5p	
hsa-miR-20a-5p	
hsa-miR-20b-5p	
hsa-miR-26b-5p	
hsa-miR-30c-5p	
hsa-miR-34a-5p	
hsa-miR-132-3p	
hsa-miR-151a-5p	
hsa-miR-195-5p	
hsa-miR-199a-3p	
hsa-miR-223-3p	
hsa-miR-224-5p	
hsa-miR-296-5p	
hsa-miR-363-3p	
hsa-miR-532-5p	
hsa-miR-769-3p	

**Figure 1:**
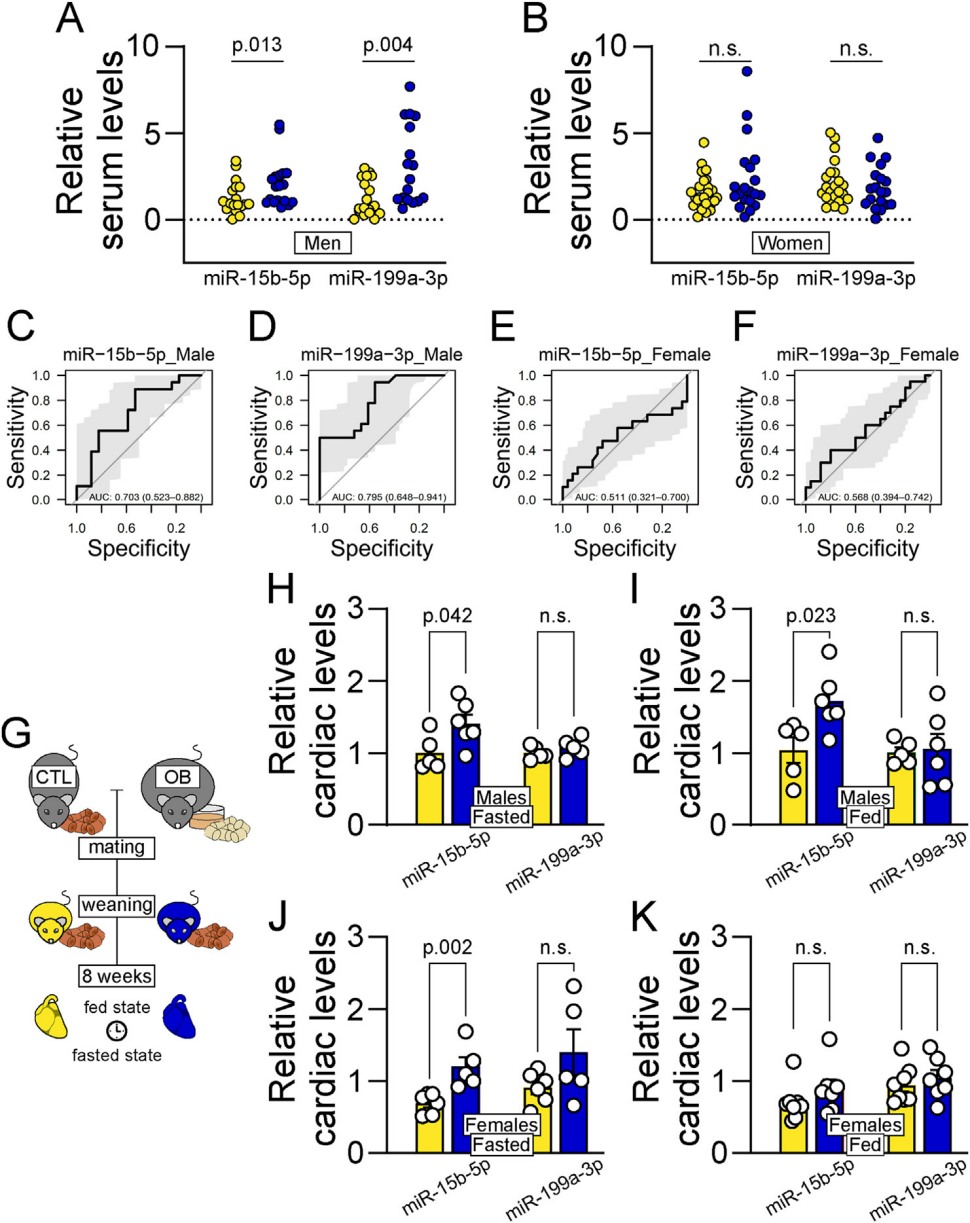
**MiR-15b-5p as a biomarker of exposure to maternal obesity.** (A-B) Quantification of serum levels of miR-15b-5p and miR-199a-3p in male (A) and female (B) patients from The Helsinki Birth Cohort Study. Yellow circles represent data from individuals born to healthy low-BMI women; Blue circles represent data from individuals born to women with high BMI. Statistical significance calculated by two-tailed Mann-Whitney exact test. Male control offspring n = 18, male obese offspring n = 18, female control offspring n = 25, female obese offspring n = 20. PCR failed data points (undetermined) are omitted for miR-199a-3p. (C-F) Receiver operating characteristic curves illustrating the diagnostic ability of miR-15b-5p (C) and miR-199a-3p (D) to predict if male patients were born to women with obesity, and the ability of miR-15b-5p (E) and miR-199a-3p (F) to predict if female patients were born to women with obesity. (G) Representative schematics of mouse model. (H-K) Relative miR-15b-5p and miR-199a-3p levels in cardiac tissue of 8-week-old fed (H) and fasted (I) male, and fed (J) and fasted (K) female offspring of dams fed a control (green bars) or a high-fat-high-sucrose diet (red bars). Statistical significance calculated by unpaired two-tailed Student t-test. Male control offspring n = 5, obese offspring n = 6. Female control fasted offspring n = 6, fed offspring n = 7; female obese fasted offspring n = 5, fed offspring n = 7. One outlier data point was removed from offspring of obese dams in panel H according to ROUT test (Q = 1 %) for cardiac miR-199a-3p levels.

To establish if the aforementioned miRNAs identified as biomarkers of coronary diseases were also linked to exposure to maternal obesity, we used a deeply phenotyped cohort, the Helsinki Birth Cohort Study, in which it has been shown that maternal BMI is positively correlated with offspring CVD [9]. A subset was selected for the current study based on pre-delivery maternal BMI (n = 42 control BMI and n = 40 high BMI). In this subset, there was a small but significant increase in maternal age in the high BMI group. Consistent with data from the whole cohort, birth weight was increased in babies born to the high BMI women (Table S2). At mean age 69 years, serum levels of both miR-15b-5p and miR-199a-3p were significantly higher in men born to mothers with higher pre-delivery BMI (Figure 1A). Differences in serum miRNA levels were not statistically significant in women (Figure 1B). Consistent with these data, receiver-operating characteristic (ROC) analysis revealed that serum miR-15b-5p (Figure 1C) and miR-199a-3p (Figure 1D) were potential biomarkers of exposure to maternal obesity in the adult male, but not female offspring (Figure 1E, F). No statistically significant correlation was observed between current offspring BMI and miR-15b-5p and miR-199a-3p serum levels (Corr. = −0.03, p = 0.8 and Corr. = 0.02, p = 0.9, n = 82). Specificity in regulation of miR-15b-5p and miR-199a-3p was evidenced by the lack of regulation of other highly abundant miRNAs, miR-451a and miR-144-3p (Fig. S2).

To investigate if the measured changes in serum miRNAs could reflect regulation in cardiac levels, we measured these miRNAs in the hearts of 8-week-old mouse offspring born to obese dams compared to age-matched control mice (Figure 1G). miR-15b-5p was significantly up-regulated in the cardiac tissue of male offspring of obese mums, but no changes in miR-199a-3p levels were observed in either the fasted (Figure 1H) or fed state (Figure 1I). miR-15b-5p was upregulated in female hearts after overnight fasting (Figure 1J), but there was no effect of maternal obesity on the expression of miR-15b-5p or miR-199a-3p in hearts of fed female offspring (Figure 1K). Using factorial ANOVA, we also observed an effect of sex on cardiac miR-15b-5p levels with males expressing significantly higher levels of the miRNA with predicted (least squares) mean expression in males = 1.38 and predicted (least squares) mean expression in females = 0.79 (p < 0.001). Mouse offspring body weight did not differ between experimental groups (male CTL = 24.3 ± 0.5, n = 16 vs male OB = 25.0 ± 0.6 n = 18 and female CTL = 21.6, ±0.9, n = 12 vs female OB = 20.7 ± 0.9, n = 11).

### 3.2 Overexpressing miR-15b-5p causes profound changes to the proteome *in vitro*

**Figure 2:**
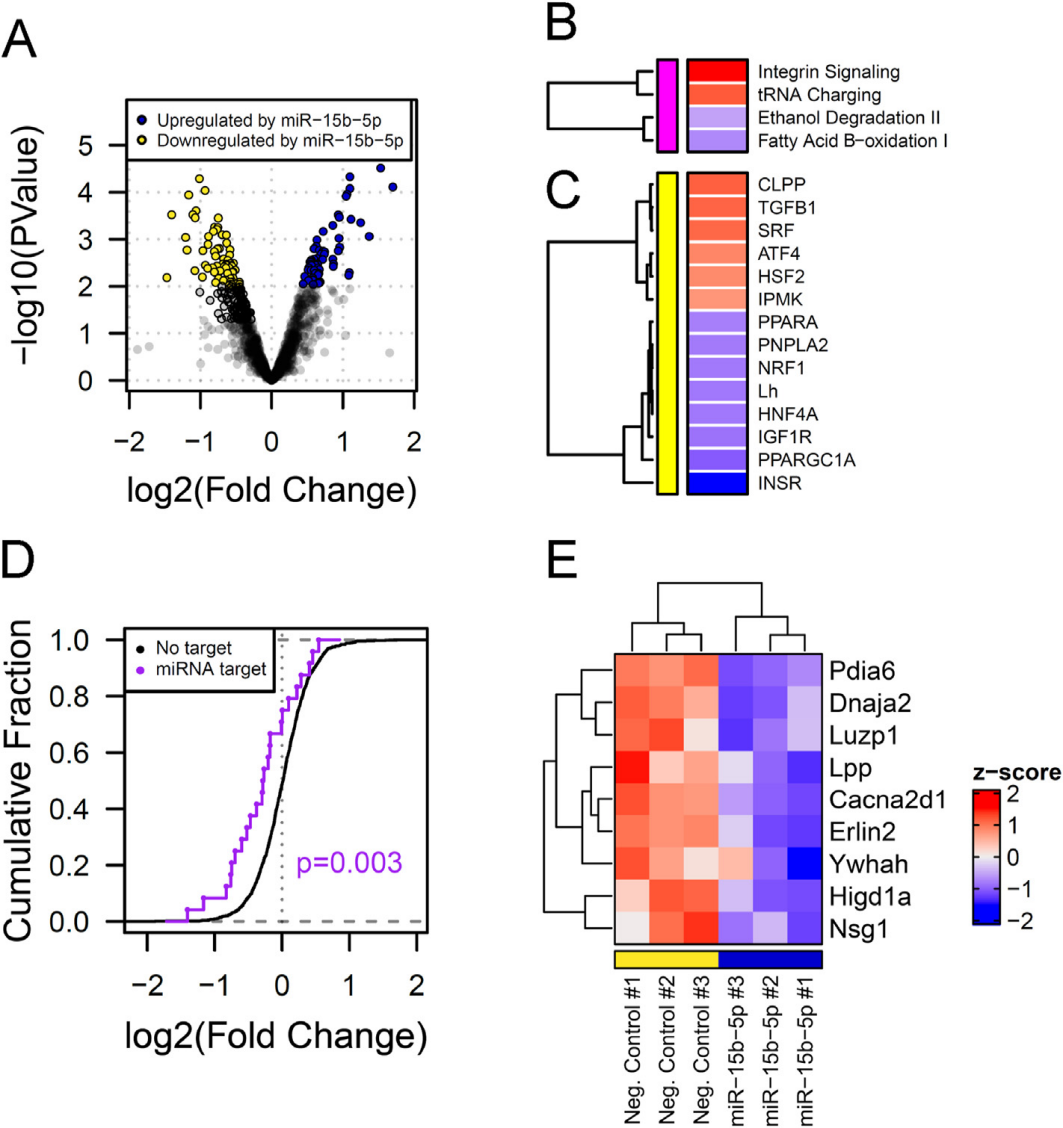
**miR-15b-5p overexpression leads to changes on the proteome of a cardiomyocyte-like cell line.** (A) Changes in protein levels in H9c2 cells transfected with miR-15b-5p mimic detected by MSMS. Coloured dots represent significantly regulated proteins (P < 0.05). (B-C) Canonical pathways' (B) and upstream regulators' (C) z-score, as predicted by IPA pathway enrichment analysis. (D) Cumulative fraction of fold change of putative targets predicted by TargetScan v7.1 in H9c2 cells transfected with a miR-15b-5p mimic compared to cells transfected with a negative control mimic. Statistical differences between distributions were calculated using two-sided Kolmogorov-Smirnov tests. (E) Z-score of protein levels in individual experiments with H9c2 cells transfected with a negative control or with a miR-15b-5p mimic. For A-E, negative control n = 3, miR-15b-5p mimic n = 3.

To explore the role of miR-15b-5p in regulating cardiac function and metabolism, and to investigate the potential consequences of its dysregulation on the cardiomyocyte, we transfected H9c2 cells with mimics of miR-15b-5p and analysed changes in the cell proteome after 24 h. We observed that increasing miR-15b-5p levels led to the dysregulation of 125 proteins [adj. p-value <0.1 (60 upregulated and 65 downregulated)] (Figure 2A). A post-hoc pathway enrichment analysis revealed that one of the main canonical pathways affected was fatty acid oxidation, which was downregulated (Figure 2B). Consistent with this finding, close examination of predicted activity of upstream regulators using IPA identified inactivation of transcription factors and receptors associated with regulation of lipid metabolism and with mitochondrial biogenesis (PPARA, NRF1, PPARGC1A) (Figure 2C). The regulation of lipid metabolism related genes was in accordance with a pathway enrichment analysis that predicted targets of miR-15b-5p enriched in fatty acid metabolism associated pathways in KEGG (Fig. S3). Levels of *in-silico* predicted targets of miR-15b-5p [high confidence prediction score according to TargetScan v7.1 (Cumulative weighted context++ score < −0.3)] were also affected. These data show a reduced cumulative fraction of fold change (Figure 2D) and nine targets being significantly downregulated (p < 0.05; six targets with adj. p-value <0.1) (Figure 2E), including a mitochondrial factor responsive to hypoxia, Higd1a [[39], [40], [41]], in H9c2 cells transfected with a miR-15b-5p mimic compared to cells transfected with a negative control mimic. To investigate whether miR15b-5p expression could be regulated during the progression of cardiovascular diseases (CVD), we exposed differentiated H9c2 cells to conditions that simulate the complex milieu experienced by cardiomyocytes during CVD, including exposure to high angiotensin II levels, starvation and oxidative stress. These factors did not lead to increased expression of miR-15b-5p (Fig. S4).

### 3.3 miR-15b-5p regulates cardiomyocyte metabolism *in vitro*

**Figure 3:**
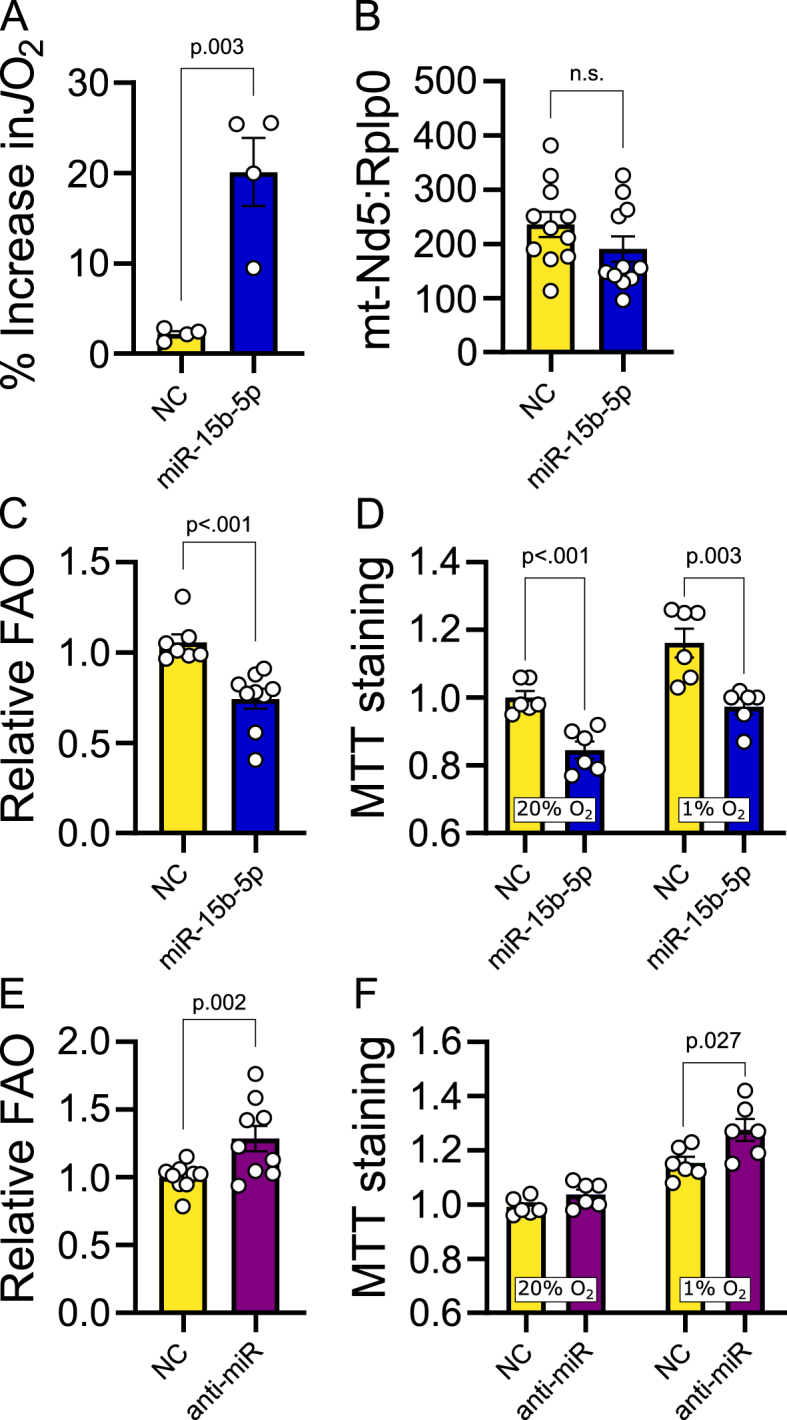
**miR-15b-5p regulates oxidative metabolism in cardiac cells**. (A) Increase in *J*O_2_ upon cytochrome c addition following digitonin permeabilization in differentiated H9c2 cells transfected with a miR-15b-5p mimic compared to cells transfected with a negative control mimic. Four iterations of this experiment were performed. Negative control n = 4, miR-15b-5p mimic n = 4. (B) mtDNA:nDNA ratio calculated by relative *Nd5*:*Rplp0* amplification in differentiated H9c2 cells transfected with a miR-15b-5p mimic compared to cells transfected with a negative control mimic. Three iterations of this experiment were performed. Negative control n = 11, miR-15b-5p mimic n = 11. (C) Estimated relative fatty acid oxidation in H9c2 cells transfected with a miR-15b-5p mimic compared to cells transfected with a negative control mimic. Three iterations of this experiment were performed. Negative control n = 7, miR-15b-5p mimic n = 9. One outlier removed from NC group (ROUT Q = 1 %). (D) Relative MTT staining in differentiated H9c2 cells cultured at standard (O_2_ = 20 %) and hypoxic conditions (O_2_ = 1 %) conditions transfected with a miR-15b-5p antisense compared to cells transfected with a negative control oligo. Negative control n = 6, miR-15b-5p mimic n = 6. (E) Estimated relative fatty acid oxidation in H9c2 cells transfected with a miR-15b-5p mimic compared to cells transfected with a negative control mimic. Three iterations of this experiment were performed. Negative control n = 9, miR-15b-5p mimic n = 9. (F) Relative MTT staining in differentiated H9c2 cells cultured at normoxic (O_2_ = 20 %) and hypoxic conditions (O_2_ = 1 %) conditions transfected with a miR-15b-5p antisense compared to cells transfected with a negative control oligo. Negative control n = 6, miR-15b-5p antisense n = 6. Figures B, C and E: statistical significance calculated by linear mixed-effects models with iteration as random effect. Figures D and F: statistical significance calculated by unpaired two-tailed Student t-test.

Consistent with the pathway enrichment analysis, we observed that overexpressing miR-15b-5p in mature H9c2 cells caused loss of outer mitochondrial membrane stability after permeabilization with digitonin (Figure 3A) without significantly affecting mitochondrial DNA: nuclear DNA ratio (Figure 3B). Over-expression of the miRNA also promoted a reduction in fatty acid oxidation (Figure 3C) and led to an overall impairment in cell viability (as assessed by MTT assay) both under normoxic and hypoxic conditions (Figure 3D). Conversely, transfection of cardiomyocytes with an antisense oligo to miR-15b-5p increased fatty acid oxidation (Figure 3E) and improved cellular viability under hypoxic conditions (Figure 3F).

### 3.4 Isolated hearts from offspring of obese dams are more susceptible to ischemia-reperfusion associated damage and miR-15b-5p is released in response to ischaemia

**Figure 4:**
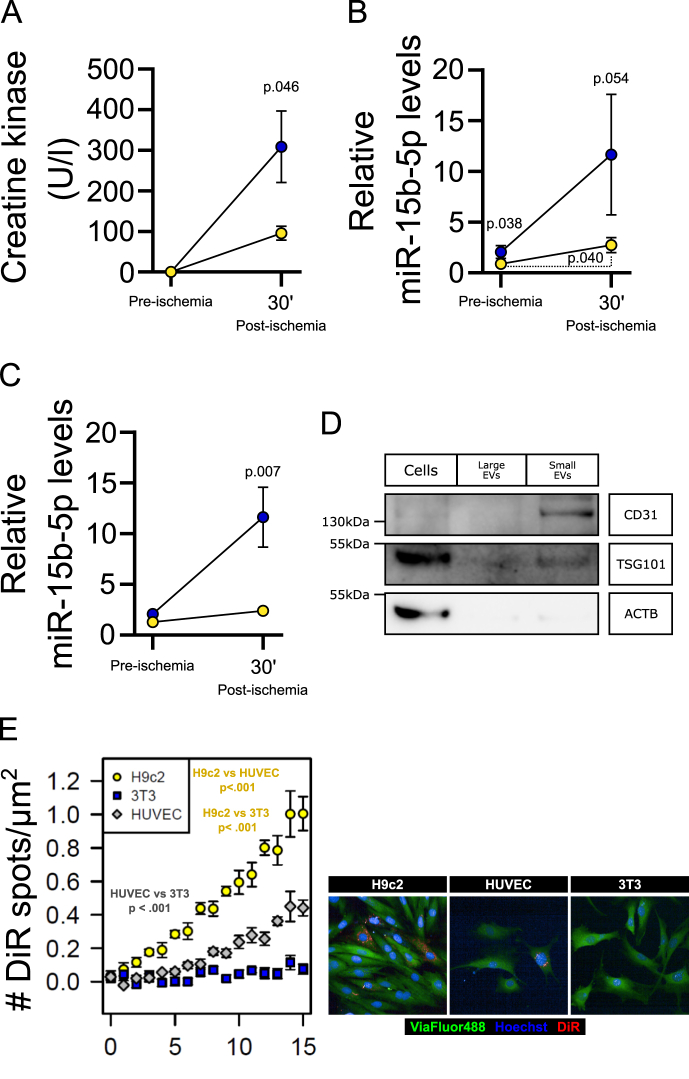
**Hearts from the offspring of obese dams are more susceptible to ischemic damage.** (A) Creatine kinase levels in Langendorff perfusates of *ex vivo* hearts from offspring of control (yellow circles) and obese (blue circles) dams pre and post ischemia-reperfusion. Statistical significance calculated by unpaired one-tailed Student t-test. Control offspring n = 4, obese offspring n = 6. (B) Levels of miR-15b-5p in Langendorff perfusates of *ex vivo* hearts from offspring of control (yellow circles) and obese (blue circles) dams before (pre) and post ischemia-reperfusion. Statistical significance between groups calculated using one-tailed unpaired Student t-test; Statistical significance between different time points calculated using two-tailed paired Student t-test. Control offspring n = 8, obese offspring n = 6. (C) Levels of miR-15b-5p in EVs isolated from Langendorff perfusates of *ex vivo* hearts from offspring of control (yellow circles) and obese (blue circles) dams before (pre) and post ischemia-reperfusion. Statistical significance between groups calculated using two-tailed unpaired Student t-test. Control offspring n = 7, obese offspring n = 5. (D) Validation of extracellular vesicle fractioning by Western Blotting. (E) Number of DiR fluorescent spots detected within cells adjusted for cell area, with representative image at time point 15. Statistical significance between cells across time calculated using mixed-effects model. n = 3 per cell line. One outlier data point was removed from offspring of obese dams pre-ischemia in panel C according to ROUT test (Q = 1 %) for miR-15b-5p levels.

We then tested if hearts of male offspring from obese dams have increased susceptibility to the effects of ischaemia-reperfusion using a modified Langendorff set up. Under baseline conditions there were no differences in release of creatine kinase (CK, a marker of cardiac damage) from spontaneously beating hearts from the offspring of control or obese dams (Figure 4A). However, inducing a short period of ischemia followed by reperfusion led to significant release of cardiac CK. The hearts from offspring of obese dams released greater levels of CK compared to controls, indicating an increased susceptibility to ischemia-reperfusion damage as a consequence of *in utero* exposure to maternal obesity (Figure 4A). Levels of miR-15b-5p released into cardiac perfusates also increased following ischaemia/reperfusion, with perfusates collected from offspring of obese dams showing the highest levels of miR-15b-5p both during baseline and following ischemia-reperfusion (Figure 4B), an effect not observed with other miRNAs such as the highly expressed cardiac miRNAs miR-144-3p and miR-451a (Fig. S5), suggesting specific release of miR-15b-5p. Fractional separation of perfusates by sequential ultracentrifugation indicated that released miR-15b-5p was contained within small extracellular vesicles, which was increased in post-ischemia/reperfusion perfusates from offspring of obese dams (Figure 4C). Extracellular vesicle fractioning was validated using Nanoparticle tracker analysis, transmission electron microscopy and Western blotting. Between 2.2e6 and 6.05e7 particles, with an average size ranging from 99 to 144 nm were purified into the EV fraction from each sample (Figs. S6A and 6B). No differences in small EV sizes were observed between groups (CTL mean = 110.3 ± 3.5 nm, n = 6; OB mean = 116.0 ± 8.0 nm, n = 5). The cellular fraction was enriched for actin and TSG101 but did not show expression of the EV specific marker CD31. In contrast, the EV fraction contained CD31 and TSG101 and did not contain the cellular marker actin (Figure 4D). To explore which cell types express miR-15b-5p and therefore could be the potential source of released miR-15b-5p from the heart, we isolated fetal cardiomyocytes from other cell types within the heart (i.e.: endothelial cells and fibroblasts). Both the cardiomyocyte fraction and non-cardiomyocyte fraction expressed high levels of miR-15b-5p therefore both could be the potential source of the released miRNA (cardiomyocytes PCR Cq = 28.8 ± 0.4 and non-cardiomyocytes PCR Cq = 24.6 ± 0.5).

To identify potential target tissues of extracellular vesicles released by the isolated hearts, we screened the ability of isolated cardiac perfusate EVs to fuse and be taken up by the cardiomyocyte-like cells (H9c2), endothelial-like cells (HUVEC) and fibroblasts (undifferentiated 3T3 cells). The cardiomyocytes and endothelial-like cells both took up the cardiac EVs, whereas the fibroblasts did not (Figure 4E).

## 4 Discussion

It is established that maternal obesity during pregnancy programs an increased cardiovascular risk in the adult offspring [[6], [7], [8]], however underlying mechanisms remain poorly defined, precluding identification of disease biomarkers and of preventative therapy. Adopting an integrative approach, combining measurements in humans with *in vivo* and isolated organ experiments in mice and analysis in cell systems, here we show that: 1) increased gestational BMI is associated with increased serum miRNA-15b levels in human offspring (independent of current offspring BMI) and cardiac miRNA-15b expression in mice offspring; 2) overexpressing miR-15b-5p causes changes to a cardiomyocyte-like cell line proteome *in vitro*; 3) miR-15b-5p regulates cardiomyocyte metabolism *in vitro*; 4) isolated hearts from offspring of obese dams are more susceptible to ischemia-reperfusion damage, as measured by cardiac CK release and 5) miR-15b-5p is released from mouse hearts basally and increased in response to ischemia. This release is magnified in adult mouse offspring born to obese dams. Therefore, the data support the inter-related hypotheses tested, identifying both a potential mechanism linking fetal exposure to maternal obesity and overnutrition with adverse long-term cardiovascular metabolism and health, as well as a candidate biomarker that can be measured in human serum.

MiRNAs are promising biomarkers of CVD [21], possessing the main required properties including: being detectable in easily accessible human fluids (e.g., blood, serum, saliva, urine), being present before disease pathology thus predicting the future onset of diseases in high-risk individuals and being stably measurable in the circulation. Furthermore, they can be secreted by diseased tissues; indeed, unique signatures of circulating miRNAs have been associated with a wide range of human pathologies [42,43]. The current data show the novel findings that maternal obesity/adiposity is associated with a sex-dependent increase in miRNA-15b in both humans and mice. Importantly, miRNA-15b-5p was not associated with current BMI or adiposity in either the mouse model or in humans. This miRNA belongs to the miRNA-15b/16-2 cluster, which is highly conserved among mammalian species [44], suggesting an important regulatory role throughout evolution. miR-15b-5p is predicted to exert regulatory control over several genes associated with metabolic, proliferative and signaling pathways in both humans and rodents. An important metabolic target, ACOX1, is essential in the initial steps of fatty acid oxidation, indicating that miR-15b-5p could play an important role as a modulator of lipid metabolism. This conveys its role in signaling pathways controlling cellular metabolism, posing miR-15b-5p as an important factor in the development of metabolic diseases.

Given that it is known that maternal BMI correlates positively with premature death from cardiovascular diseases [5] and with CVD in the offspring in the Helsinki Birth Cohort Study [9], we suggest that miRNA-15b levels is predictive of increased risk of CVD in male individuals. Our current study was not powered to address cardiovascular outcomes therefore unfortunately we could not test associations with cardiovascular outcome. Although the tissue source of the miR-15b-5p in human serum is unknown, our studies in mice show that a) maternal obesity leads to an increased programmed expression of this miRNA in the heart, b) it is released from the heart, and c) its cardiac release is greater both under basal conditions and after ischemia-reperfusion in adult offspring of obese dams. Therefore, this is consistent with miR-15b-5p being both a marker of cardiac ischemic damage and of exposure to a suboptimal *in utero* environment that increases heart disease risk in the adult offspring.

Circulating miRNAs can be contained within EVs. These EVs represent novel routes of intercellular/interorgan communication, with the miRNAs contained within them acting as a means by which gene expression in one tissue can influence the physiology of another [45]. Recent studies of cardiac-derived EVs show that they can shuttle contents, including miRNAs, to neighboring cells or distant tissues to control homeostasis [46,47]. More importantly, metabolic disturbances and ischemic events change the miRNA cargo from cardiomyocyte-derived EVs, affecting their ability to regulate several local and distant processes, including angiogenesis, myocardial injury, and pulmonary inflammatory responses [46,48,49]. Early studies identified that hypoxic conditions increase the secretion of cardiac EVs and alter their cargo [50]. Later studies demonstrated that miRNAs contained within cardiac fibroblast can be delivered to cardiomyocytes and contribute to the control of hypertrophy [51,52] and protect against ischemia-reperfusion injury [53]. However, thus far, these studies have not addressed the potential of the miRNA EV cargo to regulate cell metabolism. In our study, using freshly isolated mouse hearts in a Langendorff preparation, we observed that a significant fraction of miR-15b-5p was released from the heart in small EVs. In contrast to larger vesicles, these are mostly composed of actively secreted vesicles like exosomes, providing evidence for a potential role for this miRNA as a paracrine and endocrine signaling molecule. The destination of these cardiac EVs is currently unknown. However, our *in vitro* studies suggest that they have the potential to fuse with and be taken up by both cardiomyocytes and endothelial cells. These findings provide novel insight into how increased cardiac levels of miR-15b-5p could impact on cardiac metabolism and function. Up-regulation of miRNA-15b has been reported in human failing hearts, as well as in various human and mouse models of cardiac hypertrophy [[54], [55], [56], [57]], and it has been associated with cardiomyocyte survival [57], proliferation [58] and ATP production [59]. However, causal relationships between these effects have not been demonstrated. We show that overexpressing miR-15b-5p causes profound changes to the cardiac proteome and that these changes are accompanied by outer mitochondrial membrane instability and impaired fatty acid oxidative metabolism, both of which would be expected to negatively impact on cardiac function.

## 5 Conclusions

These novel findings suggest that serum levels of miRNA-15b in middle-aged people could be used to identify individuals that experienced a suboptimal *in utero* environment. More importantly, our experimental data suggest that elevated miRNA-15b levels may be used to detect individuals at increased risk of CVD and who would therefore benefit most from targeted intervention strategies, such as anti-sense technology, before pathologies develop and the disease process becomes irreversible. These potential therapeutic strategies are long-term possibilities that demand further investigation and safety assessments.

We recognize that the current study has some limitations: a) The cell lines utilized are immortalized cells that do not retain all of the original characteristics of the source cells. To address this, we cultured them in conditions that contributed to the maintenance of the original phenotype or, in the case of 3T3 cells, to maintain their fibroblast multipotent phenotype. b) The source of circulatory miR-15b-5p in humans cannot be determined using the currently available technologies and methods. Therefore, we cannot rule out the possibility of organs and tissues other than the heart contributing to the source of this miRNA in the human circulation. c) The sample size of the human studies prevented us from drawing direct associations between circulating miR-15b-5p and CVD or cardiac events risk in the population. Nevertheless, the dysregulation of cardiac miR-15b-5p leading to increased vulnerability to ischaemic damage in our experimental studies provides a potential underlying mechanism by which exposure to an obesogenic environment *in utero* may lead to increased risk of cardiovascular disease.

In conclusion, we have identified miR-15b-5p, a maternally programmed miRNA, as an important regulator of cardiac cellular metabolism and viability and a potential biomarker of increased cardiovascular risk in the offspring exposed to maternal obesity during development. These findings represent an important step towards the mechanistic understanding of early life programming of cardiac diseases and offer a new potential target for potential oligonucleotide-based therapies. The evidence that part of the circulating cardiac derived miR-15b-5p is contained in EVs raises the exciting possibility that it represents a novel route of communication between an ischaemic heart and other tissues.

## Funding

This work was supported by the 10.13039/501100000274British Heart Foundation [PG/14/20/3076, RG/17/12/33167]; the Medical Research Council [MRC_MC_UU_00014/4]. EL and BDT were the recipients of two British Heart Foundation scholarships (FS/12/64/30001 and FS/4yPhD/F/20/34124C, respectively). JAF was the recipient of two São Paulo State Research Foundation (FAPESP) fellowships (2014/17012-4 and 2017/03525-8). SEO, AJM and DG are members of the BHF Cambridge Centre for Research Excellence (RE/18/1/34212). AC-C is the recipient of a Beca Postdoctorado en el Extranjero fellowship (Agencia Nacional de Investigación y Desarrollo - ANID, 74220049).

## CRediT authorship contribution statement

**Lucas C. Pantaleão:** Data curation, Formal analysis, Investigation, Methodology, Visualization, Writing – original draft, Writing – review & editing. **Elena Loche:** Conceptualization, Data curation, Formal analysis, Investigation, Writing – original draft. **Denise S. Fernandez-Twinn:** Investigation. **Laura Dearden:** Investigation. **Adriana Córdova-Casanova:** Formal analysis, Investigation. **Clive Osmond:** Formal analysis, Investigation. **Minna K. Salonen:** Formal analysis, Investigation. **Eero Kajantie:** Formal analysis, Investigation. **Youguo Niu:** Investigation. **Juliana de Almeida-Faria:** Investigation. **Benjamin D. Thackray:** Investigation. **Tuija M. Mikkola:** Formal analysis. **Dino A. Giussani:** Data curation, Formal analysis. **Andrew J. Murray:** Formal analysis, Investigation. **Martin Bushell:** Formal analysis. **Johan G. Eriksson:** Data curation, Formal analysis, Investigation, Project administration, Resources, Supervision. **Susan E. Ozanne:** Conceptualization, Data curation, Formal analysis, Funding acquisition, Project administration, Resources, Writing – original draft, Writing – review & editing.

## Declaration of competing interest

The authors have declared that no conflict of interest exists.

## Data Availability

Data will be made available on request.
