# Supplementary figures and images for: Programming of cardiac metabolism by miR-15b-5p, a miRNA released in cardiac extracellular vesicles following ischemia-reperfusion injury
Source: Mol Metab. 2024 Jan 11;80:101875. doi: 10.1016/j.molmet.2024.101875 (PMC10832484; doi:10.1016/j.molmet.2024.101875)

# A

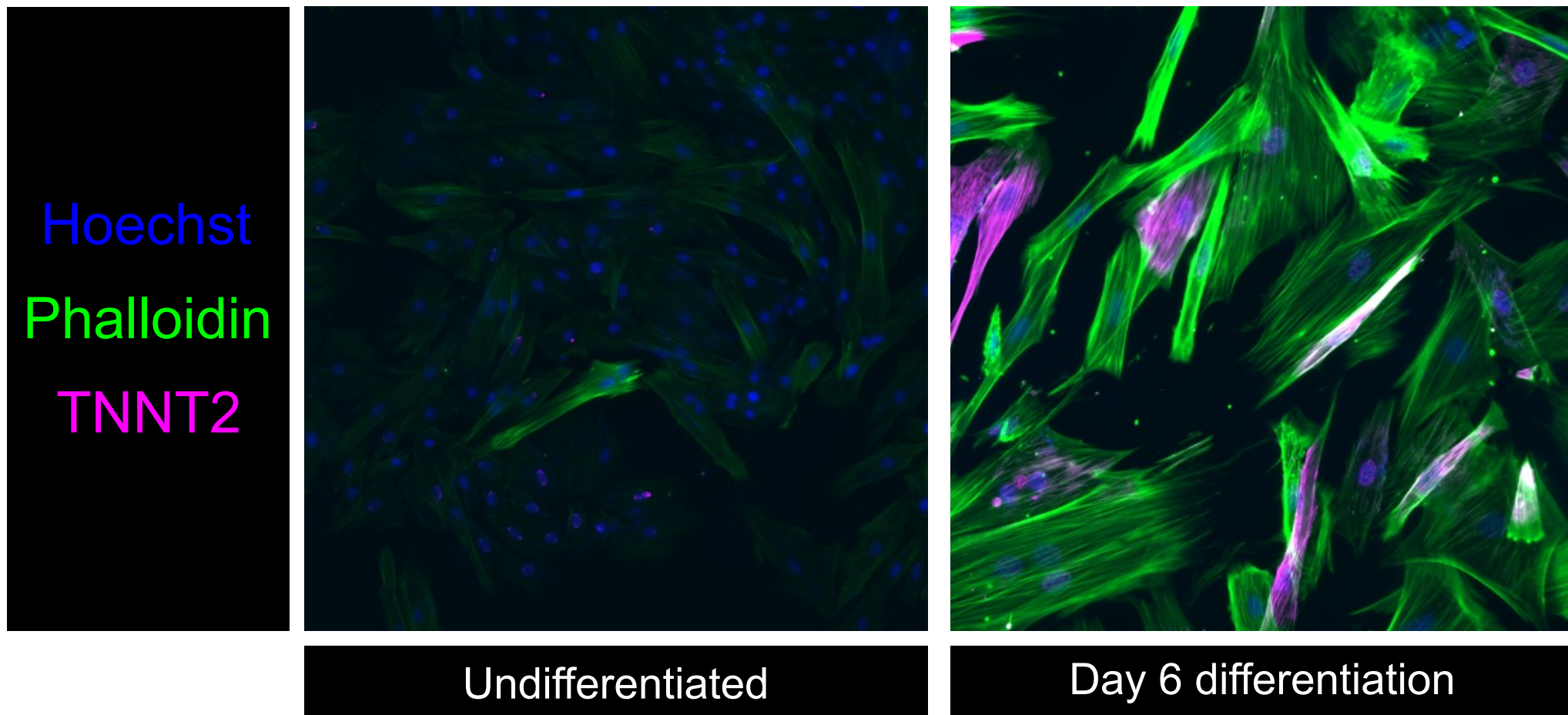

# B

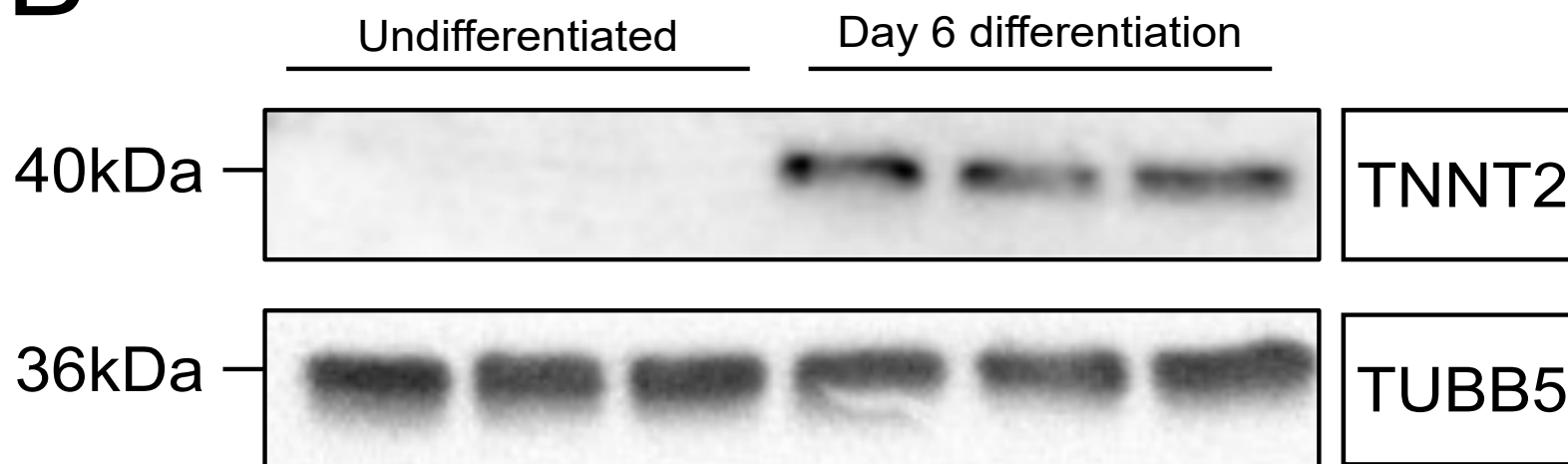

Supplement: Multimedia component 1 [file mmc1.pdf]

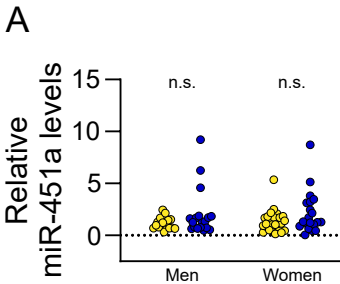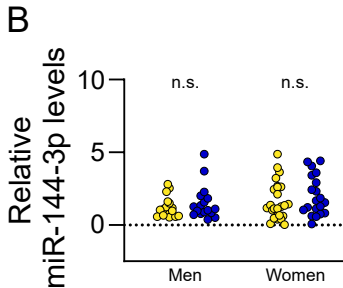

Supplement: Multimedia component 2 [file mmc2.pdf]

## Human

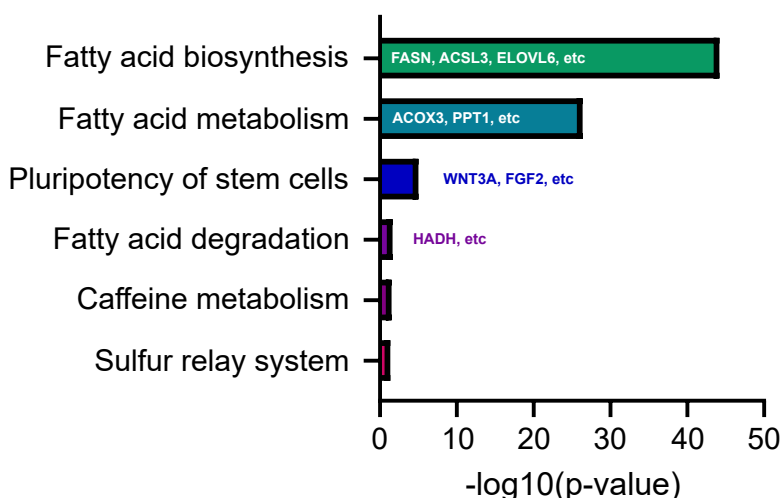

## Mouse

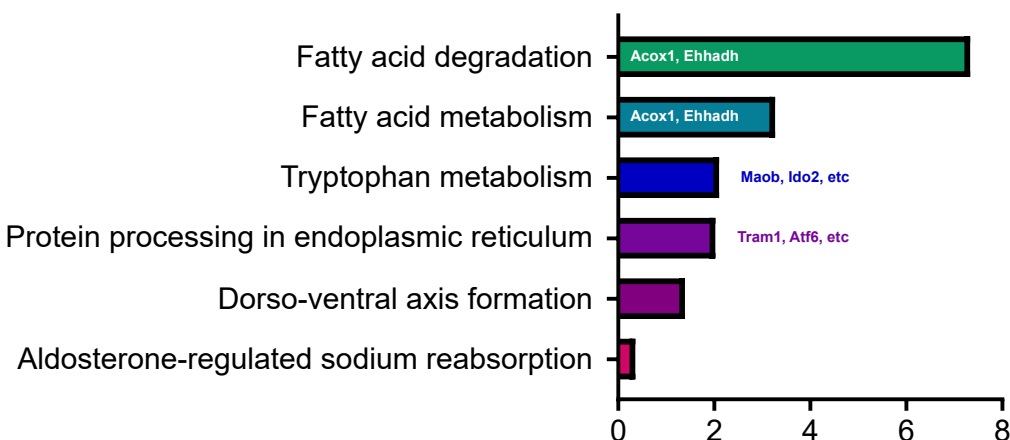

## Rat

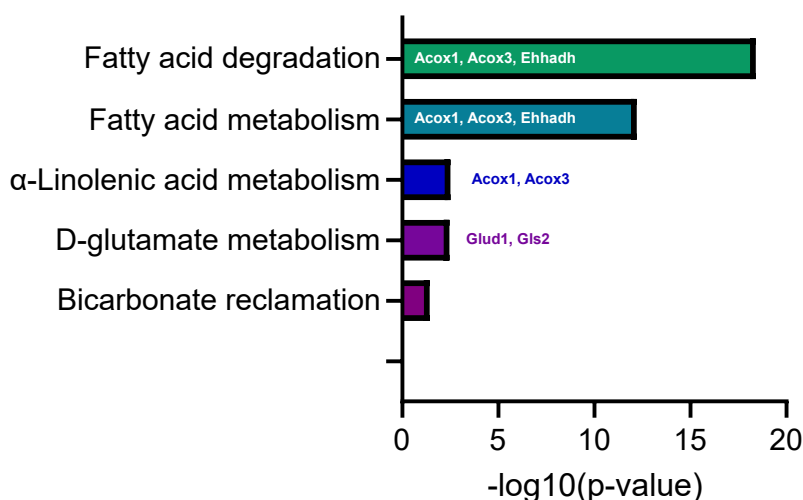

Supplement: Multimedia component 3 [file mmc3.pdf]

Relative miR-15b-5p  
levels

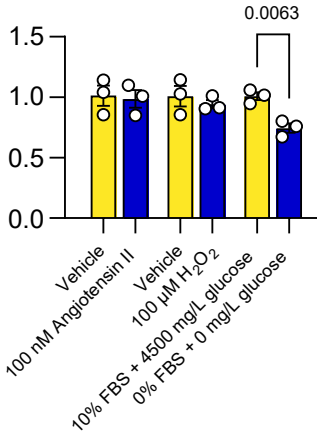

Supplement: Multimedia component 4 [file mmc4.pdf]

A

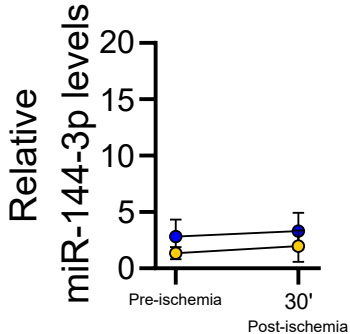

B

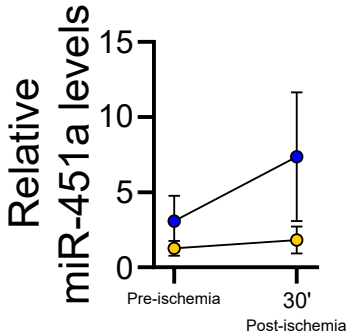

Supplement: Multimedia component 5 [file mmc5.pdf]
