# Supplementary material for: Programming of cardiac metabolism by miR-15b-5p, a miRNA released in cardiac extracellular vesicles following ischemia-reperfusion injury
Source: Mol Metab. 2024 Jan 11;80:101875. doi: 10.1016/j.molmet.2024.101875 (PMC10832484; doi:10.1016/j.molmet.2024.101875)
Supplement: Multimedia component 6 [file mmc6.pdf]

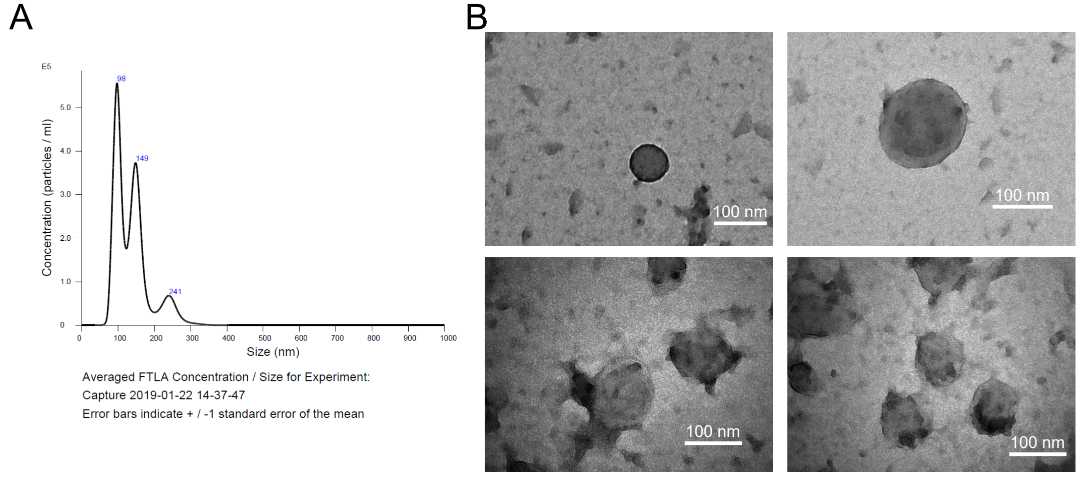
A
B
E5
98
149
241
5.0
4.0
3.0
2.0
1.0
0
Concentration (particles / ml)
0
100
200
300
400
500
600
700
800
900
1000
Size (nm)
Averaged FTLA Concentration / Size for Experiment:
Capture 2019-01-22 14-37-47
Error bars indicate + / -1 standard error of the mean
100 nm
100 nm
100 nm
100 nm
