# Supplementary material for: Programming of cardiac metabolism by miR-15b-5p, a miRNA released in cardiac extracellular vesicles following ischemia-reperfusion injury
Source: Mol Metab. 2024 Jan 11;80:101875. doi: 10.1016/j.molmet.2024.101875 (PMC10832484; doi:10.1016/j.molmet.2024.101875)
Supplement: Multimedia component 7 [file mmc7.docx]

Table S2. Differences between maternal and offspring low and high BMI groups. Helsinki Birth Cohort Study.

| Measurement | Control mothers (n=42); Offspring M=17, F=25 | | High BMI mothers (n=40) Offspring M=19, F=21 | | p-value, adjusted for sex. |
| --- | --- | --- | --- | --- | --- |
|  | Mean | SD | Mean | SD |  |
|  |  |  |  |  |  |
| Mother |  |  |  |  |  |
|  |  |  |  |  |  |
| Age at delivery (years) | 27.9 | 5.2 | 30.9 | 4.7 | 0.005 |
| Parity | 1.9 | 1.1 | 2.2 | 1.3 | 0.2 |
| Height (cm) | 157.0 | 6.6 | 156.1 | 4.6 | 0.7 |
| Weight (kg) | 64.6 | 5.8 | 79.2 | 7.3 | <0.001 |
| BMI (kg/m^2^) | 26.2 | 0.4 | 32.5 | 2.6 | <0.001 |
|  |  |  |  |  |  |
|  |  |  |  |  |  |
| Newborn offspring |  |  |  |  |  |
|  |  |  |  |  |  |
| Weight (g) | 3287.5 | 483.1 | 3605.1 | 3605.2 | 0.007 |
|  |  |  |  |  |  |
| Adult offspring |  |  |  |  |  |
|  |  |  |  |  |  |
| Age (years) | 69.9 | 1.8 | 68.4 | 2.4 | 0.002 |
| Height (cm) | 166.3 | 9.4 | 168.2 | 8.8 | 0.5 |
| Weight (kg) | 73.8 | 12.4 | 77.9 | 12.0 | 0.1 |
| BMI (kg/m^2^) | 26.6 | 3.8 | 27.6 | 3.9 | 0.4 |
| Waist circumference (cm) | 90.5 | 11.9 | 95.5 | 11.9 | 0.09 |
| Percentage fat (%) | 29.3 | 8.6 | 29.4 | 8.0 | 0.5 |
|  |  |  |  |  |  |
|  |  |  |  |  |  |
