# Supplementary material for: Programming of cardiac metabolism by miR-15b-5p, a miRNA released in cardiac extracellular vesicles following ischemia-reperfusion injury
Source: Mol Metab. 2024 Jan 11;80:101875. doi: 10.1016/j.molmet.2024.101875 (PMC10832484; doi:10.1016/j.molmet.2024.101875)
Supplement: Multimedia component 8 [file mmc8.docx]

Table S3. qPCR primer sequences

| Primer | Sequence |
| --- | --- |
| Nd5 (forward) | AGCAATCTGTGCTCTCACCC |
| Nd5 (reverse) | GGCCTAGTTGGCTGGATGTT |
| Rplp0 (forward) | GGAACGTGGGCTTTGTGTTC |
| Rplp0 (reverse) | ACCTTATTGGCCAGCAGCAT |
