# Supplementary material for: Programming of cardiac metabolism by miR-15b-5p, a miRNA released in cardiac extracellular vesicles following ischemia-reperfusion injury
Source: Mol Metab. 2024 Jan 11;80:101875. doi: 10.1016/j.molmet.2024.101875 (PMC10832484; doi:10.1016/j.molmet.2024.101875)
Supplement: Multimedia component 9 [file mmc9.docx]

Protein IDs Majority protein IDs Peptide counts (all) Peptide counts (razor+unique) Peptide counts (unique) Protein names Gene names Fasta headers Number of proteins Peptides Razor + unique peptides Unique peptides Peptides 152p_1 Peptides 152p_2 Peptides 152p_3 Peptides 152p_4 Peptides 152p_5 Peptides 152p_6 Razor + unique peptides 152p_1 Razor + unique peptides 152p_2 Razor + unique peptides 152p_3 Razor + unique peptides 152p_4 Razor + unique peptides 152p_5 Razor + unique peptides 152p_6 Unique peptides 152p_1 Unique peptides 152p_2 Unique peptides 152p_3 Unique peptides 152p_4 Unique peptides 152p_5 Unique peptides 152p_6 Sequence coverage [%] Unique + razor sequence coverage [%] Unique sequence coverage [%] Mol. weight [kDa] Sequence length Sequence lengths Fraction average Fraction 1 Fraction 2 Fraction 3 Fraction 4 Fraction 5 Fraction 6 Fraction 7 Fraction 8 Fraction 9 Fraction 10 Fraction 11 Fraction 12 Q-value Score Identification type 152p_1 Identification type 152p_2 Identification type 152p_3 Identification type 152p_4 Identification type 152p_5 Identification type 152p_6 Ratio M/L Ratio M/L normalized Ratio M/L variability [%] Ratio M/L count Ratio M/L iso-count Ratio M/L type Ratio H/L Ratio H/L normalized Ratio H/L variability [%] Ratio H/L count Ratio H/L iso-count Ratio H/L type Ratio H/M Ratio H/M normalized Ratio H/M variability [%] Ratio H/M count Ratio H/M iso-count Ratio H/M type Ratio M/L 152p_1 Ratio M/L 152p_2 Ratio M/L 152p_3 Ratio M/L 152p_4 Ratio M/L 152p_5 Ratio M/L 152p_6 Ratio M/L normalized 152p_1 Ratio M/L normalized 152p_2 Ratio M/L normalized 152p_3 Ratio M/L normalized 152p_4 Ratio M/L normalized 152p_5 Ratio M/L normalized 152p_6 Ratio M/L variability [%] 152p_1 Ratio M/L variability [%] 152p_2 Ratio M/L variability [%] 152p_3 Ratio M/L variability [%] 152p_4 Ratio M/L variability [%] 152p_5 Ratio M/L variability [%] 152p_6 Ratio M/L count 152p_1 Ratio M/L count 152p_2 Ratio M/L count 152p_3 Ratio M/L count 152p_4 Ratio M/L count 152p_5 Ratio M/L count 152p_6 Ratio M/L iso-count 152p_1 Ratio M/L iso-count 152p_2 Ratio M/L iso-count 152p_3 Ratio M/L iso-count 152p_4 Ratio M/L iso-count 152p_5 Ratio M/L iso-count 152p_6 Ratio M/L type 152p_1 Ratio M/L type 152p_2 Ratio M/L type 152p_3 Ratio M/L type 152p_4 Ratio M/L type 152p_5 Ratio M/L type 152p_6 Ratio H/L 152p_1 Ratio H/L 152p_2 Ratio H/L 152p_3 Ratio H/L 152p_4 Ratio H/L 152p_5 Ratio H/L 152p_6 Ratio H/L normalized 152p_1 Ratio H/L normalized 152p_2 Ratio H/L normalized 152p_3 Ratio H/L normalized 152p_4 Ratio H/L normalized 152p_5 Ratio H/L normalized 152p_6 Ratio H/L variability [%] 152p_1 Ratio H/L variability [%] 152p_2 Ratio H/L variability [%] 152p_3 Ratio H/L variability [%] 152p_4 Ratio H/L variability [%] 152p_5 Ratio H/L variability [%] 152p_6 Ratio H/L count 152p_1 Ratio H/L count 152p_2 Ratio H/L count 152p_3 Ratio H/L count 152p_4 Ratio H/L count 152p_5 Ratio H/L count 152p_6 Ratio H/L iso-count 152p_1 Ratio H/L iso-count 152p_2 Ratio H/L iso-count 152p_3 Ratio H/L iso-count 152p_4 Ratio H/L iso-count 152p_5 Ratio H/L iso-count 152p_6 Ratio H/L type 152p_1 Ratio H/L type 152p_2 Ratio H/L type 152p_3 Ratio H/L type 152p_4 Ratio H/L type 152p_5 Ratio H/L type 152p_6 Ratio H/M 152p_1 Ratio H/M 152p_2 Ratio H/M 152p_3 Ratio H/M 152p_4 Ratio H/M 152p_5 Ratio H/M 152p_6 Ratio H/M normalized 152p_1 Ratio H/M normalized 152p_2 Ratio H/M normalized 152p_3 Ratio H/M normalized 152p_4 Ratio H/M normalized 152p_5 Ratio H/M normalized 152p_6 Ratio H/M variability [%] 152p_1 Ratio H/M variability [%] 152p_2 Ratio H/M variability [%] 152p_3 Ratio H/M variability [%] 152p_4 Ratio H/M variability [%] 152p_5 Ratio H/M variability [%] 152p_6 Ratio H/M count 152p_1 Ratio H/M count 152p_2 Ratio H/M count 152p_3 Ratio H/M count 152p_4 Ratio H/M count 152p_5 Ratio H/M count 152p_6 Ratio H/M iso-count 152p_1 Ratio H/M iso-count 152p_2 Ratio H/M iso-count 152p_3 Ratio H/M iso-count 152p_4 Ratio H/M iso-count 152p_5 Ratio H/M iso-count 152p_6 Ratio H/M type 152p_1 Ratio H/M type 152p_2 Ratio H/M type 152p_3 Ratio H/M type 152p_4 Ratio H/M type 152p_5 Ratio H/M type 152p_6 Sequence coverage 152p_1 [%] Sequence coverage 152p_2 [%] Sequence coverage 152p_3 [%] Sequence coverage 152p_4 [%] Sequence coverage 152p_5 [%] Sequence coverage 152p_6 [%] Intensity Intensity L Intensity M Intensity H Intensity 152p_1 Intensity L 152p_1 Intensity M 152p_1 Intensity H 152p_1 Intensity 152p_2 Intensity L 152p_2 Intensity M 152p_2 Intensity H 152p_2 Intensity 152p_3 Intensity L 152p_3 Intensity M 152p_3 Intensity H 152p_3 Intensity 152p_4 Intensity L 152p_4 Intensity M 152p_4 Intensity H 152p_4 Intensity 152p_5 Intensity L 152p_5 Intensity M 152p_5 Intensity H 152p_5 Intensity 152p_6 Intensity L 152p_6 Intensity M 152p_6 Intensity H 152p_6 Only identified by site Reverse Potential contaminant id Peptide IDs Peptide is razor Mod. peptide IDs Evidence IDs MS/MS IDs Best MS/MS Oxidation (M) site IDs Oxidation (M) site positions

A0A096MIS5;Q5PQM0 A0A096MIS5;Q5PQM0 1;1 1;1 1;1 Transmembrane protein 168 Tmem168 tr|A0A096MIS5|A0A096MIS5_RAT Transmembrane protein 168 OS=Rattus norvegicus OX=10116 GN=Tmem168 PE=1 SV=1;sp|Q5PQM0|TM168_RAT Transmembrane protein 168 OS=Rattus norvegicus OX=10116 GN=Tmem168 PE=1 SV=1 2 1 1 1 1 0 0 1 1 1 1 0 0 1 1 1 1 0 0 1 1 1 2.2 2.2 2.2 78.886 697 697;697 1 5 0.0029659 2.2901 By MS/MS By MS/MS By MS/MS By MS/MS 0.41307 0.8578 25.641 4 3 Median 0.56473 1.0757 8.8875 4 3 Median 1.5939 1.265 26.03 4 3 Median NaN NaN NaN 0.26062 0.43184 0.41307 NaN NaN NaN 0.56894 1.0444 0.8578 NaN NaN NaN NaN NaN 3.6276 0 0 0 1 1 2 0 0 0 1 0 2 Median Median Median Median Median Median NaN NaN NaN 0.51306 0.49065 0.65838 NaN NaN NaN 1.0386 1.0126 1.173 NaN NaN NaN NaN NaN 7.2909 0 0 0 1 1 2 0 0 0 1 0 2 Median Median Median Median Median Median NaN NaN NaN 1.9686 0.96381 1.5939 NaN NaN NaN 1.4847 0.81373 1.265 NaN NaN NaN NaN NaN 5.2209 0 0 0 1 1 2 0 0 0 1 0 2 Median Median Median Median Median Median 2.2 0 0 2.2 2.2 2.2 30400000 18134000 4715000 7551100 2040400 2040400 0 0 0 0 0 0 0 0 0 0 8504600 5246900 723930 2533800 9082400 4979100 2028900 2074400 10773000 5867800 1962200 2943000 0 5224 True 5501 47394;47395;47396;47397;47398 68232;68233;68234;68235;68236 68234

A2VD14;A0A096MIV5;F1M8H5;A0A096MJ15 A2VD14;A0A096MIV5;F1M8H5 3;3;2;1 3;3;2;1 3;3;2;1 Abcf2 tr|A2VD14|A2VD14_RAT ATP-binding cassette subfamily F member 2 OS=Rattus norvegicus OX=10116 GN=Abcf2 PE=1 SV=1;tr|A0A096MIV5|A0A096MIV5_RAT ATP-binding cassette subfamily F member 2 OS=Rattus norvegicus OX=10116 GN=Abcf2 PE=1 SV=1;tr|F1M8H5|F1M8H5_RAT ATP 4 3 3 3 2 2 1 1 0 1 2 2 1 1 0 1 2 2 1 1 0 1 4.8 4.8 4.8 71.426 624 624;627;434;251 4.43 4 3 0 4.2573 By MS/MS By MS/MS By MS/MS By MS/MS By MS/MS 0.453 1.6177 57.947 6 3 Median 0.71115 1.7508 27.799 6 3 Median 1.1146 0.82385 44.924 6 3 Median 0.49723 0.61082 0.33421 1.3246 NaN 0.3863 1.8428 1.8849 1.0381 4.6916 NaN 1.0475 NaN 40.048 NaN NaN NaN NaN 1 2 1 1 0 1 0 2 0 1 0 0 Median Median Median Median Median Median 0.6952 0.53222 0.87974 0.72747 NaN 0.67441 1.6681 1.3669 2.0792 1.8376 NaN 1.6036 NaN 50.007 NaN NaN NaN NaN 1 2 1 1 0 1 0 2 0 1 0 0 Median Median Median Median Median Median 1.3963 0.87133 1.7734 0.5492 NaN 1.6566 0.95804 0.66845 1.3457 0.41473 NaN 1.26 NaN 8.2193 NaN NaN NaN NaN 1 2 1 1 0 1 0 2 0 1 0 0 Median Median Median Median Median Median 3.2 3 1.8 1.8 0 1.8 22151000 9846000 5024300 7281000 4558900 2134400 937900 1486600 3421900 1339200 942960 1139700 3688400 1834400 583660 1270400 4520300 1726200 1398000 1396100 0 0 0 0 5961800 2811800 1161800 1988200 1 2960;6783;12344 True;True;True 3134;7139;13093 28030;28031;60955;114174;114175;114176;114177 39563;39564;89238;165912;165913;165914;165915;165916 39563;89238;165912

A0A096MIV6 A0A096MIV6 2 2 2 Wbscr17 tr|A0A096MIV6|A0A096MIV6_RAT Uncharacterized protein (Fragment) OS=Rattus norvegicus OX=10116 GN=Galnt17 PE=4 SV=1 1 2 2 2 1 1 0 0 1 0 1 1 0 0 1 0 1 1 0 0 1 0 5.9 5.9 5.9 47.717 422 422 4.33 2 1 0.0038168 2.0509 By MS/MS By matching By MS/MS 1.2775 3.4737 81.328 3 1 Plateau 1.053 2.5308 57.103 3 1 Median 1.2197 0.90691 15.541 3 1 Median 1.0473 1.2775 NaN NaN 0.26455 NaN 3.0969 3.4737 NaN NaN 0.80468 NaN NaN NaN NaN NaN NaN NaN 1 1 0 0 1 0 0 0 0 0 1 0 Median Median Median Median Median Median 1.3437 1.053 NaN NaN 0.38764 NaN 2.802 2.5308 NaN NaN 0.99433 NaN NaN NaN NaN NaN NaN NaN 1 1 0 0 1 0 0 0 0 0 1 0 Median Median Median Median Median Median 1.2197 0.94078 NaN NaN 1.4653 NaN 0.90691 0.75265 NaN NaN 1.0247 NaN NaN NaN NaN NaN NaN NaN 1 1 0 0 1 0 0 0 0 0 1 0 Median Median Median Median Median Median 2.4 2.4 0 0 3.6 0 7009900 2405500 1987400 2617000 1862300 621530 590560 650250 2141800 477270 811470 853080 0 0 0 0 0 0 0 0 3005800 1306700 585350 1113700 0 0 0 0 2 2869;12114 True;True 3040;12851 27145;112048;112049 38118;163001 38118;163001

E9PT29;A0A096MIX2;A0A096MJW9 E9PT29;A0A096MIX2;A0A096MJW9 6;6;4 4;4;3 4;4;3 Ddx17 tr|E9PT29|E9PT29_RAT DEAD-box helicase 17 OS=Rattus norvegicus OX=10116 GN=Ddx17 PE=1 SV=3;tr|A0A096MIX2|A0A096MIX2_RAT DEAD (Asp-Glu-Ala-Asp) box polypeptide 17, isoform CRA_a OS=Rattus norvegicus OX=10116 GN=Ddx17 PE=1 SV=1;tr|A0A096MJW9|A0A096MJW9_RAT D 3 6 4 4 5 6 5 5 4 4 3 4 3 3 3 2 3 4 3 3 3 2 11.5 6.9 6.9 72.459 650 650;652;231 4.27 1 3 7 11 0 35.429 By MS/MS By MS/MS By MS/MS By MS/MS By MS/MS By MS/MS 0.53968 1.7496 48.269 22 9 Leave out requantified 0.70793 1.7337 40.692 22 9 Leave out requantified 1.3084 0.95667 2.636 22 9 Leave out requantified 0.59159 0.44649 0.83085 0.50114 0.74467 0.60182 2.2055 1.3909 2.4784 1.7868 5.3894 2.3138 41.37 27.934 50.455 9.0391 91.304 39.532 5 5 3 4 3 2 1 1 1 4 1 1 Leave out requantified Leave out requantified Median Median Plateau Median 0.69384 0.56792 1.2961 0.66347 1.6584 0.84692 1.6694 1.4465 3.6904 1.6847 4.9847 2.3776 22.16 18.734 64.974 44.967 87.727 12.644 5 5 3 4 3 2 1 1 1 4 1 1 Leave out requantified Leave out requantified Plateau Median Plateau Median 1.2997 1.2066 1.4366 1.3239 1.3595 1.2791 0.91428 0.95478 1.09 0.99982 0.92668 0.96416 8.4588 1.8051 39.12 58.135 24.217 1.1039 5 5 3 4 3 2 1 1 1 4 1 1 Leave out requantified Leave out requantified Plateau Median Median Median 9.7 11.5 9.7 9.7 8.2 7.8 153790000 56776000 41838000 55172000 25507000 9564600 6947400 8994800 37151000 15620000 9646100 11885000 18629000 6375600 5080900 7172100 17925000 7384600 4883400 5657400 22681000 6254400 6885500 9541400 31893000 11577000 8394700 11922000 3 975;4617;6277;9884;10370;11346 False;True;True;False;True;True 1035;4866;6617;10480;10990;12036 8556;8557;8558;8559;8560;8561;8562;8563;8564;8565;8566;8567;8568;8569;8570;8571;8572;8573;8574;8575;8576;8577;8578;8579;8580;8581;8582;41768;41769;41770;41771;41772;41773;41774;41775;41776;41777;56775;56776;88935;88936;88937;88938;88939;88940;88941;93556;93557;93558;93559;93560;93561;103979;103980;103981;103982 12278;12279;12280;12281;12282;12283;12284;12285;12286;12287;12288;12289;12290;12291;12292;12293;12294;12295;12296;12297;12298;12299;12300;12301;12302;12303;12304;12305;12306;12307;12308;12309;12310;12311;12312;12313;12314;12315;12316;12317;12318;12319;12320;12321;12322;12323;12324;59771;59772;59773;59774;59775;59776;59777;59778;59779;59780;83297;129338;129339;129340;129341;129342;129343;129344;129345;129346;129347;129348;135800;135801;135802;135803;135804;135805;135806;135807;135808;151298;151299;151300;151301 12310;59779;83297;129343;135802;151299

A0A096MIY2;Q5D023;A0A096MKA1;Q62698;A0A096MIX4 A0A096MIY2;Q5D023;A0A096MKA1;Q62698 5;5;4;4;2 5;5;4;4;2 5;5;4;4;2 Cytoplasmic dynein 1 light intermediate chain 2 Dync1li2 tr|A0A096MIY2|A0A096MIY2_RAT Cytoplasmic dynein 1 light intermediate chain 2 OS=Rattus norvegicus OX=10116 GN=Dync1li2 PE=1 SV=1;tr|Q5D023|Q5D023_RAT Cytoplasmic dynein 1 light intermediate chain 2 OS=Rattus norvegicus OX=10116 GN=Dync1li2 PE=1 SV=1;tr|A0A 5 5 5 5 5 4 3 2 2 2 5 4 3 2 2 2 5 4 3 2 2 2 13.4 13.4 13.4 51.545 470 470;492;268;497;56 4.36 6 11 8 0 10.189 By MS/MS By MS/MS By MS/MS By MS/MS By MS/MS By MS/MS 0.34694 1.4559 60.472 24 17 Leave out requantified 0.45454 0.81645 39.605 24 17 Leave out requantified 1.4868 1.1791 35.672 24 17 Leave out requantified 0.32825 0.27052 0.38278 0.35298 0.63678 0.32347 1.254 0.98067 1.3281 1.385 1.8143 1.3199 68.835 51.488 70.658 79.022 36.22 17.17 8 4 3 3 3 3 7 1 3 3 1 2 Median Leave out requantified Median Median Plateau Median 0.69552 0.29801 0.47722 0.39326 0.44228 0.66443 1.6353 0.52838 1.4339 0.97549 1.8782 1.6183 37.186 55.552 64.372 8.805 30.786 25.014 8 4 3 3 3 3 7 1 3 3 1 2 Median Leave out requantified Median Median Plateau Median 2.0626 1.1345 1.1845 2.2925 0.90197 1.4868 1.4737 0.96709 0.97037 1.7492 0.63169 1.1854 38.228 26.316 32.923 59.632 3.4971 49.725 8 4 3 3 3 3 7 1 3 3 1 2 Median Leave out requantified Plateau Plateau Median Median 13.4 9.6 9.1 5.3 5.5 5.3 82316000 43614000 14247000 24455000 24822000 12284000 3527800 9010300 15684000 9400600 2938500 3344600 9324000 5574800 1787400 1961800 9870300 5192600 1338300 3339400 5786600 2686800 1587000 1512800 16829000 8475200 3067800 5286200 4 1797;2938;5908;7536;8345 True;True;True;True;True 1899;3112;6233;7993;8855 16129;16130;16131;16132;16133;16134;27885;27886;27887;27888;27889;27890;27891;27892;53717;53718;67832;67833;67834;67835;67836;67837;67838;75439;75440 22886;22887;22888;22889;22890;22891;39366;39367;39368;39369;39370;39371;39372;39373;78768;78769;98690;98691;98692;98693;98694;98695;98696;98697;109639 22890;39367;78768;98690;109639 0 364

F1LMC6;A0A096MIZ5;P13413 F1LMC6;A0A096MIZ5;P13413 2;2;2 2;2;2 2;2;2 Troponin I, slow skeletal muscle Tnni1 tr|F1LMC6|F1LMC6_RAT Troponin I, slow skeletal muscle OS=Rattus norvegicus OX=10116 GN=Tnni1 PE=1 SV=2;tr|A0A096MIZ5|A0A096MIZ5_RAT Troponin I, slow skeletal muscle OS=Rattus norvegicus OX=10116 GN=Tnni1 PE=1 SV=1;sp|P13413|TNNI1_RAT Troponin I, slow skele 3 2 2 2 1 0 0 1 0 0 1 0 0 1 0 0 1 0 0 1 0 0 11.8 11.8 11.8 21.697 187 187;187;187 9 3 0.0077273 1.6941 By MS/MS By MS/MS 0.43738 1.8853 NaN 1 0 Median 0.68141 1.9433 NaN 1 0 Median 1.7047 1.1774 NaN 1 0 Median NaN NaN NaN 0.43738 NaN NaN NaN NaN NaN 1.8853 NaN NaN NaN NaN NaN NaN NaN NaN 0 0 0 1 0 0 0 0 0 0 0 0 Median Median Median Median Median Median NaN NaN NaN 0.68141 NaN NaN NaN NaN NaN 1.9433 NaN NaN NaN NaN NaN NaN NaN NaN 0 0 0 1 0 0 0 0 0 0 0 0 Median Median Median Median Median Median NaN NaN NaN 1.7047 NaN NaN NaN NaN NaN 1.1774 NaN NaN NaN NaN NaN NaN NaN NaN 0 0 0 1 0 0 0 0 0 0 0 0 Median Median Median Median Median Median 6.4 0 0 5.3 0 0 5223300 3779400 579590 864380 2698300 2698300 0 0 0 0 0 0 0 0 0 0 2525000 1081100 579590 864380 0 0 0 0 0 0 0 0 5 7896;11722 True;True 8383;8384;12440 71756;71757;108220 104447;104448;157658;157659 104448;157658

A0A096MJ24;M0R3X6;A0A0G2JSV2;P47727 A0A096MJ24;M0R3X6;A0A0G2JSV2;P47727 1;1;1;1 1;1;1;1 1;1;1;1 Carbonyl reductase [NADPH] 1 Cbr1 tr|A0A096MJ24|A0A096MJ24_RAT Carbonyl reductase 1 OS=Rattus norvegicus OX=10116 GN=Cbr1 PE=1 SV=1;tr|M0R3X6|M0R3X6_RAT Carbonyl reductase [NADPH] 1-like OS=Rattus norvegicus OX=10116 GN=LOC102556347 PE=1 SV=1;tr|A0A0G2JSV2|A0A0G2JSV2_RAT Carbonyl reductase 4 1 1 1 1 1 1 1 0 1 1 1 1 1 0 1 1 1 1 1 0 1 6.8 6.8 6.8 22.915 207 207;277;277;277 8.42 7 5 0 8.9568 By MS/MS By MS/MS By MS/MS By MS/MS By MS/MS 0.23295 1.1043 22.193 10 8 Median 0.30243 1.0272 20.447 10 8 Median 1.4675 1.0022 15.298 10 8 Median 0.19204 0.21609 0.26228 0.22067 NaN 0.31251 1.0995 1.0309 1.1576 1.0309 NaN 1.3809 NaN 8.396 4.8944 35.268 NaN NaN 1 2 2 4 0 1 0 2 2 4 0 0 Median Median Median Median Median Median 0.28359 0.26249 0.32781 0.35486 NaN 0.4049 0.96804 0.91579 1.1035 1.0572 NaN 1.3927 NaN 15.296 9.1845 26.485 NaN NaN 1 2 2 4 0 1 0 2 2 4 0 0 Median Median Median Median Median Median 1.629 1.2147 1.2498 1.5476 NaN 1.5771 1.0297 0.94631 0.87279 1.0422 NaN 1.1859 NaN 23.132 15.336 14.546 NaN NaN 1 2 2 4 0 1 0 2 2 4 0 0 Median Median Median Median Median Median 6.8 6.8 6.8 6.8 0 6.8 27733000 17380000 3811800 6540800 2733100 1847900 285220 599960 5286800 3613500 750130 923170 3966200 2546400 406740 1013100 11371000 7140500 1420900 2809800 0 0 0 0 4375800 2232200 948820 1194800 6 9777 True 10369 88094;88095;88096;88097;88098;88099;88100;88101;88102;88103;88104;88105 128129;128130;128131;128132;128133;128134;128135;128136;128137;128138;128139;128140;128141;128142 128136

A0A096MJ40;F1M4Q3 A0A096MJ40;F1M4Q3 6;6 6;6 6;6 Hmcn1 tr|A0A096MJ40|A0A096MJ40_RAT Hemicentin 1 OS=Rattus norvegicus OX=10116 GN=Hmcn1 PE=1 SV=2;tr|F1M4Q3|F1M4Q3_RAT Hemicentin 1 OS=Rattus norvegicus OX=10116 GN=Hmcn1 PE=1 SV=3 2 6 6 6 1 2 4 2 2 3 1 2 4 2 2 3 1 2 4 2 2 3 1.9 1.9 1.9 551.99 5103 5103;5635 1.17 15 3 0 10.343 By MS/MS By MS/MS By MS/MS By MS/MS By MS/MS By MS/MS 0.19244 1.049 84.432 12 4 Median 0.32033 0.93606 95.724 12 4 Median 1.7523 1.3861 45.653 12 4 Leave out requantified 0.18037 0.17877 1.7619 0.17361 0.9903 NaN 0.54909 0.66698 3.8775 1.0268 1.8797 NaN NaN 37.293 74.112 70.191 NaN NaN 1 2 4 4 1 0 0 0 3 1 0 0 Median Median Median Median Median Median 0.25215 0.23251 1.8819 0.32033 2.4983 NaN 1.0068 0.70684 3.3384 0.77858 4.157 NaN NaN 29.426 88.217 121.62 NaN NaN 1 2 4 4 1 0 0 0 3 1 0 0 Median Median Median Median Median Median 1.3781 1.2765 1.0725 2.0847 2.2282 NaN 0.92608 0.9602 0.87587 1.2092 1.9143 NaN NaN 2.1965 20.978 26.351 NaN NaN 1 2 4 4 1 0 0 0 3 1 0 0 Median Median Median Median Median Median 0.3 0.5 1.1 0.5 0.5 1.1 401860000 258800000 46754000 96303000 26069000 18463000 3378600 4227300 55131000 38176000 7076300 9878900 42382000 23458000 8134400 10790000 264620000 177830000 27031000 59757000 7104500 875670 1134400 5094400 6556200 0 0 6556200 7 4051;4674;8452;8972;11714;12300 True;True;True;True;True;True 4279;4926;8967;9506;12432;13048 36493;36494;36495;36496;36497;36498;36499;42432;42433;42434;42435;76308;79977;108140;113854;113855;113856;113857 51490;51491;51492;60692;60693;60694;60695;110802;116444;157499;165499;165500;165501;165502;165503 51491;60692;110802;116444;157499;165501 1 2804

A0A096MJ94;E9PTV4 A0A096MJ94;E9PTV4 1;1 1;1 1;1 Bms1 tr|A0A096MJ94|A0A096MJ94_RAT BMS1 ribosome biogenesis factor (Fragment) OS=Rattus norvegicus OX=10116 GN=Bms1 PE=1 SV=1;tr|E9PTV4|E9PTV4_RAT BMS1 ribosome biogenesis factor OS=Rattus norvegicus OX=10116 GN=Bms1 PE=1 SV=3 2 1 1 1 1 1 1 1 1 1 1 1 1 1 1 1 1 1 1 1 1 1 1.5 1.5 1.5 117.84 1046 1046;1064 1.5 6 3 1 0 4.0367 By MS/MS By MS/MS By MS/MS By matching By MS/MS By MS/MS 1.7822 5.0763 23.142 9 1 Linear 2.1796 6.0066 24.767 9 1 Linear 1.5569 1.1772 26.474 9 1 Median 1.2432 1.9613 1.482 1.8451 1.6182 1.6435 3.3924 4.2035 4.4334 4.1592 3.6112 3.2559 NaN 16.682 NaN NaN 53.085 3.7018 1 2 1 1 2 2 0 0 0 0 1 0 Median Median Median Median Median Median 2.1796 1.9018 2.052 2.8304 2.4521 2.3479 4.362 3.6579 4.9469 5.8342 4.6227 3.9092 NaN 36.162 NaN NaN 32.179 25.185 1 2 1 1 2 2 0 0 0 0 1 0 Median Median Median Median Median Median 1.4142 1.0084 1.4666 1.5955 1.6737 1.8294 1.0666 0.81493 1.1658 1.1772 1.3681 1.608 NaN 16.219 NaN NaN 10.214 6.8025 1 2 1 1 2 2 0 0 0 0 1 0 Median Median Median Median Median Median 1.5 1.5 1.5 1.5 1.5 1.5 57606000 11683000 18299000 27624000 4513800 931270 1510700 2071900 11875000 2569400 4425100 4880100 8880700 1834400 3022300 4024000 10824000 2088000 3237300 5498800 15877000 3136300 4383200 8357200 5636200 1123200 1720400 2792500 8 6862 True 7220 61782;61783;61784;61785;61786;61787;61788;61789;61790;61791 90356;90357;90358;90359;90360;90361;90362;90363;90364;90365 90359

D4A560;A0A096MJ99 D4A560;A0A096MJ99 1;1 1;1 1;1 Vamp4 tr|D4A560|D4A560_RAT Vesicle-associated membrane protein 4 OS=Rattus norvegicus OX=10116 GN=Vamp4 PE=1 SV=1;tr|A0A096MJ99|A0A096MJ99_RAT Vesicle-associated membrane protein 4 OS=Rattus norvegicus OX=10116 GN=Vamp4 PE=1 SV=1 2 1 1 1 0 0 0 0 0 1 0 0 0 0 0 1 0 0 0 0 0 1 13.5 13.5 13.5 16.358 141 141;141 11 1 0.0064605 1.8107 By MS/MS NaN NaN NaN 0 0 Median NaN NaN NaN 0 0 Median NaN NaN NaN 0 0 Median NaN NaN NaN NaN NaN NaN NaN NaN NaN NaN NaN NaN NaN NaN NaN NaN NaN NaN 0 0 0 0 0 0 0 0 0 0 0 0 Median Median Median Median Median Median NaN NaN NaN NaN NaN NaN NaN NaN NaN NaN NaN NaN NaN NaN NaN NaN NaN NaN 0 0 0 0 0 0 0 0 0 0 0 0 Median Median Median Median Median Median NaN NaN NaN NaN NaN NaN NaN NaN NaN NaN NaN NaN NaN NaN NaN NaN NaN NaN 0 0 0 0 0 0 0 0 0 0 0 0 Median Median Median Median Median Median 0 0 0 0 0 13.5 652040 0 0 652040 0 0 0 0 0 0 0 0 0 0 0 0 0 0 0 0 0 0 0 0 652040 0 0 652040 9 4963 True 5222 45386 65368 65368 2 67

A0A096MJB3;M0R565 A0A096MJB3;M0R565 1;1 1;1 1;1 Wdr82 tr|A0A096MJB3|A0A096MJB3_RAT WD repeat domain 82 (Fragment) OS=Rattus norvegicus OX=10116 GN=Wdr82 PE=1 SV=1;tr|M0R565|M0R565_RAT WD repeat domain 82 OS=Rattus norvegicus OX=10116 GN=Wdr82 PE=1 SV=2 2 1 1 1 0 1 0 1 1 1 0 1 0 1 1 1 0 1 0 1 1 1 9.3 9.3 9.3 25.305 227 227;313 7.75 1 3 0 7.5293 By MS/MS By MS/MS By MS/MS By MS/MS 0.59229 2.8292 22.103 4 1 Median 0.75776 2.6149 10.295 4 1 Median 1.364 1.0096 27.799 4 1 Median NaN 0.54915 NaN 0.47079 0.73055 0.63882 NaN 2.6417 NaN 2.2789 3.8334 3.0301 NaN NaN NaN NaN NaN NaN 0 1 0 1 1 1 0 0 0 0 1 0 Median Median Median Median Median Median NaN 0.72382 NaN 0.79328 0.92989 0.71139 NaN 2.5339 NaN 2.6985 2.9691 2.326 NaN NaN NaN NaN NaN NaN 0 1 0 1 1 1 0 0 0 0 1 0 Median Median Median Median Median Median NaN 1.4617 NaN 1.7058 1.2729 0.84154 NaN 1.1373 NaN 1.1574 0.89634 0.63787 NaN NaN NaN NaN NaN NaN 0 1 0 1 1 1 0 0 0 0 1 0 Median Median Median Median Median Median 0 9.3 0 9.3 9.3 9.3 15698000 6023600 4033800 5640200 0 0 0 0 3130800 1350100 676470 1104200 0 0 0 0 4122400 1590700 1201100 1330600 5451600 1809500 1261000 2381200 2992800 1273300 895290 824210 10 8898 True 9429 79393;79394;79395;79396 115492;115493;115494;115495;115496;115497;115498;115499;115500 115495 3 29

D4A8U9;A0A096MJB5 D4A8U9;A0A096MJB5 1;1 1;1 1;1 Unc45b tr|D4A8U9|D4A8U9_RAT Unc-45 homolog B (C. elegans) (Predicted) OS=Rattus norvegicus OX=10116 GN=Unc45b PE=4 SV=1;tr|A0A096MJB5|A0A096MJB5_RAT Unc-45 myosin chaperone B OS=Rattus norvegicus OX=10116 GN=Unc45b PE=4 SV=1 2 1 1 1 0 0 0 0 0 1 0 0 0 0 0 1 0 0 0 0 0 1 1.9 1.9 1.9 81.343 735 735;929 4 1 0.0093044 1.511 By MS/MS 0.22517 0.44923 NaN 1 0 Median 0.2745 0.48566 NaN 1 0 Median 1.2633 1.0844 NaN 1 0 Median NaN NaN NaN NaN NaN 0.22517 NaN NaN NaN NaN NaN 0.44923 NaN NaN NaN NaN NaN NaN 0 0 0 0 0 1 0 0 0 0 0 0 Median Median Median Median Median Median NaN NaN NaN NaN NaN 0.2745 NaN NaN NaN NaN NaN 0.48566 NaN NaN NaN NaN NaN NaN 0 0 0 0 0 1 0 0 0 0 0 0 Median Median Median Median Median Median NaN NaN NaN NaN NaN 1.2633 NaN NaN NaN NaN NaN 1.0844 NaN NaN NaN NaN NaN NaN 0 0 0 0 0 1 0 0 0 0 0 0 Median Median Median Median Median Median 0 0 0 0 0 1.9 3519500 2077100 586260 856180 0 0 0 0 0 0 0 0 0 0 0 0 0 0 0 0 0 0 0 0 3519500 2077100 586260 856180 11 764 True 805 6629 9457;9458 9458

A0A096MKB7;A0A0G2KA61;A0A096MJD3;P67874 A0A096MKB7;A0A0G2KA61;A0A096MJD3;P67874 1;1;1;1 1;1;1;1 1;1;1;1 Casein kinase II subunit beta Csnk2b tr|A0A096MKB7|A0A096MKB7_RAT Casein kinase II subunit beta (Fragment) OS=Rattus norvegicus OX=10116 GN=Csnk2b PE=1 SV=1;tr|A0A0G2KA61|A0A0G2KA61_RAT Casein kinase II subunit beta OS=Rattus norvegicus OX=10116 GN=Csnk2b PE=1 SV=1;tr|A0A096MJD3|A0A096MJD3_RA 4 1 1 1 0 1 1 0 1 1 0 1 1 0 1 1 0 1 1 0 1 1 5.7 5.7 5.7 16.066 141 141;151;164;215 8.8 1 4 1 -2 By MS/MS By MS/MS By MS/MS By MS/MS 0.38498 1.6015 17.569 5 2 Median 0.54133 1.7256 16.726 5 2 Median 1.4305 1.0937 12.746 5 2 Median NaN 0.37278 0.38498 NaN 0.45296 0.31068 NaN 1.5936 1.6015 NaN 2.0167 1.3727 NaN 21.772 NaN NaN NaN NaN 0 2 1 0 1 1 0 1 0 0 1 0 Median Median Median Median Median Median NaN 0.48856 0.54133 NaN 0.55958 0.5017 NaN 1.6056 1.8259 NaN 1.6839 1.7256 NaN 31.661 NaN NaN NaN NaN 0 2 1 0 1 1 0 1 0 0 1 0 Median Median Median Median Median Median NaN 1.2927 1.4864 NaN 1.2354 1.4703 NaN 1.0213 1.0937 NaN 0.84463 1.1056 NaN 13.518 NaN NaN NaN NaN 0 2 1 0 1 1 0 1 0 0 1 0 Median Median Median Median Median Median 0 5.7 5.7 0 5.7 5.7 9138400 4700100 1703600 2734700 0 0 0 0 2125300 1243100 418960 463210 1843600 1035500 354980 453130 0 0 0 0 2614000 1078400 483740 1051900 2555500 1343100 445890 766510 + 12 1653 True 1745 14513;14514;14515;14516;14517 20629;20630;20631;20632;20633 20630 4 44

A0A096MJI4 A0A096MJI4 1 1 1 Gigyf2 tr|A0A096MJI4|A0A096MJI4_RAT Uncharacterized protein (Fragment) OS=Rattus norvegicus OX=10116 GN=Gigyf2 PE=1 SV=1 1 1 1 1 1 1 1 0 1 1 1 1 1 0 1 1 1 1 1 0 1 1 2.8 2.8 2.8 64.047 538 538 2.6 2 3 0.0015053 2.3769 By MS/MS By MS/MS By MS/MS By matching By matching 0.87008 2.205 19.427 5 1 Median 0.97855 1.8175 25.928 5 1 Median 1.1443 0.89106 20.437 5 1 Median 0.87008 1.1673 0.96161 NaN 0.85565 0.81679 1.9483 2.4479 2.416 NaN 2.205 1.53 NaN NaN NaN NaN NaN NaN 1 1 1 0 1 1 0 1 0 0 0 0 Median Median Median Median Median Median 0.8502 1.3357 0.73203 NaN 0.97855 1.0817 1.401 2.3635 1.3278 NaN 1.8175 2.2003 NaN NaN NaN NaN NaN NaN 1 1 1 0 1 1 0 1 0 0 0 0 Median Median Median Median Median Median 1.2973 1.1443 0.75671 NaN 1.0893 1.1466 1.0084 0.95133 0.59874 NaN 0.83032 0.89106 NaN NaN NaN NaN NaN NaN 1 1 1 0 1 1 0 1 0 0 0 0 Median Median Median Median Median Median 2.8 2.8 2.8 0 2.8 2.8 52374000 18305000 17337000 16732000 7578900 2563300 2121400 2894300 6835000 1955600 1985400 2894000 9042200 3398600 3560900 2082700 0 0 0 0 17467000 6293900 6211200 4962400 11450000 4093300 3458400 3898800 13 9417 True 9984 84838;84839;84840;84841;84842 123545;123546;123547 123547 5 263

A0A096MJJ6;M0R9S4;A0A0G2K3Z5;A0FKI7 A0A096MJJ6;M0R9S4;A0A0G2K3Z5;A0FKI7 1;1;1;1 1;1;1;1 1;1;1;1 Acyl-CoA-binding domain-containing protein 5 Acbd5;LOC100910957 tr|A0A096MJJ6|A0A096MJJ6_RAT Acyl-CoA-binding domain-containing protein 5 OS=Rattus norvegicus OX=10116 GN=Acbd5 PE=1 SV=1;tr|M0R9S4|M0R9S4_RAT Acyl-CoA-binding domain-containing protein 5 OS=Rattus norvegicus OX=10116 PE=4 SV=1;tr|A0A0G2K3Z5|A0A0G2K3Z5_RA 4 1 1 1 0 1 0 0 0 0 0 1 0 0 0 0 0 1 0 0 0 0 3.8 3.8 3.8 52.389 470 470;507;508;506 5 1 0.0038204 2.0598 By MS/MS NaN NaN NaN 0 0 Median NaN NaN NaN 0 0 Median NaN NaN NaN 0 0 Median NaN NaN NaN NaN NaN NaN NaN NaN NaN NaN NaN NaN NaN NaN NaN NaN NaN NaN 0 0 0 0 0 0 0 0 0 0 0 0 Median Median Median Median Median Median NaN NaN NaN NaN NaN NaN NaN NaN NaN NaN NaN NaN NaN NaN NaN NaN NaN NaN 0 0 0 0 0 0 0 0 0 0 0 0 Median Median Median Median Median Median NaN NaN NaN NaN NaN NaN NaN NaN NaN NaN NaN NaN NaN NaN NaN NaN NaN NaN 0 0 0 0 0 0 0 0 0 0 0 0 Median Median Median Median Median Median 0 3.8 0 0 0 0 885930 885930 0 0 0 0 0 0 885930 885930 0 0 0 0 0 0 0 0 0 0 0 0 0 0 0 0 0 0 14 9776 True 10368 88093 128128 128128

A0A096MK75;A0A096MJM1;Q32PX6 A0A096MK75;A0A096MJM1;Q32PX6 2;2;2 1;1;1 1;1;1 Rhog tr|A0A096MK75|A0A096MK75_RAT Ras homolog family member G (Fragment) OS=Rattus norvegicus OX=10116 GN=Rhog PE=1 SV=1;tr|A0A096MJM1|A0A096MJM1_RAT Ras homolog family member G (Fragment) OS=Rattus norvegicus OX=10116 GN=Rhog PE=1 SV=6;tr|Q32PX6|Q32PX6_RAT Ras 3 2 1 1 2 2 2 2 2 2 1 1 1 1 1 1 1 1 1 1 1 1 15.7 9.6 9.6 19.872 178 178;185;191 10 8 0.00051467 2.6114 By MS/MS By MS/MS By MS/MS By MS/MS By MS/MS By MS/MS 0.30082 1.3226 33.859 6 4 Median 0.40832 1.3259 13.483 6 4 Median 1.3468 0.9371 49.512 6 4 Median NaN 0.52057 0.26192 0.2601 0.39339 NaN NaN 2.2486 1.1051 1.181 1.7714 NaN NaN NaN 34.784 6.6261 NaN NaN 0 1 2 2 1 0 0 1 2 0 1 0 Median Median Median Median Median Median NaN 0.30216 0.40404 0.45682 0.49259 NaN NaN 1.0483 1.3259 1.3624 1.4282 NaN NaN NaN 3.4746 17.139 NaN NaN 0 1 2 2 1 0 0 1 2 0 1 0 Median Median Median Median Median Median NaN 0.58044 1.5426 1.9843 1.2522 NaN NaN 0.43484 1.0771 1.3837 0.86907 NaN NaN NaN 31.31 44.456 NaN NaN 0 1 2 2 1 0 0 1 2 0 1 0 Median Median Median Median Median Median 15.7 15.7 15.7 15.7 15.7 15.7 9208400 5311000 1255800 2641600 0 0 0 0 1072100 737340 174970 159760 2308700 1408600 362940 537190 4269300 2380200 618000 1271100 1100500 784880 99939 215670 457900 0 0 457900 15 1708;3208 False;True 1804;3391 14888;14889;14890;14891;14892;14893;14894;14895;14896;14897;14898;14899;14900;14901;29731;29732;29733;29734;29735;29736;29737;29738 21108;21109;21110;21111;21112;21113;21114;21115;21116;21117;21118;21119;21120;21121;21122;21123;21124;21125;21126;21127;21128;21129;41665;41666;41667;41668;41669;41670;41671;41672;41673 21109;41665

A0A096MJR6 A0A096MJR6 1 1 1 Parp4 tr|A0A096MJR6|A0A096MJR6_RAT Poly [ADP-ribose] polymerase (Fragment) OS=Rattus norvegicus OX=10116 PE=1 SV=1 1 1 1 1 1 1 1 0 1 0 1 1 1 0 1 0 1 1 1 0 1 0 2 2 2 89.891 804 804 1.2 4 1 0.00055556 3.1167 By MS/MS By MS/MS By MS/MS By MS/MS 1.1576 2.1565 29.585 3 3 Median 0.74506 1.2854 93.045 2 2 Median 0.56765 0.49435 102.41 2 2 Median NaN 1.0857 1.4882 NaN NaN NaN NaN 1.905 3.0334 NaN NaN NaN NaN 17.539 NaN NaN NaN NaN 0 2 1 0 0 0 0 2 1 0 0 0 Median Median Median Median Median Median NaN 1.3772 0.40307 NaN NaN NaN NaN 2.4818 0.66573 NaN NaN NaN NaN NaN NaN NaN NaN NaN 0 1 1 0 0 0 0 1 1 0 0 0 Median Median Median Median Median Median NaN 1.1897 0.27085 NaN NaN NaN NaN 1.0198 0.23963 NaN NaN NaN NaN NaN NaN NaN NaN NaN 0 1 1 0 0 0 0 1 1 0 0 0 Median Median Median Median Median Median 2 2 2 0 2 0 6590900 1726800 3145300 1718800 0 0 0 0 4423500 1193400 1857100 1373000 2167400 533410 1288300 345750 0 0 0 0 0 0 0 0 0 0 0 0 16 7942 True 8432 72182;72183;72184;72185;72186 105023;105024;105025;105026;105027 105027 6 95

A0A096MJW2;A0A0H2UHL1;Q66H50;A0A096MJJ7 A0A096MJW2;A0A0H2UHL1;Q66H50;A0A096MJJ7 7;7;7;5 7;7;7;5 7;7;7;5 Fatty acyl-CoA reductase 1 Far1 tr|A0A096MJW2|A0A096MJW2_RAT Fatty acyl-CoA reductase OS=Rattus norvegicus OX=10116 GN=Far1 PE=3 SV=1;tr|A0A0H2UHL1|A0A0H2UHL1_RAT Fatty acyl-CoA reductase OS=Rattus norvegicus OX=10116 GN=Far1 PE=3 SV=1;sp|Q66H50|FACR1_RAT Fatty acyl-CoA reductase 1 OS=Ra 4 7 7 7 2 2 2 4 6 3 2 2 2 4 6 3 2 2 2 4 6 3 18.6 18.6 18.6 59.375 515 515;574;515;213 1.87 19 4 0 19.214 By MS/MS By MS/MS By MS/MS By MS/MS By MS/MS By MS/MS 1.0964 2.6577 32.66 21 5 Leave out requantified 1.8683 4.1176 14.429 21 5 Leave out requantified 1.7386 1.3445 22.488 21 5 Leave out requantified 1.2305 1.3463 1.3404 0.97923 1.0285 0.7366 3.2583 3.4405 4.3507 2.602 2.6777 4.1704 5.5614 14.739 7.8717 19.573 36.357 64.408 2 2 3 5 5 4 0 0 0 2 2 1 Median Median Median Leave out requantified Leave out requantified Linear 1.4137 1.5972 1.6696 2.0089 2.3839 3.66 2.9516 3.1731 3.4239 4.5102 5.1636 10.049 23.195 23.805 16.123 15.657 12.664 82.912 2 2 3 5 5 4 0 0 0 2 2 1 Median Median Plateau Leave out requantified Leave out requantified Linear 1.4233 1.1609 1.2138 2.101 2.121 2.2989 1.015 0.92388 1.0517 1.4036 1.7394 1.798 3.9971 5.0273 9.6465 21.568 14.787 19.384 2 2 3 5 5 4 0 0 0 2 2 1 Median Median Plateau Leave out requantified Leave out requantified Median 4.9 4.9 4.9 8.9 15.7 7.4 186070000 45019000 49319000 91735000 12990000 3332700 4129500 5528400 16085000 4261800 5847500 5976000 21952000 6161300 7167500 8623600 56028000 13947000 13339000 28741000 47851000 9272900 13095000 25483000 31167000 8043300 5741200 17383000 17 508;703;5107;6233;11500;11713;12324 True;True;True;True;True;True;True 534;740;5372;6573;12205;12430;12431;13073 4463;6150;6151;6152;6153;6154;6155;6156;6157;6158;6159;46466;46467;46468;46469;46470;56501;105181;105182;108137;108138;108139;114043 6292;8776;8777;8778;8779;8780;8781;8782;8783;8784;8785;8786;66905;66906;66907;66908;66909;82938;152982;152983;157495;157496;157497;157498;165758 6292;8783;66908;82938;152983;157496;165758 7 184

A0A096MJZ2 A0A096MJZ2 2 2 2 Tbl2 tr|A0A096MJZ2|A0A096MJZ2_RAT RCG21429, isoform CRA_b OS=Rattus norvegicus OX=10116 GN=Tbl2 PE=1 SV=1 1 2 2 2 1 1 0 1 1 2 1 1 0 1 1 2 1 1 0 1 1 2 5.2 5.2 5.2 49.613 442 442 6.5 5 5 0 53.519 By MS/MS By MS/MS By MS/MS By MS/MS By MS/MS 0.20733 1.0318 30.187 7 4 Median 0.30901 0.9868 25.144 7 4 Median 1.3802 0.95961 42.238 7 4 Median 0.16683 0.23078 NaN 0.14039 0.25703 0.27706 0.78548 1.2212 NaN 0.65594 1.2219 1.1656 NaN 23.84 NaN NaN NaN 30.857 1 2 0 1 1 2 0 2 0 0 1 1 Median Median Median Median Median Median 0.31152 0.29736 NaN 0.33279 0.35677 0.23235 1.0436 1.0666 NaN 0.9868 1.1071 0.65323 NaN 13.108 NaN NaN NaN 16.975 1 2 0 1 1 2 0 2 0 0 1 1 Median Median Median Median Median Median 1.911 1.2885 NaN 2.3916 1.3881 0.82286 1.3717 0.93949 NaN 1.6669 0.95961 0.60577 NaN 15.272 NaN NaN NaN 43.681 1 2 0 1 1 2 0 2 0 0 1 1 Median Median Median Median Median Median 2.9 2.9 0 2.9 2.9 5.2 29665000 20353000 4013800 5298200 2496100 1955900 189270 350890 8539900 5479400 1181300 1879200 0 0 0 0 4361800 2964400 452500 944820 3035500 2231000 287440 517100 11232000 7722500 1903200 1606100 18 974;6865 True;True 1034;7223 8547;8548;8549;8550;8551;8552;8553;8554;8555;61800 12264;12265;12266;12267;12268;12269;12270;12271;12272;12273;12274;12275;12276;12277;90373 12270;90373

Q5FVT1;A0A0G2K694;A0A096MJZ5;F1LR97;Q62796 Q5FVT1;A0A0G2K694;A0A096MJZ5;F1LR97;Q62796 1;1;1;1;1 1;1;1;1;1 1;1;1;1;1 RalA-binding protein 1 Ralbp1;LOC304239 tr|Q5FVT1|Q5FVT1_RAT RCG55460, isoform CRA_a OS=Rattus norvegicus OX=10116 GN=Ralbp1 PE=1 SV=1;tr|A0A0G2K694|A0A0G2K694_RAT RalA-binding protein 1 OS=Rattus norvegicus OX=10116 GN=Ralbp1 PE=1 SV=1;tr|A0A096MJZ5|A0A096MJZ5_RAT Uncharacterized protein (Fragm 5 1 1 1 0 1 1 0 0 1 0 1 1 0 0 1 0 1 1 0 0 1 3.7 3.7 3.7 75.035 647 647;693;714;776;647 4 3 0.0005291 2.7933 By MS/MS By MS/MS By MS/MS 0.57524 1.8448 6.6452 3 1 Plateau 0.54993 1.4697 23.136 3 1 Median 1.1082 0.88535 21.388 3 1 Median NaN 0.47009 0.57524 NaN NaN 0.84503 NaN 1.6404 1.8448 NaN NaN 1.8358 NaN NaN NaN NaN NaN NaN 0 1 1 0 0 1 0 1 0 0 0 0 Median Median Median Median Median Median NaN 0.54993 0.45165 NaN NaN 0.89614 NaN 1.4697 1.0816 NaN NaN 1.702 NaN NaN NaN NaN NaN NaN 0 1 1 0 0 1 0 1 0 0 0 0 Median Median Median Median Median Median NaN 1.1698 0.79756 NaN NaN 1.1082 NaN 0.89808 0.61577 NaN NaN 0.88535 NaN NaN NaN NaN NaN NaN 0 1 1 0 0 1 0 1 0 0 0 0 Median Median Median Median Median Median 0 3.7 3.7 0 0 3.7 18434000 8410400 4900500 5123500 0 0 0 0 6251800 3213100 1316900 1721700 6765200 3064800 1568100 2132300 0 0 0 0 0 0 0 0 5417500 2132500 2015600 1269500 19 195 True 203 1757;1758;1759 2382;2383;2384;2385 2384

A0A096MK15 A0A096MK15 27 27 12 Neb tr|A0A096MK15|A0A096MK15_RAT Nebulin OS=Rattus norvegicus OX=10116 GN=Neb PE=1 SV=1 1 27 27 12 20 1 6 1 4 3 20 1 6 1 4 3 10 0 0 1 0 1 5.3 5.3 2.3 772.09 6666 6666 5.61 15 1 1 21 0 201.08 By MS/MS By MS/MS By MS/MS By MS/MS By MS/MS By MS/MS 3.4797 7.5164 33.478 24 18 Leave out requantified 1.9182 4.1 12.905 18 12 Leave out requantified 0.60175 0.51945 24.237 18 12 Leave out requantified 0.04733 8.4208 3.0197 NaN 3.4886 4.0099 0.18919 21.235 7.1269 NaN 9.1918 8.5956 151.43 NaN 5.9818 NaN 168.66 22.395 10 1 5 0 5 3 9 1 2 0 4 2 Median Median Leave out requantified Median Median Plateau 0.028037 6.4732 2.3626 NaN 1.7746 1.4184 0.080652 12.405 4.2571 NaN 3.8933 2.4673 207.31 NaN 34.863 NaN 42.393 28.956 5 1 5 0 4 3 4 1 2 0 3 2 Median Median Leave out requantified Median Median Median 1.2996 0.76871 0.76175 NaN 0.48115 0.41544 0.96926 0.61452 0.60251 NaN 0.40146 0.34824 159.61 NaN 14.954 NaN 19.487 28.721 5 1 5 0 4 3 4 1 2 0 3 2 Median Median Leave out requantified Median Median Median 4.1 0.4 1.4 0.1 1 0.8 198260000 127790000 42890000 27578000 113810000 109330000 2484500 2000200 7669800 767120 3690600 3212100 32338000 5235900 14919000 12183000 728010 728010 0 0 36947000 10498000 17750000 8698400 6764300 1233600 4045600 1485000 20 462;1770;2652;2828;3125;3904;3906;3909;4104;4226;4227;4228;5104;5467;5958;5974;7089;7333;7405;8249;8250;8384;8517;9542;9669;11594;12170 True;True;True;True;True;True;True;True;True;True;True;True;True;True;True;True;True;True;True;True;True;True;True;True;True;True;True 483;1871;2815;2999;3305;4125;4127;4130;4334;4458;4459;4460;5369;5767;6286;6302;7482;7770;7858;8755;8756;8896;9036;10122;10257;12301;12908 4075;15918;25051;26714;29059;35243;35244;35250;35251;35252;35258;35259;35260;35261;35262;35263;36893;38222;38223;38224;38225;46424;50024;54207;54286;54287;63742;66003;66785;74722;74723;74724;75707;76748;86072;87315;106420;112719 5757;22610;35071;37541;40828;40829;49532;49533;49539;49540;49541;49542;49548;49549;49550;49551;49552;49553;52002;54100;54101;54102;66845;72033;79783;79916;79917;92909;92910;96076;97202;108639;108640;108641;110027;111473;125221;127059;154737;163963 5757;22610;35071;37541;40828;49533;49542;49550;52002;54100;54101;54102;66845;72033;79783;79917;92909;96076;97202;108639;108641;110027;111473;125221;127059;154737;163963 8 603

A0A1W2Q6E9;A0A096MK30;O35763 A0A1W2Q6E9;A0A096MK30;O35763 21;21;18 21;21;18 16;16;13 Moesin Msn tr|A0A1W2Q6E9|A0A1W2Q6E9_RAT Moesin OS=Rattus norvegicus OX=10116 GN=Msn PE=1 SV=1;tr|A0A096MK30|A0A096MK30_RAT Moesin OS=Rattus norvegicus OX=10116 GN=Msn PE=1 SV=1;sp|O35763|MOES_RAT Moesin OS=Rattus norvegicus OX=10116 GN=Msn PE=1 SV=3 3 21 21 16 15 17 17 12 14 16 15 17 17 12 14 16 12 14 13 10 11 12 41.3 41.3 33.7 67.651 576 576;577;577 3.73 49 12 4 93 91 5 1 0 323.31 By MS/MS By MS/MS By MS/MS By MS/MS By MS/MS By MS/MS 0.16146 0.64597 15.984 208 111 Leave out requantified 0.29065 0.87368 11.171 204 107 Leave out requantified 1.8016 1.3075 14.586 204 107 Leave out requantified 0.13884 0.16862 0.16514 0.16722 0.16572 0.16635 0.58254 0.552 0.67505 0.71589 0.75847 0.64025 20.595 16.261 17.518 35.11 16.052 22.919 38 32 33 37 37 31 22 20 15 24 16 14 Leave out requantified Leave out requantified Leave out requantified Leave out requantified Leave out requantified Leave out requantified 0.33805 0.28219 0.29537 0.26707 0.29053 0.28249 0.97269 0.83837 0.89446 0.88696 0.88212 0.82182 17.398 9.5147 15.86 19.213 14.967 12.973 38 31 32 35 37 31 22 19 14 22 16 14 Leave out requantified Leave out requantified Leave out requantified Leave out requantified Leave out requantified Leave out requantified 2.4302 1.7131 1.7939 1.7311 1.6594 1.8026 1.6964 1.3526 1.3639 1.1266 1.1527 1.349 17.513 12.698 17.265 31.378 18.662 18.242 38 31 32 35 37 31 22 19 14 22 16 14 Leave out requantified Leave out requantified Leave out requantified Leave out requantified Leave out requantified Leave out requantified 23.8 33.9 37.2 19.1 22.4 26 3054100000 2070000000 353990000 630110000 324600000 217170000 33694000 73745000 367280000 244480000 45569000 77231000 562820000 377120000 71373000 114330000 639460000 436500000 70013000 132940000 475670000 324310000 56571000 94788000 684240000 470400000 76766000 137080000 21 855;856;1091;2313;2613;2779;3287;3312;3929;5015;5072;5623;5718;5964;8208;8281;8357;8513;9234;10505;10671 True;True;True;True;True;True;True;True;True;True;True;True;True;True;True;True;True;True;True;True;True 901;902;1156;1157;2467;2775;2949;3476;3502;4151;5275;5276;5335;5935;6033;6292;8712;8789;8790;8867;8868;9032;9794;11132;11315 7406;7407;7408;7409;7410;7411;7412;7413;7414;7415;7416;7417;7418;7419;7420;7421;7422;7423;7424;7425;7426;7427;7428;7429;7430;7431;7432;7433;7434;7435;7436;7437;7438;7439;7440;7441;7442;7443;7444;7445;7446;7447;7448;7449;7450;7451;9562;9563;9564;9565;9566;9567;9568;9569;9570;9571;9572;9573;9574;9575;9576;9577;9578;9579;9580;9581;9582;9583;9584;9585;9586;9587;9588;9589;9590;9591;9592;9593;9594;9595;9596;9597;9598;9599;9600;9601;9602;9603;9604;9605;9606;9607;9608;21844;21845;24703;24704;24705;24706;24707;24708;24709;24710;24711;24712;24713;24714;24715;24716;24717;24718;24719;24720;24721;24722;24723;24724;24725;24726;24727;26345;26346;26347;30223;30224;30225;30226;30227;30228;30229;30230;30231;30232;30398;30399;30400;30401;30402;30403;30404;30405;30406;30407;30408;35446;45712;45713;45714;45715;45716;45717;45718;45719;45720;45721;45722;45723;45724;45725;45726;45727;45728;45729;45730;46243;46244;46245;51372;51373;51374;51375;51376;51377;51378;51379;51380;51381;51939;51940;51941;51942;51943;51944;51945;51946;51947;51948;51949;51950;51951;54223;54224;74463;74464;74465;74466;74467;74468;74469;74470;74935;74936;74937;74938;74939;74940;74941;74942;74943;74944;74945;74946;74947;74948;74949;74950;75486;75487;75488;75489;75490;75491;75492;75493;75494;75495;75496;75497;75498;75499;75500;75501;75502;75503;75504;75505;75506;75507;75508;75509;75510;75511;75512;75513;75514;76738;82654;82655;82656;82657;82658;94667;94668;94669;96358 10660;10661;10662;10663;10664;10665;10666;10667;10668;10669;10670;10671;10672;10673;10674;10675;10676;10677;10678;10679;10680;10681;10682;10683;10684;10685;10686;10687;10688;10689;10690;10691;10692;10693;10694;10695;10696;10697;10698;10699;10700;10701;10702;10703;10704;10705;10706;10707;10708;10709;10710;10711;10712;10713;10714;10715;10716;10717;10718;10719;10720;10721;10722;10723;13768;13769;13770;13771;13772;13773;13774;13775;13776;13777;13778;13779;13780;13781;13782;13783;13784;13785;13786;13787;13788;13789;13790;13791;13792;13793;13794;13795;13796;13797;13798;13799;13800;13801;13802;13803;13804;13805;13806;13807;13808;13809;13810;13811;13812;13813;13814;13815;13816;13817;13818;13819;13820;13821;13822;13823;13824;13825;13826;13827;13828;13829;13830;13831;13832;13833;13834;13835;13836;13837;30645;30646;30647;34548;34549;34550;34551;34552;34553;34554;34555;34556;34557;34558;34559;34560;34561;34562;34563;34564;34565;34566;34567;34568;34569;34570;34571;34572;34573;34574;34575;34576;34577;34578;36889;36890;36891;42420;42421;42422;42423;42424;42425;42426;42427;42428;42429;42430;42431;42432;42433;42434;42435;42436;42437;42688;42689;42690;42691;42692;42693;42694;42695;42696;42697;42698;42699;49795;65869;65870;65871;65872;65873;65874;65875;65876;65877;65878;65879;65880;65881;65882;65883;65884;65885;65886;65887;65888;65889;65890;65891;65892;66616;66617;66618;66619;74236;74237;74238;74239;74240;74241;74242;74243;74244;74245;74246;74247;74248;74249;74250;74251;75184;75185;75186;75187;75188;75189;75190;75191;75192;75193;75194;75195;75196;75197;75198;75199;75200;75201;75202;75203;75204;79804;79805;108324;108325;108326;108327;108328;108329;108330;108982;108983;108984;108985;108986;108987;108988;108989;108990;108991;108992;108993;108994;108995;108996;108997;108998;108999;109000;109001;109002;109003;109697;109698;109699;109700;109701;109702;109703;109704;109705;109706;109707;109708;109709;109710;109711;109712;109713;109714;109715;109716;109717;109718;109719;109720;109721;109722;109723;109724;109725;109726;111461;120115;120116;120117;137549;137550;137551;137552;137553;137554;140116 10678;10702;13773;30647;34551;36890;42435;42696;49795;65877;66619;74249;75185;79804;108324;108999;109713;111461;120116;137552;140116 9;10;11;12;13;14 199;317;420;432;450;576

A0A096MK61;M0R3U4 A0A096MK61;M0R3U4 4;3 4;3 4;3 LOC102555086;LOC100910835 tr|A0A096MK61|A0A096MK61_RAT Cartilage-associated protein-like 1 OS=Rattus norvegicus OX=10116 GN=Crtapl1 PE=1 SV=1;tr|M0R3U4|M0R3U4_RAT Cartilage-associated protein-like 1 OS=Rattus norvegicus OX=10116 GN=Crtapl1 PE=1 SV=1 2 4 4 4 2 2 2 3 0 2 2 2 2 3 0 2 2 2 2 3 0 2 11.2 11.2 11.2 46.49 403 403;209 6 1 13 1 0 6.9831 By MS/MS By MS/MS By MS/MS By MS/MS By MS/MS 0.21244 1.1152 9.5892 14 7 Leave out requantified 0.332 1.146 18.408 14 7 Leave out requantified 1.4949 1.0984 10.338 14 7 Leave out requantified 0.21693 0.33747 0.23925 0.22813 NaN 0.23689 1.1295 1.6687 1.1509 1.0856 NaN 1.0957 14.117 32.343 12.258 20.415 NaN 21.496 3 3 2 4 0 2 1 2 0 2 0 2 Median Median Median Median Median Median 0.33506 0.34982 0.34981 0.28851 NaN 0.32093 1.2132 1.2818 1.2075 0.87383 NaN 1.0271 15.824 11.421 0.58376 11.312 NaN 17.438 3 3 2 4 0 2 1 2 0 2 0 2 Plateau Median Median Median Median Median 1.4302 1.2648 1.6223 1.3012 NaN 1.3548 0.97334 1.0382 1.2403 0.89628 NaN 1.0617 22.076 31.691 1.2516 29.024 NaN 4.7505 3 3 2 4 0 2 1 2 0 2 0 2 Plateau Median Median Median Median Median 6.9 6.9 6.9 6.9 0 6.9 68122000 43233000 10203000 14686000 6486500 4310100 760490 1415900 7030200 4178900 1031200 1820100 15267000 9077600 2661200 3528300 14952000 9535400 2186600 3230000 0 0 0 0 24386000 16131000 3563700 4691300 22 561;7366;8737;9443 True;True;True;True 591;7811;9264;10012 4998;4999;5000;5001;5002;5003;5004;66292;66293;66294;66295;66296;66297;78082;85146 7076;7077;7078;7079;7080;7081;7082;7083;7084;7085;7086;7087;7088;96539;96540;96541;96542;96543;96544;96545;96546;113541;113542;113543;123979 7085;96543;113542;123979

A0A096MK73;P13668 A0A096MK73;P13668 1;1 1;1 1;1 Stathmin Stmn1 tr|A0A096MK73|A0A096MK73_RAT Stathmin (Fragment) OS=Rattus norvegicus OX=10116 GN=Stmn1 PE=1 SV=1;sp|P13668|STMN1_RAT Stathmin OS=Rattus norvegicus OX=10116 GN=Stmn1 PE=1 SV=2 2 1 1 1 0 1 1 1 1 1 0 1 1 1 1 1 0 1 1 1 1 1 9.5 9.5 9.5 14.637 126 126;149 10.7 2 5 0 6.6983 By MS/MS By MS/MS By MS/MS By MS/MS By MS/MS 0.27151 1.1325 19.724 2 2 Median 0.16338 0.50095 8.1773 2 2 Median 0.60175 0.41995 16.601 2 2 Median NaN NaN 0.34691 0.2125 NaN NaN NaN NaN 1.302 0.98503 NaN NaN NaN NaN NaN NaN NaN NaN 0 0 1 1 0 0 0 0 1 1 0 0 Median Median Median Median Median Median NaN NaN 0.17605 0.15162 NaN NaN NaN NaN 0.4728 0.53077 NaN NaN NaN NaN NaN NaN NaN NaN 0 0 1 1 0 0 0 0 1 1 0 0 Median Median Median Median Median Median NaN NaN 0.50749 0.71351 NaN NaN NaN NaN 0.37343 0.47225 NaN NaN NaN NaN NaN NaN NaN NaN 0 0 1 1 0 0 0 0 1 1 0 0 Median Median Median Median Median Median 0 9.5 9.5 9.5 9.5 9.5 5077800 4426200 313490 338150 0 0 0 0 434070 434070 0 0 1488100 1238500 127450 122130 1888500 1486500 186040 216020 863280 863280 0 0 403850 403850 0 0 23 1259 True 1331 11271;11272;11273;11274;11275;11276;11277 16192;16193;16194;16195;16196;16197;16198 16197

A0A096MK92;A0A096MJ06;D4ACI9 A0A096MK92;A0A096MJ06;D4ACI9 2;1;1 2;1;1 2;1;1 Ift88 tr|A0A096MK92|A0A096MK92_RAT Intraflagellar transport 88 OS=Rattus norvegicus OX=10116 GN=Ift88 PE=4 SV=1;tr|A0A096MJ06|A0A096MJ06_RAT Intraflagellar transport 88 (Fragment) OS=Rattus norvegicus OX=10116 GN=Ift88 PE=4 SV=1;tr|D4ACI9|D4ACI9_RAT Intraflagell 3 2 2 2 0 1 2 1 1 2 0 1 2 1 1 2 0 1 2 1 1 2 4.6 4.6 4.6 93.054 825 825;175;815 3.14 3 4 0 20.472 By MS/MS By MS/MS By MS/MS By matching By MS/MS 0.30227 0.83135 11.285 3 0 Median 0.4067 0.85852 13.953 3 0 Median 1.4172 1.0333 29.589 3 0 Median NaN NaN 0.24703 NaN 0.35208 0.30227 NaN NaN 0.83135 NaN 0.91047 0.72749 NaN NaN NaN NaN NaN NaN 0 0 1 0 1 1 0 0 0 0 0 0 Median Median Median Median Median Median NaN NaN 0.4706 NaN 0.3853 0.4067 NaN NaN 1.0716 NaN 0.85852 0.8282 NaN NaN NaN NaN NaN NaN 0 0 1 0 1 1 0 0 0 0 0 0 Median Median Median Median Median Median NaN NaN 2.0636 NaN 1.0613 1.4172 NaN NaN 1.4103 NaN 0.78061 1.0333 NaN NaN NaN NaN NaN NaN 0 0 1 0 1 1 0 0 0 0 0 0 Median Median Median Median Median Median 0 2.3 4.6 2.3 2.3 4.6 27394000 16041000 4597300 6755100 0 0 0 0 0 0 0 0 9116900 4694100 1516400 2906400 0 0 0 0 8164300 4947000 1687400 1529900 10112000 6400200 1393500 2318700 24 3958;8614 True;True 4181;9137 35670;35671;35672;77419;77420;77421;77422 50129;112588;112589;112590;112591 50129;112590

A0A096MKB0 A0A096MKB0 3 3 3 Rab24 tr|A0A096MKB0|A0A096MKB0_RAT RAB24, member RAS oncogene family OS=Rattus norvegicus OX=10116 GN=Rab24 PE=1 SV=1 1 3 3 3 2 2 1 2 2 1 2 2 1 2 2 1 2 2 1 2 2 1 18.7 18.7 18.7 23.144 203 203 4.31 8 2 3 0 4.8056 By MS/MS By MS/MS By MS/MS By MS/MS By MS/MS By MS/MS 0.53585 1.5494 36.913 12 2 Leave out requantified 0.57293 1.3254 39.173 12 2 Leave out requantified 1.052 0.8122 3.6909 12 2 Leave out requantified 0.46821 0.50114 0.64897 0.58508 0.52331 0.64589 1.2977 1.4374 1.9847 1.2933 1.5208 1.915 NaN 62.284 28.498 24.617 63.498 8.842 1 2 3 2 2 2 0 0 0 0 1 1 Median Median Median Median Median Median 0.48765 0.57998 0.65874 0.59512 0.60435 0.73768 1.0903 1.361 2.0205 1.213 1.3081 1.6524 NaN 83.932 29.376 14.371 27.681 21.347 1 2 3 2 2 2 0 0 0 0 1 1 Median Median Plateau Median Median Median 1.1037 1.1376 1.1109 1.0535 1.0674 1.0639 0.81022 0.93312 0.93332 0.78783 0.8237 0.79361 NaN 23.548 15.063 0.84763 41.362 12.56 1 2 3 2 2 2 0 0 0 0 1 1 Median Median Plateau Median Median Median 14.3 9.9 5.4 9.9 14.3 5.4 59065000 29352000 13831000 15881000 12059000 7368700 2302400 2388100 6701500 3449800 1660600 1591000 7686400 3299100 2238500 2148800 18821000 9394200 4063000 5363600 5572000 2774200 1110000 1687800 8224800 3066300 2456900 2701600 25 1081;10307;10968 True;True;True 1146;10924;11633 9497;9498;92784;92785;92786;92787;92788;92789;92790;92791;92792;99588;99589 13674;13675;134709;134710;134711;134712;134713;144600;144601 13675;134709;144600 15 181

A0A096MKC0 A0A096MKC0 1 1 1 Gigyf2 tr|A0A096MKC0|A0A096MKC0_RAT Uncharacterized protein (Fragment) OS=Rattus norvegicus OX=10116 GN=Gigyf2 PE=1 SV=1 1 1 1 1 1 0 0 0 0 0 1 0 0 0 0 0 1 0 0 0 0 0 2.4 2.4 2.4 83.432 740 740 2 1 0.00052383 2.7347 By MS/MS 0.86763 1.5452 NaN 1 1 Median 0.91519 1.3627 NaN 1 1 Median 1.0548 0.86646 NaN 1 1 Median 0.86763 NaN NaN NaN NaN NaN 1.5452 NaN NaN NaN NaN NaN NaN NaN NaN NaN NaN NaN 1 0 0 0 0 0 1 0 0 0 0 0 Median Median Median Median Median Median 0.91519 NaN NaN NaN NaN NaN 1.3627 NaN NaN NaN NaN NaN NaN NaN NaN NaN NaN NaN 1 0 0 0 0 0 1 0 0 0 0 0 Median Median Median Median Median Median 1.0548 NaN NaN NaN NaN NaN 0.86646 NaN NaN NaN NaN NaN NaN NaN NaN NaN NaN NaN 1 0 0 0 0 0 1 0 0 0 0 0 Median Median Median Median Median Median 2.4 0 0 0 0 0 3326400 998040 1057700 1270600 3326400 998040 1057700 1270600 0 0 0 0 0 0 0 0 0 0 0 0 0 0 0 0 0 0 0 0 26 7325 True 7760 65956 96005 96005 16 499

A0A0G2JXR0;A0A096MKD4;A0A0G2JYM0;A0A096MJ01 A0A0G2JXR0;A0A096MKD4;A0A0G2JYM0;A0A096MJ01 5;5;5;3 5;5;5;3 5;5;5;3 Ldb3 tr|A0A0G2JXR0|A0A0G2JXR0_RAT LIM domain-binding 3 OS=Rattus norvegicus OX=10116 GN=Ldb3 PE=1 SV=1;tr|A0A096MKD4|A0A096MKD4_RAT LIM domain-binding 3 OS=Rattus norvegicus OX=10116 GN=Ldb3 PE=1 SV=1;tr|A0A0G2JYM0|A0A0G2JYM0_RAT LIM domain-binding 3 OS=Rattus 4 5 5 5 3 2 1 1 2 1 3 2 1 1 2 1 3 2 1 1 2 1 27.1 27.1 27.1 31.427 288 288;679;729;726 5.86 8 1 5 0 12.236 By MS/MS By MS/MS By MS/MS By MS/MS By MS/MS By MS/MS 0.37258 0.86041 93.951 9 9 Median 0.35453 0.62979 68.222 9 9 Median 1.2892 1.0104 59.886 9 9 Median 0.10152 0.27849 0.39386 0.37258 0.31576 0.47415 0.40652 0.86041 1.0093 0.8108 0.71215 0.9049 224.81 NaN 87.207 NaN 51.694 NaN 2 1 2 1 2 1 2 1 2 1 2 1 Median Median Median Median Median Median 0.14581 0.22981 0.24101 0.48942 0.37076 0.61129 0.40424 0.58057 0.49253 0.8224 0.7606 1.0205 161.96 NaN 34.765 NaN 30.937 NaN 2 1 2 1 2 1 2 1 2 1 2 1 Median Median Median Median Median Median 1.4362 0.8252 0.6119 1.3136 1.1742 1.2892 1.0497 0.6391 0.48261 1.0104 0.91218 1.1343 64.159 NaN 121.12 NaN 27.066 NaN 2 1 2 1 2 1 2 1 2 1 2 1 Median Median Median Median Median Median 16 11.5 7.3 5.6 11.1 7.3 61282000 52010000 4498500 4772900 38300000 36829000 688380 782180 4384300 3102900 681520 599840 6065600 4516500 854650 694450 3418700 2200800 555430 662540 6471900 3777400 1261900 1432500 2641400 1583500 456620 601350 27 1855;2047;2326;8983;11849 True;True;True;True;True 1963;2168;2480;9518;12576 16929;18967;18968;21956;21957;21958;21959;80077;80078;109745;109746;109747;109748;109749 23891;26772;26773;30769;30770;30771;30772;30773;30774;30775;30776;116575;116576;116577;159846;159847;159848;159849;159850 23891;26773;30770;116575;159846

A0A096MKE0;A0A096MJA9;A0A0G2K2B5;A0A096MK70 A0A096MKE0;A0A096MJA9;A0A0G2K2B5 9;8;8;1 9;8;8;1 9;8;8;1 Asph tr|A0A096MKE0|A0A096MKE0_RAT Aspartate-beta-hydroxylase OS=Rattus norvegicus OX=10116 GN=Asph PE=1 SV=1;tr|A0A096MJA9|A0A096MJA9_RAT Aspartate-beta-hydroxylase OS=Rattus norvegicus OX=10116 GN=Asph PE=1 SV=1;tr|A0A0G2K2B5|A0A0G2K2B5_RAT Aspartate-beta-hydr 4 9 9 9 4 5 5 7 8 4 4 5 5 7 8 4 4 5 5 7 8 4 19 19 19 82.661 737 737;657;789;291 3.78 1 8 36 0 22.825 By MS/MS By MS/MS By MS/MS By MS/MS By MS/MS By MS/MS 0.34092 1.1344 12.494 43 11 Leave out requantified 0.43547 1.0079 11.484 43 11 Leave out requantified 1.2002 0.86639 17.792 43 11 Leave out requantified 0.41554 0.27276 0.378 0.34684 0.38924 0.30772 0.93829 0.78856 1.252 1.2284 1.2696 0.99185 12.556 24.759 12.944 11.155 12.412 11.431 8 9 5 8 8 5 4 3 1 2 1 0 Leave out requantified Leave out requantified Leave out requantified Leave out requantified Leave out requantified Leave out requantified 0.56608 0.45146 0.44963 0.41151 0.42847 0.4152 0.99382 1.0002 1.1121 1.0164 1.0645 0.97416 13.251 17.881 9.0581 12.425 12.131 14.431 8 9 5 8 8 5 4 3 1 2 1 0 Leave out requantified Leave out requantified Leave out requantified Leave out requantified Leave out requantified Leave out requantified 1.4697 1.4518 1.1644 1.186 1.0989 1.3203 1.0427 1.1464 0.88935 0.81676 0.82617 0.96175 39.318 19.768 22.733 13.138 19.23 9.9118 8 9 5 8 8 5 4 3 1 2 1 0 Leave out requantified Leave out requantified Leave out requantified Leave out requantified Leave out requantified Leave out requantified 8 10 9.9 13.2 16.7 7.2 391370000 219280000 78611000 93475000 24880000 12784000 5167000 6928400 54617000 30011000 10592000 14014000 38643000 21196000 8168400 9277700 91587000 49740000 20911000 20937000 94425000 55101000 17467000 21857000 87217000 50450000 16306000 20461000 28 1044;1092;1097;4152;5973;6279;7431;10178;10375 True;True;True;True;True;True;True;True;True 1107;1158;1163;4382;6301;6619;7885;7886;10787;10995 9075;9076;9077;9078;9079;9080;9609;9610;9611;9612;9613;9638;9639;9640;37338;54278;54279;54280;54281;54282;54283;54284;54285;56791;56792;56793;56794;56795;56796;56797;66977;66978;66979;66980;66981;66982;66983;66984;66985;66986;91648;93585;93586;93587;93588 13001;13002;13003;13004;13005;13006;13007;13838;13839;13840;13841;13868;13869;13870;13871;52685;52686;79902;79903;79904;79905;79906;79907;79908;79909;79910;79911;79912;79913;79914;79915;83312;83313;83314;83315;83316;83317;83318;83319;83320;83321;97475;97476;97477;97478;97479;97480;97481;97482;97483;97484;97485;97486;133105;135840;135841;135842;135843;135844 13004;13839;13869;52685;79914;83320;97484;133105;135843 17;18;19 446;582;584

Q5U2M4;A0A096MKE9;A0A096MJI5 Q5U2M4;A0A096MKE9 6;6;1 6;6;1 6;6;1 DNA ligase Lig3 tr|Q5U2M4|Q5U2M4_RAT DNA ligase OS=Rattus norvegicus OX=10116 GN=Lig3 PE=1 SV=1;tr|A0A096MKE9|A0A096MKE9_RAT DNA ligase OS=Rattus norvegicus OX=10116 GN=Lig3 PE=1 SV=1 3 6 6 6 3 1 4 2 1 3 3 1 4 2 1 3 3 1 4 2 1 3 8.6 8.6 8.6 105.44 943 943;1002;83 3.71 4 10 0 11.629 By MS/MS By MS/MS By MS/MS By MS/MS By MS/MS By MS/MS 0.55834 1.0852 12.299 12 6 Leave out requantified 0.77508 1.4091 32.589 12 6 Leave out requantified 1.2653 0.95895 14.54 12 6 Leave out requantified 0.4748 0.61511 0.40641 0.52705 0.78325 0.71446 0.80051 1.0852 1.0817 1.1549 1.674 1.7918 24.361 NaN 39.297 29.151 NaN 60.316 2 1 4 2 1 2 1 0 2 1 1 1 Median Median Median Median Median Median 0.85125 0.56322 0.80706 0.82404 0.8295 0.7584 1.1429 0.87429 1.5335 1.4081 1.694 1.4634 29.612 NaN 9.3598 1.1499 NaN 6.3238 2 1 4 2 1 2 1 0 2 1 1 1 Median Median Median Median Median Median 1.7588 1.1034 1.7388 1.5293 1.059 0.74404 1.1899 0.95895 1.3348 1.1774 0.83326 0.56827 1.2032 NaN 37.158 32.962 NaN 26.244 2 1 4 2 1 2 1 0 2 1 1 1 Median Median Median Median Median Median 3.3 1.3 5.3 2.3 1.6 5.3 36981000 15271000 7983300 13727000 4576800 2560000 672270 1344500 1613500 772760 432790 407930 10880000 4377700 2060200 4442600 7303600 2831500 1498600 2973500 2302000 1154300 453920 693740 10305000 3574800 2865600 3864400 29 44;3867;4954;5527;6766;8988 True;True;True;True;True;True 46;4084;5213;5832;7122;9524 314;315;316;34801;45336;45337;45338;50638;50639;60832;60833;60834;60835;80114 439;48814;65293;65294;65295;72902;72903;89078;89079;89080;89081;89082;116626 439;48814;65295;72902;89081;116626

A0A096MKG6;F1M6V1;Q6P747 A0A096MKG6;F1M6V1;Q6P747 2;2;2 2;2;2 2;2;2 Heterochromatin protein 1-binding protein 3 Hp1bp3 tr|A0A096MKG6|A0A096MKG6_RAT Heterochromatin protein 1-binding protein 3 (Fragment) OS=Rattus norvegicus OX=10116 GN=Hp1bp3 PE=1 SV=1;tr|F1M6V1|F1M6V1_RAT Heterochromatin protein 1-binding protein 3 OS=Rattus norvegicus OX=10116 GN=Hp1bp3 PE=1 SV=3;sp|Q6P7 3 2 2 2 2 2 2 2 2 1 2 2 2 2 2 1 2 2 2 2 2 1 8.2 8.2 8.2 32.586 294 294;553;553 4.46 7 6 0 24.968 By MS/MS By MS/MS By MS/MS By MS/MS By MS/MS By MS/MS 0.4353 1.5277 16.425 12 4 Leave out requantified 0.60544 1.5403 21.621 12 4 Leave out requantified 1.758 1.2943 5.3006 12 4 Leave out requantified 0.4532 0.45106 0.27587 0.4443 0.54422 NaN 1.7633 1.5298 0.94281 1.5374 1.67 NaN 26.369 16.622 20.452 33.207 28.817 NaN 2 2 2 3 3 0 1 0 0 1 2 0 Median Median Median Median Plateau Median 0.67992 0.47336 0.51568 0.70065 0.50419 NaN 1.6803 1.2432 1.3004 1.7947 1.2815 NaN 26.556 19.002 2.3252 32.354 42.355 NaN 2 2 2 3 3 0 1 0 0 1 2 0 Median Median Median Median Median Median 1.6475 1.0498 1.758 1.6675 1.4564 NaN 1.1316 0.80272 1.3307 1.212 1.019 NaN 13.694 1.5485 1.3743 23.446 29.265 NaN 2 2 2 3 3 0 1 0 0 1 2 0 Median Median Median Median Median Median 8.2 8.2 8.2 8.2 8.2 4.4 67773000 30644000 14604000 22526000 10560000 4624500 2256200 3679000 10983000 5084900 2788900 3108700 12069000 6363000 2191500 3514500 22424000 9341000 5044900 8037800 11738000 5230600 2322100 4185700 0 0 0 0 30 1307;4015 True;True 1383;4241 11672;11673;11674;11675;11676;11677;11678;36241;36242;36243;36244;36245;36246 16758;16759;16760;16761;16762;16763;16764;16765;16766;16767;16768;16769;51146;51147;51148;51149;51150;51151;51152;51153;51154 16765;51149 20 5

A0A096P6L8;F1LST1;P04937 A0A096P6L8;F1LST1;P04937 16;15;15 16;15;15 16;15;15 Fibronectin;Anastellin Fn1 tr|A0A096P6L8|A0A096P6L8_RAT Fibronectin OS=Rattus norvegicus OX=10116 GN=Fn1 PE=1 SV=2;tr|F1LST1|F1LST1_RAT Fibronectin OS=Rattus norvegicus OX=10116 GN=Fn1 PE=1 SV=3;sp|P04937|FINC_RAT Fibronectin OS=Rattus norvegicus OX=10116 GN=Fn1 PE=1 SV=2 3 16 16 16 10 10 10 13 12 15 10 10 10 13 12 15 10 10 10 13 12 15 9.4 9.4 9.4 272.44 2477 2477;2387;2477 2.47 61 94 52 34 5 5 2 1 1 0 323.31 By MS/MS By MS/MS By MS/MS By MS/MS By MS/MS By MS/MS 0.92942 2.8974 17.367 246 34 Leave out requantified 1.2303 3.1182 17.157 246 34 Leave out requantified 1.3655 1.0039 9.1936 246 34 Leave out requantified 0.86658 0.98857 0.88315 0.96458 0.85109 0.95997 2.4419 3.0121 3.2575 2.492 2.4752 3.2773 20.394 6.3038 37.03 15.967 16.046 27.844 40 31 35 45 45 50 8 7 4 3 7 5 Leave out requantified Leave out requantified Leave out requantified Leave out requantified Leave out requantified Leave out requantified 1.2676 1.0655 1.0838 1.2148 1.1761 1.35 2.6367 2.645 2.7543 3.1425 3.1691 3.3691 11.703 17.034 17.548 23.864 35.183 20.605 40 31 35 45 45 50 8 7 4 3 7 5 Leave out requantified Leave out requantified Leave out requantified Leave out requantified Leave out requantified Leave out requantified 1.3325 1.1824 1.1307 1.4726 1.5369 1.3999 0.95435 0.92014 0.86519 1.0233 1.1532 1.0233 14.747 11.999 17.902 7.8362 7.6863 11.866 40 31 35 45 45 50 8 7 4 3 7 5 Leave out requantified Leave out requantified Leave out requantified Leave out requantified Leave out requantified Leave out requantified 5.3 5.7 6.2 7.9 7.5 9 12818000000 3681100000 3970200000 5166900000 1939700000 606390000 586220000 747060000 1791400000 541110000 563240000 687020000 1579500000 469660000 521750000 588070000 2521800000 710770000 743520000 1067500000 2430700000 711720000 727360000 991630000 2555100000 641460000 828090000 1085600000 31 1592;2134;2656;3903;4291;5902;6721;8471;8769;9107;10191;10929;11160;11989;12141;12225 True;True;True;True;True;True;True;True;True;True;True;True;True;True;True;True 1681;2259;2819;4124;4524;6227;7076;8987;8988;9297;9657;10802;11593;11833;12723;12878;12968;12969 14145;14146;14147;19917;19918;19919;19920;19921;19922;19923;19924;19925;19926;19927;19928;19929;19930;19931;19932;19933;19934;19935;19936;19937;25068;25069;25070;25071;25072;25073;25074;25075;25076;25077;25078;25079;25080;25081;25082;25083;25084;25085;35197;35198;35199;35200;35201;35202;35203;35204;35205;35206;35207;35208;35209;35210;35211;35212;35213;35214;35215;35216;35217;35218;35219;35220;35221;35222;35223;35224;35225;35226;35227;35228;35229;35230;35231;35232;35233;35234;35235;35236;35237;35238;35239;35240;35241;35242;38634;38635;38636;38637;38638;38639;38640;38641;38642;38643;38644;38645;38646;38647;38648;38649;38650;38651;38652;38653;38654;38655;38656;38657;38658;38659;38660;38661;38662;38663;38664;38665;38666;53673;53674;53675;53676;53677;53678;53679;60474;60475;76392;76393;76394;76395;76396;76397;76398;76399;76400;76401;76402;76403;76404;76405;76406;76407;76408;76409;76410;76411;78330;81413;81414;81415;81416;81417;81418;81419;81420;81421;91790;91791;99231;99232;99233;99234;99235;99236;99237;99238;99239;99240;99241;99242;99243;99244;99245;99246;99247;99248;99249;99250;99251;101628;111054;111055;111056;111057;111058;111059;111060;111061;111062;111063;111064;111065;111066;111067;111068;111069;111070;111071;111072;111073;111074;111075;111076;111077;111078;111079;111080;111081;111082;111083;111084;111085;111086;111087;111088;111089;111090;111091;111092;111093;112457;112458;112459;112460;112461;112462;112463;112464;112465;112466;112467;112468;112469;112470;112471;112472;112473;112474;112475;112476;112477;112478;113230;113231;113232;113233;113234;113235;113236;113237;113238 20171;20172;20173;28146;28147;28148;28149;28150;28151;28152;28153;28154;28155;28156;28157;28158;28159;28160;28161;28162;28163;28164;28165;28166;28167;28168;28169;28170;28171;28172;28173;28174;28175;28176;35086;35087;35088;35089;35090;35091;35092;35093;35094;35095;35096;35097;35098;35099;35100;35101;35102;35103;35104;35105;49433;49434;49435;49436;49437;49438;49439;49440;49441;49442;49443;49444;49445;49446;49447;49448;49449;49450;49451;49452;49453;49454;49455;49456;49457;49458;49459;49460;49461;49462;49463;49464;49465;49466;49467;49468;49469;49470;49471;49472;49473;49474;49475;49476;49477;49478;49479;49480;49481;49482;49483;49484;49485;49486;49487;49488;49489;49490;49491;49492;49493;49494;49495;49496;49497;49498;49499;49500;49501;49502;49503;49504;49505;49506;49507;49508;49509;49510;49511;49512;49513;49514;49515;49516;49517;49518;49519;49520;49521;49522;49523;49524;49525;49526;49527;49528;49529;49530;49531;54702;54703;54704;54705;54706;54707;54708;54709;54710;54711;54712;54713;54714;54715;54716;54717;54718;54719;54720;54721;54722;54723;54724;54725;54726;54727;54728;54729;54730;54731;54732;54733;54734;54735;54736;54737;54738;54739;54740;54741;54742;54743;54744;54745;54746;54747;54748;54749;54750;54751;54752;54753;54754;54755;54756;54757;54758;54759;54760;54761;54762;54763;54764;54765;54766;54767;54768;54769;54770;54771;54772;54773;54774;54775;54776;54777;54778;54779;54780;78703;78704;78705;78706;78707;78708;78709;78710;78711;78712;78713;88621;88622;110912;110913;110914;110915;110916;110917;110918;110919;110920;110921;110922;110923;110924;110925;110926;110927;110928;110929;110930;110931;110932;110933;110934;110935;110936;110937;110938;110939;110940;110941;110942;110943;110944;110945;110946;110947;110948;110949;110950;110951;110952;110953;110954;110955;110956;113963;118407;118408;118409;118410;118411;118412;118413;118414;133287;133288;133289;133290;144121;144122;144123;144124;144125;144126;144127;144128;144129;144130;144131;144132;144133;144134;144135;144136;144137;144138;144139;144140;144141;147636;161557;161558;161559;161560;161561;161562;161563;161564;161565;161566;161567;161568;161569;161570;161571;161572;161573;161574;161575;161576;161577;161578;161579;161580;161581;161582;161583;161584;161585;161586;161587;161588;161589;161590;161591;161592;161593;161594;161595;161596;161597;161598;161599;161600;161601;161602;161603;161604;161605;161606;161607;161608;161609;161610;161611;161612;161613;161614;161615;161616;161617;161618;161619;161620;161621;161622;161623;161624;161625;161626;161627;161628;161629;161630;161631;161632;161633;161634;161635;161636;161637;161638;161639;163512;163513;163514;163515;163516;163517;163518;163519;163520;163521;163522;163523;163524;163525;163526;163527;163528;163529;163530;163531;163532;163533;163534;163535;163536;163537;163538;163539;163540;163541;163542;163543;163544;163545;163546;163547;163548;163549;163550;163551;163552;163553;163554;163555;163556;163557;163558;163559;163560;163561;164645;164646;164647;164648;164649;164650;164651;164652;164653;164654;164655;164656 20173;28170;35104;49458;54771;78713;88622;110940;113963;118411;133290;144137;147636;161619;163526;164650 21;22 1046;1066

A0A097BW25;D3ZAF5 A0A097BW25;D3ZAF5 14;13 14;13 10;9 Postn tr|A0A097BW25|A0A097BW25_RAT Periostin OS=Rattus norvegicus OX=10116 GN=Postn PE=1 SV=1;tr|D3ZAF5|D3ZAF5_RAT Periostin OS=Rattus norvegicus OX=10116 GN=Postn PE=1 SV=1 2 14 14 10 4 7 7 8 7 9 4 7 7 8 7 9 3 5 5 5 5 6 24 24 18.3 87.152 783 783;810 4.02 1 4 52 8 0 71.957 By MS/MS By MS/MS By MS/MS By MS/MS By MS/MS By MS/MS 1.8814 6.2723 41.112 55 16 Leave out requantified 1.8558 4.6705 35.516 55 16 Leave out requantified 0.91744 0.69088 8.9086 55 16 Leave out requantified 2.214 2.2161 2.1776 1.9456 1.9279 1.8814 6.988 6.132 8.1839 6.838 6.4656 6.9942 34.81 47.525 41.827 46.75 38.619 32.686 7 8 9 9 9 13 2 0 4 1 3 6 Leave out requantified Leave out requantified Leave out requantified Leave out requantified Leave out requantified Leave out requantified 1.9879 1.9301 2.0467 1.8558 2.1355 1.5737 4.5774 4.6705 5.8328 4.3773 4.8165 4.4659 46.255 43.094 27.589 43.165 36.208 41.929 7 8 9 9 9 13 2 0 4 1 3 6 Leave out requantified Leave out requantified Leave out requantified Leave out requantified Leave out requantified Leave out requantified 0.89503 0.92352 0.88121 0.93836 0.97042 0.94609 0.63442 0.72988 0.6442 0.66781 0.69878 0.72736 16.144 10.136 10.196 19.942 6.5928 9.7813 7 8 9 9 9 13 2 0 4 1 3 6 Leave out requantified Leave out requantified Leave out requantified Leave out requantified Leave out requantified Leave out requantified 6.8 14 12.3 14 13 14 1006800000 202330000 406740000 397740000 90073000 18070000 37115000 34888000 142780000 31513000 54980000 56291000 134140000 29807000 53166000 51171000 201000000 38152000 87420000 75433000 161940000 30528000 63710000 67698000 276870000 54261000 110350000 112250000 32 2121;2764;2765;2885;3401;4100;4361;5113;7386;7504;8427;11023;11186;12313 True;True;True;True;True;True;True;True;True;True;True;True;True;True 2244;2934;2935;3057;3592;4330;4599;5379;7834;7960;8941;11692;11862;13062 19830;26207;26208;26209;26210;26211;26212;26213;26214;26215;27335;31040;31041;31042;36824;36825;36826;36827;39285;39286;46502;46503;46504;46505;46506;46507;46508;46509;66550;67576;67577;76108;76109;76110;76111;76112;76113;100154;102160;102161;102162;102163;102164;102165;102166;113946;113947;113948;113949;113950;113951;113952;113953;113954;113955;113956;113957;113958;113959;113960;113961;113962;113963;113964;113965 28041;36715;36716;36717;36718;36719;36720;36721;36722;36723;38351;43569;43570;43571;43572;43573;51913;51914;51915;51916;51917;51918;51919;51920;51921;55610;55611;66959;66960;66961;66962;66963;66964;66965;66966;66967;66968;66969;66970;66971;96913;96914;96915;98378;98379;110576;110577;110578;145508;148389;148390;148391;148392;148393;148394;148395;148396;148397;165620;165621;165622;165623;165624;165625;165626;165627;165628;165629;165630;165631;165632;165633;165634;165635;165636;165637;165638;165639;165640;165641;165642;165643;165644;165645;165646;165647 28041;36719;36723;38351;43571;51913;55611;66960;96915;98378;110577;145508;148395;165641 23 546

A0A0A0MXU4;B0BNG0 A0A0A0MXU4;B0BNG0 4;4 4;4 4;4 ER membrane protein complex subunit 2 Emc2 tr|A0A0A0MXU4|A0A0A0MXU4_RAT ER membrane protein complex subunit 2 OS=Rattus norvegicus OX=10116 GN=Emc2 PE=4 SV=2;sp|B0BNG0|EMC2_RAT ER membrane protein complex subunit 2 OS=Rattus norvegicus OX=10116 GN=Emc2 PE=2 SV=1 2 4 4 4 1 1 1 2 1 4 1 1 1 2 1 4 1 1 1 2 1 4 16.9 16.9 16.9 34.513 296 296;297 7.9 4 14 2 0 6.1391 By MS/MS By MS/MS By MS/MS By MS/MS By MS/MS By MS/MS 0.35513 1.7086 70.282 4 3 Median 0.25655 0.83427 8.7196 4 3 Median 0.7911 0.5656 73.6 4 3 Median NaN NaN NaN 0.56613 NaN 0.22277 NaN NaN NaN 2.749 NaN 1.0619 NaN NaN NaN NaN NaN 76.383 0 0 0 1 0 3 0 0 0 1 0 2 Median Median Median Median Median Median NaN NaN NaN 0.31296 NaN 0.25221 NaN NaN NaN 0.9736 NaN 0.82774 NaN NaN NaN NaN NaN 2.639 0 0 0 1 0 3 0 0 0 1 0 2 Median Median Median Median Median Median NaN NaN NaN 0.5528 NaN 1.6364 NaN NaN NaN 0.37343 NaN 1.2389 NaN NaN NaN NaN NaN 82.481 0 0 0 1 0 3 0 0 0 1 0 2 Median Median Median Median Median Plateau 4.7 4.7 4.7 9.8 4.7 16.9 9845000 6527400 1736100 1581500 0 0 0 0 0 0 0 0 0 0 0 0 1559400 807030 386820 365570 0 0 0 0 8285600 5720400 1349300 1215900 33 1426;11789;12064;12311 True;True;True;True 1509;12511;12799;13060 12811;12812;12813;12814;12815;12816;12817;12818;12819;12820;12821;12822;12823;12824;12825;12826;108907;108908;111613;113941 18351;18352;18353;18354;18355;18356;18357;18358;18359;18360;18361;18362;18363;18364;18365;18366;158620;158621;162339;165615 18364;158621;162339;165615 24 287

A0A0A0MXW9;A0A0G2K8K9;P84586;D4AE41;Q4V898 A0A0A0MXW9;A0A0G2K8K9;P84586;D4AE41;Q4V898 5;5;5;5;5 5;5;5;5;5 5;5;5;5;5 RNA-binding motif protein, X chromosome retrogene-like;RNA-binding motif protein, X chromosome retrogene-like, N-terminally processed;RNA binding motif protein, X-linked-like-1;RNA-binding motif protein, X chromosome;RNA-binding motif protein, X chromosome, N-terminally processed Rbmxrtl;Rbmxl1;Rbmx tr|A0A0A0MXW9|A0A0A0MXW9_RAT RNA-binding motif protein, X-linked-like-1 OS=Rattus norvegicus OX=10116 GN=Rbmxl1 PE=1 SV=1;tr|A0A0G2K8K9|A0A0G2K8K9_RAT RNA binding motif protein, X-linked-like 1B OS=Rattus norvegicus OX=10116 GN=Rbmxl1b PE=4 SV=1;sp|P84586| 5 5 5 5 2 2 2 3 3 4 2 2 2 3 3 4 2 2 2 3 3 4 12.2 12.2 12.2 40.07 368 368;388;388;388;390 6.28 1 1 11 12 0 61.613 By MS/MS By MS/MS By MS/MS By MS/MS By MS/MS By MS/MS 0.21415 1.1499 49.38 24 10 Leave out requantified 0.28702 0.90728 14.877 24 10 Leave out requantified 1.1901 0.83642 30.77 24 10 Leave out requantified 0.21416 0.21243 0.25546 0.21852 0.2858 0.20691 1.1425 1.1396 1.0508 1.0774 1.4825 0.99964 38.565 6.9468 NaN 13.318 23.25 53.054 5 4 1 5 4 5 2 0 1 3 3 1 Median Leave out requantified Median Median Median Leave out requantified 0.24258 0.24069 0.28077 0.24091 0.28343 0.30213 0.77297 0.88852 0.81479 0.75069 0.91737 0.87493 52.387 6.1143 NaN 30.503 14.259 30.604 5 4 1 5 4 5 2 0 1 3 3 1 Median Leave out requantified Median Median Median Leave out requantified 1.0412 1.1813 1.0991 1.1908 1.0904 1.0793 0.70906 0.84504 0.85333 0.82337 0.77675 0.71214 23.294 4.263 NaN 27.058 27.483 38.133 5 4 1 5 4 5 2 0 1 3 3 1 Median Leave out requantified Median Median Median Leave out requantified 5.7 5.7 5.7 7.9 5.7 10.1 125670000 82970000 18795000 23903000 11697000 8000500 1689500 2007300 27340000 18434000 3827800 5077700 4887400 3433400 766500 687500 23181000 15874000 2875200 4432000 20041000 12390000 3319800 4331700 38522000 24838000 6316400 7366800 34 730;6135;9734;11087;11088 True;True;True;True;True 768;6472;10324;11758;11759 6353;55637;55638;55639;55640;55641;55642;55643;55644;55645;55646;55647;87848;101003;101004;101005;101006;101007;101008;101009;101010;101011;101012;101013;101014 9071;81661;81662;81663;81664;81665;81666;81667;81668;81669;81670;81671;81672;81673;81674;81675;81676;127797;146738;146739;146740;146741;146742;146743;146744;146745;146746;146747;146748;146749;146750;146751;146752;146753;146754;146755 9071;81676;127797;146742;146755

A0A0A0MXY4;A0A1W2Q676;A0A0G2JU73;F1LP64 A0A0A0MXY4;A0A1W2Q676;A0A0G2JU73;F1LP64 1;1;1;1 1;1;1;1 1;1;1;1 E3 ubiquitin-protein ligase TRIP12 Trip12 tr|A0A0A0MXY4|A0A0A0MXY4_RAT E3 ubiquitin-protein ligase TRIP12 OS=Rattus norvegicus OX=10116 GN=Trip12 PE=1 SV=2;tr|A0A1W2Q676|A0A1W2Q676_RAT E3 ubiquitin-protein ligase TRIP12 OS=Rattus norvegicus OX=10116 GN=Trip12 PE=1 SV=1;tr|A0A0G2JU73|A0A0G2JU73_RAT 4 1 1 1 0 1 0 0 1 0 0 1 0 0 1 0 0 1 0 0 1 0 1.8 1.8 1.8 218.1 1975 1975;1976;2038;2025 2 3 0 14.777 By MS/MS By MS/MS 1.6596 3.2395 44.726 2 2 Median 1.6827 3.0461 68.27 2 2 Median 1.0139 0.83846 22.164 2 2 Median NaN 1.2476 NaN NaN 2.2077 NaN NaN 2.3612 NaN NaN 4.4446 NaN NaN NaN NaN NaN NaN NaN 0 1 0 0 1 0 0 1 0 0 1 0 Median Median Median Median Median Median NaN 1.0783 NaN NaN 2.6258 NaN NaN 1.8797 NaN NaN 4.9362 NaN NaN NaN NaN NaN NaN NaN 0 1 0 0 1 0 0 1 0 0 1 0 Median Median Median Median Median Median NaN 0.86435 NaN NaN 1.1894 NaN NaN 0.71684 NaN NaN 0.98072 NaN NaN NaN NaN NaN NaN NaN 0 1 0 0 1 0 0 1 0 0 1 0 Median Median Median Median Median Median 0 1.8 0 0 1.8 0 15740000 1855900 8965400 4919100 0 0 0 0 8713000 995260 5568900 2148900 0 0 0 0 0 0 0 0 7027300 860640 3396500 2770200 0 0 0 0 35 461 True 482 4072;4073;4074 5754;5755;5756 5754

A0A0A0MY00;P70584 A0A0A0MY00;P70584 6;6 6;6 6;6 Short/branched chain specific acyl-CoA dehydrogenase, mitochondrial Acadsb tr|A0A0A0MY00|A0A0A0MY00_RAT Short/branched chain-specific acyl-CoA dehydrogenase, mitochondrial OS=Rattus norvegicus OX=10116 GN=Acadsb PE=1 SV=2;sp|P70584|ACDSB_RAT Short/branched chain specific acyl-CoA dehydrogenase, mitochondrial OS=Rattus norvegicus 2 6 6 6 3 2 1 6 5 6 3 2 1 6 5 6 3 2 1 6 5 6 21.1 21.1 21.1 52.042 469 469;432 5.88 6 2 24 1 0 16.569 By MS/MS By MS/MS By MS/MS By MS/MS By MS/MS By MS/MS 0.26375 1.3294 39.353 26 17 Leave out requantified 0.3409 1.1262 19.023 24 15 Leave out requantified 1.0468 0.74111 30.093 24 15 Leave out requantified 0.262 0.29053 0.28854 0.27284 0.27186 0.31814 1.1779 1.489 1.2736 1.303 1.4438 1.2888 38.708 11.781 NaN 51.403 29.784 33.86 6 2 1 7 5 5 4 0 1 4 5 3 Median Median Median Leave out requantified Median Median 0.50818 0.31657 0.26232 0.30228 0.4383 0.34093 1.4764 1.1238 0.86292 1.1002 1.324 0.9564 27.532 24.329 NaN 27.705 21.145 34.191 5 2 1 7 5 4 3 0 1 4 5 2 Median Median Median Leave out requantified Median Median 1.9767 1.052 0.90911 0.97621 1.4957 1.0761 1.4803 0.77324 0.59778 0.66271 0.86699 0.72495 37.958 9.8089 NaN 57.548 22.085 26.613 5 2 1 7 5 4 3 0 1 4 5 2 Median Median Median Leave out requantified Median Median 8.5 4.7 2.8 21.1 16.6 21.1 112440000 71943000 18052000 22444000 11847000 8063800 1621800 2161800 8453400 5976700 1256800 1220000 3351200 3046600 136600 167960 26098000 14610000 5024800 6463500 35687000 23948000 4536400 7203000 27002000 16298000 5475200 5228200 36 3619;5095;6204;9980;11538;12336 True;True;True;True;True;True 3821;5360;6544;10581;12243;13085 32868;32869;46390;46391;46392;46393;46394;46395;46396;46397;56283;56284;56285;90021;90022;90023;105691;105692;105693;105694;105695;105696;114139;114140;114141;114142;114143;114144;114145;114146;114147;114148;114149 46078;46079;46080;66804;66805;66806;66807;66808;66809;66810;66811;82637;82638;82639;130851;130852;130853;153734;153735;153736;153737;153738;153739;165875;165876;165877;165878;165879;165880;165881;165882;165883;165884;165885 46078;66809;82638;130852;153739;165879 25;26;27;28 83;89;102;103

A0A0A0MY07;Q9R085 A0A0A0MY07;Q9R085 1;1 1;1 1;1 Ubiquitin carboxyl-terminal hydrolase;Ubiquitin carboxyl-terminal hydrolase 15 Usp15 tr|A0A0A0MY07|A0A0A0MY07_RAT Ubiquitin carboxyl-terminal hydrolase 15 OS=Rattus norvegicus OX=10116 GN=Usp15 PE=1 SV=1;sp|Q9R085|UBP15_RAT Ubiquitin carboxyl-terminal hydrolase 15 OS=Rattus norvegicus OX=10116 GN=Usp15 PE=1 SV=1 2 1 1 1 1 0 0 0 0 1 1 0 0 0 0 1 1 0 0 0 0 1 2.1 2.1 2.1 109.24 952 952;952 3.67 1 2 0.00054348 2.9572 By MS/MS By MS/MS 0.4001 0.73891 21.036 2 0 Median 0.56437 0.84972 6.9169 2 0 Median 1.4291 1.0718 15.724 2 0 Median 0.38779 NaN NaN NaN NaN 0.41281 0.63679 NaN NaN NaN NaN 0.85742 NaN NaN NaN NaN NaN NaN 1 0 0 0 0 1 0 0 0 0 0 0 Median Median Median Median Median Median 0.63805 NaN NaN NaN NaN 0.4992 0.80916 NaN NaN NaN NaN 0.89232 NaN NaN NaN NaN NaN NaN 1 0 0 0 0 1 0 0 0 0 0 0 Median Median Median Median Median Median 1.7619 NaN NaN NaN NaN 1.1592 1.1978 NaN NaN NaN NaN 0.95898 NaN NaN NaN NaN NaN NaN 1 0 0 0 0 1 0 0 0 0 0 0 Median Median Median Median Median Median 2.1 0 0 0 0 2.1 5950400 2970800 1258400 1721200 1417200 727520 319290 370420 0 0 0 0 0 0 0 0 0 0 0 0 0 0 0 0 4533200 2243300 939090 1350800 37 322 True 340 3105;3106;3107 4374;4375;4376;4377 4374

A0A0A0MY09;Q66HD0;A0A0G2K4I4 A0A0A0MY09;Q66HD0;A0A0G2K4I4 42;42;36 42;42;36 41;41;35 Endoplasmin Hsp90b1 tr|A0A0A0MY09|A0A0A0MY09_RAT Endoplasmin OS=Rattus norvegicus OX=10116 GN=Hsp90b1 PE=1 SV=1;sp|Q66HD0|ENPL_RAT Endoplasmin OS=Rattus norvegicus OX=10116 GN=Hsp90b1 PE=1 SV=2;tr|A0A0G2K4I4|A0A0G2K4I4_RAT Endoplasmin OS=Rattus norvegicus OX=10116 GN=Hsp90b1 3 42 42 41 24 32 32 27 31 34 24 32 32 27 31 34 24 31 31 26 30 33 58.8 58.8 56.7 92.899 804 804;804;643 4.08 13 16 115 384 69 29 11 6 4 6 0 323.31 By MS/MS By MS/MS By MS/MS By MS/MS By MS/MS By MS/MS 0.16154 0.63736 18.135 556 297 Leave out requantified 0.22625 0.64834 19.095 549 289 Leave out requantified 1.3959 1.0056 11.183 546 289 Leave out requantified 0.15577 0.15312 0.17528 0.15004 0.17021 0.16382 0.61476 0.61616 0.70749 0.59429 0.71084 0.63616 18.214 18.371 19.629 16 16.12 16.632 87 98 89 91 89 102 42 51 53 52 44 55 Leave out requantified Leave out requantified Leave out requantified Leave out requantified Leave out requantified Leave out requantified 0.21265 0.23535 0.22096 0.22681 0.23572 0.23274 0.56737 0.70309 0.64843 0.69602 0.65208 0.64415 15.212 18.843 16.367 19.765 9.4236 54.747 86 97 89 91 86 100 41 50 53 51 41 53 Leave out requantified Leave out requantified Leave out requantified Leave out requantified Leave out requantified Leave out requantified 1.343 1.5443 1.1797 1.5669 1.3687 1.435 0.93977 1.1725 0.87656 1.0675 0.94967 1.0324 11.243 14.603 12.542 9.3678 9.7899 18.355 86 97 89 91 86 97 41 50 53 51 41 53 Leave out requantified Leave out requantified Leave out requantified Leave out requantified Leave out requantified Leave out requantified 35.4 44 46.9 43.8 42.5 46 28869000000 20626000000 3473500000 4769500000 2521700000 1805800000 313160000 402800000 2945900000 2096600000 341960000 507270000 3982600000 2815800000 540840000 625960000 5512200000 3982100000 609680000 920430000 4977200000 3555400000 608300000 813490000 8929100000 6370000000 1059500000 1499600000 38 1042;1043;1515;2002;2003;2570;2571;2689;2692;2712;2713;2720;2823;2860;3180;3308;3444;3604;4715;4716;4738;5342;5519;5520;5674;6213;6255;6410;6901;7612;8575;8576;9225;9288;9289;9329;9728;10231;10232;12194;12195;12286 True;True;True;True;True;True;True;True;True;True;True;True;True;True;True;True;True;True;True;True;True;True;True;True;True;True;True;True;True;True;True;True;True;True;True;True;True;True;True;True;True;True 1105;1106;1601;2121;2122;2731;2732;2853;2856;2878;2879;2880;2881;2888;2994;3031;3363;3498;3638;3804;3805;3806;4967;4968;4990;5625;5822;5823;5824;5987;6553;6595;6757;7259;7260;8071;8072;9095;9096;9785;9850;9851;9892;10318;10845;10846;12934;12935;13033 9068;9069;9070;9071;9072;9073;9074;13465;18385;18386;18387;18388;18389;18390;18391;18392;18393;18394;18395;18396;18397;18398;18399;18400;18401;18402;18403;18404;18405;18406;18407;18408;18409;18410;18411;18412;18413;18414;18415;18416;18417;18418;18419;18420;18421;18422;18423;18424;18425;18426;18427;18428;18429;18430;18431;18432;18433;18434;18435;18436;18437;18438;18439;18440;18441;18442;18443;18444;18445;18446;18447;18448;18449;18450;18451;18452;18453;18454;18455;18456;18457;18458;18459;18460;18461;18462;18463;18464;18465;18466;18467;18468;18469;18470;18471;24405;24406;24407;24408;24409;24410;24411;24412;24413;24414;24415;24416;24417;24418;24419;24420;24421;24422;24423;24424;24425;24426;24427;24428;24429;24430;25430;25431;25432;25433;25434;25435;25436;25437;25438;25439;25440;25445;25446;25447;25448;25787;25788;25789;25790;25791;25792;25793;25794;25795;25796;25797;25798;25799;25800;25801;25802;25803;25804;25805;25806;25807;25808;25809;25810;25811;25812;25813;25814;25815;25816;25817;25818;25819;25820;25821;25822;25823;25824;25825;25826;25827;25828;25829;25853;25854;25855;25856;25857;25858;25859;25860;26663;26664;26665;26666;26667;26668;26669;26670;26671;26672;26673;26674;26675;26676;26677;26678;26679;26680;26681;26682;26683;26684;26685;26686;26687;26688;26689;26690;26691;26692;26693;26694;26695;26696;26697;27074;27075;27076;27077;27078;27079;27080;27081;27082;27083;27084;27085;27086;27087;27088;27089;27090;27091;27092;27093;27094;27095;27096;27097;29542;29543;29544;29545;29546;29547;29548;30372;31443;31444;31445;31446;31447;31448;31449;31450;31451;31452;31453;31454;31455;32783;32784;32785;32786;32787;32788;32789;32790;32791;32792;32793;32794;32795;32796;32797;32798;32799;32800;32801;32802;32803;32804;32805;42640;42641;42642;42643;42644;42645;42646;42647;42648;42649;42650;42651;42652;42653;42654;42655;42656;42657;42658;42659;42660;42661;42662;42663;42664;42665;42666;43032;43033;43034;43035;43036;43037;48425;48426;48427;48428;48429;48430;48431;48432;48433;48434;48435;48436;48437;48438;48439;48440;48441;48442;48443;48444;48445;48446;48447;48448;48449;48450;48451;48452;48453;48454;48455;50562;50563;50564;50565;50566;50567;50568;50569;50570;50571;50572;50573;50574;50575;50576;50577;50578;50579;50580;50581;50582;50583;50584;50585;50586;50587;50588;50589;51695;51696;51697;51698;51699;51700;51701;51702;51703;51704;51705;51706;56323;56324;56325;56326;56327;56328;56616;56617;56618;56619;56620;56621;56622;56623;56624;56625;56626;56627;56628;56629;56630;56631;56632;56633;56634;57958;62063;62064;62065;62066;62067;62068;62069;62070;62071;62072;62073;62074;62075;68546;68547;68548;68549;68550;68551;68552;68553;68554;68555;68556;68557;68558;68559;68560;68561;68562;68563;68564;68565;68566;68567;68568;68569;68570;68571;68572;68573;68574;68575;68576;68577;68578;68579;68580;68581;68582;68583;68584;68585;68586;68587;68588;68589;68590;68591;68592;68593;68594;68595;68596;68597;68598;68599;68600;68601;68602;68603;68604;68605;68606;68607;68608;68609;68610;68611;68612;68613;68614;68615;68616;68617;68618;68619;68620;68621;68622;68623;68624;68625;68626;68627;68628;68629;68630;77097;77098;77099;77100;77101;77102;77103;77104;77105;77106;77107;77108;82528;82529;82530;82531;82532;82533;82534;82535;82536;82537;82538;82539;82540;82541;82542;82543;82544;82545;82546;82547;82548;82549;82550;82551;82552;82553;82554;82555;82556;82557;82558;82559;82560;82561;82562;82563;82564;82565;82566;82567;82568;82569;82570;82571;83319;83320;83321;83322;83323;83324;83325;83326;83327;83328;83329;83330;83331;83332;83333;83334;83335;83336;83337;83338;83339;83340;83341;83342;83343;83344;83345;83346;83347;83348;83349;83350;83351;83352;83353;83354;83355;83356;83357;83358;83359;83360;83361;83362;83363;83833;87806;92067;92068;92069;92070;92071;92072;92073;92074;92075;92076;92077;92078;92079;92080;92081;112843;112844;112845;112846;112847;112848;112849;112850;112851;112852;112853;113700;113701;113702;113703;113704;113705 12992;12993;12994;12995;12996;12997;12998;12999;13000;19237;25899;25900;25901;25902;25903;25904;25905;25906;25907;25908;25909;25910;25911;25912;25913;25914;25915;25916;25917;25918;25919;25920;25921;25922;25923;25924;25925;25926;25927;25928;25929;25930;25931;25932;25933;25934;25935;25936;25937;25938;25939;25940;25941;25942;25943;25944;25945;25946;25947;25948;25949;25950;25951;25952;25953;25954;25955;25956;25957;25958;25959;25960;25961;25962;25963;25964;25965;25966;25967;25968;25969;25970;25971;25972;25973;25974;25975;25976;25977;25978;25979;25980;25981;25982;25983;25984;25985;25986;25987;25988;25989;25990;25991;25992;25993;25994;25995;25996;25997;25998;25999;26000;26001;26002;26003;26004;26005;26006;26007;26008;26009;26010;26011;26012;26013;26014;26015;26016;26017;26018;26019;26020;26021;26022;26023;26024;26025;26026;26027;26028;26029;26030;26031;26032;26033;26034;26035;26036;26037;26038;26039;26040;26041;26042;26043;26044;26045;26046;26047;26048;26049;26050;26051;34131;34132;34133;34134;34135;34136;34137;34138;34139;34140;34141;34142;34143;34144;34145;34146;34147;34148;34149;34150;34151;34152;34153;34154;34155;34156;34157;34158;34159;34160;34161;34162;34163;34164;34165;34166;34167;34168;34169;34170;34171;34172;34173;34174;34175;34176;35572;35573;35574;35575;35576;35577;35578;35579;35580;35581;35582;35583;35584;35591;35592;35593;35594;36037;36038;36039;36040;36041;36042;36043;36044;36045;36046;36047;36048;36049;36050;36051;36052;36053;36054;36055;36056;36057;36058;36059;36060;36061;36062;36063;36064;36065;36066;36067;36068;36069;36070;36071;36072;36073;36074;36075;36076;36077;36078;36079;36080;36081;36082;36083;36084;36085;36086;36087;36088;36089;36090;36091;36092;36093;36094;36095;36096;36097;36098;36099;36100;36101;36102;36103;36104;36105;36106;36107;36108;36109;36110;36111;36112;36113;36114;36115;36116;36117;36118;36119;36120;36121;36122;36123;36124;36125;36126;36127;36128;36129;36130;36131;36132;36133;36134;36135;36158;36159;36160;36161;36162;36163;36164;36165;36166;36167;36168;36169;36170;37429;37430;37431;37432;37433;37434;37435;37436;37437;37438;37439;37440;37441;37442;37443;37444;37445;37446;37447;37448;37449;37450;37451;37452;37453;37454;37455;37456;37457;37458;37459;37460;37461;37462;37463;37464;37465;37466;37467;37468;37469;37470;37471;37472;37473;37474;37475;37476;37477;37478;37479;37480;37481;37482;37483;37484;37485;37486;37487;37488;37489;37490;37491;37492;37493;37494;37495;37496;37497;37498;37499;37500;37501;37502;37503;37504;37505;37506;37507;37508;37509;37510;37511;37512;37513;37514;37515;37516;37517;37518;37519;37520;37521;37522;37523;37524;38020;38021;38022;38023;38024;38025;38026;38027;38028;38029;38030;38031;38032;38033;38034;38035;38036;38037;38038;38039;38040;38041;38042;38043;38044;38045;38046;38047;38048;38049;38050;38051;38052;38053;38054;38055;38056;38057;38058;38059;41418;41419;41420;41421;41422;42643;44090;44091;44092;44093;44094;44095;44096;44097;44098;44099;44100;44101;44102;44103;44104;44105;44106;44107;44108;44109;44110;44111;44112;44113;44114;45968;45969;45970;45971;45972;45973;45974;45975;45976;45977;45978;45979;45980;45981;45982;45983;45984;45985;45986;45987;45988;45989;45990;45991;45992;45993;45994;45995;45996;45997;45998;45999;46000;46001;46002;46003;60951;60952;60953;60954;60955;60956;60957;60958;60959;60960;60961;60962;60963;60964;60965;60966;60967;60968;60969;60970;60971;60972;60973;60974;60975;60976;60977;60978;60979;60980;60981;60982;60983;60984;60985;60986;60987;60988;60989;60990;60991;60992;60993;60994;61544;61545;61546;61547;61548;61549;69713;69714;69715;69716;69717;69718;69719;69720;69721;69722;69723;69724;69725;69726;69727;69728;69729;69730;69731;69732;69733;69734;69735;69736;69737;69738;69739;69740;69741;69742;69743;69744;69745;69746;69747;69748;69749;69750;69751;69752;69753;69754;69755;69756;69757;69758;69759;69760;69761;69762;69763;69764;69765;69766;69767;69768;69769;69770;69771;69772;69773;69774;72781;72782;72783;72784;72785;72786;72787;72788;72789;72790;72791;72792;72793;72794;72795;72796;72797;72798;72799;72800;72801;72802;72803;72804;72805;72806;72807;72808;72809;72810;72811;72812;72813;72814;72815;72816;72817;72818;72819;72820;72821;72822;72823;72824;72825;72826;72827;72828;72829;72830;72831;72832;72833;72834;72835;72836;72837;72838;72839;72840;72841;72842;72843;72844;72845;72846;74719;74720;74721;74722;74723;74724;74725;74726;74727;74728;74729;74730;74731;74732;74733;74734;74735;74736;74737;74738;74739;74740;74741;74742;74743;74744;74745;74746;74747;74748;74749;74750;74751;74752;74753;74754;74755;74756;74757;74758;82694;82695;82696;82697;82698;82699;82700;82701;82702;83069;83070;83071;83072;83073;83074;83075;83076;83077;83078;83079;83080;83081;83082;83083;83084;83085;83086;83087;83088;83089;83090;83091;83092;83093;83094;83095;83096;83097;83098;83099;83100;83101;83102;83103;85004;85005;90740;90741;90742;90743;90744;90745;90746;90747;90748;90749;90750;90751;90752;90753;90754;90755;99795;99796;99797;99798;99799;99800;99801;99802;99803;99804;99805;99806;99807;99808;99809;99810;99811;99812;99813;99814;99815;99816;99817;99818;99819;99820;99821;99822;99823;99824;99825;99826;99827;99828;99829;99830;99831;99832;99833;99834;99835;99836;99837;99838;99839;99840;99841;99842;99843;99844;99845;99846;99847;99848;99849;99850;99851;99852;99853;99854;99855;99856;99857;99858;99859;99860;99861;99862;99863;99864;99865;99866;99867;99868;99869;99870;99871;99872;99873;99874;99875;99876;99877;99878;99879;99880;99881;99882;99883;99884;99885;99886;99887;99888;99889;99890;99891;99892;99893;99894;99895;99896;99897;99898;99899;99900;99901;99902;99903;99904;99905;99906;99907;99908;99909;99910;99911;99912;99913;99914;99915;99916;99917;99918;99919;99920;99921;99922;99923;99924;99925;99926;99927;99928;99929;99930;99931;99932;99933;99934;99935;99936;99937;99938;99939;99940;99941;99942;99943;99944;99945;99946;99947;99948;99949;99950;99951;99952;99953;99954;99955;99956;99957;99958;99959;99960;99961;99962;99963;99964;99965;99966;99967;99968;99969;99970;99971;99972;99973;99974;99975;99976;99977;99978;99979;99980;99981;99982;99983;99984;99985;99986;99987;99988;99989;99990;99991;99992;99993;99994;99995;99996;99997;99998;99999;100000;100001;100002;100003;100004;100005;100006;112020;112021;112022;112023;112024;112025;112026;112027;112028;112029;112030;112031;112032;112033;112034;112035;112036;112037;112038;112039;112040;112041;112042;112043;112044;112045;112046;112047;112048;112049;119923;119924;119925;119926;119927;119928;119929;119930;119931;119932;119933;119934;119935;119936;119937;119938;119939;119940;119941;119942;119943;119944;119945;119946;119947;119948;119949;119950;119951;119952;119953;119954;119955;119956;119957;119958;119959;119960;119961;119962;119963;119964;119965;119966;119967;119968;119969;119970;119971;119972;119973;119974;119975;119976;119977;119978;119979;119980;119981;119982;119983;119984;119985;121275;121276;121277;121278;121279;121280;121281;121282;121283;121284;121285;121286;121287;121288;121289;121290;121291;121292;121293;121294;121295;121296;121297;121298;121299;121300;121301;121302;121303;121304;121305;121306;121307;121308;121309;121310;121311;121312;121313;121314;121315;121316;121317;121318;121319;121320;121321;121322;121323;121324;121325;121326;121327;121328;121329;121330;121331;121332;121333;121334;121335;121336;121337;121338;121339;121340;121341;121342;121343;121344;121345;121346;121347;121348;121349;121350;121351;121352;121353;121354;121355;121356;121357;121358;121359;121360;121361;121362;121363;121364;121365;121366;121979;127716;133686;133687;133688;133689;133690;133691;133692;133693;133694;133695;133696;133697;133698;133699;133700;133701;133702;133703;133704;133705;133706;133707;133708;133709;133710;133711;133712;133713;133714;133715;133716;133717;133718;133719;164122;164123;164124;164125;164126;164127;164128;164129;164130;164131;164132;164133;164134;164135;164136;164137;164138;165296;165297;165298;165299;165300;165301;165302;165303 12997;13000;19237;25900;26006;34164;34176;35583;35593;36071;36104;36161;37476;38048;41422;42643;44108;45977;60984;60994;61545;69750;72840;72846;74727;82698;83077;85005;90742;99967;112040;112049;119936;121351;121366;121979;127716;133686;133688;164132;164138;165299 29;30;31;32;33;34;35;36;37;38 85;86;154;425;426;531;541;622;658;662

A0A0A0MY31;F1LQX8;C7E1V2;F1LNT1;P29994;A0A0G2KAH9 A0A0A0MY31;F1LQX8;C7E1V2;F1LNT1;P29994;A0A0G2KAH9 12;12;12;12;12;9 11;11;11;11;11;9 11;11;11;11;11;9 Inositol 1,4,5-trisphosphate receptor type 1 Itpr1 tr|A0A0A0MY31|A0A0A0MY31_RAT Inositol 1,4,5-trisphosphate receptor type 1 OS=Rattus norvegicus OX=10116 GN=Itpr1 PE=1 SV=2;tr|F1LQX8|F1LQX8_RAT Inositol 1,4,5-trisphosphate receptor type 1 OS=Rattus norvegicus OX=10116 GN=Itpr1 PE=1 SV=3;tr|C7E1V2|C7E1V2_R 6 12 11 11 9 9 10 7 11 9 8 8 9 6 10 8 8 8 9 6 10 8 7.2 6.9 6.9 308.54 2709 2709;2733;2748;2773;2750;2574 1.23 52 9 3 0 97.404 By MS/MS By MS/MS By MS/MS By MS/MS By MS/MS By MS/MS 0.31129 0.84579 29.696 61 35 Leave out requantified 0.30623 0.7462 11.864 61 35 Leave out requantified 1.022 0.76595 23.154 61 35 Leave out requantified 0.23187 0.31822 0.2891 0.27645 0.31338 0.31452 0.58618 0.85844 0.97882 0.62971 0.84415 1.0392 35.11 29.824 27.456 11.623 30.161 15.873 10 8 13 6 12 12 6 4 8 3 6 8 Leave out requantified Leave out requantified Leave out requantified Leave out requantified Leave out requantified Leave out requantified 0.36404 0.2681 0.39727 0.29197 0.26246 0.29157 1.0268 0.69659 0.91787 0.61137 0.65552 0.73617 23.655 19.83 20.176 9.9381 21.09 15.857 10 8 13 6 12 12 6 4 8 3 6 8 Leave out requantified Leave out requantified Leave out requantified Leave out requantified Leave out requantified Leave out requantified 1.6593 1.1462 1.2349 0.91937 0.84436 0.85709 1.2209 0.87609 0.92448 0.65913 0.66262 0.6034 29.112 21.45 34.775 5.3387 27.832 17.424 10 8 13 6 12 12 6 4 8 3 6 8 Leave out requantified Leave out requantified Leave out requantified Leave out requantified Leave out requantified Leave out requantified 4.9 5.1 5.8 3.7 6.6 5.4 628000000 397070000 114470000 116460000 46101000 28213000 7374600 10513000 81924000 47389000 16872000 17663000 112060000 64948000 21680000 25434000 76064000 49054000 11638000 15373000 163210000 107770000 29822000 25621000 148640000 99695000 27085000 21859000 39 89;440;1280;1380;2577;3184;5096;6615;6852;8696;9297;9721 True;True;True;True;True;True;True;True;True;False;True;True 92;461;1352;1460;2738;3367;5361;6970;7210;9220;9859;10311 737;738;739;740;741;742;3960;3961;3962;11403;12259;12260;12261;12262;12263;12264;12265;24445;24446;24447;24448;24449;24450;24451;24452;24453;24454;24455;24456;24457;24458;24459;29561;29562;29563;29564;29565;46398;46399;46400;46401;59689;59690;59691;59692;59693;59694;61696;61697;61698;61699;61700;61701;61702;77856;77857;77858;77859;77860;77861;83435;83436;83437;83438;83439;87783;87784;87785;87786;87787 1006;1007;1008;1009;1010;1011;1012;1013;1014;5598;5599;5600;5601;5602;5603;16379;17555;17556;17557;17558;17559;17560;17561;34202;34203;34204;34205;34206;34207;34208;34209;34210;34211;34212;34213;34214;34215;34216;34217;34218;34219;41439;41440;41441;41442;41443;66812;66813;66814;66815;66816;66817;66818;66819;87520;87521;87522;87523;87524;87525;87526;90221;90222;90223;90224;90225;90226;90227;90228;90229;113232;113233;113234;113235;113236;113237;113238;121474;121475;121476;121477;121478;127691;127692;127693;127694;127695 1014;5598;16379;17558;34206;41439;66816;87520;90226;113236;121475;127693

A0A0A0MY43;F1LPQ2 A0A0A0MY43;F1LPQ2 2;2 2;2 2;2 Activating signal cointegrator 1 complex subunit 3 Ascc3 tr|A0A0A0MY43|A0A0A0MY43_RAT Activating signal cointegrator 1 complex subunit 3 OS=Rattus norvegicus OX=10116 GN=Ascc3 PE=1 SV=1;sp|F1LPQ2|ASCC3_RAT Activating signal cointegrator 1 complex subunit 3 OS=Rattus norvegicus OX=10116 GN=Ascc3 PE=3 SV=1 2 2 2 2 0 0 0 2 0 0 0 0 0 2 0 0 0 0 0 2 0 0 1.2 1.2 1.2 250.53 2201 2201;2197 1 2 0.0038443 2.1077 By MS/MS 0.49588 1.0876 29.106 2 2 Median 0.64917 1.3266 19.469 2 2 Median 1.3091 0.98354 9.5261 2 2 Median NaN NaN NaN 0.49588 NaN NaN NaN NaN NaN 1.0876 NaN NaN NaN NaN NaN 29.106 NaN NaN 0 0 0 2 0 0 0 0 0 2 0 0 Median Median Median Median Median Median NaN NaN NaN 0.64917 NaN NaN NaN NaN NaN 1.3266 NaN NaN NaN NaN NaN 19.469 NaN NaN 0 0 0 2 0 0 0 0 0 2 0 0 Median Median Median Median Median Median NaN NaN NaN 1.3091 NaN NaN NaN NaN NaN 0.98354 NaN NaN NaN NaN NaN 9.5261 NaN NaN 0 0 0 2 0 0 0 0 0 2 0 0 Median Median Median Median Median Median 0 0 0 1.2 0 0 17433000 9248600 3888400 4296300 0 0 0 0 0 0 0 0 0 0 0 0 17433000 9248600 3888400 4296300 0 0 0 0 0 0 0 0 40 7094;11224 True;True 7487;11903 63755;102495 92921;148867 92921;148867 39 425

A0A0A0MY48;A0A0A0MY49;P39052 A0A0A0MY48;A0A0A0MY49;P39052 1;1;1 1;1;1 1;1;1 Dynamin-2 Dnm2 tr|A0A0A0MY48|A0A0A0MY48_RAT Dynamin-2 OS=Rattus norvegicus OX=10116 GN=Dnm2 PE=1 SV=2;tr|A0A0A0MY49|A0A0A0MY49_RAT Dynamin-2 OS=Rattus norvegicus OX=10116 GN=Dnm2 PE=1 SV=2;sp|P39052|DYN2_RAT Dynamin-2 OS=Rattus norvegicus OX=10116 GN=Dnm2 PE=1 SV=1 3 1 1 1 0 0 0 0 1 1 0 0 0 0 1 1 0 0 0 0 1 1 1.7 1.7 1.7 97.253 860 860;869;870 4 2 0.0093666 1.5399 By MS/MS By MS/MS NaN NaN NaN 0 0 Median NaN NaN NaN 0 0 Median NaN NaN NaN 0 0 Median NaN NaN NaN NaN NaN NaN NaN NaN NaN NaN NaN NaN NaN NaN NaN NaN NaN NaN 0 0 0 0 0 0 0 0 0 0 0 0 Median Median Median Median Median Median NaN NaN NaN NaN NaN NaN NaN NaN NaN NaN NaN NaN NaN NaN NaN NaN NaN NaN 0 0 0 0 0 0 0 0 0 0 0 0 Median Median Median Median Median Median NaN NaN NaN NaN NaN NaN NaN NaN NaN NaN NaN NaN NaN NaN NaN NaN NaN NaN 0 0 0 0 0 0 0 0 0 0 0 0 Median Median Median Median Median Median 0 0 0 0 1.7 1.7 693990 693990 0 0 0 0 0 0 0 0 0 0 0 0 0 0 0 0 0 0 693990 693990 0 0 0 0 0 0 41 783 True 824 6781;6782 9732;9733 9732 40 332

A0A0G2JSG6;P29410 A0A0G2JSG6;P29410 3;2 3;2 3;2 Adenylate kinase 2, mitochondrial;Adenylate kinase 2, mitochondrial, N-terminally processed Ak2 tr|A0A0G2JSG6|A0A0G2JSG6_RAT Adenylate kinase 2, mitochondrial OS=Rattus norvegicus OX=10116 GN=Ak2 PE=1 SV=1;sp|P29410|KAD2_RAT Adenylate kinase 2, mitochondrial OS=Rattus norvegicus OX=10116 GN=Ak2 PE=2 SV=2 2 3 3 3 1 2 0 3 1 1 1 2 0 3 1 1 1 2 0 3 1 1 14.2 14.2 14.2 25.529 232 232;239 8.64 4 7 0 8.5928 By MS/MS By MS/MS By MS/MS By MS/MS By MS/MS 0.36873 1.5437 37.757 5 5 Median 0.42948 1.331 56.114 4 4 Median 1.0332 0.71537 24.709 4 4 Median 0.51872 0.35849 NaN 0.35047 NaN NaN 2.8303 1.6778 NaN 1.465 NaN NaN NaN 51.688 NaN 7.4041 NaN NaN 1 2 0 2 0 0 1 2 0 2 0 0 Median Median Median Median Median Median 0.50898 0.40262 NaN 0.36239 NaN NaN 1.7601 1.3936 NaN 1.0065 NaN NaN NaN 88.675 NaN NaN NaN NaN 1 2 0 1 0 0 1 2 0 1 0 0 Median Median Median Median Median Median 0.98122 1.1231 NaN 1.0879 NaN NaN 0.66788 0.87747 NaN 0.75303 NaN NaN NaN 36.141 NaN NaN NaN NaN 1 2 0 1 0 0 1 2 0 1 0 0 Median Median Median Median Median Median 5.6 11.2 0 14.2 5.6 5.6 12776000 10324000 1141000 1311300 447160 287970 86748 72434 5149700 3912200 420540 817030 0 0 0 0 4797100 3741600 633690 421830 2382100 2382100 0 0 0 0 0 0 42 1000;1338;6839 True;True;True 1062;1414;7197 8776;8777;8778;11916;61508;61509;61510;61511;61512;61513;61514 12609;12610;12611;17094;89978;89979;89980;89981;89982;89983;89984 12611;17094;89982 41 78

A0A0G2JSH2;P29147 A0A0G2JSH2;P29147 2;2 2;2 2;2 D-beta-hydroxybutyrate dehydrogenase, mitochondrial Bdh1 tr|A0A0G2JSH2|A0A0G2JSH2_RAT 3-hydroxybutyrate dehydrogenase, type 1, isoform CRA_a OS=Rattus norvegicus OX=10116 GN=Bdh1 PE=1 SV=1;sp|P29147|BDH_RAT D-beta-hydroxybutyrate dehydrogenase, mitochondrial OS=Rattus norvegicus OX=10116 GN=Bdh1 PE=1 SV=2 2 2 2 2 0 1 0 0 2 2 0 1 0 0 2 2 0 1 0 0 2 2 7.8 7.8 7.8 38.333 344 344;343 8 5 0 3.3608 By MS/MS By MS/MS By MS/MS 0.38272 1.8371 39.355 4 3 Median 0.35684 1.1452 16.967 3 2 Median 0.97985 0.75873 33.74 3 2 Median NaN 0.40388 NaN NaN 0.36266 0.27754 NaN 1.7982 NaN NaN 1.903 1.2611 NaN NaN NaN NaN NaN 56.234 0 1 0 0 1 2 0 1 0 0 1 1 Median Median Median Median Median Median NaN NaN NaN NaN 0.28847 0.38354 NaN NaN NaN NaN 0.92105 1.2139 NaN NaN NaN NaN NaN 8.2348 0 0 0 0 1 2 0 0 0 0 1 1 Median Median Median Median Median Median NaN NaN NaN NaN 0.79541 1.1866 NaN NaN NaN NaN 0.56012 0.91301 NaN NaN NaN NaN NaN 26.178 0 0 0 0 1 2 0 0 0 0 1 1 Median Median Median Median Median Median 0 2.9 0 0 7.8 7.8 6554100 3913100 1345000 1296000 0 0 0 0 529370 325800 133060 70517 0 0 0 0 0 0 0 0 3662100 2247600 784800 629690 2362600 1339700 427100 595840 43 1444;1469 True;True 1527;1553 12927;12928;12929;13125;13126 18507;18508;18509;18767;18768 18507;18768

A0A0G2JSH4;A0A0G2KB98;P18266 A0A0G2JSH4;A0A0G2KB98;P18266 1;1;1 1;1;1 1;1;1 Glycogen synthase kinase-3 beta Gsk3b tr|A0A0G2JSH4|A0A0G2JSH4_RAT Glycogen synthase kinase 3 beta, isoform CRA_b OS=Rattus norvegicus OX=10116 GN=Gsk3b PE=1 SV=1;tr|A0A0G2KB98|A0A0G2KB98_RAT Glycogen synthase kinase-3 beta OS=Rattus norvegicus OX=10116 GN=Gsk3b PE=1 SV=1;sp|P18266|GSK3B_RAT G 3 1 1 1 1 1 1 1 0 1 1 1 1 1 0 1 1 1 1 1 0 1 5.7 5.7 5.7 46.71 420 420;430;420 6.29 5 2 0 5.4214 By MS/MS By MS/MS By MS/MS By MS/MS By MS/MS 0.78882 3.8933 27.75 6 2 Median 0.96209 2.8636 12.248 6 2 Median 1.1998 0.88214 27.567 6 2 Median 0.62515 0.75083 0.61191 0.82874 NaN 1.121 3.4254 3.8713 2.2791 3.9154 NaN 4.8164 NaN NaN NaN NaN NaN 2.6818 1 1 1 1 0 2 0 0 1 0 0 1 Median Median Median Median Median Median 0.87463 0.95638 1.0186 0.99969 NaN 0.9068 3.1997 3.5381 2.6784 2.9901 NaN 2.6467 NaN NaN NaN NaN NaN 5.0277 1 1 1 1 0 2 0 0 1 0 0 1 Median Median Median Median Median Median 1.6166 1.086 1.6646 1.3256 NaN 0.86215 1.0914 0.84309 1.3289 0.923 NaN 0.66971 NaN NaN NaN NaN NaN 10.804 1 1 1 1 0 2 0 0 1 0 0 1 Median Median Median Median Median Median 5.7 5.7 5.7 5.7 0 5.7 26428000 9409400 8573700 8445300 3609800 1295600 910270 1403900 2417300 864800 707420 845060 2306300 1056700 512230 737410 4568600 1774800 1503900 1289900 0 0 0 0 13526000 4417400 4940000 4169100 44 11835 True 12561 109627;109628;109629;109630;109631;109632;109633 159683;159684;159685;159686;159687;159688;159689;159690;159691;159692;159693;159694;159695 159692

A0A0G2JSH6;Q9WUD2 A0A0G2JSH6;Q9WUD2 3;3 3;3 3;3 Transient receptor potential cation channel subfamily V member 2 Trpv2 tr|A0A0G2JSH6|A0A0G2JSH6_RAT Transient receptor potential cation channel subfamily V member 2 OS=Rattus norvegicus OX=10116 GN=Trpv2 PE=1 SV=1;sp|Q9WUD2|TRPV2_RAT Transient receptor potential cation channel subfamily V member 2 OS=Rattus norvegicus OX=1011 2 3 3 3 3 2 2 2 2 2 3 2 2 2 2 2 3 2 2 2 2 2 8.1 8.1 8.1 86.729 761 761;761 1 13 0 9.748 By MS/MS By MS/MS By MS/MS By MS/MS By MS/MS By MS/MS 0.43196 0.96582 28.087 5 2 Median 0.32522 0.78633 19.977 5 2 Median 0.81117 0.59044 30.204 5 2 Median 0.43823 0.58722 0.32029 0.43196 0.34071 NaN 1.1528 1.6224 0.76637 0.94324 0.96582 NaN NaN NaN NaN NaN NaN NaN 1 1 1 1 1 0 1 0 0 1 0 0 Median Median Median Median Median Median 0.42057 0.32522 0.41973 0.2779 0.28971 NaN 0.92549 0.78633 0.84548 0.56257 0.65975 NaN NaN NaN NaN NaN NaN NaN 1 1 1 1 1 0 1 0 0 1 0 0 Median Median Median Median Median Median 0.9597 0.51786 1.0675 0.64336 0.81117 NaN 0.69021 0.40772 0.88613 0.48511 0.59044 NaN NaN NaN NaN NaN NaN NaN 1 1 1 1 1 0 1 0 0 1 0 0 Median Median Median Median Median Median 8.1 5.7 5.7 5.7 4.7 4.7 45894000 27610000 9348200 8935400 10597000 6911900 1511600 2173300 10635000 4677100 3449600 2508100 6607300 3510500 1439900 1657000 8528100 5366900 1764900 1396400 6611900 4229000 1182300 1200700 2914600 2914600 0 0 45 7807;10494;10719 True;True;True 8288;11121;11363 70693;70694;70695;70696;70697;70698;94610;94611;94612;96784;96785;96786;96787 102982;102983;102984;102985;102986;102987;137481;137482;137483;140665;140666;140667;140668;140669;140670;140671 102986;137481;140668

A0A0G2JSH9;P35704 A0A0G2JSH9;P35704 3;2 3;2 3;2 Peroxiredoxin-2 Prdx2 tr|A0A0G2JSH9|A0A0G2JSH9_RAT Peroxiredoxin-2 OS=Rattus norvegicus OX=10116 GN=Prdx2 PE=1 SV=1;sp|P35704|PRDX2_RAT Peroxiredoxin-2 OS=Rattus norvegicus OX=10116 GN=Prdx2 PE=1 SV=3 2 3 3 3 1 0 1 3 2 2 1 0 1 3 2 2 1 0 1 3 2 2 25.8 25.8 25.8 21.797 198 198;198 9.5 10 7 1 0 12.237 By MS/MS By MS/MS By MS/MS By MS/MS By MS/MS 0.14395 0.65811 21.983 14 7 Leave out requantified 0.27584 0.92104 13.863 14 7 Leave out requantified 1.9088 1.2899 15.876 14 7 Leave out requantified NaN NaN 0.13246 0.14602 0.16515 0.58433 NaN NaN 0.55495 0.67688 0.86245 3.8823 NaN NaN 23.429 17.147 66.767 155.29 0 0 2 7 2 3 0 0 0 4 1 2 Median Median Median Median Median Median NaN NaN 0.25775 0.2825 0.30774 0.27692 NaN NaN 0.85751 0.91399 0.89237 1.0147 NaN NaN 2.6954 7.2943 10.61 23.75 0 0 2 7 2 3 0 0 0 4 1 2 Median Median Median Median Median Median NaN NaN 1.7243 1.8324 1.7924 0.4739 NaN NaN 1.2359 1.2209 1.331 0.28494 NaN NaN 24.062 14.216 20.15 149.65 0 0 2 7 2 3 0 0 0 4 1 2 Median Median Median Median Median Median 12.6 0 5.1 25.8 17.7 17.7 75842000 52448000 8934200 14460000 393680 393680 0 0 0 0 0 0 6342000 4402300 735420 1204300 47388000 34140000 4514200 8734100 8530200 5347700 781520 2401000 13188000 8164300 2903000 2121100 46 1209;7426;9470 True;True;True 1278;7880;10039 10573;10574;10575;10576;10577;10578;10579;10580;10581;66939;66940;66941;85262;85263;85264;85265;85266;85267 15197;15198;15199;15200;15201;15202;15203;15204;15205;15206;15207;15208;15209;97408;97409;97410;124133;124134;124135;124136;124137;124138;124139;124140;124141;124142;124143;124144 15206;97408;124137

A0A0G2JSI1;Q9JLJ3 A0A0G2JSI1;Q9JLJ3 9;8 9;8 9;8 4-trimethylaminobutyraldehyde dehydrogenase Aldh9a1 tr|A0A0G2JSI1|A0A0G2JSI1_RAT 4-trimethylaminobutyraldehyde dehydrogenase OS=Rattus norvegicus OX=10116 GN=Aldh9a1 PE=1 SV=1;sp|Q9JLJ3|AL9A1_RAT 4-trimethylaminobutyraldehyde dehydrogenase OS=Rattus norvegicus OX=10116 GN=Aldh9a1 PE=1 SV=1 2 9 9 9 9 8 9 9 6 8 9 8 9 9 6 8 9 8 9 9 6 8 26.6 26.6 26.6 54.05 497 497;494 5.17 14 1 3 22 55 1 2 4 0 78.006 By MS/MS By MS/MS By MS/MS By MS/MS By MS/MS By MS/MS 0.1212 0.67163 10.515 68 38 Leave out requantified 0.22175 0.83818 13.754 67 37 Leave out requantified 1.9286 1.4539 9.3978 67 37 Leave out requantified 0.099943 0.12044 0.13179 0.11274 0.23998 0.14076 0.53847 0.63451 0.67662 0.65489 1.0056 0.75993 8.4301 20.396 13.815 19.418 38.899 23.162 17 13 12 9 6 11 9 7 9 4 4 5 Leave out requantified Leave out requantified Leave out requantified Leave out requantified Median Leave out requantified 0.20954 0.16293 0.21251 0.2532 0.29817 0.25666 0.80365 0.59949 0.77862 0.90093 0.8409 0.97068 8.8286 11.725 7.4238 15.943 14.559 6.0618 17 13 12 8 6 11 9 7 9 3 4 5 Leave out requantified Leave out requantified Leave out requantified Leave out requantified Median Leave out requantified 2.1397 1.4157 1.815 2.2394 1.2922 1.9957 1.4736 0.97766 1.4137 1.4067 0.84716 1.5174 8.3593 17.134 10.824 29.28 40.357 21.583 17 13 12 8 6 11 9 7 9 3 4 5 Leave out requantified Leave out requantified Leave out requantified Leave out requantified Median Leave out requantified 26.6 24.5 26.6 26.6 19.3 24.5 802580000 589900000 73058000 139630000 103390000 75674000 9732500 17987000 64773000 48986000 6941900 8845300 129190000 94122000 14606000 20464000 121460000 92798000 8457800 20203000 53479000 40910000 4659300 7909500 330290000 237410000 28661000 64217000 47 417;2839;3207;3487;3547;4236;9901;10825;11696 True;True;True;True;True;True;True;True;True 437;3010;3390;3683;3746;4468;10498;10499;11476;11477;12412;12413 3757;3758;3759;3760;3761;3762;3763;3764;3765;26768;26769;26770;26771;26772;26773;26774;26775;26776;29719;29720;29721;29722;29723;29724;29725;29726;29727;29728;29729;29730;31727;31728;31729;31730;31731;31732;31733;31734;31735;31736;31737;31738;31739;31740;31741;32250;32251;32252;38307;38308;38309;38310;38311;38312;38313;38314;38315;38316;89152;89153;89154;89155;89156;89157;89158;89159;89160;89161;89162;89163;89164;89165;89166;89167;89168;89169;89170;89171;89172;89173;89174;89175;89176;89177;97858;97859;97860;97861;97862;97863;97864;97865;97866;97867;107870;107871;107872;107873;107874;107875;107876;107877 5253;5254;5255;5256;5257;5258;5259;5260;5261;5262;5263;5264;5265;5266;37608;37609;37610;37611;37612;37613;37614;37615;37616;41642;41643;41644;41645;41646;41647;41648;41649;41650;41651;41652;41653;41654;41655;41656;41657;41658;41659;41660;41661;41662;41663;41664;44485;44486;44487;44488;44489;44490;44491;44492;44493;44494;44495;44496;44497;44498;44499;44500;44501;44502;44503;44504;44505;44506;44507;45179;45180;45181;54216;54217;54218;54219;54220;54221;54222;54223;54224;54225;54226;54227;54228;54229;54230;129637;129638;129639;129640;129641;129642;129643;129644;129645;129646;129647;129648;129649;129650;129651;129652;129653;129654;129655;129656;129657;129658;129659;129660;129661;129662;129663;129664;129665;129666;129667;129668;129669;129670;129671;142130;142131;142132;142133;142134;142135;142136;142137;142138;142139;142140;157033;157034;157035;157036;157037;157038;157039;157040;157041 5262;37616;41663;44499;45179;54222;129644;142136;157035 42;43 238;487

A0A0G2JSJ3;A0A0G2JVB0;Q07647;A0A0G2JX37 A0A0G2JSJ3;A0A0G2JVB0;Q07647 3;3;3;1 3;3;3;1 3;3;3;1 Solute carrier family 2, facilitated glucose transporter member 3 Slc2a3 tr|A0A0G2JSJ3|A0A0G2JSJ3_RAT Solute carrier family 2 (Facilitated glucose transporter), member 3 OS=Rattus norvegicus OX=10116 GN=Slc2a3 PE=1 SV=1;tr|A0A0G2JVB0|A0A0G2JVB0_RAT Solute carrier family 2, facilitated glucose transporter member 3 OS=Rattus norv 4 3 3 3 2 2 2 2 3 3 2 2 2 2 3 3 2 2 2 2 3 3 5.9 5.9 5.9 53.562 493 493;495;493;482 1.57 17 10 2 1 0 8.144 By MS/MS By MS/MS By MS/MS By MS/MS By MS/MS By MS/MS 0.42194 1.1738 35.48 30 7 Leave out requantified 0.81108 1.8437 20.537 30 7 Leave out requantified 1.904 1.4104 7.473 30 7 Leave out requantified 0.46207 0.41911 0.38297 0.35236 0.37413 0.41218 1.2985 1.3646 1.7464 0.92983 1.2292 0.90572 9.2876 20.769 41.507 12.173 58 25.457 3 3 4 4 6 10 0 0 1 0 1 5 Median Median Linear Leave out requantified Median Median 0.76239 0.61719 0.73438 0.83243 0.84487 0.8223 1.7092 1.7116 2.4238 1.8657 2.0922 2.0788 9.8424 20.414 27.067 10.993 49.277 38.619 3 3 4 4 6 10 0 0 1 0 1 5 Median Plateau Linear Leave out requantified Leave out requantified Leave out requantified 1.7538 1.4943 1.8386 2.4333 1.9296 1.9667 1.3263 1.2424 1.4297 1.7371 1.4644 1.423 6.0929 5.1547 21.555 5.9442 5.2974 10.915 3 3 4 4 6 10 0 0 1 0 1 5 Median Median Median Leave out requantified Leave out requantified Leave out requantified 5.9 5.9 5.9 5.9 5.9 5.9 464060000 216190000 85000000 162870000 29368000 13114000 5719300 10535000 41057000 18792000 10002000 12263000 68208000 32680000 12647000 22882000 74978000 34260000 12406000 28312000 138200000 63515000 25251000 49433000 112250000 53828000 18975000 39442000 48 4559;4560;8447 True;True;True 4807;4808;8962 41351;41352;41353;41354;41355;41356;41357;41358;41359;41360;41361;41362;41363;41364;41365;41366;76277;76278;76279;76280;76281;76282;76283;76284;76285;76286;76287;76288;76289;76290 59234;59235;59236;59237;59238;59239;59240;59241;59242;59243;59244;59245;59246;59247;59248;59249;59250;59251;59252;59253;59254;59255;110762;110763;110764;110765;110766;110767;110768;110769;110770;110771;110772;110773;110774;110775;110776;110777;110778;110779 59235;59251;110768 44 483

A0A0G2JSK5;P49134 A0A0G2JSK5;P49134 22;18 22;18 22;18 Integrin beta-1 Itgb1 tr|A0A0G2JSK5|A0A0G2JSK5_RAT Integrin beta OS=Rattus norvegicus OX=10116 GN=Itgb1 PE=1 SV=1;sp|P49134|ITB1_RAT Integrin beta-1 OS=Rattus norvegicus OX=10116 GN=Itgb1 PE=2 SV=1 2 22 22 22 16 15 16 16 17 20 16 15 16 16 17 20 16 15 16 16 17 20 28.1 28.1 28.1 88.344 798 798;799 5.16 16 20 111 147 37 19 19 28 31 18 18 15 0 323.31 By MS/MS By MS/MS By MS/MS By MS/MS By MS/MS By MS/MS 1.0637 3.6978 25.111 446 74 Leave out requantified 1.6033 3.8452 16.765 446 74 Leave out requantified 1.5517 1.1285 10.976 446 74 Leave out requantified 1.2044 1.1162 1.1534 1.0069 0.99619 0.94435 4.2274 3.9464 3.9744 3.2653 3.2332 3.5195 27.239 18.385 30.921 28.003 28.217 30.913 69 64 71 81 82 79 12 8 12 10 13 19 Leave out requantified Leave out requantified Leave out requantified Leave out requantified Leave out requantified Leave out requantified 1.5804 1.5564 1.4926 1.651 1.6269 1.8284 3.6255 4.1674 3.4505 3.8213 3.8522 4.5577 22.442 14.31 26.675 23.777 21.51 31.449 69 64 71 81 82 79 12 8 12 10 13 19 Leave out requantified Leave out requantified Leave out requantified Leave out requantified Leave out requantified Leave out requantified 1.3174 1.4154 1.3045 1.7272 1.7094 1.9253 0.92434 1.1002 0.95481 1.2168 1.1886 1.3592 10.647 9.9959 2.4748 10.171 11.882 10.579 69 64 71 81 82 79 12 8 12 10 13 19 Leave out requantified Leave out requantified Leave out requantified Leave out requantified Leave out requantified Leave out requantified 21.7 21.3 21.7 21.7 24.3 24.1 13663000000 3508400000 3838700000 6315700000 1300700000 336650000 411500000 552570000 1136800000 292890000 346590000 497350000 1613800000 428750000 521320000 663700000 2720100000 713990000 723630000 1282500000 3059900000 788410000 839090000 1432400000 3831500000 947720000 996600000 1887200000 49 1656;1694;2678;2679;3829;4039;4040;5630;5631;6487;6507;6508;6607;6614;6637;7186;8489;8554;8996;8997;10870;11990 True;True;True;True;True;True;True;True;True;True;True;True;True;True;True;True;True;True;True;True;True;True 1750;1790;2842;2843;4045;4266;4267;5942;5943;6838;6858;6859;6962;6969;6992;7592;7593;9007;9074;9532;9533;11529;11530;12724 14524;14525;14526;14527;14528;14529;14530;14531;14532;14533;14534;14535;14536;14537;14538;14778;14779;14780;14781;14782;14783;14784;25376;25377;25378;25379;25380;25381;25382;25383;25384;25385;25386;25387;25388;25389;25390;25391;25392;25393;25394;25395;25396;25397;25398;25399;25400;25401;25402;25403;25404;25405;34427;34428;34429;34430;34431;34432;34433;34434;34435;34436;34437;34438;34439;34440;34441;34442;34443;34444;34445;34446;34447;34448;34449;36411;36412;36413;36414;36415;36416;36417;36418;36419;36420;36421;36422;36423;36424;36425;36426;36427;36428;36429;36430;36431;36432;36433;36434;36435;36436;36437;36438;36439;36440;36441;36442;51403;51404;58394;58395;58396;58397;58398;58399;58400;58401;58402;58403;58404;58405;58406;58407;58408;58409;58410;58411;58412;58550;58551;58552;58553;58554;58555;58556;58557;58558;58559;58560;58561;58562;58563;58564;58565;58566;58567;58568;58569;58570;58571;58572;58573;58574;58575;58576;58577;58578;58579;58580;58581;58582;58583;58584;58585;58586;58587;58588;58589;58590;58591;58592;58593;58594;58595;58596;58597;58598;58599;58600;58601;58602;58603;58604;58605;58606;58607;59624;59625;59626;59627;59628;59629;59630;59631;59632;59633;59634;59635;59636;59637;59638;59639;59640;59641;59642;59643;59644;59645;59646;59647;59648;59649;59650;59651;59652;59653;59654;59655;59675;59676;59677;59678;59679;59680;59681;59682;59683;59684;59685;59686;59687;59688;59885;64506;64507;64508;64509;64510;64511;64512;64513;64514;64515;64516;64517;64518;64519;64520;64521;64522;64523;64524;64525;64526;64527;64528;64529;64530;64531;64532;64533;64534;64535;64536;64537;64538;64539;64540;64541;64542;64543;64544;76535;76960;76961;76962;76963;76964;76965;76966;76967;76968;76969;76970;76971;80218;80219;80220;80221;80222;80223;80224;80225;80226;80227;80228;80229;80230;80231;80232;80233;80234;80235;80236;80237;80238;80239;80240;80241;80242;80243;80244;80245;80246;80247;80248;80249;80250;80251;80252;80253;80254;80255;80256;80257;80258;80259;80260;80261;80262;80263;80264;80265;80266;80267;80268;80269;80270;80271;80272;80273;80274;80275;80276;80277;80278;80279;80280;80281;80282;80283;80284;80285;80286;80287;80288;80289;80290;80291;80292;80293;80294;80295;80296;80297;80298;80299;80300;80301;80302;80303;80304;80305;80306;80307;80308;80309;80310;80311;80312;80313;80314;80315;80316;80317;80318;80319;80320;80321;80322;80323;80324;80325;80326;80327;80328;80329;80330;80331;80332;80333;80334;80335;80336;80337;80338;80339;80340;80341;80342;80343;80344;80345;80346;80347;80348;80349;80350;80351;80352;80353;80354;80355;80356;80357;80358;80359;80360;80361;80362;80363;80364;80365;80366;80367;80368;80369;80370;80371;98632;98633;98634;98635;98636;98637;98638;98639;98640;98641;98642;98643;98644;98645;98646;98647;98648;98649;98650;98651;98652;98653;98654;98655;98656;98657;98658;98659;98660;98661;98662;98663;98664;98665;98666;98667;98668;98669;98670;111094 20641;20642;20643;20644;20645;20646;20647;20648;20649;20650;20651;20652;20653;20654;20655;20656;20657;20658;20659;20660;20661;20662;20663;20664;20665;20666;20955;20956;20957;20958;20959;20960;20961;20962;20963;20964;20965;20966;20967;35501;35502;35503;35504;35505;35506;35507;35508;35509;35510;35511;35512;35513;35514;35515;35516;35517;35518;35519;35520;35521;35522;35523;35524;35525;35526;35527;35528;35529;35530;35531;35532;35533;35534;35535;35536;35537;35538;35539;35540;35541;35542;35543;35544;35545;35546;35547;35548;35549;48231;48232;48233;48234;48235;48236;48237;48238;48239;48240;48241;48242;48243;48244;48245;48246;48247;48248;48249;48250;48251;48252;48253;48254;48255;48256;48257;48258;48259;48260;48261;48262;48263;48264;48265;48266;48267;48268;48269;48270;48271;51371;51372;51373;51374;51375;51376;51377;51378;51379;51380;51381;51382;51383;51384;51385;51386;51387;51388;51389;51390;51391;51392;51393;51394;51395;51396;51397;51398;51399;51400;51401;51402;51403;51404;51405;51406;51407;51408;51409;51410;51411;51412;51413;51414;51415;51416;51417;51418;51419;51420;51421;51422;51423;51424;51425;51426;51427;51428;51429;51430;51431;51432;74283;74284;74285;74286;74287;74288;74289;74290;85588;85589;85590;85591;85592;85593;85594;85595;85596;85597;85598;85599;85600;85601;85602;85603;85604;85605;85606;85607;85608;85609;85610;85611;85612;85613;85614;85615;85616;85807;85808;85809;85810;85811;85812;85813;85814;85815;85816;85817;85818;85819;85820;85821;85822;85823;85824;85825;85826;85827;85828;85829;85830;85831;85832;85833;85834;85835;85836;85837;85838;85839;85840;85841;85842;85843;85844;85845;85846;85847;85848;85849;85850;85851;85852;85853;85854;85855;85856;85857;85858;85859;85860;85861;85862;85863;85864;85865;85866;85867;85868;85869;85870;85871;85872;85873;85874;85875;85876;85877;85878;85879;85880;85881;85882;85883;85884;85885;85886;85887;85888;85889;85890;85891;85892;85893;85894;85895;85896;85897;85898;85899;85900;85901;87406;87407;87408;87409;87410;87411;87412;87413;87414;87415;87416;87417;87418;87419;87420;87421;87422;87423;87424;87425;87426;87427;87428;87429;87430;87431;87432;87433;87434;87435;87436;87437;87438;87439;87440;87441;87442;87443;87444;87445;87446;87447;87448;87449;87450;87451;87452;87453;87454;87455;87456;87457;87458;87459;87460;87461;87462;87463;87464;87465;87466;87467;87492;87493;87494;87495;87496;87497;87498;87499;87500;87501;87502;87503;87504;87505;87506;87507;87508;87509;87510;87511;87512;87513;87514;87515;87516;87517;87518;87519;87839;93965;93966;93967;93968;93969;93970;93971;93972;93973;93974;93975;93976;93977;93978;93979;93980;93981;93982;93983;93984;93985;93986;93987;93988;93989;93990;93991;93992;93993;93994;93995;93996;93997;93998;93999;94000;94001;94002;94003;94004;94005;94006;94007;94008;94009;94010;94011;94012;94013;94014;94015;94016;94017;94018;94019;94020;94021;94022;94023;94024;94025;94026;94027;94028;94029;94030;94031;94032;94033;94034;94035;94036;94037;94038;94039;94040;94041;94042;94043;94044;94045;94046;94047;94048;94049;94050;94051;94052;94053;94054;94055;111128;111776;111777;111778;111779;111780;111781;111782;111783;111784;111785;111786;111787;111788;111789;111790;111791;116771;116772;116773;116774;116775;116776;116777;116778;116779;116780;116781;116782;116783;116784;116785;116786;116787;116788;116789;116790;116791;116792;116793;116794;116795;116796;116797;116798;116799;116800;116801;116802;116803;116804;116805;116806;116807;116808;116809;116810;116811;116812;116813;116814;116815;116816;116817;116818;116819;116820;116821;116822;116823;116824;116825;116826;116827;116828;116829;116830;116831;116832;116833;116834;116835;116836;116837;116838;116839;116840;116841;116842;116843;116844;116845;116846;116847;116848;116849;116850;116851;116852;116853;116854;116855;116856;116857;116858;116859;116860;116861;116862;116863;116864;116865;116866;116867;116868;116869;116870;116871;116872;116873;116874;116875;116876;116877;116878;116879;116880;116881;116882;116883;116884;116885;116886;116887;116888;116889;116890;116891;116892;116893;116894;116895;116896;116897;116898;116899;116900;116901;116902;116903;116904;116905;116906;116907;116908;116909;116910;116911;116912;116913;116914;116915;116916;116917;116918;116919;116920;116921;116922;116923;116924;116925;116926;116927;116928;116929;116930;116931;116932;116933;116934;116935;116936;116937;116938;116939;116940;116941;116942;116943;116944;116945;116946;116947;116948;116949;116950;116951;116952;116953;116954;116955;116956;116957;116958;116959;116960;116961;116962;116963;116964;116965;116966;116967;116968;116969;116970;116971;116972;116973;116974;116975;116976;116977;116978;116979;116980;116981;116982;116983;116984;116985;116986;116987;116988;116989;116990;116991;116992;116993;116994;116995;116996;116997;116998;116999;117000;117001;117002;117003;117004;117005;117006;117007;117008;117009;117010;117011;117012;117013;117014;117015;117016;117017;117018;117019;117020;117021;117022;117023;117024;117025;117026;117027;117028;117029;117030;117031;117032;117033;117034;117035;117036;117037;117038;117039;117040;117041;117042;117043;117044;143265;143266;143267;143268;143269;143270;143271;143272;143273;143274;143275;143276;143277;143278;143279;143280;143281;143282;143283;143284;143285;143286;143287;143288;143289;143290;143291;143292;143293;143294;143295;143296;143297;143298;143299;143300;143301;143302;143303;143304;143305;143306;143307;143308;143309;143310;143311;143312;143313;143314;143315;143316;143317;143318;143319;143320;143321;143322;143323;143324;143325;143326;143327;143328;143329;143330;143331;143332;143333;143334;143335;143336;143337;143338;143339;143340;143341;143342;143343;143344;143345;143346;143347;143348;143349;143350;143351;161640 20653;20965;35527;35539;48254;51384;51421;74283;74289;85611;85809;85901;87456;87514;87839;94031;111128;111789;116772;116920;143288;161640 45;46;47;48 149;155;193;771

A0A0G2JSK7;Q4JM44 A0A0G2JSK7;Q4JM44 1;1 1;1 1;1 Phosphatidylcholine:ceramide cholinephosphotransferase 2 Sgms2 tr|A0A0G2JSK7|A0A0G2JSK7_RAT Phosphatidylcholine:ceramide cholinephosphotransferase 2 OS=Rattus norvegicus OX=10116 GN=Sgms2 PE=4 SV=1;sp|Q4JM44|SMS2_RAT Phosphatidylcholine:ceramide cholinephosphotransferase 2 OS=Rattus norvegicus OX=10116 GN=Sgms2 PE=1 S 2 1 1 1 0 1 0 0 0 0 0 1 0 0 0 0 0 1 0 0 0 0 9.9 9.9 9.9 42.209 365 365;365 1 1 0.0028986 2.146 By MS/MS NaN NaN NaN 0 0 Median NaN NaN NaN 0 0 Median NaN NaN NaN 0 0 Median NaN NaN NaN NaN NaN NaN NaN NaN NaN NaN NaN NaN NaN NaN NaN NaN NaN NaN 0 0 0 0 0 0 0 0 0 0 0 0 Median Median Median Median Median Median NaN NaN NaN NaN NaN NaN NaN NaN NaN NaN NaN NaN NaN NaN NaN NaN NaN NaN 0 0 0 0 0 0 0 0 0 0 0 0 Median Median Median Median Median Median NaN NaN NaN NaN NaN NaN NaN NaN NaN NaN NaN NaN NaN NaN NaN NaN NaN NaN 0 0 0 0 0 0 0 0 0 0 0 0 Median Median Median Median Median Median 0 9.9 0 0 0 0 3085400 0 0 3085400 0 0 0 0 3085400 0 0 3085400 0 0 0 0 0 0 0 0 0 0 0 0 0 0 0 0 50 6971 True 7346 62740 91615 91615 49 1

D3ZLW4;A0A0G2JSL4;P49816 D3ZLW4;A0A0G2JSL4;P49816 3;3;3 3;3;3 3;3;3 Tuberin Tsc2 tr|D3ZLW4|D3ZLW4_RAT Tuberin OS=Rattus norvegicus OX=10116 GN=Tsc2 PE=1 SV=3;tr|A0A0G2JSL4|A0A0G2JSL4_RAT Tuberin OS=Rattus norvegicus OX=10116 GN=Tsc2 PE=1 SV=1;sp|P49816|TSC2_RAT Tuberin OS=Rattus norvegicus OX=10116 GN=Tsc2 PE=1 SV=1 3 3 3 3 2 1 3 1 3 3 2 1 3 1 3 3 2 1 3 1 3 3 2.4 2.4 2.4 198.6 1785 1785;1809;1809 1.8 10 1 1 3 0 36.339 By MS/MS By MS/MS By MS/MS By MS/MS By MS/MS By MS/MS 0.57598 1.6018 24.149 12 6 Leave out requantified 0.81232 1.7834 12.592 12 6 Leave out requantified 1.5337 1.2006 1.9665 12 6 Leave out requantified 0.60085 0.53875 0.56377 0.52044 0.60223 0.64755 1.4692 1.0999 1.519 1.1253 1.8534 1.4102 25.214 NaN 22.774 NaN 12.402 28.374 3 1 2 1 2 3 2 1 0 1 0 2 Median Median Median Median Median Median 0.68756 0.51517 0.60337 0.67014 1.0704 0.76492 1.2479 0.89509 1.2885 1.3563 2.4685 1.8489 2.9294 NaN 14.84 NaN 40.025 15.788 3 1 2 1 2 3 2 1 0 1 0 2 Median Median Median Median Median Plateau 1.7308 0.95622 1.1074 1.2876 1.8727 1.312 1.2494 0.80918 0.89048 0.97719 1.4762 1.1841 19.337 NaN 22.168 NaN 31.274 40.363 3 1 2 1 2 3 2 1 0 1 0 2 Plateau Median Median Median Median Median 1.6 1.1 2.4 1.1 2.4 2.4 94811000 43670000 21604000 29537000 9596600 4756900 1922800 2917000 5040400 2452300 981380 1606800 12522000 6085700 2696800 3739500 7800300 3789400 1776000 2234900 27084000 11367000 6411700 9305100 32768000 15218000 7815600 9734000 51 8112;8690;11519 True;True;True 8613;9214;12224 73614;73615;73616;73617;77840;77841;77842;105436;105437;105438;105439;105440;105441;105442;105443 107146;107147;107148;107149;113216;113217;113218;153357;153358;153359;153360;153361;153362;153363;153364;153365;153366 107146;113216;153364

A0A0G2JSM4;O35152 A0A0G2JSM4;O35152 2;1 2;1 2;1 BET1-like protein Bet1l tr|A0A0G2JSM4|A0A0G2JSM4_RAT BET1-like protein OS=Rattus norvegicus OX=10116 GN=Bet1l PE=1 SV=1;sp|O35152|BET1L_RAT BET1-like protein OS=Rattus norvegicus OX=10116 GN=Bet1l PE=1 SV=1 2 2 2 2 0 1 1 1 0 0 0 1 1 1 0 0 0 1 1 1 0 0 27 27 27 12.41 111 111;111 11.3 2 1 0 3.7394 By MS/MS By MS/MS By MS/MS 0.57778 3.0779 21.881 3 2 Median 1.0689 3.6265 34.154 3 2 Median 1.9089 1.3778 20.773 3 2 Median NaN 0.55996 0.57778 0.86626 NaN NaN NaN 2.8224 3.0779 4.2734 NaN NaN NaN NaN NaN NaN NaN NaN 0 1 1 1 0 0 0 1 0 1 0 0 Median Median Median Median Median Median NaN 1.0689 0.80138 1.6843 NaN NaN NaN 3.6265 2.7477 5.4198 NaN NaN NaN NaN NaN NaN NaN NaN 0 1 1 1 0 0 0 1 0 1 0 0 Median Median Median Median Median Median NaN 1.9089 1.3554 1.9443 NaN NaN NaN 1.3778 0.96784 1.3957 NaN NaN NaN NaN NaN NaN NaN NaN 0 1 1 1 0 0 0 1 0 1 0 0 Median Median Median Median Median Median 0 18.9 8.1 18.9 0 0 8368200 2938000 1735000 3695100 0 0 0 0 5309300 1907800 1155500 2246100 782800 371820 186740 224240 2276000 658380 392850 1224800 0 0 0 0 0 0 0 0 52 286;8723 True;True 304;9250 2679;2680;78009 3771;3772;113444 3772;113444

A0A0G2JSP3;B5DF89 A0A0G2JSP3;B5DF89 9;9 9;9 9;9 Cullin-3 Cul3 tr|A0A0G2JSP3|A0A0G2JSP3_RAT Cullin-3 OS=Rattus norvegicus OX=10116 GN=Cul3 PE=1 SV=1;sp|B5DF89|CUL3_RAT Cullin-3 OS=Rattus norvegicus OX=10116 GN=Cul3 PE=1 SV=2 2 9 9 9 3 3 3 4 8 7 3 3 3 4 8 7 3 3 3 4 8 7 14.3 14.3 14.3 86.467 746 746;768 4 30 0 43.922 By MS/MS By MS/MS By MS/MS By MS/MS By MS/MS By MS/MS 0.44838 1.1825 23.595 27 9 Leave out requantified 0.56771 1.3825 21.319 27 9 Leave out requantified 1.3041 1.0179 16.407 26 9 Leave out requantified 0.32345 0.34127 0.61144 0.3138 0.44054 0.40526 1.0438 0.94445 1.7975 1.2369 1.3185 1.0913 15.228 36.854 28.052 53.533 21.59 29.643 3 4 2 4 8 6 1 3 0 1 2 2 Median Median Median Leave out requantified Leave out requantified Leave out requantified 0.74205 0.53614 0.53767 0.72802 0.59678 0.55465 1.779 1.4703 1.2306 1.7435 1.4001 1.1656 25.416 25.627 19.67 14.467 15.179 22.917 3 4 2 4 8 6 1 3 0 1 2 2 Plateau Median Median Leave out requantified Leave out requantified Leave out requantified 2.4705 1.4339 1.0492 2.2918 1.4615 1.4344 1.7101 1.1416 0.81328 1.6293 1.1059 1.1287 22.855 44.931 29.452 62.105 16.164 21.078 3 4 2 4 7 6 1 3 0 1 2 2 Plateau Median Median Leave out requantified Leave out requantified Leave out requantified 4.7 5.1 5.4 5.9 13.1 11.8 214220000 105730000 42938000 65552000 9721600 4831200 1651500 3238800 29103000 15158000 5826600 8118600 28223000 13529000 7761800 6931700 37383000 17757000 5863100 13764000 62900000 30489000 12309000 20101000 46891000 23967000 9526100 13398000 53 865;2852;3730;4341;7382;7746;9888;10539;12327 True;True;True;True;True;True;True;True;True 911;3023;3937;4574;7829;8225;10484;11168;13076 7521;7522;7523;7524;26936;26937;26938;26939;33639;33640;33641;33642;33643;39041;39042;39043;39044;39045;39046;66508;70100;70101;70102;70103;88994;88995;94967;94968;114055;114056 10812;10813;10814;10815;10816;10817;37842;37843;37844;37845;47114;47115;47116;47117;47118;55293;55294;55295;55296;55297;55298;55299;55300;55301;55302;55303;96852;102088;102089;102090;102091;129402;129403;138041;138042;165775;165776;165777 10816;37844;47117;55293;96852;102088;129402;138041;165775

A0A0G2JSP8;P00564 A0A0G2JSP8;P00564 3;3 3;3 3;3 Creatine kinase M-type Ckm tr|A0A0G2JSP8|A0A0G2JSP8_RAT Creatine kinase M-type OS=Rattus norvegicus OX=10116 GN=Ckm PE=1 SV=1;sp|P00564|KCRM_RAT Creatine kinase M-type OS=Rattus norvegicus OX=10116 GN=Ckm PE=1 SV=2 2 3 3 3 3 0 0 0 0 0 3 0 0 0 0 0 3 0 0 0 0 0 8.9 8.9 8.9 43.018 381 381;381 9 3 0 35.815 By MS/MS 0.038836 0.15524 116.46 2 2 Median 0.019937 0.05735 0.32984 2 2 Median 0.51336 0.33166 116.79 2 2 Median 0.038836 NaN NaN NaN NaN NaN 0.15524 NaN NaN NaN NaN NaN 116.46 NaN NaN NaN NaN NaN 2 0 0 0 0 0 2 0 0 0 0 0 Median Median Median Median Median Median 0.019937 NaN NaN NaN NaN NaN 0.05735 NaN NaN NaN NaN NaN 0.32984 NaN NaN NaN NaN NaN 2 0 0 0 0 0 2 0 0 0 0 0 Median Median Median Median Median Median 0.51336 NaN NaN NaN NaN NaN 0.33166 NaN NaN NaN NaN NaN 116.79 NaN NaN NaN NaN NaN 2 0 0 0 0 0 2 0 0 0 0 0 Median Median Median Median Median Median 8.9 0 0 0 0 0 63692000 61466000 1081900 1143400 63692000 61466000 1081900 1143400 0 0 0 0 0 0 0 0 0 0 0 0 0 0 0 0 0 0 0 0 54 4525;6692;7965 True;True;True 4772;7047;8455 41207;60265;72566 59012;59013;88335;105557;105558 59012;88335;105558

A0A0G2JSR0;Q9R1Z0 A0A0G2JSR0;Q9R1Z0 9;9 8;8 8;8 Voltage-dependent anion-selective channel protein 3 Vdac3 tr|A0A0G2JSR0|A0A0G2JSR0_RAT Voltage-dependent anion-selective channel protein 3 OS=Rattus norvegicus OX=10116 GN=Vdac3 PE=1 SV=1;sp|Q9R1Z0|VDAC3_RAT Voltage-dependent anion-selective channel protein 3 OS=Rattus norvegicus OX=10116 GN=Vdac3 PE=1 SV=2 2 9 8 8 8 9 7 8 8 8 7 8 6 7 7 7 7 8 6 7 7 7 39.6 36 36 30.783 283 283;283 5.78 37 11 1 1 12 45 24 4 2 0 111.91 By MS/MS By MS/MS By MS/MS By MS/MS By MS/MS By MS/MS 0.13291 0.60915 21.236 98 55 Leave out requantified 0.15756 0.46475 20.193 95 52 Leave out requantified 1.3708 0.9927 8.8542 95 52 Leave out requantified 0.12444 0.15922 0.11785 0.10507 0.13291 0.14337 0.71208 0.55501 0.6208 0.46979 0.68932 0.64812 31.772 6.7592 8.431 41.239 27.42 19.714 11 12 12 20 19 24 6 7 6 15 10 11 Leave out requantified Leave out requantified Leave out requantified Leave out requantified Leave out requantified Leave out requantified 0.16164 0.19251 0.16993 0.19596 0.15756 0.16543 0.58423 0.51045 0.57214 0.46496 0.48994 0.51936 14.311 13.774 34.818 24.064 49.449 16.303 11 12 11 20 19 22 6 7 5 15 10 9 Leave out requantified Leave out requantified Leave out requantified Leave out requantified Leave out requantified Leave out requantified 1.5099 1.1531 1.444 1.5435 1.25 1.2316 0.98849 0.8803 0.99863 0.98165 0.95723 0.95738 15.754 8.7332 11.228 18.379 40.55 16.392 11 12 11 20 19 22 6 7 5 15 10 9 Leave out requantified Leave out requantified Leave out requantified Leave out requantified Leave out requantified Leave out requantified 33.9 39.6 26.9 33.9 35.7 33.9 1407200000 1049800000 145270000 212130000 132260000 97267000 14135000 20858000 195890000 148430000 22328000 25132000 121460000 93212000 12095000 16152000 315910000 231850000 30219000 53844000 360660000 271020000 36142000 53502000 281010000 208020000 30350000 42638000 55 1688;6103;6732;6767;6768;9016;11058;11487;12008 True;True;False;True;True;True;True;True;True 1784;6437;7087;7123;7124;9555;11728;12192;12743 14746;14747;14748;14749;14750;14751;14752;14753;14754;14755;14756;14757;14758;14759;14760;14761;14762;14763;55196;55197;55198;55199;55200;55201;55202;55203;55204;55205;55206;60569;60570;60571;60572;60573;60574;60575;60576;60577;60578;60579;60580;60581;60582;60583;60584;60585;60586;60587;60588;60589;60590;60591;60592;60593;60594;60595;60596;60597;60598;60599;60600;60601;60602;60603;60604;60605;60606;60607;60608;60609;60610;60611;60612;60613;60614;60615;60616;60617;60618;60619;60620;60621;60622;60623;60624;60625;60626;60627;60628;60629;60630;60631;60632;60633;60634;60635;60636;60637;60836;60837;60838;60839;60840;60841;60842;60843;60844;60845;60846;60847;60848;60849;60850;60851;60852;60853;60854;60855;60856;60857;60858;60859;60860;60861;60862;60863;60864;60865;60866;60867;60868;60869;60870;60871;60872;60873;60874;60875;60876;60877;60878;60879;60880;60881;60882;60883;60884;80516;80517;80518;80519;80520;80521;80522;80523;80524;80525;80526;80527;80528;80529;80530;80531;80532;80533;80534;80535;80536;80537;80538;80539;80540;80541;80542;80543;80544;80545;80546;80547;80548;80549;80550;80551;80552;80553;80554;80555;100682;100683;100684;100685;100686;100687;100688;100689;105062;105063;105064;105065;105066;105067;105068;105069;105070;111191;111192 20912;20913;20914;20915;20916;20917;20918;20919;20920;20921;20922;20923;20924;20925;20926;20927;20928;20929;20930;20931;20932;20933;20934;20935;20936;20937;20938;81149;81150;81151;81152;81153;81154;81155;81156;81157;81158;81159;88731;88732;88733;88734;88735;88736;88737;88738;88739;88740;88741;88742;88743;88744;88745;88746;88747;88748;88749;88750;88751;88752;88753;88754;88755;88756;88757;88758;88759;88760;88761;88762;88763;88764;88765;88766;88767;88768;88769;88770;88771;88772;88773;88774;88775;88776;88777;88778;88779;88780;88781;88782;88783;88784;88785;88786;88787;88788;88789;88790;88791;88792;88793;88794;88795;88796;88797;88798;88799;88800;88801;88802;88803;88804;88805;88806;88807;88808;88809;88810;88811;88812;88813;88814;88815;88816;88817;88818;88819;88820;88821;88822;88823;88824;88825;88826;88827;89083;89084;89085;89086;89087;89088;89089;89090;89091;89092;89093;89094;89095;89096;89097;89098;89099;89100;89101;89102;89103;89104;89105;89106;89107;89108;89109;89110;89111;89112;89113;89114;89115;89116;89117;89118;89119;89120;89121;89122;89123;89124;89125;89126;89127;89128;89129;89130;89131;89132;89133;89134;89135;89136;89137;89138;89139;89140;89141;89142;117207;117208;117209;117210;117211;117212;117213;117214;117215;117216;117217;117218;117219;117220;117221;117222;117223;117224;117225;117226;117227;117228;117229;117230;117231;117232;117233;117234;117235;117236;117237;117238;117239;117240;117241;117242;117243;117244;117245;117246;117247;117248;117249;117250;117251;117252;117253;117254;117255;117256;117257;117258;146308;146309;146310;146311;146312;146313;146314;146315;152826;152827;152828;152829;152830;152831;152832;152833;152834;152835;152836;152837;152838;161761;161762 20918;81157;88788;89091;89125;117215;146311;152833;161762

A0A0G2JSR7;P43244 A0A0G2JSR7;P43244 6;6 6;6 6;6 Matrin-3 Matr3 tr|A0A0G2JSR7|A0A0G2JSR7_RAT Matrin-3 OS=Rattus norvegicus OX=10116 GN=Matr3 PE=1 SV=1;sp|P43244|MATR3_RAT Matrin-3 OS=Rattus norvegicus OX=10116 GN=Matr3 PE=1 SV=2 2 6 6 6 3 3 4 4 2 2 3 3 4 4 2 2 3 3 4 4 2 2 11.5 11.5 11.5 94.501 845 845;845 3.5 13 13 0 113 By MS/MS By MS/MS By MS/MS By MS/MS By MS/MS By MS/MS 0.48306 1.2882 18.766 20 6 Leave out requantified 0.58844 1.1977 15.29 21 6 Leave out requantified 1.1418 0.93576 4.4825 21 6 Leave out requantified 0.51793 0.49133 0.43297 0.4995 0.51328 0.46578 1.7524 1.3437 1.4549 1.0933 1.051 1.1804 21.262 15.157 17.909 0.50546 2.0047 33.383 3 4 5 4 2 2 1 2 1 0 1 1 Median Median Leave out requantified Leave out requantified Median Median 0.58232 0.55663 0.50184 0.55716 0.58012 0.63299 1.1977 1.1802 1.0878 1.011 1.0379 1.3392 8.146 25.268 12.126 7.1177 33.818 15.258 3 4 6 4 2 2 1 2 1 0 1 1 Median Median Leave out requantified Leave out requantified Median Median 1.1292 1.1842 1.1361 1.1102 1.1399 1.4658 0.68162 0.82748 0.89342 0.84836 0.90648 1.1841 14.448 21.752 5.4695 12.733 14.017 5.9554 3 4 6 4 2 2 1 2 1 0 1 1 Median Median Leave out requantified Leave out requantified Median Median 4.6 4.6 6.9 6.4 4 3.2 116520000 57304000 26625000 32593000 17671000 8691000 4017600 4962900 16696000 7821700 4113100 4761300 35897000 18516000 7591600 9789100 21681000 10297000 5720500 5663200 7832500 4008200 1618100 2206300 16743000 7969600 3563700 5209900 56 5445;6017;6105;7450;8056;10192 True;True;True;True;True;True 5745;6347;6439;7905;8552;10803 49895;49896;49897;49898;49899;49900;49901;49902;54707;54708;54709;54710;54711;55213;55214;55215;55216;55217;55218;55219;67083;67084;73134;91792;91793;91794 71872;71873;71874;71875;71876;71877;71878;71879;71880;80526;80527;81166;81167;81168;81169;81170;81171;81172;81173;81174;81175;97615;97616;106340;106341;133291;133292;133293 71880;80527;81169;97616;106340;133291

A0A0G2JSS8;Q9R063 A0A0G2JSS8;Q9R063 5;5 5;5 5;5 Peroxiredoxin-5, mitochondrial Prdx5 tr|A0A0G2JSS8|A0A0G2JSS8_RAT Peroxiredoxin 5, isoform CRA_c OS=Rattus norvegicus OX=10116 GN=Prdx5 PE=1 SV=1;sp|Q9R063|PRDX5_RAT Peroxiredoxin-5, mitochondrial OS=Rattus norvegicus OX=10116 GN=Prdx5 PE=1 SV=1 2 5 5 5 2 2 1 4 4 2 2 2 1 4 4 2 2 2 1 4 4 2 23.5 23.5 23.5 22.206 213 213;213 10.7 2 10 12 5 0 25.384 By MS/MS By MS/MS By MS/MS By MS/MS By MS/MS By MS/MS 0.24692 1.2706 8.714 24 6 Leave out requantified 0.32124 1.0765 13.462 24 6 Leave out requantified 1.4018 0.88432 6.87 24 6 Leave out requantified 0.25412 0.25663 0.27116 0.25947 0.23943 0.25923 1.5114 1.1974 1.2673 1.3051 1.2172 1.4192 27.906 14.451 8.0869 6.0443 20.493 15.27 3 2 3 6 6 4 1 0 0 2 3 0 Median Median Median Leave out requantified Median Leave out requantified 0.25123 0.24539 0.25945 0.32583 0.39117 0.34014 0.82399 0.84187 0.8285 1.0798 1.1735 1.0978 23.193 2.7263 3.9077 2.8633 7.9696 15.601 3 2 3 6 6 4 1 0 0 2 3 0 Median Median Median Leave out requantified Median Leave out requantified 1.272 0.91433 1.0564 1.3197 1.5751 1.3353 0.8814 0.67235 0.75432 0.87345 1.0797 0.87433 19.823 16.46 18.696 5.0787 12.028 3.3549 3 2 3 6 6 4 1 0 0 2 3 0 Median Median Median Leave out requantified Median Leave out requantified 8.5 8.5 8 16 19.7 8.5 147960000 91933000 24554000 31474000 6650000 4393100 1093200 1163600 18599000 12644000 3265000 2689800 14067000 9298400 2187200 2581400 42689000 26085000 7470900 9132900 38285000 22913000 5318100 10054000 27672000 16600000 5219800 5852600 57 4687;5953;8578;11158;11159 True;True;True;True;True 4939;6281;9098;11831;11832 42468;54185;77111;77112;101603;101604;101605;101606;101607;101608;101609;101610;101611;101612;101613;101614;101615;101616;101617;101618;101619;101620;101621;101622;101623;101624;101625;101626;101627 60738;79754;112052;112053;147593;147594;147595;147596;147597;147598;147599;147600;147601;147602;147603;147604;147605;147606;147607;147608;147609;147610;147611;147612;147613;147614;147615;147616;147617;147618;147619;147620;147621;147622;147623;147624;147625;147626;147627;147628;147629;147630;147631;147632;147633;147634;147635 60738;79754;112053;147608;147628

A0A0G2JSS9;Q0ZHH6 A0A0G2JSS9;Q0ZHH6 6;5 6;5 6;5 Atlastin-3 Atl3 tr|A0A0G2JSS9|A0A0G2JSS9_RAT Atlastin-3 OS=Rattus norvegicus OX=10116 GN=Atl3 PE=1 SV=1;sp|Q0ZHH6|ATLA3_RAT Atlastin-3 OS=Rattus norvegicus OX=10116 GN=Atl3 PE=2 SV=2 2 6 6 6 3 3 4 2 2 4 3 3 4 2 2 4 3 3 4 2 2 4 17.9 17.9 17.9 60.249 536 536;541 4.65 11 2 19 27 1 0 56.172 By MS/MS By MS/MS By MS/MS By MS/MS By MS/MS By MS/MS 0.25922 1.2972 12.966 50 18 Leave out requantified 0.30439 1.1216 24.707 48 17 Leave out requantified 1.0459 0.82 19.096 48 17 Leave out requantified 0.29279 0.29096 0.28757 0.25419 0.27859 0.25379 1.5 1.356 1.3857 1.394 1.3932 1.0575 21.721 11.752 20.625 5.7011 43.526 16.507 7 9 11 7 7 9 2 2 4 3 3 4 Leave out requantified Leave out requantified Leave out requantified Leave out requantified Median Leave out requantified 0.28474 0.26878 0.27002 0.33373 0.32255 0.35029 1.0326 0.98146 0.97244 1.0883 1.0545 1.2433 8.1321 30.161 14.526 13.444 32.074 24.415 7 9 9 7 7 9 2 2 3 3 3 4 Leave out requantified Leave out requantified Leave out requantified Leave out requantified Median Leave out requantified 0.9738 0.95046 0.95406 1.4518 1.2227 1.2923 0.70015 0.73812 0.74928 0.97135 0.84181 0.99036 6.6224 25.882 18.674 2.7225 24.92 21.495 7 9 9 7 7 9 2 2 3 3 3 4 Leave out requantified Leave out requantified Leave out requantified Leave out requantified Median Leave out requantified 10.4 7.3 11.4 5.6 7.3 14.2 503420000 324790000 89257000 89380000 53580000 36267000 9356300 7956200 65033000 40197000 13153000 11682000 104800000 67797000 21206000 15794000 79628000 52608000 12689000 14331000 41636000 24362000 7638800 9635100 158750000 103560000 25213000 29982000 58 2016;3427;5523;8183;9524;11777 True;True;True;True;True;True 2137;3618;5827;8687;10098;10099;12498 18680;18681;31221;31222;31223;31224;31225;50594;50595;50596;50597;50598;50599;50600;50601;74236;85884;85885;85886;85887;85888;85889;85890;85891;85892;85893;85894;85895;85896;85897;85898;85899;85900;85901;85902;85903;85904;85905;85906;85907;85908;85909;85910;85911;85912;85913;85914;85915;85916;85917;85918;85919;85920;85921;85922;85923;85924;85925;85926;108785 26350;26351;26352;26353;43814;43815;43816;43817;72849;72850;72851;72852;72853;72854;72855;72856;72857;108002;124969;124970;124971;124972;124973;124974;124975;124976;124977;124978;124979;124980;124981;124982;124983;124984;124985;124986;124987;124988;124989;124990;124991;124992;124993;124994;124995;124996;124997;124998;124999;125000;125001;125002;125003;125004;125005;125006;125007;125008;125009;125010;125011;125012;125013;125014;125015;125016;125017;125018;125019;125020;125021;125022;125023;125024;125025;125026;125027;158442 26353;43817;72849;108002;124970;158442 50;51;52 136;338;362

A0A0G2JST1 A0A0G2JST1 1 1 1 tr|A0A0G2JST1|A0A0G2JST1_RAT Adhesion G protein-coupled receptor G6 OS=Rattus norvegicus OX=10116 GN=Adgrg6 PE=1 SV=1 1 1 1 1 1 0 1 0 1 1 1 0 1 0 1 1 1 0 1 0 1 1 1.4 1.4 1.4 133.22 1193 1193 1 6 0 5.5821 By MS/MS By matching By MS/MS By MS/MS 1.633 3.7786 9.2698 4 1 Median 1.6209 3.146 33.738 4 1 Median 1.347 1.0666 25.429 4 1 Median 1.3363 NaN 1.9596 NaN 1.6498 1.6164 3.6345 NaN 4.3142 NaN 3.9284 3.4981 NaN NaN NaN NaN NaN NaN 1 0 1 0 1 1 0 0 0 0 1 0 Median Median Median Median Median Median 1.7708 NaN 1.4837 NaN 2.561 1.3355 3.5746 NaN 2.7689 NaN 5.2721 2.4597 NaN NaN NaN NaN NaN NaN 1 0 1 0 1 1 0 0 0 0 1 0 Median Median Median Median Median Median 1.1688 NaN 1.5582 NaN 1.5523 1.0716 0.86938 NaN 1.3104 NaN 1.3085 0.82045 NaN NaN NaN NaN NaN NaN 1 0 1 0 1 1 0 0 0 0 1 0 Median Median Median Median Median Median 1.4 0 1.4 0 1.4 1.4 25284000 5996200 8689500 10598000 4814800 1250700 1651200 1912900 0 0 0 0 5139800 978100 1771800 2389900 0 0 0 0 8779000 1970000 2701100 4107900 6550000 1797400 2565400 2187200 59 9478 True 10047 85337;85338;85339;85340;85341;85342 124221;124222;124223;124224;124225 124225

A0A0G2K611;A0A0G2JSU1;A0A0G2KAS7;G3V846;P24942 A0A0G2K611;A0A0G2JSU1;A0A0G2KAS7;G3V846;P24942 2;2;2;2;2 2;2;2;2;2 2;2;2;2;2 Amino acid transporter;Excitatory amino acid transporter 1 Slc1a3 tr|A0A0G2K611|A0A0G2K611_RAT Amino acid transporter OS=Rattus norvegicus OX=10116 GN=Slc1a3 PE=1 SV=1;tr|A0A0G2JSU1|A0A0G2JSU1_RAT Amino acid transporter OS=Rattus norvegicus OX=10116 GN=Slc1a3 PE=1 SV=1;tr|A0A0G2KAS7|A0A0G2KAS7_RAT Amino acid transporter 5 2 2 2 0 0 1 2 2 1 0 0 1 2 2 1 0 0 1 2 2 1 9.3 9.3 9.3 47.15 431 431;497;498;543;543 1.22 7 2 0 5.5292 By MS/MS By MS/MS By MS/MS By MS/MS 0.62223 1.3373 34.74 8 6 Median 0.56617 1.1417 49.289 8 6 Median 1.0928 0.79675 32.969 8 6 Median NaN NaN 1.1117 0.56033 0.50636 0.67023 NaN NaN 2.4251 0.9744 1.3128 1.386 NaN NaN NaN 42.675 21.795 24.237 0 0 1 3 2 2 0 0 0 3 2 1 Median Median Median Median Median Median NaN NaN 1.7604 0.61689 0.48861 0.61488 NaN NaN 3.1551 1.2984 0.90499 1.0739 NaN NaN NaN 25.341 30.192 56.926 0 0 1 3 2 2 0 0 0 3 2 1 Median Median Median Plateau Median Median NaN NaN 1.4605 1.4145 0.96495 0.90118 NaN NaN 1.2533 0.73901 0.69647 0.71515 NaN NaN NaN 29.684 50.236 25.919 0 0 1 3 2 2 0 0 0 3 2 1 Median Median Median Plateau Median Median 0 0 3.9 9.3 9.3 3.9 57001000 28514000 13845000 14642000 0 0 0 0 0 0 0 0 4292600 1286900 1570300 1435500 21888000 11875000 4477900 5534600 18041000 9785000 4234800 4021200 12780000 5566800 3562100 3650700 60 5851;7781 True;True 6174;8262 53186;53187;70561;70562;70563;70564;70565;70566;70567 77791;77792;102767;102768;102769;102770;102771;102772;102773 77792;102772 53;54;55 397;407;431

A0A0H2UHY9;A0A0G2KAY2;A0A0G2JSW6;P09739 A0A0H2UHY9;A0A0G2KAY2;A0A0G2JSW6;P09739 6;6;6;6 6;6;6;6 6;6;6;6 Troponin T, fast skeletal muscle Tnnt3 tr|A0A0H2UHY9|A0A0H2UHY9_RAT Troponin T, fast skeletal muscle OS=Rattus norvegicus OX=10116 GN=Tnnt3 PE=1 SV=1;tr|A0A0G2KAY2|A0A0G2KAY2_RAT Troponin T, fast skeletal muscle OS=Rattus norvegicus OX=10116 GN=Tnnt3 PE=1 SV=1;tr|A0A0G2JSW6|A0A0G2JSW6_RAT Tropo 4 6 6 6 6 1 1 1 1 1 6 1 1 1 1 1 6 1 1 1 1 1 15.6 15.6 15.6 29.741 250 250;253;259;259 8.62 2 9 23 0 127.72 By MS/MS By MS/MS By MS/MS By MS/MS By MS/MS By MS/MS 0.65384 3.4946 212.1 16 9 Median 0.91289 3.1359 293.57 13 6 Median 1.3939 1.0508 155.18 13 6 Median 0.026204 0.83116 0.92489 1.1658 0.70213 0.8059 0.10475 4.0876 4.7181 5.7953 3.6843 3.8827 195.26 18.394 NaN 6.2432 NaN NaN 9 2 1 2 1 1 8 0 0 0 0 1 Median Median Median Median Median Median 0.018768 1.3858 1.583 1.2499 0.87227 1.1233 0.062091 4.8589 5.5035 4.0965 2.7851 3.7166 292.58 26.401 NaN 15.474 NaN NaN 6 2 1 2 1 1 5 0 0 0 0 1 Median Median Median Median Median Median 0.46463 1.7294 2.1155 1.1985 1.8524 1.3939 0.32245 1.3113 1.4178 0.8071 1.3045 1.0508 206.35 3.6737 NaN 15.297 NaN NaN 6 2 1 2 1 1 5 0 0 0 0 1 Median Median Median Median Median Median 15.6 6 6 6 6 6 600450000 569220000 19601000 11623000 575980000 561110000 13338000 1522500 4976500 1296900 1271200 2408400 3829100 1133200 592970 2102900 8826700 2922800 2610300 3293600 2503900 1117300 636860 749720 4336800 1639500 1151600 1545700 61 841;842;5248;5249;6700;6701 True;True;True;True;True;True 886;887;888;5526;5527;7055;7056 7259;7260;7261;7262;7263;7264;7265;7266;7267;7268;7269;7270;7271;7272;7273;7274;7275;7276;7277;7278;7279;7280;7281;47539;47540;47541;47542;47543;47544;47545;47546;60304;60305;60306 10455;10456;10457;10458;10459;10460;10461;10462;10463;10464;10465;10466;10467;10468;10469;10470;10471;10472;10473;10474;10475;10476;10477;10478;10479;10480;10481;10482;10483;10484;10485;10486;10487;10488;68413;68414;68415;68416;68417;68418;68419;68420;68421;68422;68423;68424;68425;68426;68427;68428;68429;68430;68431;68432;68433;68434;68435;68436;68437;68438;68439;68440;68441;88386;88387;88388;88389;88390 10461;10488;68426;68433;88386;88387 56 142

A0A0G2JSY2;D4A129;D3ZL24 A0A0G2JSY2;D4A129;D3ZL24 4;3;3 1;1;1 1;1;1 Cast tr|A0A0G2JSY2|A0A0G2JSY2_RAT Calpastatin OS=Rattus norvegicus OX=10116 GN=Cast PE=1 SV=1;tr|D4A129|D4A129_RAT Calpastatin OS=Rattus norvegicus OX=10116 GN=Cast PE=1 SV=2;tr|D3ZL24|D3ZL24_RAT Calpastatin OS=Rattus norvegicus OX=10116 GN=Cast PE=1 SV=2 3 4 1 1 1 2 3 2 1 2 0 0 1 0 0 0 0 0 1 0 0 0 8.9 4.4 4.4 73.155 675 675;176;652 4 1 1 -2 By matching By matching By MS/MS By matching By matching By matching NaN NaN NaN 0 0 Median NaN NaN NaN 0 0 Median NaN NaN NaN 0 0 Median NaN NaN NaN NaN NaN NaN NaN NaN NaN NaN NaN NaN NaN NaN NaN NaN NaN NaN 0 0 0 0 0 0 0 0 0 0 0 0 Median Median Median Median Median Median NaN NaN NaN NaN NaN NaN NaN NaN NaN NaN NaN NaN NaN NaN NaN NaN NaN NaN 0 0 0 0 0 0 0 0 0 0 0 0 Median Median Median Median Median Median NaN NaN NaN NaN NaN NaN NaN NaN NaN NaN NaN NaN NaN NaN NaN NaN NaN NaN 0 0 0 0 0 0 0 0 0 0 0 0 Median Median Median Median Median Median 2.1 5.6 8.9 5.6 2.1 5.6 860860 860860 0 0 0 0 0 0 0 0 0 0 860860 860860 0 0 0 0 0 0 0 0 0 0 0 0 0 0 + 62 1113;2808;3822;9492 False;True;False;False 1180;2979;4038;10061 9799;26613;34385;34386;34387;34388;34389;85443;85444;85445;85446;85447;85448;85449;85450 14097;37372;48179;48180;48181;48182;48183;124371;124372;124373;124374;124375;124376;124377;124378 14097;37372;48183;124374 57 140

A0A0G2JVK4;A0A0G2JSY3;P42676 A0A0G2JVK4;A0A0G2JSY3;P42676 10;10;10 10;10;10 10;10;10 Neurolysin, mitochondrial Nln tr|A0A0G2JVK4|A0A0G2JVK4_RAT Neurolysin, mitochondrial OS=Rattus norvegicus OX=10116 GN=Nln PE=1 SV=1;tr|A0A0G2JSY3|A0A0G2JSY3_RAT Neurolysin (Metallopeptidase M3 family), isoform CRA_a OS=Rattus norvegicus OX=10116 GN=Nln PE=1 SV=1;sp|P42676|NEUL_RAT Neur 3 10 10 10 6 3 5 8 6 8 6 3 5 8 6 8 6 3 5 8 6 8 15.4 15.4 15.4 77.801 681 681;704;704 4.41 22 15 0 32.291 By MS/MS By MS/MS By MS/MS By MS/MS By MS/MS By MS/MS 0.24797 0.95299 58.232 32 26 Leave out requantified 0.33197 0.82597 26.857 31 25 Leave out requantified 1.2585 0.97642 42.318 31 25 Leave out requantified 0.28859 0.33298 0.31122 0.32822 0.26783 0.24273 1.0396 1.2338 1.3182 0.87408 0.88238 0.84874 28.287 19.5 32.008 21.191 54.11 16.54 6 3 3 6 7 7 4 3 3 5 5 6 Median Median Median Median Median Median 0.3211 0.28987 0.59735 0.33578 0.35986 0.2659 0.69698 0.89729 1.6787 0.60708 0.74009 0.66653 33.353 33.578 71.362 13.487 29.342 41.788 6 3 3 6 7 6 4 3 3 5 5 5 Median Median Plateau Median Median Median 0.88086 0.96501 1.4197 0.93624 1.2585 1.131 0.63927 0.75568 1.0885 0.65161 0.97642 0.8476 39.849 15.109 49.248 15.975 36.669 41.875 6 3 3 6 7 6 4 3 3 5 5 5 Median Median Plateau Median Median Median 9.5 6.5 7.9 13.2 9.8 12.2 207550000 129520000 33159000 44868000 30651000 18802000 5382300 6466500 23432000 13467000 4321400 5643300 28468000 17538000 4146800 6783200 30425000 19608000 5173800 5643200 33699000 20137000 6063100 7498900 60873000 39969000 8071400 12833000 63 351;352;353;1894;7618;8454;9937;10026;10709;12293 True;True;True;True;True;True;True;True;True;True 369;370;371;2004;8079;8969;10538;10629;11353;13040 3344;3345;3346;3347;3348;3349;3350;3351;3352;3353;3354;3355;17254;17255;68693;68694;68695;68696;68697;76314;76315;76316;76317;89572;89573;89574;90458;90459;96729;96730;96731;113758;113759;113760;113761;113762;113763 4708;4709;4710;4711;4712;4713;4714;4715;4716;4717;4718;4719;4720;4721;24338;24339;100094;100095;100096;100097;100098;110807;110808;110809;110810;130220;130221;130222;131440;131441;140600;140601;140602;165380;165381;165382;165383;165384;165385 4712;4719;4721;24339;100098;110807;130222;131441;140602;165385

A0A0G2JSZ1;Q6GQP4;A0A0G2K3Z3;B0BN19 A0A0G2JSZ1;Q6GQP4;A0A0G2K3Z3;B0BN19 2;2;1;1 2;2;1;1 2;2;1;1 Ras-related protein Rab-31 Rab31;Rab22a tr|A0A0G2JSZ1|A0A0G2JSZ1_RAT Ras-related protein Rab-31 OS=Rattus norvegicus OX=10116 GN=Rab31 PE=4 SV=1;sp|Q6GQP4|RAB31_RAT Ras-related protein Rab-31 OS=Rattus norvegicus OX=10116 GN=Rab31 PE=1 SV=2;tr|A0A0G2K3Z3|A0A0G2K3Z3_RAT RAB22A, member RAS oncogen 4 2 2 2 2 2 2 2 2 2 2 2 2 2 2 2 2 2 2 2 2 2 13.8 13.8 13.8 21.499 195 195;194;187;194 9.59 13 12 2 0 12.648 By MS/MS By MS/MS By MS/MS By MS/MS By MS/MS By MS/MS 0.7755 3.3702 5.6975 22 7 Leave out requantified 0.69627 2.0618 1.5993 22 7 Leave out requantified 0.85819 0.59921 14.475 22 7 Leave out requantified 0.80174 0.65457 0.76637 0.76167 0.79693 0.75449 3.2048 2.8274 3.2337 3.5533 3.5885 3.2014 11.111 33.891 27.858 5.9689 17.488 3.8427 2 2 4 6 5 3 1 2 1 0 3 0 Median Median Median Leave out requantified Median Median 0.51 0.45006 0.47558 0.72788 0.71921 0.67104 1.4671 1.5614 1.5606 2.1999 2.104 1.888 4.7638 35.785 38.323 7.713 25.804 22.449 2 2 4 6 5 3 1 2 1 0 3 0 Median Median Median Leave out requantified Median Median 0.67182 0.68757 0.67445 0.88527 0.98707 0.93074 0.43403 0.5151 0.49216 0.61411 0.68508 0.6586 1.3762 1.8942 8.7895 20.27 17.722 6.8428 2 2 4 6 5 3 1 2 1 0 3 0 Median Median Median Leave out requantified Median Plateau 13.8 13.8 13.8 13.8 13.8 13.8 70376000 29509000 21642000 19224000 2188400 1021300 682880 484150 3097200 1327900 1164900 604420 6939100 3034700 2408200 1496200 33407000 13637000 10054000 9716200 15460000 6529600 4741000 4189900 9284000 3959200 2591200 2733600 64 3561;11056 True;True 3760;11726 32352;32353;32354;32355;32356;32357;32358;32359;32360;32361;32362;32363;32364;32365;32366;32367;32368;32369;100658;100659;100660;100661;100662;100663;100664;100665;100666 45340;45341;45342;45343;45344;45345;45346;45347;45348;45349;45350;45351;45352;45353;45354;45355;45356;45357;45358;45359;45360;45361;45362;45363;45364;45365;45366;146270;146271;146272;146273;146274;146275;146276;146277;146278;146279;146280;146281;146282;146283 45345;146281

A0A0G2JSZ5;Q63081 A0A0G2JSZ5;Q63081 12;12 12;12 12;12 Protein disulfide-isomerase A6 Pdia6 tr|A0A0G2JSZ5|A0A0G2JSZ5_RAT Protein disulfide-isomerase A6 OS=Rattus norvegicus OX=10116 GN=Pdia6 PE=1 SV=1;sp|Q63081|PDIA6_RAT Protein disulfide-isomerase A6 OS=Rattus norvegicus OX=10116 GN=Pdia6 PE=1 SV=2 2 12 12 12 8 8 10 7 5 8 8 8 10 7 5 8 8 8 10 7 5 8 29.9 29.9 29.9 48.76 445 445;440 6.05 5 6 6 9 10 66 14 6 6 5 4 3 0 178.74 By MS/MS By MS/MS By MS/MS By MS/MS By MS/MS By MS/MS 0.18115 0.95558 7.3306 110 54 Leave out requantified 0.18255 0.65523 10.792 105 49 Leave out requantified 0.99571 0.69159 9.2085 105 49 Leave out requantified 0.17199 0.17248 0.18544 0.16706 0.18895 0.19648 0.96109 0.95013 0.91081 0.97917 0.96419 0.97099 13.358 12.491 17.233 4.9364 0.42944 12.422 22 18 14 17 19 20 11 10 6 6 14 7 Leave out requantified Leave out requantified Leave out requantified Leave out requantified Leave out requantified Leave out requantified 0.18994 0.22182 0.18068 0.17457 0.16756 0.1744 0.6947 0.84069 0.67972 0.61483 0.55928 0.54303 11.86 12.533 16.98 21.964 14.677 13.685 21 18 12 17 18 19 10 10 4 6 13 6 Leave out requantified Leave out requantified Leave out requantified Leave out requantified Leave out requantified Leave out requantified 1.124 1.2471 0.98306 1.0023 0.86328 0.9153 0.73972 0.85936 0.76123 0.64865 0.53995 0.63704 9.2191 23.525 6.5317 13.494 8.2695 20.299 21 18 12 17 18 19 10 10 4 6 13 6 Leave out requantified Leave out requantified Leave out requantified Leave out requantified Leave out requantified Leave out requantified 18.7 21.6 27.2 18.2 13.7 23.8 1767300000 1308800000 230080000 228430000 174270000 130340000 20770000 23155000 322280000 233610000 43483000 45179000 197340000 143640000 26934000 26762000 425330000 324950000 50351000 50030000 225340000 163450000 30762000 31132000 422770000 312820000 57781000 52175000 65 1880;1881;2306;2497;2499;4901;4902;5587;6000;7591;7592;8119 True;True;True;True;True;True;True;True;True;True;True;True 1989;1990;2460;2657;2659;5159;5160;5897;6330;8050;8051;8620 17144;17145;17146;17147;21820;21821;21822;21823;21824;21825;21826;23602;23603;23604;23605;23606;23607;23608;23609;23610;23611;23612;23613;23614;23615;23616;23617;23618;23619;23620;23621;23622;23623;23624;23625;23626;23627;23628;23629;23630;23631;23632;23633;23634;23635;23636;23637;23638;23639;23640;23641;23642;23643;23644;23645;23646;23647;23648;23649;23650;23651;23652;23653;23654;23655;23656;23657;23658;23659;23660;23661;23662;23663;23664;23665;23666;23667;23668;23669;23670;23671;23672;23673;23674;23675;23676;23677;23680;23681;44617;44618;44619;44620;44621;44622;44623;44624;44625;44626;44627;44628;44629;44630;44631;51130;51131;51132;51133;51134;54483;54484;54485;54486;54487;68336;68337;68338;68339;68340;68341;68342;68343;68344;68345;68346;68347;68348;68349;68350;68351;68352;68353;68354;68355;68356;68357;68358;73643;73644;73645 24168;24169;24170;24171;30598;30599;30600;30601;30602;30603;30604;30605;30606;30607;30608;30609;30610;30611;30612;30613;30614;30615;30616;30617;30618;30619;30620;30621;33057;33058;33059;33060;33061;33062;33063;33064;33065;33066;33067;33068;33069;33070;33071;33072;33073;33074;33075;33076;33077;33078;33079;33080;33081;33082;33083;33084;33085;33086;33087;33088;33089;33090;33091;33092;33093;33094;33095;33096;33097;33098;33099;33100;33101;33102;33103;33104;33105;33106;33107;33108;33109;33110;33111;33112;33113;33114;33115;33116;33117;33118;33119;33120;33121;33122;33123;33124;33125;33126;33127;33128;33129;33130;33131;33132;33133;33134;33135;33136;33137;33138;33139;33140;33141;33142;33143;33144;33145;33146;33147;33148;33149;33150;33151;33152;33153;33154;33155;33156;33157;33158;33159;33160;33163;33164;63950;63951;63952;63953;63954;63955;63956;63957;63958;63959;63960;63961;63962;63963;63964;63965;63966;63967;63968;63969;63970;63971;63972;63973;63974;63975;73761;73762;73763;73764;73765;80220;80221;80222;80223;80224;80225;99472;99473;99474;99475;99476;99477;99478;99479;99480;99481;99482;99483;99484;99485;99486;99487;99488;99489;99490;99491;99492;99493;99494;99495;99496;99497;99498;99499;99500;99501;99502;99503;99504;99505;99506;99507;99508;99509;99510;99511;107179;107180;107181;107182;107183 24168;24171;30609;33102;33163;63971;63975;73763;80220;99484;99503;107180

G3V786;A0A0G2JT64 G3V786;A0A0G2JT64 2;2 2;2 2;2 Akr1b8 tr|G3V786|G3V786_RAT Aldo-keto reductase family 1, member B8 OS=Rattus norvegicus OX=10116 GN=Akr1b8 PE=1 SV=1;tr|A0A0G2JT64|A0A0G2JT64_RAT Aldo-keto reductase family 1, member B8 OS=Rattus norvegicus OX=10116 GN=Akr1b8 PE=1 SV=1 2 2 2 2 1 1 1 1 2 1 1 1 1 1 2 1 1 1 1 1 2 1 7.6 7.6 7.6 36.16 316 316;316 5.47 4 1 1 5 4 0.00055157 3.0684 By MS/MS By MS/MS By MS/MS By MS/MS By MS/MS By MS/MS 0.42543 2.035 37.082 14 0 Median 0.50562 1.598 36.315 14 0 Median 1.1728 0.78388 18.193 14 0 Median 0.40911 0.45811 0.47341 0.36561 0.4185 0.44078 1.1161 1.516 1.6838 1.9842 2.2728 2.3373 NaN 64.604 51.042 50.945 15.722 4.5171 1 2 2 3 4 2 0 0 0 0 0 0 Median Median Median Median Linear Median 0.38289 0.47593 0.4382 0.52133 0.50898 0.56269 0.76673 1.2172 1.1418 1.6427 1.5807 1.8041 NaN 34.607 54.263 44.709 32.653 7.7477 1 2 2 3 4 2 0 0 0 0 0 0 Median Median Median Median Median Median 0.89536 1.0886 0.88447 1.2758 1.1725 1.2097 0.67467 0.83177 0.66768 0.85657 0.74911 0.78672 NaN 31.612 7.1994 5.3913 24.967 10.519 1 2 2 3 4 2 0 0 0 0 0 0 Median Median Median Median Median Median 2.8 2.8 2.8 2.8 7.6 2.8 213140000 113730000 46315000 53103000 4534100 2568500 991050 974570 19371000 10352000 4472000 4547100 29481000 15499000 7078900 6903800 68384000 36389000 13568000 18427000 52466000 28970000 11545000 11951000 38908000 19948000 8660500 10300000 66 1317;2988 True;True 1393;3162 11728;11729;11730;11731;11732;11733;11734;11735;11736;11737;11738;11739;11740;11741;28186 16837;16838;16839;16840;16841;16842;16843;16844;16845;16846;16847;16848;16849;16850;16851;16852;16853;39768 16850;39768

A0A0G2JT68;D3ZGS5 A0A0G2JT68;D3ZGS5 7;7 7;7 7;7 Uaca tr|A0A0G2JT68|A0A0G2JT68_RAT Uveal autoantigen with coiled-coil domains and ankyrin repeats OS=Rattus norvegicus OX=10116 GN=Uaca PE=1 SV=1;tr|D3ZGS5|D3ZGS5_RAT Uveal autoantigen with coiled-coil domains and ankyrin repeats OS=Rattus norvegicus OX=10116 GN 2 7 7 7 3 3 5 3 4 3 3 3 5 3 4 3 3 3 5 3 4 3 5.1 5.1 5.1 159.16 1393 1393;1410 2.5 4 4 16 0 7.1685 By MS/MS By MS/MS By MS/MS By MS/MS By MS/MS By MS/MS 0.789 1.683 45.766 24 3 Leave out requantified 0.68115 1.2598 21.457 24 3 Leave out requantified 0.92542 0.70958 28.485 24 3 Leave out requantified 0.54259 0.69576 0.78908 0.90747 0.80186 0.72464 1.2126 1.2997 1.7914 1.803 1.7805 1.3498 70.132 88.459 36.764 11.614 25.452 29.78 3 4 6 3 4 4 0 0 1 0 0 2 Leave out requantified Leave out requantified Leave out requantified Leave out requantified Leave out requantified Median 0.52313 0.59044 0.51532 0.87823 0.81583 0.88086 0.90162 1.0257 0.96227 1.4748 1.4509 1.6806 15.256 13.621 22.091 7.1889 32.946 18.953 3 4 6 3 4 4 0 0 1 0 0 2 Leave out requantified Leave out requantified Leave out requantified Leave out requantified Leave out requantified Median 0.8345 0.92275 0.7121 1.0124 1.0028 1.126 0.64895 0.76472 0.54833 0.8022 0.78101 0.87012 47.745 46.281 43.252 24.28 26.549 16.644 3 4 6 3 4 4 0 0 1 0 0 2 Leave out requantified Leave out requantified Leave out requantified Leave out requantified Leave out requantified Median 2.2 2.2 3.6 2.3 3.2 2.5 221450000 108010000 53749000 59690000 17877000 9972700 3322400 4581500 30879000 14915000 7702200 8262200 56878000 30741000 13154000 12983000 20570000 8005300 5702200 6862200 55839000 28524000 13790000 13525000 39407000 15853000 10078000 13475000 67 1246;5320;5965;6051;6789;8315;9824 True;True;True;True;True;True;True 1318;5603;6293;6385;7146;8825;10418 11204;11205;11206;48359;54225;54226;54872;54873;54874;54875;54876;54877;54878;54879;61035;61036;61037;61038;75215;75216;88472;88473;88474;88475 16096;16097;16098;16099;16100;69636;79806;80718;80719;80720;80721;80722;80723;80724;80725;80726;80727;80728;89331;109367;109368;128635;128636;128637;128638;128639;128640 16100;69636;79806;80722;89331;109367;128639 58 622

A0A0G2JT88;Q4V884 A0A0G2JT88;Q4V884 2;2 2;2 2;2 Cdc16 tr|A0A0G2JT88|A0A0G2JT88_RAT Cell division cycle 16 OS=Rattus norvegicus OX=10116 GN=Cdc16 PE=1 SV=1;tr|Q4V884|Q4V884_RAT CDC16 cell division cycle 16 homolog (S. cerevisiae) OS=Rattus norvegicus OX=10116 GN=Cdc16 PE=1 SV=1 2 2 2 2 1 1 1 1 2 0 1 1 1 1 2 0 1 1 1 1 2 0 4 4 4 69.103 602 602;620 4.67 2 4 0.0094509 1.6051 By MS/MS By MS/MS By MS/MS By MS/MS By MS/MS 0.32674 1.0645 96.12 6 3 Median 0.51414 1.1408 95.416 6 3 Plateau 1.4551 1.0263 25.019 6 3 Median 0.34298 0.26354 0.31127 0.45196 0.10363 NaN 1.1174 0.80254 1.014 1.626 0.44319 NaN NaN NaN NaN NaN 180.35 NaN 1 1 1 1 2 0 0 0 1 0 2 0 Median Median Median Median Median Median 0.51026 0.45625 0.38226 0.57937 0.12077 NaN 1.1354 1.1462 0.93286 1.4793 0.37478 NaN NaN NaN NaN NaN 165.8 NaN 1 1 1 1 2 0 0 0 1 0 2 0 Median Median Median Median Median Median 1.5562 1.7014 1.2281 1.7821 1.1654 NaN 1.117 1.3212 0.93052 1.3459 0.80952 NaN NaN NaN NaN NaN 21.576 NaN 1 1 1 1 2 0 0 0 1 0 2 0 Median Median Median Median Median Median 1.3 1.3 1.3 1.3 4 0 50529000 38986000 4048800 7494400 3133100 1701700 564690 866700 4259600 2277600 605410 1376500 4896600 2929400 823030 1144100 5386100 3059200 699800 1627100 32854000 29018000 1355900 2479900 0 0 0 0 68 7196;8300 True;True 7603;8810 64576;64577;64578;64579;64580;75088 94092;94093;94094;94095;94096;109207 94095;109207 59 1

A0A0G2JT93;Q9WU82 A0A0G2JT93;Q9WU82 3;3 3;3 3;3 Catenin beta-1 Ctnnb1 tr|A0A0G2JT93|A0A0G2JT93_RAT Catenin (Cadherin associated protein), beta 1, isoform CRA_a OS=Rattus norvegicus OX=10116 GN=Ctnnb1 PE=1 SV=1;sp|Q9WU82|CTNB1_RAT Catenin beta-1 OS=Rattus norvegicus OX=10116 GN=Ctnnb1 PE=1 SV=1 2 3 3 3 2 3 2 2 2 2 2 3 2 2 2 2 2 3 2 2 2 2 7 7 7 85.47 781 781;781 2.87 16 2 3 20 4 0 32.874 By MS/MS By MS/MS By MS/MS By MS/MS By MS/MS By MS/MS 1.0312 3.6362 7.8468 42 8 Leave out requantified 1.4014 3.7164 3.8559 42 8 Leave out requantified 1.3278 0.95965 12.092 42 8 Leave out requantified 0.97918 1.1748 1.1936 0.83106 1.1218 1.0322 4.0544 4.1913 3.7252 2.5417 4.3661 3.3988 0.5892 27.968 31.524 34.065 18.022 16.831 7 8 7 6 7 7 0 3 1 1 2 1 Leave out requantified Leave out requantified Leave out requantified Leave out requantified Leave out requantified Leave out requantified 1.4434 1.3761 1.3418 1.3641 1.4562 1.277 3.6365 4.1088 3.4358 3.3088 3.7557 3.2405 12.052 9.1198 4.0872 8.7059 8.505 8.7103 7 8 7 6 7 7 0 3 1 1 2 1 Leave out requantified Leave out requantified Leave out requantified Leave out requantified Leave out requantified Leave out requantified 1.4554 1.1706 1.0454 1.5667 1.2901 1.2359 1.017 0.91967 0.82979 1.0776 0.92821 0.93474 5.3595 12.493 34.158 1.5491 7.3815 24.006 7 8 7 6 7 7 0 3 1 1 2 1 Leave out requantified Leave out requantified Leave out requantified Leave out requantified Leave out requantified Leave out requantified 3.8 7 3.8 3.8 3.8 3.8 595210000 181050000 178290000 235870000 76825000 22442000 23475000 30909000 93216000 26171000 27712000 39333000 73209000 20617000 23916000 28677000 108210000 39096000 25918000 43194000 119580000 34500000 38131000 46950000 124170000 38226000 39134000 46809000 69 1350;4773;6395 True;True;True 1428;5025;6741 11969;11970;11971;11972;11973;11974;11975;11976;11977;11978;11979;11980;11981;11982;11983;11984;11985;11986;11987;11988;11989;11990;11991;43368;57828;57829;57830;57831;57832;57833;57834;57835;57836;57837;57838;57839;57840;57841;57842;57843;57844;57845;57846;57847;57848 17148;17149;17150;17151;17152;17153;17154;17155;17156;17157;17158;17159;17160;17161;17162;17163;17164;17165;17166;17167;17168;17169;17170;17171;17172;17173;17174;17175;17176;17177;17178;17179;61985;61986;84817;84818;84819;84820;84821;84822;84823;84824;84825;84826;84827;84828;84829;84830;84831;84832;84833;84834;84835;84836;84837;84838;84839;84840;84841;84842;84843;84844;84845;84846;84847;84848;84849;84850;84851;84852;84853;84854;84855;84856 17148;61986;84841 60;61;62 8;12;14

A0A0G2JTA0 A0A0G2JTA0 1 1 1 tr|A0A0G2JTA0|A0A0G2JTA0_RAT FAS-associated factor 2 OS=Rattus norvegicus OX=10116 GN=Faf2 PE=1 SV=1 1 1 1 1 1 1 1 1 1 1 1 1 1 1 1 1 1 1 1 1 1 1 3.4 3.4 3.4 52.46 445 445 4.82 2 3 6 0 120.77 By MS/MS By MS/MS By MS/MS By MS/MS By MS/MS By MS/MS 0.39448 1.9336 46.52 10 2 Median 0.42296 1.5427 24.612 9 1 Median 1.1628 0.77254 18.368 9 1 Median 0.39099 0.41218 0.44639 0.30589 0.34884 0.41942 2.0427 2.0506 1.4351 1.0655 1.9567 2.1313 11.604 20.822 40.475 101.35 NaN NaN 2 2 2 2 1 1 0 0 1 1 0 0 Median Median Median Median Median Median 0.4096 0.38971 0.42296 0.41435 0.44164 0.51126 1.4509 1.3847 1.5003 1.101 1.5948 1.7805 18.024 16.378 NaN 47.7 NaN NaN 2 2 1 2 1 1 0 0 0 1 0 0 Median Median Median Median Median Median 1.1793 0.92112 1.1628 1.3681 1.2675 1.1388 0.80987 0.67983 0.89414 0.96447 0.77254 0.88072 13.304 3.761 NaN 33.384 NaN NaN 2 2 1 2 1 1 0 0 0 1 0 0 Median Median Median Median Median Median 3.4 3.4 3.4 3.4 3.4 3.4 84145000 48035000 17518000 18592000 10374000 5509400 2313100 2551000 12334000 6854400 2957600 2522300 11796000 6728900 2836400 2230800 20481000 13381000 3247900 3851800 13504000 7387800 2969700 3146200 15657000 8173700 3193600 4289600 70 126 True 131 971;972;973;974;975;976;977;978;979;980;981 1308;1309;1310;1311;1312;1313;1314;1315;1316;1317;1318;1319;1320;1321;1322;1323;1324;1325;1326 1320

A0A0G2JTA5;A0A0G2JZM5 A0A0G2JTA5;A0A0G2JZM5 1;1 1;1 1;1 tr|A0A0G2JTA5|A0A0G2JTA5_RAT Euchromatic histone lysine methyltransferase 2 OS=Rattus norvegicus OX=10116 GN=Ehmt2 PE=1 SV=1;tr|A0A0G2JZM5|A0A0G2JZM5_RAT Euchromatic histone lysine methyltransferase 2 OS=Rattus norvegicus OX=10116 GN=Ehmt2 PE=1 SV=1 2 1 1 1 0 0 1 0 1 0 0 0 1 0 1 0 0 0 1 0 1 0 2.3 2.3 2.3 128.48 1172 1172;1206 3 2 0.00054025 2.9218 By MS/MS By MS/MS NaN NaN NaN 0 0 Median NaN NaN NaN 0 0 Median NaN NaN NaN 0 0 Median NaN NaN NaN NaN NaN NaN NaN NaN NaN NaN NaN NaN NaN NaN NaN NaN NaN NaN 0 0 0 0 0 0 0 0 0 0 0 0 Median Median Median Median Median Median NaN NaN NaN NaN NaN NaN NaN NaN NaN NaN NaN NaN NaN NaN NaN NaN NaN NaN 0 0 0 0 0 0 0 0 0 0 0 0 Median Median Median Median Median Median NaN NaN NaN NaN NaN NaN NaN NaN NaN NaN NaN NaN NaN NaN NaN NaN NaN NaN 0 0 0 0 0 0 0 0 0 0 0 0 Median Median Median Median Median Median 0 0 2.3 0 2.3 0 1882500 0 823510 1058900 0 0 0 0 0 0 0 0 1058900 0 0 1058900 0 0 0 0 823510 0 823510 0 0 0 0 0 71 6 True 6 52;53 69;70 69 63 21

A0A0G2JTA7;D4AAY3;A0A0G2JTS0;A0A0G2JTF2;Q6P730 A0A0G2JTA7;D4AAY3;A0A0G2JTS0;A0A0G2JTF2;Q6P730 2;1;1;1;1 2;1;1;1;1 2;1;1;1;1 Disabled homolog 2-interacting protein Rasal2;Dab2ip tr|A0A0G2JTA7|A0A0G2JTA7_RAT RAS protein activator-like 2 OS=Rattus norvegicus OX=10116 GN=Rasal2 PE=1 SV=1;tr|D4AAY3|D4AAY3_RAT RAS protein activator like 2 (Predicted), isoform CRA_a OS=Rattus norvegicus OX=10116 GN=Rasal2 PE=1 SV=1;tr|A0A0G2JTS0|A0A0G2J 5 2 2 2 1 1 1 1 1 1 1 1 1 1 1 1 1 1 1 1 1 1 2.3 2.3 2.3 143.67 1279 1279;937;1064;1161;996 3 8 0.00296 2.2852 By MS/MS By MS/MS By MS/MS By MS/MS By MS/MS By MS/MS 1.4381 2.6826 54.675 3 3 Plateau 1.4184 2.2192 30.164 3 3 Median 1.0914 0.86179 23.063 3 3 Median 0.62841 NaN NaN 1.7163 NaN 1.4381 1.0317 NaN NaN 2.6363 NaN 2.6826 NaN NaN NaN NaN NaN NaN 1 0 0 1 0 1 1 0 0 1 0 1 Median Median Median Median Median Median 1.1133 NaN NaN 1.8731 NaN 1.4184 1.4111 NaN NaN 2.4992 NaN 2.2192 NaN NaN NaN NaN NaN NaN 1 0 0 1 0 1 1 0 0 1 0 1 Median Median Median Median Median Median 1.7716 NaN NaN 1.0914 NaN 0.98629 1.2044 NaN NaN 0.86179 NaN 0.77396 NaN NaN NaN NaN NaN NaN 1 0 0 1 0 1 1 0 0 1 0 1 Median Median Median Median Median Median 1.2 1.2 1.2 1.2 1.2 1.1 11149000 2158700 3930800 5059100 1413300 375010 272200 766140 2286800 0 861480 1425300 0 0 0 0 1761000 390880 437950 932130 0 0 0 0 5687500 1392800 2359200 1935500 72 869;9659 True;True 915;10247 7561;7562;7563;7564;7565;7566;7567;87215 10867;10868;10869;10870;10871;10872;10873;126913 10867;126913

A0A0G2JTD1 A0A0G2JTD1 1 1 1 tr|A0A0G2JTD1|A0A0G2JTD1_RAT Regulation of nuclear pre-mRNA domain-containing 2 OS=Rattus norvegicus OX=10116 GN=Rprd2 PE=1 SV=1 1 1 1 1 0 0 0 0 1 1 0 0 0 0 1 1 0 0 0 0 1 1 1.8 1.8 1.8 154.31 1451 1451 3 2 0 3.2562 By MS/MS By MS/MS 1.3074 2.5049 1.3697 2 2 Median 2.0716 3.2298 34.07 2 2 Median 1.5845 1.2576 28.839 2 2 Median NaN NaN NaN NaN 1.2762 1.3394 NaN NaN NaN NaN 2.5293 2.4807 NaN NaN NaN NaN NaN NaN 0 0 0 0 1 1 0 0 0 0 1 1 Median Median Median Median Median Median NaN NaN NaN NaN 1.6304 2.6323 NaN NaN NaN NaN 2.5384 4.1096 NaN NaN NaN NaN NaN NaN 0 0 0 0 1 1 0 0 0 0 1 1 Median Median Median Median Median Median NaN NaN NaN NaN 1.2776 1.9652 NaN NaN NaN NaN 1.0256 1.5421 NaN NaN NaN NaN NaN NaN 0 0 0 0 1 1 0 0 0 0 1 1 Median Median Median Median Median Median 0 0 0 0 1.8 1.8 10831000 2139600 3640900 5050300 0 0 0 0 0 0 0 0 0 0 0 0 0 0 0 0 5485700 845220 1036300 3604100 5345200 1294400 2604600 1446200 73 7863 True 8349 71570;71571 104209;104210 104209

A0A0G2JTD7;F1LNR1;F1LNR0;F1LNQ9;A0A0G2JUI5;A0A1B0GWM1;A0A1B0GWS3 A0A0G2JTD7;F1LNR1;F1LNR0;F1LNQ9;A0A0G2JUI5;A0A1B0GWM1 4;4;4;4;4;3;1 4;4;4;4;4;3;1 4;4;4;4;4;3;1 Clasp1 tr|A0A0G2JTD7|A0A0G2JTD7_RAT Cytoplasmic linker-associated protein 1 OS=Rattus norvegicus OX=10116 GN=Clasp1 PE=1 SV=1;tr|F1LNR1|F1LNR1_RAT Cytoplasmic linker-associated protein 1 OS=Rattus norvegicus OX=10116 GN=Clasp1 PE=1 SV=3;tr|F1LNR0|F1LNR0_RAT Cytop 7 4 4 4 1 2 2 3 3 3 1 2 2 3 3 3 1 2 2 3 3 3 5.1 5.1 5.1 161 1458 1458;1467;1525;1535;1551;345;815 2.83 1 3 12 2 0 29.621 By MS/MS By MS/MS By MS/MS By MS/MS By MS/MS By MS/MS 1.0222 1.8918 10.884 15 4 Leave out requantified 1.0271 1.7262 16.905 15 4 Leave out requantified 1.0422 0.82173 27.304 15 4 Leave out requantified 0.91774 0.90914 0.72975 0.855 1.0222 1.022 1.5881 1.9954 1.426 1.6662 2.0611 1.8984 24.254 20.046 NaN 49.119 13.834 10.599 2 3 1 3 3 3 0 1 1 1 0 1 Median Plateau Median Median Leave out requantified Median 1.2755 1.2683 1.0309 0.95802 0.78734 0.94208 1.8019 2.3348 1.6524 1.6168 1.419 1.8154 13.198 37.664 NaN 230.99 14.918 19.934 2 3 1 3 3 3 0 1 1 1 0 1 Median Plateau Median Median Leave out requantified Median 1.3833 1.2735 1.4127 1.0237 0.67871 1.0841 1.0298 1.0561 1.1768 0.80872 0.51951 0.8377 5.1781 33.523 NaN 170.96 44.011 13.189 2 3 1 3 3 3 0 1 1 1 0 1 Median Median Median Plateau Leave out requantified Median 1.5 2.4 2.4 3.4 4.1 4.1 91949000 33816000 29775000 28358000 5806000 1815000 1876800 2114200 11896000 3751800 3937200 4206900 4934200 1363100 1566800 2004300 13721000 6594000 4248200 2878500 26901000 9930000 8994900 7976500 28691000 10363000 9150800 9177900 74 2755;6377;7025;7465 True;True;True;True 2925;6723;7406;7920 26135;26136;26137;26138;26139;26140;57660;57661;63280;67210;67211;67212;67213;67214;67215;67216;67217;67218 36621;36622;36623;36624;36625;36626;84551;84552;92340;97812;97813;97814;97815;97816;97817;97818;97819;97820;97821;97822;97823;97824;97825;97826 36623;84551;92340;97818

Q499R8;A0A0G2JTG7;Q8VHV7;G3V9Q3 Q499R8;A0A0G2JTG7;Q8VHV7 3;3;3;1 3;3;3;1 3;3;3;1 Heterogeneous nuclear ribonucleoprotein H;Heterogeneous nuclear ribonucleoprotein H, N-terminally processed Hnrnph1 tr|Q499R8|Q499R8_RAT Heterogeneous nuclear ribonucleoprotein H OS=Rattus norvegicus OX=10116 GN=Hnrnph1 PE=1 SV=1;tr|A0A0G2JTG7|A0A0G2JTG7_RAT Heterogeneous nuclear ribonucleoprotein H OS=Rattus norvegicus OX=10116 GN=Hnrnph1 PE=1 SV=1;sp|Q8VHV7|HNRH1_RAT 4 3 3 3 3 3 1 3 3 2 3 3 1 3 3 2 3 3 1 3 3 2 13.6 13.6 13.6 20.58 184 184;449;449;372 5.84 6 17 2 0 23.975 By MS/MS By MS/MS By MS/MS By MS/MS By MS/MS By MS/MS 0.17775 0.83173 11.681 21 9 Leave out requantified 0.33051 1.1627 3.5833 21 9 Leave out requantified 1.9976 1.4764 12.791 21 9 Leave out requantified 0.16686 0.19444 0.18037 0.19504 0.18134 0.16084 0.81434 1.0191 0.84949 0.96699 0.96087 0.72101 17.619 28.701 NaN 19.589 4.3344 19.603 5 8 1 3 2 2 2 6 0 0 0 1 Leave out requantified Median Median Leave out requantified Median Median 0.33944 0.34893 0.37337 0.32182 0.36012 0.49257 1.1627 1.2263 1.2531 0.99454 1.2347 1.5407 2.8291 17.621 NaN 28.047 6.3761 51.531 5 8 1 3 2 2 2 6 0 0 0 1 Leave out requantified Median Median Leave out requantified Median Median 2.0933 1.8049 1.9544 1.6721 1.939 2.925 1.4836 1.3694 1.4913 1.0741 1.2482 2.3046 13.052 19.178 NaN 14.31 28.941 77.154 5 8 1 3 2 2 2 6 0 0 0 1 Leave out requantified Median Median Leave out requantified Median Median 13.6 13.6 6 13.6 13.6 7.6 117830000 76922000 13030000 27881000 17115000 9795500 1821800 5497400 33639000 21964000 3514100 8160600 7010000 4473400 1021300 1515300 23388000 15370000 3165500 4852700 16488000 11576000 1770100 3141900 20192000 13742000 1736800 4713000 75 7134;7174;9565 True;True;True 7533;7577;10145;10146 64086;64087;64088;64089;64090;64091;64092;64093;64363;64364;64365;64366;64367;64368;64369;64370;64371;64372;64373;86206;86207;86208;86209;86210;86211 93406;93407;93408;93409;93410;93411;93412;93413;93414;93758;93759;93760;93761;93762;93763;93764;93765;93766;93767;93768;93769;93770;93771;93772;93773;125399;125400;125401;125402;125403;125404;125405;125406 93411;93763;125403 64;65;66 1;2;93

A0A0G2JTH1;A0A0U1RRZ3 A0A0G2JTH1;A0A0U1RRZ3 2;1 2;1 2;1 tr|A0A0G2JTH1|A0A0G2JTH1_RAT Uncharacterized protein OS=Rattus norvegicus OX=10116 GN=Nkrfl PE=4 SV=1;tr|A0A0U1RRZ3|A0A0U1RRZ3_RAT NFKB-repressing factor (Fragment) OS=Rattus norvegicus OX=10116 GN=Nkrf PE=4 SV=2 2 2 2 2 2 0 2 2 2 2 2 0 2 2 2 2 2 0 2 2 2 2 4.5 4.5 4.5 64.357 573 573;385 4 12 0.00052882 2.7839 By MS/MS By MS/MS By MS/MS By MS/MS By MS/MS 0.66759 2 65.972 9 9 Median 0.64099 1.2417 26.07 9 9 Median 0.81668 0.60284 59.194 9 9 Median 0.4938 NaN 0.49741 1.6834 1.0404 0.72049 1.3692 NaN 1.0078 3.9188 2.8166 1.4998 53.588 NaN 11.43 NaN 34.156 108.96 2 0 2 1 2 2 2 0 2 1 2 2 Median Median Median Median Median Median 0.46908 NaN 0.70342 0.65499 0.6471 0.61284 0.93619 NaN 1.2205 1.2417 1.406 1.0991 28.726 NaN 39.447 NaN 6.3963 34.64 2 0 2 1 2 2 2 0 2 1 2 2 Median Median Median Median Median Median 0.94994 NaN 1.4142 0.3891 0.62195 0.85059 0.7321 NaN 1.1578 0.29838 0.46968 0.73176 27.473 NaN 62.312 NaN 23.45 86.521 2 0 2 1 2 2 2 0 2 1 2 2 Median Median Median Median Median Median 4.5 0 4.5 4.5 4.5 4.5 32553000 12802000 11947000 7804200 2870400 1276800 687210 906380 0 0 0 0 4282100 2079600 969720 1232800 6000100 2775500 2130300 1094300 10270000 3773900 4016500 2479200 9130700 2896000 4143000 2091600 76 1784;7644 True;True 1886;8107 16058;16059;16060;16061;16062;16063;68998;68999;69000;69001;69002;69003 22802;22803;22804;22805;22806;22807;100563;100564;100565;100566;100567;100568 22802;100568 67 190

A0A0G2JTH4;P97829 A0A0G2JTH4;P97829 3;3 3;3 3;3 Leukocyte surface antigen CD47 Cd47 tr|A0A0G2JTH4|A0A0G2JTH4_RAT Leukocyte surface antigen CD47 OS=Rattus norvegicus OX=10116 GN=Cd47 PE=1 SV=1;sp|P97829|CD47_RAT Leukocyte surface antigen CD47 OS=Rattus norvegicus OX=10116 GN=Cd47 PE=1 SV=1 2 3 3 3 3 3 3 3 3 3 3 3 3 3 3 3 3 3 3 3 3 3 9.2 9.2 9.2 38.927 359 359;303 2.07 22 13 14 6 0 29.728 By MS/MS By MS/MS By MS/MS By MS/MS By MS/MS By MS/MS 0.44003 1.3805 22.429 54 17 Leave out requantified 0.49663 1.2847 7.3884 54 17 Leave out requantified 1.1188 0.82502 14.268 54 17 Leave out requantified 0.47808 0.54033 0.47745 0.42574 0.44868 0.42965 1.6167 1.6862 1.4464 1.1369 2.1129 1.2311 47.355 15.041 19.846 21.685 43.356 4.035 8 7 8 11 10 10 3 1 0 4 5 4 Leave out requantified Leave out requantified Leave out requantified Leave out requantified Leave out requantified Leave out requantified 0.54087 0.55197 0.51427 0.46039 0.50117 0.48114 1.2847 1.2177 1.3041 1.323 1.3191 1.1179 21.683 0.050484 11.876 15.475 15.065 12.133 8 7 8 11 10 10 3 1 0 4 5 4 Leave out requantified Leave out requantified Leave out requantified Leave out requantified Leave out requantified Leave out requantified 1.0887 1.1338 1.0763 1.095 1.1521 1.1355 0.81608 0.92506 0.82654 0.75314 0.85291 0.87898 11.766 2.5154 16.942 9.1297 29.881 5.7931 8 7 8 11 10 10 3 1 0 4 5 4 Leave out requantified Leave out requantified Leave out requantified Leave out requantified Leave out requantified Leave out requantified 9.2 9.2 9.2 9.2 9.2 9.2 1100000000 432540000 199320000 468190000 77077000 38618000 18329000 20130000 98304000 47455000 25329000 25520000 135560000 66640000 33328000 35589000 421060000 106250000 48383000 266430000 191660000 96578000 41214000 53865000 176390000 77003000 32733000 66655000 77 5409;10068;11397 True;True;True 5706;10671;12092;12093 49565;49566;49567;49568;49569;49570;49571;49572;49573;49574;49575;49576;49577;49578;49579;49580;49581;49582;49583;49584;49585;49586;49587;90711;90712;90713;90714;90715;90716;90717;90718;90719;90720;90721;90722;90723;90724;90725;90726;104372;104373;104374;104375;104376;104377;104378;104379;104380;104381;104382;104383;104384;104385;104386;104387 71431;71432;71433;71434;71435;71436;71437;71438;71439;71440;71441;71442;71443;71444;71445;71446;71447;71448;71449;71450;71451;71452;71453;71454;71455;71456;71457;71458;71459;71460;71461;71462;71463;71464;71465;71466;71467;71468;71469;71470;131752;131753;131754;131755;131756;131757;131758;131759;131760;131761;131762;131763;131764;131765;131766;131767;131768;131769;131770;131771;131772;131773;131774;151844;151845;151846;151847;151848;151849;151850;151851;151852;151853;151854;151855;151856;151857;151858;151859;151860;151861;151862;151863;151864;151865;151866;151867 71432;131763;151844 68 54

A0A0G2JTH7;F1M7B8 A0A0G2JTH7;F1M7B8 2;2 2;2 2;2 Ube3a tr|A0A0G2JTH7|A0A0G2JTH7_RAT Ubiquitin protein ligase E3A OS=Rattus norvegicus OX=10116 GN=Ube3a PE=1 SV=1;tr|F1M7B8|F1M7B8_RAT Ubiquitin protein ligase E3A OS=Rattus norvegicus OX=10116 GN=Ube3a PE=1 SV=2 2 2 2 2 0 0 1 0 1 2 0 0 1 0 1 2 0 0 1 0 1 2 2.9 2.9 2.9 95.637 832 832;868 4 4 0.0077697 1.736 By MS/MS By MS/MS By MS/MS 0.66837 1.3382 28.028 4 2 Median 0.72756 1.2796 24.668 4 2 Median 0.96112 0.84686 16.964 4 2 Median NaN NaN 0.91538 NaN 0.48678 0.66837 NaN NaN 1.9252 NaN 1.1533 1.2722 NaN NaN NaN NaN NaN 28.177 0 0 1 0 1 2 0 0 1 0 0 1 Median Median Median Median Median Median NaN NaN 0.76564 NaN 0.81878 0.63729 NaN NaN 1.3512 NaN 1.679 1.0602 NaN NaN NaN NaN NaN 18.89 0 0 1 0 1 2 0 0 1 0 0 1 Median Median Median Median Median Median NaN NaN 0.83642 NaN 1.2922 0.96112 NaN NaN 0.68522 NaN 0.99253 0.84686 NaN NaN NaN NaN NaN 13.034 0 0 1 0 1 2 0 0 1 0 0 1 Median Median Median Median Median Median 0 0 1.6 0 1.6 2.9 11848000 4731500 3319800 3797000 0 0 0 0 0 0 0 0 2411500 782270 652110 977110 0 0 0 0 4070000 1698700 1080700 1290600 5366800 2250500 1586900 1529300 78 2752;4020 True;True 2922;4246 26114;26115;26116;36264 36586;36587;51174 36587;51174

D3ZI99;A0A0G2JTI7 D3ZI99;A0A0G2JTI7 2;2 2;2 2;2 Prpf3 tr|D3ZI99|D3ZI99_RAT PRP3 pre-mRNA processing factor 3 homolog (Yeast) (Predicted), isoform CRA_a OS=Rattus norvegicus OX=10116 GN=Prpf3 PE=1 SV=1;tr|A0A0G2JTI7|A0A0G2JTI7_RAT Pre-mRNA-processing factor 3 OS=Rattus norvegicus OX=10116 GN=Prpf3 PE=1 SV=1 2 2 2 2 1 0 0 1 0 0 1 0 0 1 0 0 1 0 0 1 0 0 4.6 4.6 4.6 72.195 637 637;683 4 2 0 9.453 By MS/MS By MS/MS 0.38943 1.3916 NaN 1 0 Median 0.8507 1.8603 NaN 1 0 Median 2.2524 1.4184 NaN 1 0 Median NaN NaN NaN 0.38943 NaN NaN NaN NaN NaN 1.3916 NaN NaN NaN NaN NaN NaN NaN NaN 0 0 0 1 0 0 0 0 0 0 0 0 Median Median Median Median Median Median NaN NaN NaN 0.8507 NaN NaN NaN NaN NaN 1.8603 NaN NaN NaN NaN NaN NaN NaN NaN 0 0 0 1 0 0 0 0 0 0 0 0 Median Median Median Median Median Median NaN NaN NaN 2.2524 NaN NaN NaN NaN NaN 1.4184 NaN NaN NaN NaN NaN NaN NaN NaN 0 0 0 1 0 0 0 0 0 0 0 0 Median Median Median Median Median Median 1.9 0 0 2.7 0 0 6877900 3534700 1248200 2095000 875090 875090 0 0 0 0 0 0 0 0 0 0 6002800 2659600 1248200 2095000 0 0 0 0 0 0 0 0 79 2481;3456 True;True 2641;3650 23511;31500 32932;44181 32932;44181

A0A0G2JTL5;P52873 A0A0G2JTL5;P52873 4;4 4;4 4;4 Pyruvate carboxylase, mitochondrial Pc tr|A0A0G2JTL5|A0A0G2JTL5_RAT Pyruvate carboxylase, mitochondrial OS=Rattus norvegicus OX=10116 GN=Pc PE=1 SV=1;sp|P52873|PYC_RAT Pyruvate carboxylase, mitochondrial OS=Rattus norvegicus OX=10116 GN=Pc PE=1 SV=2 2 4 4 4 1 1 3 2 1 1 1 1 3 2 1 1 1 1 3 2 1 1 5.3 5.3 5.3 140 1277 1277;1178 3 9 0 5.5769 By MS/MS By MS/MS By MS/MS By MS/MS By MS/MS By MS/MS 0.3646 0.69284 25.303 9 7 Median 0.30328 0.45413 23.455 7 5 Median 0.8529 0.66951 25.258 7 5 Median 0.34872 0.38787 0.46394 0.29519 0.32596 0.31218 0.76884 0.71577 0.87496 0.49054 0.62769 0.59092 NaN NaN 16.686 27.391 NaN NaN 1 1 3 2 1 1 0 1 3 2 0 1 Median Median Median Median Median Median 0.20254 NaN 0.27951 0.30328 0.37123 0.33839 0.34477 NaN 0.43669 0.45413 0.64574 0.53647 NaN NaN 23.533 NaN NaN NaN 1 0 3 1 1 1 0 0 3 1 0 1 Median Median Median Median Median Median 1.1351 NaN 0.64134 1.2838 0.8529 1.0839 0.79032 NaN 0.53717 1.0161 0.66951 0.84952 NaN NaN 10.493 NaN NaN NaN 1 0 3 1 1 1 0 0 3 1 0 1 Median Median Median Median Median Median 1.3 1.3 4.2 2.3 1.3 1.3 38458000 25084000 7798200 5575500 3491400 2424900 692980 373500 2123200 1206700 616290 300250 11002000 6642000 2465100 1895000 6412500 4480100 979280 953080 8785000 5574600 1961000 1249400 6643900 4756100 1083500 804220 80 1561;2131;8424;11211 True;True;True;True 1650;2256;8938;11888 13877;19901;76060;76061;76062;76063;76064;76065;102397 19819;28127;110484;110485;110486;110487;110488;110489;148748 19819;28127;110488;148748 69 999

A0A0G2JZZ7;A0A0G2JTR4 A0A0G2JZZ7;A0A0G2JTR4 1;1 1;1 1;1 tr|A0A0G2JZZ7|A0A0G2JZZ7_RAT ABR, RhoGEF and GTPase-activating protein OS=Rattus norvegicus OX=10116 GN=Abr PE=1 SV=1;tr|A0A0G2JTR4|A0A0G2JTR4_RAT ABR, RhoGEF and GTPase-activating protein OS=Rattus norvegicus OX=10116 GN=Abr PE=1 SV=1 2 1 1 1 1 0 0 1 1 0 1 0 0 1 1 0 1 0 0 1 1 0 2.4 2.4 2.4 93.77 822 822;842 5.75 1 1 1 1 1 -2 By MS/MS By MS/MS By MS/MS NaN NaN NaN 0 0 Median NaN NaN NaN 0 0 Median NaN NaN NaN 0 0 Median NaN NaN NaN NaN NaN NaN NaN NaN NaN NaN NaN NaN NaN NaN NaN NaN NaN NaN 0 0 0 0 0 0 0 0 0 0 0 0 Median Median Median Median Median Median NaN NaN NaN NaN NaN NaN NaN NaN NaN NaN NaN NaN NaN NaN NaN NaN NaN NaN 0 0 0 0 0 0 0 0 0 0 0 0 Median Median Median Median Median Median NaN NaN NaN NaN NaN NaN NaN NaN NaN NaN NaN NaN NaN NaN NaN NaN NaN NaN 0 0 0 0 0 0 0 0 0 0 0 0 Median Median Median Median Median Median 2.4 0 0 2.4 2.4 0 56921000 56921000 0 0 9421700 9421700 0 0 0 0 0 0 0 0 0 0 7981400 7981400 0 0 39518000 39518000 0 0 0 0 0 0 + 81 11094 True 11765 101059;101060;101061;101062 146817;146818;146819;146820 146817 70 587

Q66WT9;A0A0G2JUQ5;Q498N4;A0A0G2JTT2;O55012;Q66SY1 Q66WT9;A0A0G2JUQ5;Q498N4;A0A0G2JTT2;O55012;Q66SY1 3;3;3;3;3;2 3;3;3;3;3;2 3;3;3;3;3;2 Phosphatidylinositol-binding clathrin assembly protein Picalm tr|Q66WT9|Q66WT9_RAT Clathrin-assembly lymphoid myeloid leukemia protein OS=Rattus norvegicus OX=10116 GN=Picalm PE=1 SV=1;tr|A0A0G2JUQ5|A0A0G2JUQ5_RAT Phosphatidylinositol binding clathrin assembly protein, isoform CRA_d OS=Rattus norvegicus OX=10116 GN=P 6 3 3 3 2 0 3 3 2 2 2 0 3 3 2 2 2 0 3 3 2 2 10.6 10.6 10.6 64.713 597 597;640;647;655;640;610 4.83 2 10 0 28.126 By matching By MS/MS By MS/MS By MS/MS By MS/MS 1.0218 3.9524 22.115 12 1 Leave out requantified 1.1145 3.1564 35.513 11 1 Leave out requantified 1.1761 0.77501 18.282 12 1 Leave out requantified 0.84351 NaN 1.2071 0.92025 1.0403 1.2552 2.5379 NaN 5.0468 3.3483 4.1372 5.1484 3.1711 NaN 26.104 53.414 24.043 2.4785 2 0 3 3 2 2 0 0 0 1 0 0 Median Median Leave out requantified Median Median Median 1.0586 NaN 1.0146 1.6647 1.3164 1.4348 2.2243 NaN 2.2708 4.2831 3.7065 4.027 NaN NaN 33.234 21.563 17.728 30.913 1 0 3 3 2 2 0 0 0 1 0 0 Median Median Leave out requantified Median Median Median 1.3493 NaN 0.98 1.8089 1.1023 1.333 0.99363 NaN 0.75776 1.3662 0.747 0.99072 9.2222 NaN 10.028 38.216 8.3876 6.2705 2 0 3 3 2 2 0 0 0 1 0 0 Median Median Leave out requantified Median Median Median 5.9 0 10.6 10.6 6.7 8.5 94713000 26797000 28865000 39051000 4390000 1185500 1421800 1782700 0 0 0 0 23617000 7976800 7302000 8338600 27013000 7742900 7136900 12134000 8466100 2108300 3219800 3137900 31226000 7783100 9784600 13659000 82 7985;9210;9927 True;True;True 8477;9769;10528 72747;72748;72749;72750;82445;82446;82447;82448;89504;89505;89506;89507 105835;105836;105837;119825;119826;119827;119828;130123;130124;130125;130126;130127;130128;130129;130130 105837;119826;130129

D3ZB30;A0A0G2JTV1;Q6P736;Q00438;Q9Z118 D3ZB30;A0A0G2JTV1;Q6P736;Q00438 10;10;10;10;2 10;10;10;10;2 8;8;8;8;0 Polypyrimidine tract-binding protein 1 Ptbp1 tr|D3ZB30|D3ZB30_RAT Polypyrimidine tract binding protein 1, isoform CRA_c OS=Rattus norvegicus OX=10116 GN=Ptbp1 PE=1 SV=1;tr|A0A0G2JTV1|A0A0G2JTV1_RAT Polypyrimidine tract-binding protein 1 OS=Rattus norvegicus OX=10116 GN=Ptbp1 PE=1 SV=1;tr|Q6P736|Q6P73 5 10 10 8 7 7 7 4 5 6 7 7 7 4 5 6 6 5 7 4 4 6 26.8 26.8 24 56.935 530 530;554;556;555;523 4.28 25 5 4 5 33 38 2 1 0 31.592 By MS/MS By MS/MS By MS/MS By MS/MS By MS/MS By MS/MS 0.32606 1.3229 33.637 89 47 Leave out requantified 0.41078 1.1779 18.657 89 47 Leave out requantified 1.3327 1.0206 54.313 89 47 Leave out requantified 0.26047 0.28664 0.32724 0.28873 0.35301 0.32151 1.3294 1.2711 1.4479 0.91998 1.5909 1.219 15.636 46.557 74.373 5.9363 16.638 14.378 16 14 16 15 13 15 9 6 10 8 8 6 Leave out requantified Leave out requantified Leave out requantified Leave out requantified Leave out requantified Leave out requantified 0.40319 0.38477 0.39743 0.54608 0.46662 0.48622 1.24 1.3072 1.1972 1.3585 1.4907 1.2678 10.657 47.65 18.484 21.739 13.511 20.299 16 14 16 15 13 15 9 6 10 8 8 6 Leave out requantified Leave out requantified Leave out requantified Leave out requantified Leave out requantified Leave out requantified 1.4943 1.3136 1.1245 1.6851 1.4128 1.5031 1.0567 0.97926 0.87293 1.1432 0.93591 1.1939 1.2248 76.725 67.126 29.044 3.3623 33.99 16 14 16 15 13 15 9 6 10 8 8 6 Leave out requantified Leave out requantified Leave out requantified Leave out requantified Leave out requantified Leave out requantified 17.9 14.3 20.6 9.6 11.5 18.1 758610000 430900000 134300000 193410000 101470000 59577000 16908000 24989000 124390000 72664000 23736000 27989000 118770000 68987000 21068000 28711000 139590000 78652000 23227000 37708000 121860000 65127000 22200000 34529000 152530000 85894000 27157000 39483000 83 2519;4904;6512;6757;6959;7477;7705;9391;10020;11819 True;True;True;True;True;True;True;True;True;True 2679;5162;6863;7112;7332;7333;7933;8181;9957;10623;12544 23819;23820;23821;23822;23823;23824;23825;23826;23827;23828;23829;23830;23831;23832;23833;23834;23835;23836;23837;23838;23839;23840;23841;23842;23843;23844;23845;44638;44639;44640;44641;44642;58612;58613;58614;58615;58616;58617;60765;60766;60767;62608;62609;62610;62611;62612;62613;62614;62615;62616;62617;62618;62619;62620;62621;62622;62623;62624;62625;62626;62627;62628;62629;62630;62631;62632;62633;62634;62635;62636;62637;62638;62639;62640;62641;62642;62643;62644;62645;62646;62647;62648;62649;62650;62651;62652;62653;62654;62655;62656;62657;62658;67440;67441;67442;67443;67444;67445;69913;84411;84412;84413;84414;84415;84416;84417;90359;90360;90361;90362;90363;90364;109548 33337;33338;33339;33340;33341;33342;33343;33344;33345;33346;33347;33348;33349;33350;33351;33352;33353;33354;33355;33356;33357;33358;33359;33360;33361;33362;33363;33364;33365;33366;33367;33368;33369;33370;33371;63984;63985;63986;63987;63988;63989;85907;85908;85909;85910;85911;85912;85913;85914;85915;85916;88999;89000;91436;91437;91438;91439;91440;91441;91442;91443;91444;91445;91446;91447;91448;91449;91450;91451;91452;91453;91454;91455;91456;91457;91458;91459;91460;91461;91462;91463;91464;91465;91466;91467;91468;91469;91470;91471;91472;91473;91474;91475;91476;91477;91478;91479;91480;91481;91482;91483;91484;91485;91486;91487;91488;91489;91490;91491;91492;91493;91494;91495;91496;91497;91498;91499;91500;91501;91502;91503;91504;98201;98202;98203;98204;98205;98206;98207;98208;98209;98210;98211;98212;98213;98214;101834;122821;122822;122823;122824;122825;122826;122827;131322;131323;131324;131325;131326;131327;131328;131329;159564 33351;63986;85909;88999;91482;98211;101834;122821;131329;159564 71 1

D3ZK56;A0A0G2JTW1;P61227 D3ZK56;A0A0G2JTW1;P61227 1;1;1 1;1;1 1;1;1 Ras-related protein Rap-2b Rap2c;Rap2b tr|D3ZK56|D3ZK56_RAT RAP2C, member of RAS oncogene family OS=Rattus norvegicus OX=10116 GN=Rap2c PE=1 SV=2;tr|A0A0G2JTW1|A0A0G2JTW1_RAT RAS related protein 2a OS=Rattus norvegicus OX=10116 GN=Rap2a PE=4 SV=1;sp|P61227|RAP2B_RAT Ras-related protein Rap-2b O 3 1 1 1 1 1 1 1 1 1 1 1 1 1 1 1 1 1 1 1 1 1 6 6 6 20.745 183 183;183;183 9.62 3 5 0 8.8442 By matching By MS/MS By MS/MS By MS/MS By MS/MS By matching 0.45485 2.0133 9.8369 8 1 Median 0.49337 1.6317 26.664 8 1 Median 1.0982 0.76362 13.688 8 1 Median 0.59058 0.42865 0.4662 0.48423 0.4082 0.4698 2.3607 1.8516 1.9532 2.1758 1.8381 1.9789 NaN NaN 7.8077 8.5385 NaN NaN 1 1 2 2 1 1 0 0 1 0 0 0 Median Median Median Median Median Median 0.94525 0.47813 0.5423 0.43295 0.42773 0.5705 2.7191 1.6588 1.8042 1.275 1.2402 1.6051 NaN NaN 6.9927 12.102 NaN NaN 1 1 2 2 1 1 0 0 1 0 0 0 Median Median Median Median Median Median 1.1123 1.0285 1.1564 0.97438 1.3157 1.2091 0.71861 0.77051 0.82886 0.67637 0.91314 0.85641 NaN NaN 13.208 15.888 NaN NaN 1 1 2 2 1 1 0 0 1 0 0 0 Median Median Median Median Median Median 6 6 6 6 6 6 23042000 11942000 4750500 6349200 1470800 404430 499640 566720 1456400 805260 276650 374470 4281100 2162900 892410 1225700 6416300 3604000 1273200 1539100 2746100 1564400 508930 672790 6671200 3401100 1299700 1970400 84 11948 True 12677 110556;110557;110558;110559;110560;110561;110562;110563 160946;160947;160948;160949;160950 160948

A0A0G2JTX2 A0A0G2JTX2 2 2 2 tr|A0A0G2JTX2|A0A0G2JTX2_RAT PRA1 family protein OS=Rattus norvegicus OX=10116 GN=Praf2 PE=1 SV=1 1 2 2 2 2 2 2 2 2 2 2 2 2 2 2 2 2 2 2 2 2 2 18 18 18 19.078 178 178 3.24 24 20 11 1 5 6 0 20.79 By MS/MS By MS/MS By MS/MS By MS/MS By MS/MS By MS/MS 0.3 0.75359 28.057 7 7 Median 0.31073 0.47612 34.303 7 7 Median 1.0968 0.73096 46.051 7 7 Median NaN 0.30418 0.3 0.21443 0.26137 0.31099 NaN 0.73618 0.75359 0.97281 0.65801 0.75885 NaN NaN NaN NaN NaN 40.156 0 1 1 1 1 3 0 1 1 1 1 3 Median Median Median Median Median Median NaN 0.33362 0.32564 0.26314 0.31867 0.30545 NaN 0.47403 0.45981 0.78828 0.4922 0.47612 NaN NaN NaN NaN NaN 50.001 0 1 1 1 1 3 0 1 1 1 1 3 Median Median Median Median Median Median NaN 1.0968 1.0855 1.2272 1.2192 0.98218 NaN 0.73096 0.69034 0.85288 0.76516 0.66161 NaN NaN NaN NaN NaN 76.091 0 1 1 1 1 3 0 1 1 1 1 3 Median Median Median Median Median Median 18 18 18 18 18 18 32794000 18963000 7075800 6754600 0 0 0 0 5088200 3131600 740320 1216300 3152500 1786400 666140 699890 2731600 1799700 451110 480840 8841200 5477800 1314500 2048900 12980000 6768000 3903700 2308600 85 7562;8852 True;True 8020;9381;9382 68093;68094;68095;68096;68097;68098;68099;68100;79013;79014;79015;79016;79017;79018;79019;79020;79021;79022;79023;79024;79025;79026;79027;79028;79029;79030;79031;79032;79033;79034;79035;79036;79037;79038;79039;79040;79041;79042;79043;79044;79045;79046;79047;79048;79049;79050;79051;79052;79053;79054;79055;79056;79057;79058;79059;79060;79061;79062;79063;79064;79065;79066;79067;79068;79069;79070;79071 99105;99106;99107;99108;99109;99110;99111;99112;99113;99114;99115;99116;99117;114921;114922;114923;114924;114925;114926;114927;114928;114929;114930;114931;114932;114933;114934;114935;114936;114937;114938;114939;114940;114941;114942;114943;114944;114945;114946;114947;114948;114949;114950;114951;114952;114953;114954;114955;114956;114957;114958;114959;114960;114961;114962;114963;114964;114965;114966;114967;114968;114969;114970;114971;114972;114973;114974;114975;114976;114977;114978;114979;114980;114981;114982;114983;114984;114985;114986;114987;114988;114989;114990;114991;114992;114993;114994;114995;114996 99105;114969 72 163

A0A0G2JTY6;F1LRK4 A0A0G2JTY6;F1LRK4 1;1 1;1 1;1 Grsf1 tr|A0A0G2JTY6|A0A0G2JTY6_RAT G-rich RNA sequence-binding factor 1 OS=Rattus norvegicus OX=10116 GN=Grsf1 PE=1 SV=1;tr|F1LRK4|F1LRK4_RAT G-rich RNA sequence-binding factor 1 OS=Rattus norvegicus OX=10116 GN=Grsf1 PE=1 SV=2 2 1 1 1 1 1 0 0 0 0 1 1 0 0 0 0 1 1 0 0 0 0 5.2 5.2 5.2 43.549 381 381;479 6 2 0.0037915 1.9921 By MS/MS By MS/MS 1.233 5.8605 22.068 2 0 Median 1.6883 5.8276 32.422 2 0 Median 1.3201 0.98859 16.685 2 0 Median 1.1518 1.32 NaN NaN NaN NaN 5.0138 6.8502 NaN NaN NaN NaN NaN NaN NaN NaN NaN NaN 1 1 0 0 0 0 0 0 0 0 0 0 Median Median Median Median Median Median 1.4415 1.9775 NaN NaN NaN NaN 4.6337 7.3292 NaN NaN NaN NaN NaN NaN NaN NaN NaN NaN 1 1 0 0 0 0 0 0 0 0 0 0 Median Median Median Median Median Median 1.5266 1.1416 NaN NaN NaN NaN 1.1124 0.87857 NaN NaN NaN NaN NaN NaN NaN NaN NaN NaN 1 1 0 0 0 0 0 0 0 0 0 0 Median Median Median Median Median Median 5.2 5.2 0 0 0 0 3691400 940350 1256200 1494800 1003700 299380 355680 348690 2687600 640980 900500 1146200 0 0 0 0 0 0 0 0 0 0 0 0 0 0 0 0 86 10272 True 10889 92440;92441 134211;134212 134212 73 203

D3ZGM1;A0A0G2JU15 D3ZGM1;A0A0G2JU15 2;2 2;2 2;2 Ptcd3 tr|D3ZGM1|D3ZGM1_RAT Pentatricopeptide repeat domain 3 OS=Rattus norvegicus OX=10116 GN=Ptcd3 PE=1 SV=1;tr|A0A0G2JU15|A0A0G2JU15_RAT Pentatricopeptide repeat domain 3 OS=Rattus norvegicus OX=10116 GN=Ptcd3 PE=1 SV=1 2 2 2 2 0 0 2 0 0 0 0 0 2 0 0 0 0 0 2 0 0 0 6.6 6.6 6.6 77.89 687 687;747 5 2 0 4.4145 By MS/MS 0.39702 1.231 NaN 1 1 Median 0.54719 1.2917 NaN 1 1 Median 1.3782 1.0459 NaN 1 1 Median NaN NaN 0.39702 NaN NaN NaN NaN NaN 1.231 NaN NaN NaN NaN NaN NaN NaN NaN NaN 0 0 1 0 0 0 0 0 1 0 0 0 Median Median Median Median Median Median NaN NaN 0.54719 NaN NaN NaN NaN NaN 1.2917 NaN NaN NaN NaN NaN NaN NaN NaN NaN 0 0 1 0 0 0 0 0 1 0 0 0 Median Median Median Median Median Median NaN NaN 1.3782 NaN NaN NaN NaN NaN 1.0459 NaN NaN NaN NaN NaN NaN NaN NaN NaN 0 0 1 0 0 0 0 0 1 0 0 0 Median Median Median Median Median Median 0 0 6.6 0 0 0 6216800 4910400 514390 792010 0 0 0 0 0 0 0 0 6216800 4910400 514390 792010 0 0 0 0 0 0 0 0 0 0 0 0 87 1938;4917 True;True 2052;5175 17713;44783 24936;64252;64253 24936;64252 74 141

D3ZFD2;A0A0G2JU25 D3ZFD2;A0A0G2JU25 4;4 4;4 4;4 Polypeptide N-acetylgalactosaminyltransferase Galnt2 tr|D3ZFD2|D3ZFD2_RAT Polypeptide N-acetylgalactosaminyltransferase OS=Rattus norvegicus OX=10116 GN=Galnt2 PE=1 SV=1;tr|A0A0G2JU25|A0A0G2JU25_RAT Polypeptide N-acetylgalactosaminyltransferase OS=Rattus norvegicus OX=10116 GN=Galnt2 PE=1 SV=1 2 4 4 4 2 1 3 1 2 3 2 1 3 1 2 3 2 1 3 1 2 3 12.9 12.9 12.9 53.783 473 473;542 5.2 12 3 0 8.9092 By MS/MS By MS/MS By MS/MS By MS/MS By MS/MS By MS/MS 0.31591 1.5012 3.1448 12 5 Leave out requantified 0.47231 1.3081 26.964 12 5 Leave out requantified 1.5153 1.1461 7.5896 12 5 Leave out requantified 0.35473 0.29805 0.38944 0.25106 0.23778 0.32079 2.0622 1.5024 1.535 1.3407 1.1424 1.427 51.303 NaN 14.18 NaN NaN 25.491 2 1 5 1 1 2 1 0 2 1 0 1 Median Median Median Median Median Median 0.53737 0.40851 0.49257 14.904 0.37659 0.3756 2.3251 1.4807 1.1053 53.452 1.1724 1.1579 8.5248 NaN 40.999 NaN NaN 48.95 2 1 5 1 1 2 1 0 2 1 0 1 Median Median Median Median Median Median 1.4108 1.4696 1.4485 59.363 1.4086 1.0621 1.0035 1.1205 1.0862 44.773 0.96024 0.80394 41.912 NaN 28.266 NaN NaN 43.627 2 1 5 1 1 2 1 0 2 1 0 1 Median Median Median Median Median Median 7.6 2.7 11.4 2.7 4.2 11.4 203130000 58635000 18843000 125650000 11563000 6069200 2015200 3479000 10826000 5978100 2003900 2843800 40662000 22305000 7363400 10994000 107600000 4216600 1664800 101720000 11649000 7142300 2092200 2414700 20821000 12924000 3703200 4193800 88 2870;3873;9194;10615 True;True;True;True 3041;4091;9752;11253;11254 27146;27147;27148;27149;27150;34868;82376;82377;82378;95816;95817;95818;95819;95820;95821 38119;38120;38121;38122;38123;38124;38125;48971;119744;119745;119746;139275;139276;139277;139278;139279;139280;139281;139282;139283;139284 38120;48971;119744;139276 75;76 238;246

B0BN02;A0A0G2JU49 B0BN02;A0A0G2JU49 1;1 1;1 1;1 Mtx1 tr|B0BN02|B0BN02_RAT Metaxin 1 OS=Rattus norvegicus OX=10116 GN=Mtx1 PE=1 SV=1;tr|A0A0G2JU49|A0A0G2JU49_RAT Metaxin 1 OS=Rattus norvegicus OX=10116 GN=Mtx1 PE=1 SV=1 2 1 1 1 0 0 0 1 0 0 0 0 0 1 0 0 0 0 0 1 0 0 4.9 4.9 4.9 48.443 432 432;463 7 1 0.0064905 1.8535 By MS/MS NaN NaN NaN 0 0 Median NaN NaN NaN 0 0 Median NaN NaN NaN 0 0 Median NaN NaN NaN NaN NaN NaN NaN NaN NaN NaN NaN NaN NaN NaN NaN NaN NaN NaN 0 0 0 0 0 0 0 0 0 0 0 0 Median Median Median Median Median Median NaN NaN NaN NaN NaN NaN NaN NaN NaN NaN NaN NaN NaN NaN NaN NaN NaN NaN 0 0 0 0 0 0 0 0 0 0 0 0 Median Median Median Median Median Median NaN NaN NaN NaN NaN NaN NaN NaN NaN NaN NaN NaN NaN NaN NaN NaN NaN NaN 0 0 0 0 0 0 0 0 0 0 0 0 Median Median Median Median Median Median 0 0 0 4.9 0 0 1348400 1348400 0 0 0 0 0 0 0 0 0 0 0 0 0 0 1348400 1348400 0 0 0 0 0 0 0 0 0 0 89 5395 True 5692 49477 71327 71327

F1M836;A0A0G2JU56;Q8VHF0;A0A1B0GWN5 F1M836;A0A0G2JU56;Q8VHF0;A0A1B0GWN5 3;3;3;2 3;3;3;2 3;3;3;2 MAP/microtubule affinity-regulating kinase 3 Mark3 tr|F1M836|F1M836_RAT Non-specific serine/threonine protein kinase OS=Rattus norvegicus OX=10116 GN=Mark3 PE=1 SV=1;tr|A0A0G2JU56|A0A0G2JU56_RAT Non-specific serine/threonine protein kinase OS=Rattus norvegicus OX=10116 GN=Mark3 PE=1 SV=1;sp|Q8VHF0|MARK3_RA 4 3 3 3 1 1 2 2 1 1 1 1 2 2 1 1 1 1 2 2 1 1 5.9 5.9 5.9 88.72 797 797;798;797;597 3.33 2 7 0 45.496 By MS/MS By MS/MS By MS/MS By MS/MS By MS/MS By MS/MS 1.3795 4.449 19.587 6 0 Median 1.5485 3.6116 22.041 6 0 Median 1.1349 0.85378 16.106 6 0 Median 1.4662 1.5242 1.4966 1.2979 1.2603 1.2829 5.6024 4.904 4.6051 3.9115 4.2981 3.177 NaN NaN NaN NaN NaN NaN 1 1 1 1 1 1 0 0 0 0 0 0 Median Median Median Median Median Median 1.7063 1.09 1.4054 1.7088 1.7743 1.2766 4.1555 2.7967 3.235 4.0319 4.6492 2.7578 NaN NaN NaN NaN NaN NaN 1 1 1 1 1 1 0 0 0 0 0 0 Median Median Median Median Median Median 1.3085 0.79981 0.97414 1.2296 1.2902 1.0475 0.896 0.61482 0.76237 0.87115 0.97393 0.83677 NaN NaN NaN NaN NaN NaN 1 1 1 1 1 1 0 0 0 0 0 0 Median Median Median Median Median Median 2.4 2.4 3.9 4.4 2.4 2.4 51968000 20879000 14819000 16269000 5021900 1381500 1757300 1883000 4754000 1089300 1815300 1849300 8298100 3722300 2533500 2042300 15580000 9786100 2603200 3190800 10698000 2833600 3492000 4372700 7615400 2066300 2618000 2931100 90 8821;8940;10802 True;True;True 9350;9471;11453 78600;79662;79663;79664;79665;79666;79667;79668;97728 114345;115884;115885;115886;115887;115888;115889;115890;115891;115892;115893;141943 114345;115890;141943

A0A0G2JU77 A0A0G2JU77 2 2 2 tr|A0A0G2JU77|A0A0G2JU77_RAT Eukaryotic translation initiation factor 3 subunit K OS=Rattus norvegicus OX=10116 GN=Eif3k PE=1 SV=1 1 2 2 2 0 0 0 1 1 0 0 0 0 1 1 0 0 0 0 1 1 0 13 13 13 24.792 215 215 9 2 0.0029297 2.2106 By MS/MS By MS/MS 0.43856 1.9635 20.846 2 2 Median 0.75803 2.2606 0.44351 2 2 Median 1.7284 1.186 17.826 2 2 Median NaN NaN NaN 0.50537 0.38058 NaN NaN NaN NaN 2.2754 1.6944 NaN NaN NaN NaN NaN NaN NaN 0 0 0 1 1 0 0 0 0 1 1 0 Median Median Median Median Median Median NaN NaN NaN 0.76728 0.74889 NaN NaN NaN NaN 2.2677 2.2535 NaN NaN NaN NaN NaN NaN NaN 0 0 0 1 1 0 0 0 0 1 1 0 Median Median Median Median Median Median NaN NaN NaN 1.5182 1.9678 NaN NaN NaN NaN 1.0456 1.3453 NaN NaN NaN NaN NaN NaN NaN 0 0 0 1 1 0 0 0 0 1 1 0 Median Median Median Median Median Median 0 0 0 6.5 6.5 0 5256100 2489800 1121700 1644500 0 0 0 0 0 0 0 0 0 0 0 0 4111600 1849800 973770 1288000 1144400 639990 147920 356490 0 0 0 0 91 852;3104 True;True 898;3283 7391;28940 10641;40674 10641;40674

A0A0G2JU89;F1LQI5;Q2TL32 A0A0G2JU89;F1LQI5;Q2TL32 13;13;13 13;13;13 13;13;13 E3 ubiquitin-protein ligase UBR4 Ubr4 tr|A0A0G2JU89|A0A0G2JU89_RAT E3 ubiquitin-protein ligase UBR4 OS=Rattus norvegicus OX=10116 GN=Ubr4 PE=1 SV=1;tr|F1LQI5|F1LQI5_RAT E3 ubiquitin-protein ligase UBR4 OS=Rattus norvegicus OX=10116 GN=Ubr4 PE=1 SV=1;sp|Q2TL32|UBR4_RAT E3 ubiquitin-protein liga 3 13 13 13 4 7 7 3 10 7 4 7 7 3 10 7 4 7 7 3 10 7 3.3 3.3 3.3 573.87 5193 5193;5194;5194 1.02 41 1 0 41.469 By MS/MS By MS/MS By MS/MS By MS/MS By MS/MS By MS/MS 0.3849 1.1593 40.731 33 7 Leave out requantified 0.46444 0.87985 30.856 34 7 Leave out requantified 1.0785 0.85274 30.003 34 7 Leave out requantified 0.31912 0.41695 0.4006 0.29017 0.44668 0.38029 0.82166 1.3519 1.6053 0.64806 1.1995 1.3609 26.986 20.11 41.008 58.838 43.618 30.067 4 6 5 3 10 5 0 3 1 0 2 1 Leave out requantified Leave out requantified Leave out requantified Leave out requantified Leave out requantified Leave out requantified 0.5711 0.4545 0.51771 0.38854 0.35206 0.50399 1.234 1.1542 1.3726 0.79402 0.86138 1.3933 29.723 7.8727 28.811 26.136 25.161 36.543 4 6 5 3 10 6 0 3 1 0 2 1 Leave out requantified Leave out requantified Leave out requantified Leave out requantified Leave out requantified Leave out requantified 1.6909 1.0051 1.1748 1.0785 0.77922 1.2056 1.2094 0.8037 0.95103 0.801 0.60729 0.88358 22.102 6.1715 4.3824 33.642 43.044 16.228 4 6 5 3 10 6 0 3 1 0 2 1 Leave out requantified Leave out requantified Leave out requantified Leave out requantified Leave out requantified Leave out requantified 1.1 1.8 1.7 0.7 2.4 2.1 353300000 185920000 81734000 85648000 25899000 13267000 5233900 7398100 45855000 24572000 10633000 10651000 45564000 23250000 10868000 11446000 38828000 22515000 8920100 7392700 115290000 60386000 29199000 25710000 81859000 41927000 16881000 23051000 92 129;139;429;757;1494;2952;3023;3445;4267;5039;5134;5135;7899 True;True;True;True;True;True;True;True;True;True;True;True;True 134;144;450;796;797;1579;3126;3197;3639;4500;5302;5400;5401;8387 998;999;1000;1001;1002;1060;1061;3897;6601;6602;6603;6604;6605;6606;6607;6608;13303;13304;13305;13306;13307;27979;27980;27981;27982;27983;27984;27985;28377;28378;31456;38465;38466;46083;46084;46623;46624;46625;46626;46627;46628;71782 1358;1359;1360;1361;1362;1363;1452;1453;5498;9418;9419;9420;9421;9422;9423;9424;9425;9426;9427;9428;9429;9430;9431;9432;9433;9434;19012;19013;19014;19015;19016;19017;19018;19019;19020;19021;39503;39504;39505;39506;39507;39508;39509;39510;39984;39985;44115;54422;54423;66421;66422;67127;67128;67129;67130;67131;67132;67133;67134;104498 1359;1453;5498;9418;19013;39508;39985;44115;54422;66422;67129;67133;104498 77;78;79 4320;4416;4819

A0A0G2JUA5;A0A0G2JU96 A0A0G2JUA5;A0A0G2JU96 253;251 253;251 253;251 tr|A0A0G2JUA5|A0A0G2JUA5_RAT AHNAK nucleoprotein OS=Rattus norvegicus OX=10116 GN=Ahnak PE=1 SV=1;tr|A0A0G2JU96|A0A0G2JU96_RAT AHNAK nucleoprotein OS=Rattus norvegicus OX=10116 GN=Ahnak PE=1 SV=1 2 253 253 253 198 203 204 195 213 221 198 203 204 195 213 221 198 203 204 195 213 221 75.3 75.3 75.3 581.12 5450 5450;5364 2.29 1381 1872 1270 587 104 17 4 4 1 0 323.31 By MS/MS By MS/MS By MS/MS By MS/MS By MS/MS By MS/MS 0.45141 1.3502 23.089 4991 1047 Leave out requantified 0.68693 1.5788 21.911 4993 1044 Leave out requantified 1.5144 1.0524 15.605 4985 1044 Leave out requantified 0.41145 0.43469 0.44743 0.4618 0.46767 0.48021 1.2656 1.3551 1.4227 1.2741 1.3196 1.3731 22.343 23.323 25.456 29.462 26.628 30.034 769 723 889 758 913 939 183 184 195 175 159 151 Leave out requantified Leave out requantified Leave out requantified Leave out requantified Leave out requantified Leave out requantified 0.69859 0.69935 0.68723 0.66876 0.67856 0.65245 1.5506 1.7278 1.6144 1.4112 1.6982 1.5476 23.92 30.262 26.457 24.378 24.657 22.291 769 722 889 759 915 939 183 183 195 174 159 150 Leave out requantified Leave out requantified Leave out requantified Leave out requantified Leave out requantified Leave out requantified 1.714 1.5673 1.5319 1.4332 1.4639 1.3351 1.1256 1.1952 1.0986 0.96892 1.0397 0.95064 19.542 16.012 14.53 17.649 18.843 18.582 769 720 887 758 915 936 183 183 195 174 159 150 Leave out requantified Leave out requantified Leave out requantified Leave out requantified Leave out requantified Leave out requantified 68.2 72.4 72 67.5 70.8 70.6 187950000000 87524000000 39957000000 60465000000 20855000000 9686900000 4126700000 7041100000 23134000000 10691000000 4847600000 7594700000 30742000000 14306000000 6506300000 9929600000 29535000000 13796000000 6279400000 9459000000 41501000000 19279000000 9023300000 13198000000 42181000000 19765000000 9174100000 13243000000 93 88;243;253;254;255;256;257;258;277;289;325;326;327;328;329;330;331;332;333;385;386;401;662;916;917;918;952;982;983;984;985;986;987;988;1949;3640;3673;3684;3714;3723;3724;3725;3726;3787;3843;3858;3869;3870;3874;3875;3876;3884;3885;3897;3981;4050;4074;4075;4079;4081;4094;4112;4113;4117;4118;4154;4156;4157;4168;4169;4184;4185;4186;4283;4284;4382;4383;4395;4401;4402;4403;4404;4406;4407;4408;4409;4410;4411;4414;4415;4418;4419;4423;4424;4425;4426;4427;4428;4429;4430;4431;4432;4433;4434;4446;4447;4449;4450;4451;4452;4453;4458;4459;4460;4463;4464;4467;4469;4470;4474;4475;4476;4477;4570;4594;4664;5094;5146;5147;5148;5149;5150;5279;5374;5378;5379;5380;5381;5382;5383;5384;5385;5386;5387;5388;5389;5390;5391;5392;5393;5827;5858;6110;6314;6315;6506;6540;6646;6801;6966;6967;6968;6969;6970;7006;7007;7120;7212;7213;7214;7216;7217;7218;7221;7227;7228;7286;7287;7357;8102;8959;9227;9635;9636;9637;9638;10193;10595;10596;10609;10619;10625;10631;10632;10644;10854;11066;11067;11068;11069;11070;11071;11079;11080;11083;11084;11089;11096;11114;11115;11196;11292;11293;11294;11295;11296;11297;11298;11299;11300;11301;11302;11303;11304;11305;11306;11307;11308;11507;11508;11527;11528;11529;11530;11531;11532;11533;11539;11590;11591;11605;11628;11629;11654;11670;11671;11702;11704;11724;11744;11745;11761;11809 True;True;True;True;True;True;True;True;True;True;True;True;True;True;True;True;True;True;True;True;True;True;True;True;True;True;True;True;True;True;True;True;True;True;True;True;True;True;True;True;True;True;True;True;True;True;True;True;True;True;True;True;True;True;True;True;True;True;True;True;True;True;True;True;True;True;True;True;True;True;True;True;True;True;True;True;True;True;True;True;True;True;True;True;True;True;True;True;True;True;True;True;True;True;True;True;True;True;True;True;True;True;True;True;True;True;True;True;True;True;True;True;True;True;True;True;True;True;True;True;True;True;True;True;True;True;True;True;True;True;True;True;True;True;True;True;True;True;True;True;True;True;True;True;True;True;True;True;True;True;True;True;True;True;True;True;True;True;True;True;True;True;True;True;True;True;True;True;True;True;True;True;True;True;True;True;True;True;True;True;True;True;True;True;True;True;True;True;True;True;True;True;True;True;True;True;True;True;True;True;True;True;True;True;True;True;True;True;True;True;True;True;True;True;True;True;True;True;True;True;True;True;True;True;True;True;True;True;True;True;True;True;True;True;True;True;True;True;True;True;True;True;True;True;True;True;True;True;True;True;True;True;True 91;255;269;270;271;272;273;274;295;307;343;344;345;346;347;348;349;350;351;404;405;421;698;972;973;974;975;1010;1042;1043;1044;1045;1046;1047;1048;1049;1050;2064;2065;3842;3876;3887;3919;3929;3930;3931;3932;3933;4001;4060;4075;4086;4087;4092;4093;4094;4095;4096;4104;4105;4118;4206;4278;4304;4305;4309;4311;4324;4342;4343;4347;4348;4384;4386;4387;4399;4400;4415;4416;4417;4418;4516;4517;4622;4623;4635;4641;4642;4643;4644;4646;4647;4648;4649;4650;4651;4654;4655;4658;4659;4663;4664;4665;4666;4667;4668;4669;4670;4671;4672;4673;4674;4675;4688;4689;4691;4692;4693;4694;4695;4696;4701;4702;4703;4704;4707;4708;4711;4712;4714;4715;4716;4720;4721;4722;4723;4818;4842;4915;5358;5359;5413;5414;5415;5416;5417;5560;5657;5662;5663;5664;5665;5666;5667;5668;5669;5670;5671;5672;5673;5674;5675;5676;5677;5678;5679;5680;5681;5682;5683;5684;5685;5686;5687;5688;5689;5690;6150;6182;6446;6657;6658;6659;6857;6892;7001;7158;7341;7342;7343;7344;7345;7387;7388;7517;7620;7621;7622;7625;7626;7627;7628;7629;7632;7633;7640;7641;7642;7643;7715;7716;7717;7799;7800;8603;9491;9492;9787;10221;10222;10223;10224;10804;11229;11230;11231;11245;11259;11265;11266;11267;11273;11274;11286;11509;11737;11738;11739;11740;11741;11742;11750;11751;11754;11755;11760;11767;11785;11786;11872;11974;11975;11976;11977;11978;11979;11980;11981;11982;11983;11984;11985;11986;11987;11988;11989;11990;11991;11992;11993;11994;11995;12212;12213;12232;12233;12234;12235;12236;12237;12238;12244;12297;12298;12312;12336;12337;12364;12384;12385;12419;12421;12442;12443;12465;12466;12482;12531 729;730;731;732;733;734;735;736;2273;2408;2409;2410;2411;2412;2413;2414;2415;2416;2417;2418;2419;2420;2421;2422;2423;2424;2425;2426;2592;2593;2594;2686;2687;2688;2689;2690;2691;2692;2693;2694;3111;3112;3113;3114;3115;3116;3117;3118;3119;3120;3121;3122;3123;3124;3125;3126;3127;3128;3129;3130;3131;3132;3133;3134;3135;3136;3137;3138;3139;3140;3141;3142;3143;3144;3145;3146;3147;3148;3149;3150;3151;3152;3153;3154;3155;3156;3157;3158;3159;3160;3161;3162;3163;3164;3165;3166;3167;3168;3169;3170;3171;3172;3173;3174;3175;3176;3177;3178;3179;3180;3181;3182;3183;3184;3185;3186;3187;3188;3189;3528;3529;3530;3531;3532;3533;3534;3535;3536;3537;3538;3539;3540;3541;3542;3543;3544;3545;3546;3547;3548;3549;3550;3551;3552;3553;3554;3555;3556;3557;3558;3683;5683;5684;5685;5686;5687;5688;5689;5690;5691;5692;5693;5694;5695;5696;5697;5698;5699;5700;5701;5702;5703;5704;5705;5706;5707;5708;5709;5710;5711;5712;5713;5714;5715;8052;8053;8054;8055;8056;8057;8058;8059;8060;8061;8062;8063;8064;8065;8066;8067;8068;8069;8070;8071;8072;8073;8074;8075;8076;8077;8078;8079;8080;8081;8082;8083;8084;8085;8086;8087;8088;8089;8090;8091;8092;8093;8094;8095;8096;8097;8388;8389;8390;8391;8392;8393;8394;8395;8396;8397;8398;8399;8622;8623;8624;8625;8626;8627;8628;8629;8630;8631;8632;8633;8634;8635;8636;8637;8638;8639;8640;8641;8642;8643;8644;8645;8646;8647;8648;8649;8650;8651;8652;8653;8654;8655;8656;8657;8658;8659;8660;8661;8662;8663;8664;8665;8666;8667;8668;8669;8670;8671;8672;8673;8674;8675;8676;8677;8678;8679;8680;8681;8682;8683;8684;8685;8686;8687;17792;17793;17794;17795;17796;17797;17798;17799;17800;17801;17802;17803;17804;17805;17806;17807;17808;17809;17810;17811;17812;17813;17814;17815;17816;17817;17818;17819;17820;17821;17822;17823;17824;17825;17826;17827;17828;17829;17830;17831;17832;33058;33059;33060;33253;33254;33255;33256;33257;33258;33259;33260;33261;33262;33263;33264;33265;33314;33315;33316;33317;33318;33319;33320;33321;33322;33323;33324;33325;33326;33327;33328;33329;33330;33331;33332;33333;33334;33335;33336;33337;33521;33522;33523;33578;33579;33580;33581;33582;33583;33584;33585;33586;33587;33588;33589;33590;33591;33592;33593;33594;33595;33596;33597;33598;33599;33600;33601;33602;33603;33604;33605;33606;33607;33608;33609;33610;33611;33612;33613;33614;33615;33616;33617;33618;33619;33620;33621;33622;33623;33624;33625;33626;33627;33628;33629;33630;33631;34076;34077;34078;34079;34080;34081;34082;34083;34084;34085;34086;34087;34088;34089;34090;34091;34092;34093;34094;34095;34586;34587;34588;34589;34590;34591;34592;34593;34594;34595;34596;34597;34598;34599;34600;34601;34602;34603;34654;34655;34656;34657;34658;34659;34660;34661;34662;34663;34664;34665;34666;34667;34668;34669;34670;34671;34672;34673;34674;34675;34676;34677;34678;34679;34680;34681;34682;34683;34684;34685;34804;34805;34806;34807;34808;34809;34810;34811;34812;34813;34814;34815;34816;34817;34818;34819;34820;34821;34822;34823;34824;34825;34826;34827;34869;34870;34871;34872;34873;34874;34875;34876;34877;34878;34879;34880;34881;34882;34883;34884;34885;34886;34887;34888;34889;34890;34891;34892;34893;34894;34895;34896;34897;34898;34899;34900;34901;34902;34903;34904;34905;34906;34907;34908;34909;34910;34911;34912;34913;34914;34915;34916;34917;34918;34919;34920;34921;34922;34923;34924;34925;34926;34927;34928;34929;34930;34931;34932;34933;34934;34935;34936;34937;34938;34939;34940;34941;34942;34943;34944;34945;34946;34947;34948;34949;34950;34951;34952;34953;34954;34955;34956;34957;34958;34959;34960;34961;34962;34963;34964;34965;34966;35060;35061;35062;35063;35064;35065;35066;35067;35068;35069;35070;35071;35072;35073;35074;35075;35076;35077;35078;35079;35080;35081;35164;35165;35166;35167;35168;35169;35995;35996;35997;35998;35999;36000;36001;36002;36003;36004;36005;36006;36007;36008;36009;36010;36011;36012;36013;36014;36015;36016;36017;36018;36019;36020;36491;36492;36668;36669;36670;36671;36672;36673;36700;36701;36702;36703;36704;36705;36706;36707;36708;36709;36710;36711;36712;36714;36800;36801;36802;36803;36804;36805;36806;36807;36808;36809;36810;36811;37018;37019;37020;37021;37022;37023;37024;37025;37026;37027;37054;37055;37056;37057;37058;37059;37060;37061;37062;37063;37064;37065;37066;37067;37068;37069;37070;37071;37072;37073;37074;37075;37076;37077;37078;37079;37080;37081;37082;37083;37084;37085;37086;37087;37088;37089;37090;37091;37092;37093;37094;37095;37096;37097;37098;37099;37100;37101;37102;37103;37104;37105;37106;37107;37108;37109;37110;37111;37112;37113;37114;37115;37116;37117;37118;37119;37120;37121;37122;37123;37340;37341;37342;37343;37344;37345;37346;37347;37348;37349;37350;37351;37352;37353;37354;37355;37356;37357;37358;37359;37360;37361;37362;37363;37364;37365;37366;37367;37368;37369;37370;37371;37372;37373;37376;37377;37378;37379;37380;37381;37382;37383;37384;37385;37386;37387;37388;37389;37390;37391;37392;37393;37394;37395;37396;37397;37398;37399;37400;37401;37402;37403;37404;37405;37406;37407;37408;37409;37410;37681;37682;37683;37684;37685;37686;37687;37688;37689;37690;37691;37692;37693;37694;37695;37696;37697;37698;37866;37867;37868;37869;37870;37871;37872;37873;37874;37875;37876;37877;37878;37879;37880;37881;37882;37883;37884;37885;37886;37887;37888;37889;37890;37891;37892;37893;37894;37895;37896;37897;37898;37899;37900;37901;37902;37903;37904;37905;37906;37907;37908;37909;37910;37911;37912;37913;37914;37915;38586;38587;38588;38589;38590;38591;38592;38593;38594;38595;38596;38597;38598;38599;38600;38601;38602;38603;38604;38605;38606;38607;38608;38609;38610;38611;38612;38613;38614;38615;38616;39408;39409;39410;39411;39412;39413;39414;39415;39416;39417;39418;39419;39420;39421;39422;39423;39424;39425;39426;39427;39428;39429;39430;39431;39432;39433;39434;39435;39436;39437;39438;39439;39440;39554;39555;39556;39557;39558;39559;39560;39561;39562;39563;39564;39565;39566;39567;39568;39569;39570;39571;39572;39573;39574;39575;39576;39577;39578;39579;39580;39611;39612;39613;39614;39615;39616;39617;39618;39619;39620;39621;39622;39623;39624;39625;39626;39627;39628;39629;39630;39631;39632;39633;39634;39635;39636;39637;39638;39639;39640;39641;39642;39643;39644;39645;39646;39647;39648;39649;39650;39651;39652;39653;39654;39655;39656;39657;39658;39659;39660;39661;39662;39663;39664;39665;39666;39667;39668;39669;39670;39680;39681;39682;39683;39684;39685;39686;39687;39688;39689;39690;39691;39692;39693;39694;39695;39696;39697;39698;39699;39700;39701;39702;39703;39704;39705;39706;39707;39708;39709;39710;39711;39712;39713;39714;39715;39716;39717;39718;39719;39720;39721;39722;39723;39724;39725;39726;39727;39728;39729;39730;39731;39732;39733;39734;39735;39736;39737;39738;39739;39740;39741;39742;39743;39744;39745;39746;39775;39776;39777;39778;39779;39780;39781;39782;39783;39784;39785;39786;39787;39788;39789;39790;39791;39792;39793;39794;39795;39796;39797;39798;39799;39800;39801;39802;39803;39804;39814;39815;39816;39817;39818;39819;39820;39821;39822;39823;39824;39825;39826;39827;39828;39829;39830;39831;39832;39833;39834;39835;39836;39837;39838;39839;39840;39841;39842;39843;39844;39845;39846;39847;39848;39849;39850;39851;39858;39859;39860;39861;39862;39863;39864;39865;39866;39867;39868;39869;39870;39871;39872;39873;39874;39875;39876;39877;39878;39879;39880;39881;39882;39883;39884;39885;39886;39887;39888;39889;39890;39891;39892;39893;39894;39895;39896;39897;39898;39899;39900;39901;39902;39903;39904;39905;39906;39907;39908;39909;39910;39911;39912;39913;39914;39915;39916;39917;39918;39919;39920;39921;39922;39923;39924;39925;39926;39927;39928;39929;39930;39931;39932;39933;39934;39935;39936;39937;39938;39939;39940;39941;39942;39943;39944;39945;39946;39947;39948;39949;39950;39951;39952;39953;39954;39955;39956;39957;39958;39959;39960;39961;39962;39963;39964;39965;39966;39967;39968;39969;39970;39971;39972;39973;39974;39975;39976;39977;39978;39979;39980;39981;39982;39983;39984;39985;39986;39987;39988;39989;39990;39991;39992;39993;39994;39995;39996;39997;39998;39999;40000;40001;40002;40003;40004;40005;40006;40007;40008;40009;40010;40011;40012;40013;40014;40015;40016;40017;40018;40019;40020;40021;40022;40023;40024;40025;40026;40027;40028;40029;40030;40031;40032;40033;40034;40035;40036;40037;40038;40039;40040;40041;40042;40043;40044;40045;40046;40047;40048;40049;40050;40051;40052;40053;40054;40055;40056;40057;40058;40059;40060;40061;40062;40063;40064;40065;40066;40067;40068;40069;40070;40071;40072;40073;40074;40075;40076;40077;40078;40079;40080;40081;40082;40083;40084;40085;40086;40087;40088;40089;40090;40091;40092;40093;40094;40095;40096;40097;40098;40099;40100;40101;40171;40172;40173;40174;40175;40176;40177;40178;40179;40180;40181;40182;40183;40184;40185;40186;40187;40188;40189;40190;40191;40192;40193;40194;40195;40196;40197;40198;40199;40200;40201;40202;40203;40204;40205;40206;40207;40208;40209;40211;40212;40213;40214;40215;40216;40217;40218;40219;40220;40221;40222;40223;40224;40225;40226;40227;40228;40229;40230;40231;40232;40233;40234;40235;40236;40237;40238;40239;40240;40241;40242;40243;40244;40245;40246;40247;40248;40249;40250;40251;40252;40253;40254;40255;40256;40257;40258;40259;40260;40261;40262;40263;40264;40265;40266;40267;40268;40269;40270;40271;40272;40273;40274;40275;40276;40277;40278;40279;40280;40281;40282;40283;40284;40285;40286;40287;40288;40289;40290;40291;40292;40293;40294;40295;40296;40297;40298;40299;40300;40301;40302;40303;40304;40305;40306;40307;40308;40309;40310;40311;40312;40313;40314;40315;40316;40317;40318;40319;40320;40321;40322;40323;40324;40325;40326;40327;40328;40329;40330;40331;40332;40333;40334;40335;40336;40337;40338;40369;40370;40371;40372;40373;40374;40375;40376;40377;40378;40379;40380;40381;40382;40383;40384;40385;40386;40387;40388;40389;40390;40391;40392;40393;40394;40395;40396;40397;40398;40399;40400;40401;40402;40403;40404;40405;40406;40407;40408;40409;40410;40411;40412;40413;40414;40415;40416;40417;40418;40419;40420;40421;40422;40423;40424;40425;40426;40427;40428;40429;40430;40431;40432;40433;40434;40435;40436;40437;40438;40439;40440;40441;40442;40443;40444;40445;40446;40447;40448;40449;40450;40451;40452;40453;40454;40455;40456;40457;40458;40459;40460;40461;40462;40463;40464;40465;40466;40467;40468;40469;40470;40471;40480;40481;40482;40483;40484;40485;40486;40487;40488;40489;40490;40491;40492;40493;40494;40495;40496;40497;40498;40499;40500;40501;40502;40503;40504;40505;40506;40507;40508;40509;40510;40511;40512;40513;40514;40515;40516;40517;40518;40519;40520;40521;40522;40523;40524;40525;40526;40527;40528;40529;40530;40531;40532;40533;40534;40535;40536;40537;40538;40539;40540;40541;40542;40543;40544;40545;40546;40547;40548;40549;40550;40551;40552;40553;40554;40555;40556;40557;40558;40559;40560;40594;40595;40596;40597;40598;40599;40600;40601;40602;40603;40604;40605;40606;40607;40608;40609;40610;40611;40612;40613;40614;40615;40616;40617;40618;40619;40620;40621;40622;40623;40624;40625;40626;40627;40628;40629;40630;40631;40632;40633;40634;40635;40636;40637;40638;40639;40640;40641;40642;40643;40644;40645;40646;40647;40648;40649;40654;40655;40656;40657;40658;40659;40660;40661;40662;40663;40664;40665;40666;40667;40668;40669;40670;40671;40672;40673;40674;40675;40676;40677;40678;40679;40680;40681;40682;40683;40684;40685;40686;40687;40688;40689;40690;40691;40692;40693;40694;40695;40696;40697;40698;40699;40700;40701;40702;40703;40704;40705;40706;40707;40708;40709;40710;40711;40712;40713;40714;40715;40716;40717;40718;40719;40720;40721;40722;40723;40724;40725;40726;40784;40785;40786;40787;40788;40789;40790;40791;40792;40793;40794;40795;40796;40797;40798;40799;40800;40801;40802;40803;40804;40805;40806;40807;40808;40809;40810;40811;40812;40813;40814;40815;40816;40817;40818;40819;40820;40821;40822;40823;40824;40825;40826;40827;40828;40829;40830;40831;40832;40833;40834;40835;40836;40837;40838;40839;40840;40841;40842;40843;40844;40845;40846;40847;40848;40849;40850;40851;40852;40853;40854;40855;40856;40857;40858;40859;40860;40861;40862;40863;40864;40865;40866;40867;40868;40869;40870;40871;40872;40873;40874;40875;40876;40877;40878;40879;40880;40881;40882;40883;40884;40885;40886;40887;40888;40889;40890;40891;40892;40893;40894;40895;40896;40897;40898;40899;40900;40901;40902;40903;40904;40905;40906;40907;40908;40909;41392;41393;41394;41395;41396;41397;41398;41399;41400;41401;41402;41403;41404;41405;41406;41407;41408;41409;41410;41411;41412;41413;41542;41543;41544;41545;41546;41547;41548;41549;41550;41551;41552;41553;41554;41555;41556;41557;41558;41559;41560;41561;41562;41563;41564;41565;41566;41567;41568;41569;42204;42205;46364;46365;46366;46367;46368;46369;46370;46371;46372;46373;46374;46375;46376;46377;46378;46379;46380;46381;46382;46383;46384;46385;46386;46387;46388;46389;46694;46695;46696;46697;46698;46699;46700;46701;46702;46703;46704;46705;46706;46707;46708;46709;46710;46711;46712;46713;46714;46715;46716;46717;46718;46719;46720;46721;46722;46723;46724;46725;46726;46727;46728;46729;46730;46731;46732;46733;46734;46735;46736;46737;46738;46739;46740;46741;46742;46743;46744;46745;46746;46747;46748;46749;46750;46751;46752;46753;46754;46755;46756;46757;46758;46759;46760;46761;46762;46763;46764;46765;46766;46767;46768;46769;46770;46771;46772;46773;46774;46775;46776;46777;46778;46779;46780;46781;46782;46783;46784;46785;46786;46787;46788;46789;46790;46791;46792;46793;47955;47956;47957;47958;47959;47960;47961;47962;47963;47964;47965;47966;47967;47968;47969;47970;47971;47972;47973;47974;47975;47976;47977;47978;47979;47980;47981;47982;47983;47984;47985;47986;47987;47988;47989;47990;47991;47992;47993;47994;47995;47996;47997;47998;47999;48000;48001;48002;48653;48654;48655;48656;48657;48658;48659;48660;48661;48662;48663;48664;48665;48666;48667;48668;48669;48670;48671;48672;48673;48674;48675;48676;48677;48678;48679;48680;48681;48712;48713;48714;48715;48716;48717;48718;48719;48720;48721;48722;48723;48724;48725;48726;48727;48728;48729;48730;48731;48732;48733;48734;48735;48736;48737;48738;48739;48740;48741;48742;48743;48744;48745;48746;48747;48748;48749;48750;48751;48752;48753;48754;48755;48756;48757;48758;48759;48760;48761;48762;48763;48764;48765;48766;48767;48768;48769;48770;48771;48772;48773;48774;48775;48776;48777;48778;48779;48780;48781;48782;48783;48784;48785;48786;48787;48788;48789;48790;48791;48792;48793;48794;48795;48796;48797;48798;48799;48800;48801;48802;48803;48804;48805;48806;48807;48808;48809;48810;48811;48812;48813;48814;48815;48816;48817;48818;48819;48820;48821;48822;48823;48824;48825;48826;48827;48828;48829;48830;48831;48832;48833;48834;48835;48836;48837;48838;48839;48840;48841;48842;48843;48844;48845;48846;48847;48848;48849;48850;48851;48852;48853;48854;48855;48856;48857;48858;48859;48860;48861;48862;48863;48864;48865;48866;48867;48868;48869;48870;48871;48872;48873;48874;48875;48876;48877;48878;48879;48880;48881;48882;48883;48884;48885;48886;48887;48888;48889;48890;48891;48892;48893;48894;48895;48896;48897;48898;48899;48900;48901;48902;48903;48904;48905;48906;48907;48908;48909;48910;48911;48912;48913;48914;48915;48916;48917;48918;48919;48920;48921;48922;48923;48924;48925;48926;48927;48928;48929;48930;48931;48932;48933;48934;48935;48936;48937;48938;48939;48940;48941;48942;48943;48944;48945;48946;48947;48948;48949;48950;48951;48952;48953;48954;48955;48956;48957;48958;48959;48960;48961;48962;48963;48964;48965;48966;48967;48968;48969;48970;48971;48972;48973;48974;48975;48976;48977;48978;48979;48980;48981;48982;48983;48984;48985;48986;48987;48988;48989;48990;48991;48992;48993;48994;48995;48996;48997;48998;48999;49000;49001;49002;49003;49004;49005;49006;49007;49008;49009;49010;49011;49012;49013;49014;49015;49016;49017;49018;49019;49020;49021;49022;49023;49024;49025;49026;49027;49028;49029;49030;49031;49032;49033;49034;49035;49036;49037;49038;49039;49040;49041;49042;49043;49044;49045;49046;49047;49048;49049;49050;49051;49052;49053;49054;49055;49056;49057;49058;49059;49060;49061;49062;49063;49064;49065;49066;49067;49068;49069;49070;49071;49072;49073;49074;49075;49076;49077;49078;49079;49080;49081;49082;49083;49084;49085;49086;49087;49088;49089;49090;49091;49092;49093;49094;49095;49096;49097;49098;49099;49100;49101;49102;49103;49104;49105;49106;49107;49108;49109;49110;49111;49112;49113;49114;49115;49116;49117;49118;49119;49120;49121;49122;49123;49124;49125;49126;49127;49128;49129;49130;49131;49132;49133;49134;49135;49136;49137;49138;49139;49140;49141;49142;49143;49144;49145;49146;49147;49148;49149;49150;49151;49152;49153;49154;49155;49156;49157;49158;49159;49160;49161;49162;49163;49164;49165;49166;49167;49168;49169;49170;49171;49172;49173;49174;49175;49176;49177;49178;49179;49180;49181;49182;49183;49184;49185;49186;49187;49188;49189;49190;49191;49192;49193;49194;49195;49196;49197;49198;49199;49200;49201;49202;49203;49204;49205;49206;49207;49208;49209;49210;49211;49212;49213;49214;49215;49216;49217;49218;49219;49220;49221;49222;49223;49224;49225;49226;49227;49228;49229;49230;49231;49232;49233;49234;49235;49236;49237;49238;49239;49240;49241;49242;49243;49244;49245;49246;49247;49248;49249;49250;49251;49252;49253;49254;49255;49256;49257;49258;49259;49260;49261;49262;49263;49264;49265;49266;49267;49268;49269;49270;49271;49272;49273;49274;49275;49276;49277;49278;49279;49280;49281;49282;49283;49284;49285;49286;49287;49288;49289;49290;49291;49292;49293;49294;49295;49296;49297;49298;49299;49300;49301;49302;49303;49304;49305;49306;49307;49308;49309;49310;49311;49312;49313;49314;49315;49316;49317;49318;49319;49320;49321;49322;49323;49324;49325;49326;49327;49328;49329;49330;49331;49332;49333;49334;49335;49336;49337;49338;49339;49340;49341;49342;49343;49344;49345;49346;49347;49348;49349;49350;49351;49352;49353;49354;49355;49356;49357;49358;49359;49360;49361;49362;49363;49364;49365;49366;49367;49368;49369;49370;49371;49372;49373;49374;49375;49376;49377;49378;49379;49380;49381;49382;49383;49384;49385;49386;49387;49388;49389;49390;49391;49392;49393;49394;49395;49396;49397;49398;49399;49400;49401;49402;49403;49404;49405;49406;49407;49408;49409;49410;49411;49412;49413;49414;49415;49416;49417;49418;49419;49420;49421;49422;49423;49424;49425;49426;49427;49428;49429;49430;49431;49432;49433;49434;49435;49436;49437;49438;49439;49440;49441;49442;49443;49444;49445;49446;49447;49448;49449;49450;49451;49452;49453;49454;49455;49456;49457;49458;49459;49460;49461;49462;49463;49464;49465;49466;49467;49468;52965;52966;52967;52968;52969;52970;52971;52972;52973;52974;52975;52976;52977;52978;52979;52980;52981;52982;52983;52984;52985;52986;52987;52988;52989;52990;52991;52992;52993;52994;52995;52996;52997;52998;52999;53000;53001;53002;53003;53223;53224;53225;53226;53227;53228;53229;53230;53231;53232;55505;55506;55507;55508;55509;57053;57054;57055;57056;57057;57058;57059;57060;57061;57062;57063;57064;57065;57066;57067;57068;57069;57070;57071;57072;57073;57074;57075;57076;57077;57078;57079;57080;57081;57082;57083;57084;57085;57086;57087;57088;57089;58511;58512;58513;58514;58515;58516;58517;58518;58519;58520;58521;58522;58523;58524;58525;58526;58527;58528;58529;58530;58531;58532;58533;58534;58535;58536;58537;58538;58539;58540;58541;58542;58543;58544;58545;58546;58547;58548;58549;58814;58815;58816;58817;58818;59925;59926;59927;59928;59929;59930;59931;59932;59933;59934;59935;59936;59937;61101;61102;61103;61104;61105;61106;61107;62683;62684;62685;62686;62687;62688;62689;62690;62691;62692;62693;62694;62695;62696;62697;62698;62699;62700;62701;62702;62703;62704;62705;62706;62707;62708;62709;62710;62711;62712;62713;62714;62715;62716;62717;62718;62719;62720;62721;62722;62723;62724;62725;62726;62727;62728;62729;62730;62731;62732;62733;62734;62735;62736;62737;62738;62739;63147;63148;63149;63150;63151;63152;63153;63154;63155;63156;63157;63891;63892;63893;63894;63895;63896;63897;63898;63899;63900;63901;63902;63903;64675;64676;64677;64678;64679;64680;64719;64720;64721;64722;64723;64724;64725;64726;64727;64728;64729;64730;64731;64732;64733;64734;64735;64736;64737;64738;64739;64740;64741;64742;64743;64744;64745;64746;64747;64748;64749;64750;64751;64752;64753;64754;64755;64756;64757;64758;64759;64760;64761;64762;64763;64764;64765;64766;64767;64768;64769;64770;64771;64772;64773;64774;64775;64776;64777;64778;64779;64780;64781;64782;64783;64784;64785;64786;64787;64788;64789;64790;64791;64792;64793;64794;64795;64796;64797;64798;64799;64800;64801;64802;64808;64809;64810;64811;64812;64813;64814;64815;64816;64817;64818;64819;64820;64821;64822;64823;64824;64825;64826;64827;64828;64829;64830;64831;64832;64833;64834;64835;64836;64837;64838;64839;64840;64841;64842;64843;64844;64909;64910;64911;64912;64913;64914;64915;64916;64917;64918;64919;64920;64921;64922;64923;64924;64925;64926;64927;64928;64929;64930;64931;64932;64933;64934;64935;64936;64937;64938;64939;64940;64941;64942;64943;64944;64945;65649;65650;65651;65652;65653;65654;65655;65656;65657;65658;65659;65660;65661;65662;65663;65664;65665;65666;65667;65668;65669;65670;65671;65672;65673;65674;65675;65676;65677;65678;65679;65680;65681;65682;65683;65684;65685;65686;65687;65688;65689;65690;65691;65692;65693;65694;65695;65696;65697;65698;65699;65700;66181;66182;66183;66184;66185;66186;66187;66188;66189;66190;66191;66192;66193;66194;66195;66196;66197;66198;66199;66200;66201;66202;66203;66204;66205;66206;66207;66208;66209;66210;66211;66212;66213;66214;73550;73551;73552;73553;73554;73555;73556;73557;73558;73559;73560;73561;73562;73563;73564;79803;79804;79805;79806;79807;79808;79809;79810;79811;79812;79813;79814;79815;79816;79817;79818;79819;79820;79821;79822;79823;79824;79825;79826;79827;79828;79829;79830;79831;79832;79833;79834;79835;79836;82573;82574;82575;82576;82577;82578;82579;82580;82581;82582;82583;82584;82585;82586;82587;82588;82589;82590;82591;82592;82593;82594;82595;82596;82597;82598;82599;82600;82601;82602;82603;86988;86989;86990;86991;86992;86993;86994;86995;86996;86997;86998;86999;87000;87001;87002;87003;87004;87005;87006;87007;87008;87009;87010;87011;87012;87013;87014;87015;87016;87017;87018;87019;87020;87021;87022;87023;87024;87025;87026;87027;87028;87029;87030;87031;87032;87033;87034;87035;87036;87037;87038;87039;87040;87041;87042;87043;87044;87045;87046;87047;87048;87049;87050;87051;87052;87053;87054;87055;87056;87057;87058;87059;87060;87061;87062;87063;91795;91796;91797;91798;91799;91800;91801;91802;91803;91804;91805;91806;91807;91808;91809;91810;91811;91812;91813;91814;91815;91816;91817;91818;91819;95551;95552;95553;95554;95555;95556;95557;95558;95559;95560;95561;95562;95563;95564;95565;95566;95567;95568;95569;95570;95571;95572;95573;95574;95575;95576;95577;95578;95579;95580;95581;95582;95583;95662;95663;95664;95665;95666;95667;95668;95669;95836;95837;95838;95839;95840;95841;95842;95843;95844;95845;95846;95847;95848;95849;95850;95851;95852;95853;95854;95855;95856;95857;95878;95879;95880;95881;95882;95883;95884;95885;95886;95887;95888;95889;95890;95891;95892;95893;95894;95895;95896;95897;95898;95899;95900;95901;95902;95903;95904;95905;95906;95907;95908;95909;95910;95911;95912;95913;95914;95915;95916;95917;95918;95919;95920;95921;95922;95923;95924;95925;95926;95927;95928;95929;95930;95931;95932;95933;95998;95999;96000;96001;96002;96003;96004;96005;96006;96007;96008;96009;96010;96011;96012;96013;96014;96015;96016;96017;96018;96019;96020;96021;96022;96023;96024;96025;96026;96027;96028;96029;96030;96031;96032;96033;96034;96035;96036;96037;96038;96039;96040;96041;96042;96043;96044;96045;96046;96047;96048;96049;96050;96051;96052;96053;96123;96124;96125;96126;96127;96128;96129;96130;96131;96132;96133;96134;96135;96136;96137;96138;96139;96140;98283;100744;100745;100746;100747;100748;100749;100750;100751;100752;100753;100754;100755;100756;100757;100758;100759;100760;100761;100762;100763;100764;100765;100766;100767;100768;100769;100770;100771;100772;100773;100774;100775;100776;100777;100778;100779;100780;100781;100782;100783;100784;100785;100786;100787;100788;100789;100790;100791;100792;100793;100794;100795;100796;100797;100798;100799;100800;100801;100802;100803;100804;100805;100806;100807;100808;100809;100810;100811;100912;100913;100914;100915;100916;100917;100918;100919;100920;100921;100922;100923;100924;100925;100926;100927;100928;100929;100930;100931;100932;100933;100970;100971;100972;100973;100974;100975;100976;100977;100978;100979;100980;100981;100982;100983;100984;100985;100986;100987;100988;100989;100990;100991;101015;101016;101017;101069;101070;101071;101072;101073;101074;101075;101076;101077;101078;101229;101230;101231;101232;102230;102231;102232;102233;102234;102235;102236;102237;102238;102239;102240;102241;102242;102243;102244;102245;102246;102247;102248;102249;102250;102251;102252;102253;102254;102255;102256;102257;102258;102259;102260;102261;102262;102263;102264;102265;102266;102267;103153;103154;103155;103156;103157;103158;103159;103160;103161;103162;103163;103164;103165;103166;103167;103168;103169;103170;103171;103172;103173;103174;103175;103176;103177;103178;103179;103180;103181;103182;103183;103184;103185;103186;103187;103188;103189;103190;103191;103192;103193;103194;103195;103196;103197;103198;103199;103200;103201;103202;103203;103204;103205;103206;103207;103208;103209;103210;103211;103212;103213;103214;103215;103216;103217;103218;103219;103220;103221;103222;103223;103224;103225;103226;103227;103228;103229;103230;103231;103232;103233;103234;103235;103236;103237;103238;103239;103240;103241;103242;103243;103244;103245;103246;103247;103248;103249;103250;103251;103252;103253;103254;103255;103256;103257;103258;103259;103260;103261;103262;103263;103264;103265;103266;103267;103268;103269;103270;103271;103272;103273;103274;103275;103276;103277;103278;103279;103280;103281;103282;103283;103284;103285;103286;103287;103288;103289;103290;103291;103292;103293;103294;103295;103296;103297;103298;103299;103300;103301;103302;103303;103304;103305;103306;103307;103308;103309;103310;103311;103312;103313;103314;103315;103316;103317;103318;103319;103320;103321;103322;103323;103324;103325;103326;103327;103328;103329;103330;103331;103332;103333;103334;103335;103336;103337;103338;103339;103340;103341;103342;103343;103344;103345;103346;103347;103348;103349;103350;103351;103352;103353;103354;103355;103356;103357;103358;103359;103360;103361;103362;103363;103364;103365;103366;103367;103368;103369;103370;103371;103372;103373;103374;103375;103376;103377;103378;103379;103380;103381;103382;103383;103384;103385;103386;103387;103388;103389;103390;103391;103392;103393;103394;103395;103396;103397;103398;103399;103400;103401;103402;103403;103404;103405;103406;103407;103408;103409;103410;103411;103412;103413;103414;103415;103416;103417;103418;103419;103420;103421;103422;103423;103424;103425;103426;103427;103428;103429;103430;103431;103432;103433;103434;103435;103436;103437;103438;103439;103440;103441;103442;103443;103444;103445;103446;103447;103448;103449;103450;103451;103452;103453;103454;103455;103456;103457;103458;103459;103460;103461;103462;103463;103464;103465;103466;103467;103468;103469;103470;103471;103472;103473;103474;103475;103476;103477;103478;103479;103480;103481;103482;103483;103484;103485;103486;103487;103488;103489;103490;103491;103492;103493;103494;103495;103496;103497;103498;103499;103500;103501;103502;103503;103504;103505;103506;103507;103508;103509;103510;103511;103512;103513;103514;103515;103516;103517;103518;103519;103520;103521;103522;103523;103524;103525;103526;103527;103528;103529;103530;103531;103532;103533;103534;103535;103536;103537;103538;103539;103540;103541;103542;103543;103544;103545;103546;103547;103548;103549;103550;103551;103552;103553;103554;103555;103556;103557;103558;103559;103560;103561;103562;103563;103564;103565;103566;103567;103568;103569;103570;103571;103572;103573;103574;103575;103576;103577;103578;103579;103580;103581;103582;103583;103584;103585;103586;103587;103588;103589;103590;103591;103592;103593;103594;103595;103596;103597;103598;103599;103600;103601;103602;103603;103604;103605;103606;103607;103608;103609;103610;103611;103612;103613;103614;103615;103616;103617;103618;103619;103620;103621;103622;103623;103624;103625;103626;103627;103628;103629;103630;103631;103632;103633;103634;103635;103636;103637;103638;103639;103640;103641;103642;105235;105236;105237;105238;105239;105240;105241;105242;105243;105244;105245;105246;105247;105248;105249;105250;105251;105252;105253;105254;105255;105256;105257;105258;105259;105260;105261;105262;105263;105264;105265;105266;105267;105268;105269;105270;105271;105272;105273;105274;105275;105276;105277;105278;105279;105280;105281;105282;105283;105284;105285;105286;105542;105543;105544;105545;105546;105547;105548;105549;105550;105551;105552;105553;105554;105555;105556;105557;105558;105559;105560;105561;105562;105563;105564;105565;105566;105567;105568;105569;105570;105571;105572;105573;105574;105575;105576;105577;105578;105579;105580;105581;105582;105583;105584;105585;105586;105587;105588;105589;105590;105591;105592;105593;105594;105595;105596;105597;105598;105599;105600;105601;105602;105603;105604;105605;105606;105607;105608;105609;105610;105611;105612;105613;105614;105615;105616;105617;105618;105619;105620;105621;105622;105623;105624;105625;105626;105627;105628;105629;105630;105631;105632;105633;105634;105635;105636;105637;105638;105639;105640;105641;105642;105643;105644;105645;105646;105647;105648;105649;105650;105651;105652;105653;105654;105655;105656;105657;105658;105659;105660;105661;105662;105663;105664;105665;105666;105667;105697;105698;105699;105700;105701;105702;105703;105704;105705;105706;105707;105708;105709;105710;105711;105712;105713;106361;106362;106363;106364;106365;106366;106367;106368;106369;106370;106371;106372;106373;106374;106375;106376;106377;106378;106379;106380;106381;106382;106383;106384;106385;106386;106387;106388;106389;106390;106391;106392;106393;106394;106395;106396;106397;106398;106399;106400;106401;106402;106403;106404;106405;106406;106407;106408;106451;106452;106453;106454;106455;106456;106457;106458;106459;106460;106461;106462;106463;106464;106465;106466;106467;106468;106469;106470;106471;106472;106473;106474;106739;106740;106741;106742;106743;106744;106745;106746;106747;106748;106749;106750;106751;106752;106753;106754;106755;106756;106757;106758;106759;106760;106761;106762;106763;106764;106765;106766;106767;106768;106769;106770;106771;106772;106773;106774;106775;106776;107494;107495;107496;107497;107498;107499;107500;107501;107502;107503;107504;107505;107506;107507;107508;107509;107510;107511;107512;107513;107514;107515;107516;107517;107518;107519;107692;107693;107694;107695;107696;107697;107698;107699;107700;107701;107702;107703;107704;107705;107706;107707;107708;107709;107710;107711;107712;107713;107966;107967;107968;107969;107970;107971;107972;107973;107974;107975;107976;107977;107978;107979;107980;107981;107982;107983;107984;107985;107986;107987;107988;107989;107990;107991;107992;107993;107994;107995;107996;107997;108010;108011;108012;108013;108014;108015;108016;108017;108018;108019;108020;108021;108022;108023;108024;108025;108026;108027;108028;108029;108030;108031;108032;108033;108034;108035;108036;108037;108038;108039;108040;108041;108042;108043;108044;108045;108046;108047;108048;108049;108050;108051;108052;108053;108054;108055;108056;108057;108058;108059;108060;108061;108062;108063;108064;108065;108066;108067;108234;108235;108236;108237;108238;108239;108240;108241;108242;108243;108244;108245;108246;108247;108248;108249;108250;108251;108252;108253;108254;108255;108256;108257;108258;108259;108260;108261;108262;108263;108264;108265;108266;108267;108268;108269;108270;108271;108272;108273;108274;108275;108276;108277;108278;108455;108456;108457;108458;108459;108460;108461;108462;108463;108464;108465;108466;108467;108468;108469;108470;108471;108472;108473;108474;108475;108476;108477;108478;108479;108480;108481;108482;108483;108484;108485;108486;108487;108488;108489;108490;108491;108575;108576;108577;108578;108579;108580;108581;108582;108583;108584;108585;108586;108587;108588;108589;108590;108591;108592;108593;108594;108595;108596;108597;108598;108599;108600;108601;108602;108603;108604;108605;108606;108607;108608;108609;108610;108611;108612;108613;108614;108615;108616;108617;108618;109226;109227;109228;109229;109230;109231;109232;109233;109234;109235;109236;109237;109238;109239;109240;109241;109242;109243;109244;109245;109246;109247;109248;109249;109250;109251;109252;109253;109254;109255;109256;109257;109258;109259;109260;109261;109262;109263;109264;109265;109266;109267;109268;109269;109270;109271;109272;109273;109274;109275;109276;109277;109278;109279;109280;109281;109282;109283;109284;109285;109286;109287 998;999;1000;1001;1002;1003;1004;1005;3201;3414;3415;3416;3417;3418;3419;3420;3421;3422;3423;3424;3425;3426;3427;3428;3429;3430;3431;3432;3433;3434;3435;3436;3437;3438;3649;3650;3779;3780;3781;3782;3783;3784;3785;3786;4381;4382;4383;4384;4385;4386;4387;4388;4389;4390;4391;4392;4393;4394;4395;4396;4397;4398;4399;4400;4401;4402;4403;4404;4405;4406;4407;4408;4409;4410;4411;4412;4413;4414;4415;4416;4417;4418;4419;4420;4421;4422;4423;4424;4425;4426;4427;4428;4429;4430;4431;4432;4433;4434;4435;4436;4437;4438;4439;4440;4441;4442;4443;4444;4445;4446;4447;4448;4449;4450;4451;4452;4453;4454;4455;4456;4457;4458;4459;4460;4461;4462;4463;4464;4465;4466;4467;4468;4469;4470;4471;4472;4473;4474;4475;4476;4477;4478;4479;4480;4481;4482;4483;4484;4485;4486;4487;4488;4489;4490;4491;4492;4493;4494;4495;4496;4497;4498;4499;4500;4501;4502;4503;4504;4505;4506;4507;4508;4933;4934;4935;4936;4937;4938;4939;4940;4941;4942;4943;4944;4945;4946;4947;4948;4949;4950;4951;4952;4953;4954;4955;4956;4957;4958;4959;4960;4961;4962;4963;4964;4965;4966;4967;4968;4969;4970;4971;4972;4973;4974;4975;4976;4977;4978;4979;4980;4981;4982;4983;4984;4985;4986;4987;4988;4989;4990;4991;4992;4993;4994;5147;7950;7951;7952;7953;7954;7955;7956;7957;7958;7959;7960;7961;7962;7963;7964;7965;7966;7967;7968;7969;7970;7971;7972;7973;7974;7975;7976;7977;7978;7979;7980;7981;7982;7983;7984;7985;7986;7987;7988;7989;7990;7991;7992;7993;7994;7995;7996;7997;7998;7999;8000;8001;8002;8003;8004;8005;8006;8007;8008;8009;8010;8011;8012;8013;8014;8015;8016;8017;8018;8019;8020;8021;8022;8023;8024;8025;8026;8027;8028;8029;11527;11528;11529;11530;11531;11532;11533;11534;11535;11536;11537;11538;11539;11540;11541;11542;11543;11544;11545;11546;11547;11548;11549;11550;11551;11552;11553;11554;11555;11556;11557;11558;11559;11560;11561;11562;11563;11564;11565;11566;11567;11568;11569;11570;11571;11572;11573;11574;11575;11576;11577;11578;11579;11580;11581;11582;11583;11584;11585;11586;11587;11588;11589;11590;11591;11592;11593;11594;11595;11596;11597;11598;11599;11600;11601;11602;11603;11604;11605;11606;11607;11608;11609;11610;11611;11612;11613;12042;12043;12044;12045;12046;12047;12048;12049;12050;12051;12052;12053;12054;12382;12383;12384;12385;12386;12387;12388;12389;12390;12391;12392;12393;12394;12395;12396;12397;12398;12399;12400;12401;12402;12403;12404;12405;12406;12407;12408;12409;12410;12411;12412;12413;12414;12415;12416;12417;12418;12419;12420;12421;12422;12423;12424;12425;12426;12427;12428;12429;12430;12431;12432;12433;12434;12435;12436;12437;12438;12439;12440;12441;12442;12443;12444;12445;12446;12447;12448;12449;12450;12451;12452;12453;12454;12455;12456;12457;12458;12459;12460;12461;12462;12463;12464;12465;12466;12467;12468;12469;12470;12471;12472;12473;12474;12475;12476;12477;12478;12479;12480;12481;25066;25067;25068;25069;25070;25071;25072;25073;25074;25075;25076;25077;25078;25079;25080;25081;25082;25083;25084;25085;25086;25087;25088;25089;25090;25091;25092;25093;25094;25095;25096;25097;25098;25099;25100;25101;25102;25103;25104;25105;25106;25107;25108;25109;25110;25111;25112;25113;25114;25115;25116;25117;25118;25119;25120;25121;25122;25123;25124;25125;25126;25127;25128;25129;25130;25131;25132;25133;25134;25135;25136;25137;46310;46561;46562;46563;46564;46565;46566;46567;46568;46569;46570;46571;46572;46573;46574;46575;46576;46577;46578;46579;46580;46637;46638;46639;46640;46641;46642;46643;46644;46645;46646;46647;46648;46649;46650;46651;46652;46653;46654;46655;46656;46657;46658;46659;46660;46661;46662;46663;46664;46665;46666;46667;46668;46669;46670;46671;46672;46673;46920;46921;46922;47009;47010;47011;47012;47013;47014;47015;47016;47017;47018;47019;47020;47021;47022;47023;47024;47025;47026;47027;47028;47029;47030;47031;47032;47033;47034;47035;47036;47037;47038;47039;47040;47041;47042;47043;47044;47045;47046;47047;47048;47049;47050;47051;47052;47053;47054;47055;47056;47057;47058;47059;47060;47061;47062;47063;47064;47065;47066;47067;47068;47069;47070;47071;47072;47073;47074;47075;47076;47077;47078;47079;47080;47081;47082;47083;47084;47085;47086;47087;47088;47089;47090;47091;47092;47093;47094;47095;47096;47097;47098;47099;47100;47101;47102;47103;47104;47105;47106;47107;47686;47687;47688;47689;47690;47691;47692;47693;47694;47695;47696;47697;47698;47699;47700;47701;47702;47703;47704;47705;47706;47707;47708;47709;47710;47711;47712;47713;47714;48448;48449;48450;48451;48452;48453;48454;48455;48456;48457;48458;48459;48460;48461;48462;48463;48464;48465;48466;48467;48468;48469;48470;48471;48472;48473;48474;48475;48476;48545;48546;48547;48548;48549;48550;48551;48552;48553;48554;48555;48556;48557;48558;48559;48560;48561;48562;48563;48564;48565;48566;48567;48568;48569;48570;48571;48572;48573;48574;48575;48576;48577;48578;48579;48580;48581;48582;48583;48584;48585;48586;48587;48588;48589;48590;48591;48592;48593;48594;48595;48596;48597;48598;48599;48600;48601;48602;48603;48818;48819;48820;48821;48822;48823;48824;48825;48826;48827;48828;48829;48830;48831;48832;48833;48834;48835;48836;48837;48838;48839;48840;48841;48842;48843;48844;48845;48846;48847;48848;48849;48850;48851;48852;48853;48854;48855;48856;48857;48858;48859;48860;48861;48862;48863;48864;48865;48866;48867;48868;48869;48870;48871;48872;48873;48874;48875;48876;48877;48878;48972;48973;48974;48975;48976;48977;48978;48979;48980;48981;48982;48983;48984;48985;48986;48987;48988;48989;48990;48991;48992;48993;48994;48995;48996;48997;48998;48999;49000;49001;49002;49003;49004;49005;49006;49007;49008;49009;49010;49011;49012;49013;49014;49015;49016;49017;49018;49019;49020;49021;49022;49023;49024;49025;49026;49027;49028;49029;49030;49031;49032;49033;49034;49035;49036;49037;49038;49039;49040;49041;49042;49043;49044;49045;49046;49047;49048;49049;49050;49051;49052;49053;49054;49055;49056;49057;49058;49059;49060;49061;49062;49063;49064;49065;49066;49067;49068;49069;49070;49071;49072;49073;49074;49075;49076;49077;49078;49079;49080;49081;49082;49083;49084;49085;49086;49087;49088;49089;49090;49091;49092;49093;49094;49095;49096;49097;49098;49099;49100;49101;49102;49103;49104;49105;49106;49107;49108;49109;49110;49111;49112;49113;49114;49115;49116;49117;49118;49119;49120;49121;49122;49123;49124;49125;49126;49127;49128;49129;49130;49131;49132;49133;49134;49135;49136;49137;49138;49139;49140;49141;49142;49143;49144;49145;49146;49147;49148;49149;49273;49274;49275;49276;49277;49278;49279;49280;49281;49282;49283;49284;49285;49286;49287;49288;49289;49389;49390;49391;49392;49393;49394;49395;50753;50754;50755;50756;50757;50758;50759;50760;50761;50762;50763;50764;50765;50766;50767;50768;50769;50770;50771;50772;50773;50774;50775;50776;50777;50778;50779;50780;50781;50782;50783;50784;50785;50786;50787;50788;50789;50790;50791;50792;50793;50794;50795;50796;50797;50798;50799;50800;50801;50802;50803;50804;50805;50806;50807;50808;50809;50810;50811;50812;50813;50814;50815;50816;50817;50818;50819;50820;50821;50822;50823;50824;51488;51489;51730;51731;51732;51733;51734;51735;51736;51757;51758;51759;51760;51761;51762;51763;51764;51765;51766;51767;51768;51769;51770;51771;51773;51883;51884;51885;51886;51887;51888;51889;51890;51891;51892;51893;51894;51895;51896;51897;51898;51899;52203;52204;52205;52206;52207;52208;52209;52210;52211;52212;52263;52264;52265;52266;52267;52268;52269;52270;52271;52272;52273;52274;52275;52276;52277;52278;52279;52280;52281;52282;52283;52284;52285;52286;52287;52288;52289;52290;52291;52292;52293;52294;52295;52296;52297;52298;52299;52300;52301;52302;52303;52304;52305;52306;52307;52308;52309;52310;52311;52312;52313;52314;52315;52316;52317;52318;52319;52320;52321;52322;52323;52324;52325;52326;52327;52328;52329;52330;52331;52332;52333;52334;52335;52336;52337;52338;52339;52340;52341;52342;52343;52344;52345;52346;52347;52348;52349;52350;52351;52352;52353;52354;52355;52356;52357;52358;52359;52360;52361;52362;52363;52364;52365;52366;52367;52368;52369;52370;52371;52372;52373;52374;52375;52376;52377;52378;52379;52380;52381;52382;52688;52689;52690;52691;52692;52693;52694;52695;52696;52697;52698;52699;52700;52701;52702;52703;52704;52705;52706;52707;52708;52709;52710;52711;52712;52713;52714;52715;52716;52717;52718;52719;52720;52721;52722;52723;52724;52725;52726;52727;52728;52729;52730;52731;52732;52733;52734;52735;52736;52737;52738;52739;52740;52741;52742;52743;52744;52745;52746;52747;52748;52749;52751;52752;52753;52754;52755;52756;52757;52758;52759;52760;52761;52762;52763;52764;52765;52766;52767;52768;52769;52770;52771;52772;52773;52774;52775;52776;52777;52778;52779;52780;52781;52782;52783;52784;52785;52786;52787;52788;52789;52790;52791;52792;52793;52794;52795;52796;52797;52798;52799;53211;53212;53213;53214;53215;53216;53217;53218;53219;53220;53221;53222;53223;53224;53225;53226;53227;53228;53229;53230;53231;53232;53233;53234;53235;53236;53237;53568;53569;53570;53571;53572;53573;53574;53575;53576;53577;53578;53579;53580;53581;53582;53583;53584;53585;53586;53587;53588;53589;53590;53591;53592;53593;53594;53595;53596;53597;53598;53599;53600;53601;53602;53603;53604;53605;53606;53607;53608;53609;53610;53611;53612;53613;53614;53615;53616;53617;53618;53619;53620;53621;53622;53623;53624;53625;53626;53627;53628;53629;53630;53631;53632;53633;53634;53635;53636;53637;53638;53639;53640;53641;53642;53643;53644;53645;53646;53647;53648;53649;53650;53651;53652;53653;53654;53655;53656;53657;53658;54629;54630;54631;54632;54633;54634;54635;54636;54637;54638;54639;54640;54641;54642;54643;54644;54645;54646;54647;54648;54649;54650;54651;54652;54653;54654;54655;54656;54657;54658;54659;54660;54661;54662;54663;54664;54665;54666;54667;54668;54669;54670;54671;54672;54673;54674;54675;54676;55774;55775;55776;55777;55778;55779;55780;55781;55782;55783;55784;55785;55786;55787;55788;55789;55790;55791;55792;55793;55794;55795;55796;55797;55798;55799;55800;55801;55802;55803;55804;55805;55806;55807;55808;55809;55810;55811;55812;55813;55814;55815;55816;55817;55818;55819;55820;56007;56008;56009;56010;56011;56012;56013;56014;56015;56016;56017;56018;56019;56020;56021;56022;56023;56024;56025;56026;56027;56028;56029;56030;56031;56032;56033;56034;56035;56036;56037;56038;56039;56040;56041;56042;56043;56044;56045;56046;56047;56048;56049;56093;56094;56095;56096;56097;56098;56099;56100;56101;56102;56103;56104;56105;56106;56107;56108;56109;56110;56111;56112;56113;56114;56115;56116;56117;56118;56119;56120;56121;56122;56123;56124;56125;56126;56127;56128;56129;56130;56131;56132;56133;56134;56135;56136;56137;56138;56139;56140;56141;56142;56143;56144;56145;56146;56147;56148;56149;56150;56151;56152;56153;56154;56155;56156;56157;56158;56159;56160;56161;56162;56163;56164;56165;56166;56167;56168;56169;56170;56171;56172;56173;56174;56175;56176;56177;56178;56179;56180;56181;56182;56183;56184;56185;56186;56187;56188;56189;56190;56191;56192;56193;56194;56195;56196;56197;56198;56199;56200;56201;56211;56212;56213;56214;56215;56216;56217;56218;56219;56220;56221;56222;56223;56224;56225;56226;56227;56228;56229;56230;56231;56232;56233;56234;56235;56236;56237;56238;56239;56240;56241;56242;56243;56244;56245;56246;56247;56248;56249;56250;56251;56252;56253;56254;56255;56256;56257;56258;56259;56260;56261;56262;56263;56264;56265;56266;56267;56268;56269;56270;56271;56272;56273;56274;56275;56276;56277;56278;56279;56280;56281;56282;56283;56284;56285;56286;56287;56288;56289;56290;56291;56292;56293;56294;56295;56296;56297;56298;56347;56348;56349;56350;56351;56352;56353;56354;56355;56356;56357;56358;56359;56360;56361;56362;56363;56364;56365;56366;56367;56368;56369;56370;56371;56372;56373;56374;56375;56376;56377;56378;56379;56380;56381;56382;56383;56384;56385;56386;56387;56388;56389;56390;56391;56392;56393;56394;56404;56405;56406;56407;56408;56409;56410;56411;56412;56413;56414;56415;56416;56417;56418;56419;56420;56421;56422;56423;56424;56425;56426;56427;56428;56429;56430;56431;56432;56433;56434;56435;56436;56437;56438;56439;56440;56441;56442;56443;56444;56445;56446;56447;56448;56449;56450;56451;56452;56453;56454;56455;56456;56457;56458;56459;56460;56461;56462;56463;56464;56465;56466;56467;56468;56469;56470;56475;56476;56477;56478;56479;56480;56481;56482;56483;56484;56485;56486;56487;56488;56489;56490;56491;56492;56493;56494;56495;56496;56497;56498;56499;56500;56501;56502;56503;56504;56505;56506;56507;56508;56509;56510;56511;56512;56513;56514;56515;56516;56517;56518;56519;56520;56521;56522;56523;56524;56525;56526;56527;56528;56529;56530;56531;56532;56533;56534;56535;56536;56537;56538;56539;56540;56541;56542;56543;56544;56545;56546;56547;56548;56549;56550;56551;56552;56553;56554;56555;56556;56557;56558;56559;56560;56561;56562;56563;56564;56565;56566;56567;56568;56569;56570;56571;56572;56573;56574;56575;56576;56577;56578;56579;56580;56581;56582;56583;56584;56585;56586;56587;56588;56589;56590;56591;56592;56593;56594;56595;56596;56597;56598;56599;56600;56601;56602;56603;56604;56605;56606;56607;56608;56609;56610;56611;56612;56613;56614;56615;56616;56617;56618;56619;56620;56621;56622;56623;56624;56625;56626;56627;56628;56629;56630;56631;56632;56633;56634;56635;56636;56637;56638;56639;56640;56641;56642;56643;56644;56645;56646;56647;56648;56649;56650;56651;56652;56653;56654;56655;56656;56657;56658;56659;56660;56661;56662;56663;56664;56665;56666;56667;56668;56669;56670;56671;56672;56673;56674;56675;56676;56677;56678;56679;56680;56681;56682;56683;56684;56685;56686;56687;56688;56689;56690;56691;56692;56693;56694;56695;56696;56697;56698;56699;56700;56701;56702;56703;56704;56705;56706;56707;56708;56709;56710;56711;56712;56713;56714;56715;56716;56717;56718;56719;56720;56721;56722;56723;56724;56725;56726;56727;56728;56729;56730;56731;56732;56733;56734;56735;56736;56737;56738;56739;56740;56741;56742;56743;56744;56745;56746;56747;56748;56749;56750;56751;56752;56753;56754;56755;56756;56757;56758;56759;56760;56761;56762;56763;56764;56765;56766;56767;56768;56769;56770;56771;56772;56773;56774;56775;56776;56777;56778;56779;56780;56781;56782;56783;56784;56785;56786;56787;56788;56789;56790;56791;56792;56793;56794;56795;56796;56797;56798;56799;56800;56801;56802;56803;56804;56805;56806;56807;56808;56809;56810;56811;56812;56813;56814;56815;56816;56817;56818;56819;56820;56821;56822;56823;56824;56825;56826;56827;56828;56829;56830;56831;56832;56833;56834;56835;56836;56837;56838;56839;56840;56841;56842;56843;56844;56845;56846;56847;56848;56849;56850;56851;56852;56853;56854;56855;56856;56857;56858;56859;56860;56861;56862;56863;56864;56865;56866;56867;56868;56869;56870;56871;56872;56873;56874;56875;56876;56877;56878;56879;56880;56881;56882;56883;56884;56885;56886;56887;56888;56889;56890;56891;56892;56893;56894;56895;56896;56897;56898;56899;56900;56901;56902;56903;56904;56905;56906;56907;56908;56909;56910;56911;56912;56913;56914;56915;56916;56917;56918;56919;56920;56921;56922;56923;56924;56925;56926;56927;56928;56929;56930;56931;56932;56933;56934;56935;56936;56937;56938;56939;56940;56941;56942;56943;56944;56945;56946;56947;56948;56949;56950;56951;56952;56953;56954;56955;56956;56957;56958;56959;56960;56961;56962;56963;56964;56965;56966;56967;56968;56969;56970;56971;56972;56973;56974;56975;56976;56977;56978;56979;56980;56981;56982;56983;56984;56985;56986;56987;56988;56989;56990;56991;56992;56993;56994;56995;56996;56997;56998;56999;57000;57001;57002;57003;57004;57005;57006;57007;57008;57009;57010;57011;57012;57013;57014;57015;57016;57017;57018;57019;57020;57021;57022;57023;57024;57025;57026;57027;57028;57029;57030;57031;57032;57033;57034;57035;57036;57037;57038;57039;57040;57041;57042;57043;57044;57045;57046;57047;57048;57049;57050;57051;57052;57053;57054;57055;57056;57057;57058;57059;57060;57061;57062;57063;57064;57065;57066;57067;57068;57069;57070;57071;57072;57073;57074;57075;57076;57077;57078;57079;57080;57081;57082;57083;57084;57085;57086;57087;57088;57089;57090;57091;57092;57093;57094;57095;57096;57097;57098;57099;57100;57101;57102;57103;57104;57105;57106;57107;57108;57109;57110;57111;57112;57113;57114;57115;57116;57117;57118;57119;57120;57121;57122;57123;57124;57125;57126;57127;57128;57129;57130;57131;57132;57133;57134;57135;57136;57137;57138;57139;57140;57141;57142;57143;57144;57145;57146;57147;57148;57149;57150;57151;57152;57153;57154;57155;57156;57157;57158;57159;57160;57161;57162;57163;57164;57165;57166;57167;57168;57169;57170;57171;57172;57173;57174;57175;57176;57177;57178;57179;57180;57181;57182;57183;57184;57185;57186;57187;57188;57189;57190;57191;57192;57193;57194;57195;57196;57197;57198;57199;57200;57201;57202;57279;57280;57281;57282;57283;57284;57285;57286;57287;57288;57289;57290;57291;57292;57293;57294;57295;57296;57297;57298;57299;57300;57301;57302;57303;57304;57305;57306;57307;57308;57309;57310;57311;57312;57313;57314;57315;57316;57317;57318;57319;57320;57321;57322;57323;57324;57325;57326;57327;57328;57329;57330;57331;57332;57333;57334;57335;57336;57337;57338;57339;57340;57341;57342;57343;57344;57345;57346;57347;57348;57349;57350;57351;57352;57353;57354;57355;57356;57357;57358;57359;57360;57361;57362;57363;57364;57365;57366;57367;57368;57369;57370;57371;57372;57373;57374;57375;57376;57377;57378;57379;57380;57381;57382;57384;57385;57386;57387;57388;57389;57390;57391;57392;57393;57394;57395;57396;57397;57398;57399;57400;57401;57402;57403;57404;57405;57406;57407;57408;57409;57410;57411;57412;57413;57414;57415;57416;57417;57418;57419;57420;57421;57422;57423;57424;57425;57426;57427;57428;57429;57430;57431;57432;57433;57434;57435;57436;57437;57438;57439;57440;57441;57442;57443;57444;57445;57446;57447;57448;57449;57450;57451;57452;57453;57454;57455;57456;57457;57458;57459;57460;57461;57462;57463;57464;57465;57466;57467;57468;57469;57470;57471;57472;57473;57474;57475;57476;57477;57478;57479;57480;57481;57482;57483;57484;57485;57486;57487;57488;57489;57490;57491;57492;57493;57494;57495;57496;57497;57498;57499;57500;57501;57502;57503;57504;57505;57506;57507;57508;57509;57510;57511;57512;57513;57514;57515;57516;57517;57518;57519;57520;57521;57522;57523;57524;57525;57526;57527;57528;57529;57530;57531;57532;57533;57534;57535;57536;57537;57538;57539;57540;57541;57542;57543;57544;57545;57546;57547;57548;57549;57550;57551;57552;57553;57554;57555;57556;57557;57558;57559;57560;57561;57562;57563;57564;57565;57566;57567;57568;57569;57570;57571;57572;57573;57574;57575;57576;57577;57578;57579;57580;57581;57582;57583;57584;57585;57586;57587;57588;57589;57590;57591;57592;57593;57594;57595;57596;57597;57598;57599;57600;57601;57602;57603;57604;57605;57606;57607;57608;57609;57610;57611;57612;57613;57614;57615;57616;57617;57618;57619;57620;57621;57622;57623;57624;57625;57626;57627;57628;57629;57630;57631;57632;57633;57634;57635;57636;57637;57638;57672;57673;57674;57675;57676;57677;57678;57679;57680;57681;57682;57683;57684;57685;57686;57687;57688;57689;57690;57691;57692;57693;57694;57695;57696;57697;57698;57699;57700;57701;57702;57703;57704;57705;57706;57707;57708;57709;57710;57711;57712;57713;57714;57715;57716;57717;57718;57719;57720;57721;57722;57723;57724;57725;57726;57727;57728;57729;57730;57731;57732;57733;57734;57735;57736;57737;57738;57739;57740;57741;57742;57743;57744;57745;57746;57747;57748;57749;57750;57751;57752;57753;57754;57755;57756;57757;57758;57759;57760;57761;57762;57763;57764;57765;57766;57767;57768;57769;57770;57771;57772;57773;57774;57775;57776;57777;57778;57779;57780;57781;57782;57783;57784;57785;57786;57787;57788;57789;57790;57791;57792;57793;57794;57795;57796;57797;57798;57799;57800;57801;57802;57803;57804;57805;57806;57807;57808;57809;57810;57811;57812;57813;57814;57815;57816;57817;57818;57819;57820;57821;57822;57823;57824;57825;57826;57838;57839;57840;57841;57842;57843;57844;57845;57846;57847;57848;57849;57850;57851;57852;57853;57854;57855;57856;57857;57858;57859;57860;57861;57862;57863;57864;57865;57866;57867;57868;57869;57870;57871;57872;57873;57874;57875;57876;57877;57878;57879;57880;57881;57882;57883;57884;57885;57886;57887;57888;57889;57890;57891;57892;57893;57894;57895;57896;57897;57898;57899;57900;57901;57902;57903;57904;57905;57906;57907;57908;57909;57910;57911;57912;57913;57914;57915;57916;57917;57918;57919;57920;57921;57922;57923;57924;57925;57926;57927;57928;57929;57930;57931;57932;57933;57934;57935;57936;57937;57938;57939;57940;57941;57942;57943;57944;57945;57946;57947;57948;57949;57950;57951;57952;57953;57954;57955;57956;57957;57958;57959;57960;57961;57962;57963;57964;57965;57966;57967;57968;57969;57970;57971;57972;57973;57974;57975;57976;57977;57978;57979;57980;57981;57982;58033;58034;58035;58036;58037;58038;58039;58040;58041;58042;58043;58044;58045;58046;58047;58048;58049;58050;58051;58052;58053;58054;58055;58056;58057;58058;58059;58060;58061;58062;58063;58064;58065;58066;58067;58068;58069;58070;58071;58072;58073;58074;58075;58076;58077;58078;58079;58080;58081;58082;58083;58084;58085;58086;58087;58088;58089;58090;58091;58092;58093;58094;58095;58096;58097;58098;58099;58100;58101;58102;58103;58104;58105;58106;58107;58108;58109;58110;58111;58112;58113;58114;58115;58116;58117;58118;58119;58120;58121;58122;58123;58124;58125;58126;58127;58133;58134;58135;58136;58137;58138;58139;58140;58141;58142;58143;58144;58145;58146;58147;58148;58149;58150;58151;58152;58153;58154;58155;58156;58157;58158;58159;58160;58161;58162;58163;58164;58165;58166;58167;58168;58169;58170;58171;58172;58173;58174;58175;58176;58177;58178;58179;58180;58181;58182;58183;58184;58185;58186;58187;58188;58189;58190;58191;58192;58193;58194;58195;58196;58197;58198;58199;58200;58201;58202;58203;58204;58205;58206;58207;58208;58209;58210;58211;58212;58213;58214;58215;58216;58217;58218;58309;58310;58311;58312;58313;58314;58315;58316;58317;58318;58319;58320;58321;58322;58323;58324;58325;58326;58327;58328;58329;58330;58331;58332;58333;58334;58335;58336;58337;58338;58339;58340;58341;58342;58343;58344;58345;58346;58347;58348;58349;58350;58351;58352;58353;58354;58355;58356;58357;58358;58359;58360;58361;58362;58363;58364;58365;58366;58367;58368;58369;58370;58371;58372;58373;58374;58375;58376;58377;58378;58379;58380;58381;58382;58383;58384;58385;58386;58387;58388;58389;58390;58391;58392;58393;58394;58395;58396;58397;58398;58399;58400;58401;58402;58403;58404;58405;58406;58407;58408;58409;58410;58411;58412;58413;58414;58415;58416;58417;58418;58419;58420;58421;58422;58423;58424;58425;58426;58427;58428;58429;58430;58431;58432;58433;58434;58435;58436;58437;58438;58439;58440;58441;58442;58443;58444;58445;58446;58447;58448;58449;58450;58451;58452;58453;58454;58455;58456;58457;58458;58459;58460;58461;58462;58463;58464;58465;58466;58467;58468;58469;58470;58471;58472;58473;58474;58475;58476;58477;58478;58479;58480;58481;58482;58483;58484;58485;58486;58487;58488;58489;58490;58491;58492;58493;58494;58495;58496;58497;58498;58499;58500;58501;58502;58503;58504;58505;58506;58507;58508;58509;58510;58511;58512;58513;58514;58515;58516;58517;58518;58519;58520;58521;58522;58523;58524;58525;58526;58527;58528;58529;58530;58531;58532;58533;58534;59282;59283;59284;59285;59286;59287;59288;59289;59290;59291;59292;59293;59294;59295;59296;59297;59298;59299;59300;59301;59302;59303;59304;59305;59306;59307;59308;59309;59310;59311;59464;59465;59466;59467;59468;59469;59470;59471;59472;59473;59474;59475;59476;59477;59478;59479;59480;59481;59482;59483;59484;59485;59486;59487;59488;59489;59490;59491;59492;59493;59494;59495;59496;59497;59498;59499;59500;59501;59502;59503;59504;59505;59506;59507;59508;59509;59510;59511;59512;59513;59514;59515;60332;66774;66775;66776;66777;66778;66779;66780;66781;66782;66783;66784;66785;66786;66787;66788;66789;66790;66791;66792;66793;66794;66795;66796;66797;66798;66799;66800;66801;66802;66803;67214;67215;67216;67217;67218;67219;67220;67221;67222;67223;67224;67225;67226;67227;67228;67229;67230;67231;67232;67233;67234;67235;67236;67237;67238;67239;67240;67241;67242;67243;67244;67245;67246;67247;67248;67249;67250;67251;67252;67253;67254;67255;67256;67257;67258;67259;67260;67261;67262;67263;67264;67265;67266;67267;67268;67269;67270;67271;67272;67273;67274;67275;67276;67277;67278;67279;67280;67281;67282;67283;67284;67285;67286;67287;67288;67289;67290;67291;67292;67293;67294;67295;67296;67297;67298;67299;67300;67301;67302;67303;67304;67305;67306;67307;67308;67309;67310;67311;67312;67313;67314;67315;67316;67317;67318;67319;67320;67321;67322;67323;67324;67325;67326;67327;67328;67329;67330;67331;67332;67333;67334;67335;67336;67337;67338;67339;67340;67341;67342;67343;67344;67345;67346;67347;67348;67349;67350;67351;67352;67353;67354;67355;67356;67357;67358;67359;67360;67361;67362;67363;67364;67365;67366;67367;67368;67369;67370;67371;67372;67373;67374;67375;67376;67377;67378;67379;67380;67381;67382;67383;67384;67385;67386;67387;67388;67389;67390;67391;67392;67393;67394;67395;67396;67397;67398;67399;67400;67401;67402;67403;67404;67405;67406;67407;67408;67409;67410;67411;67412;67413;67414;67415;67416;67417;67418;67419;67420;67421;67422;67423;67424;67425;67426;67427;67428;67429;67430;67431;67432;67433;67434;67435;67436;67437;67438;67439;67440;67441;67442;69066;69067;69068;69069;69070;69071;69072;69073;69074;69075;69076;69077;69078;69079;69080;69081;69082;69083;69084;69085;69086;69087;69088;69089;69090;69091;69092;69093;69094;69095;69096;69097;69098;69099;69100;69101;69102;69103;69104;69105;69106;69107;69108;69109;69110;69111;69112;69113;69114;69115;69116;69117;69118;69119;69120;69121;69122;69123;69124;69125;69126;69127;69128;69129;69130;69131;69132;69133;69134;69135;69136;69137;69138;69139;69140;69141;69142;69143;69144;69145;69146;69147;69148;69149;69150;69151;69152;69153;69154;69155;69156;69157;69158;69159;69160;69161;69162;69163;69164;69165;69166;69167;70050;70051;70052;70053;70054;70055;70056;70057;70058;70059;70060;70061;70062;70063;70064;70065;70066;70067;70068;70069;70070;70071;70072;70073;70074;70075;70076;70077;70078;70079;70080;70081;70082;70083;70084;70085;70086;70087;70088;70089;70090;70091;70092;70093;70094;70095;70096;70097;70098;70099;70100;70101;70163;70164;70165;70166;70167;70168;70169;70170;70171;70172;70173;70174;70175;70176;70177;70178;70179;70180;70181;70182;70183;70184;70185;70186;70187;70188;70189;70190;70191;70192;70193;70194;70195;70196;70197;70198;70199;70200;70201;70202;70203;70204;70205;70206;70207;70208;70209;70210;70211;70212;70213;70214;70215;70216;70217;70218;70219;70220;70221;70222;70223;70224;70225;70226;70227;70228;70229;70230;70231;70232;70233;70234;70235;70236;70237;70238;70239;70240;70241;70242;70243;70244;70245;70246;70247;70248;70249;70250;70251;70252;70253;70254;70255;70256;70257;70258;70259;70260;70261;70262;70263;70264;70265;70266;70267;70268;70269;70270;70271;70272;70273;70274;70275;70276;70277;70278;70279;70280;70281;70282;70283;70284;70285;70286;70287;70288;70289;70290;70291;70292;70293;70294;70295;70296;70297;70298;70299;70300;70301;70302;70303;70304;70305;70306;70307;70308;70309;70310;70311;70312;70313;70314;70315;70316;70317;70318;70319;70320;70321;70322;70323;70324;70325;70326;70327;70328;70329;70330;70331;70332;70333;70334;70335;70336;70337;70338;70339;70340;70341;70342;70343;70344;70345;70346;70347;70348;70349;70350;70351;70352;70353;70354;70355;70356;70357;70358;70359;70360;70361;70362;70363;70364;70365;70366;70367;70368;70369;70370;70371;70372;70373;70374;70375;70376;70377;70378;70379;70380;70381;70382;70383;70384;70385;70386;70387;70388;70389;70390;70391;70392;70393;70394;70395;70396;70397;70398;70399;70400;70401;70402;70403;70404;70405;70406;70407;70408;70409;70410;70411;70412;70413;70414;70415;70416;70417;70418;70419;70420;70421;70422;70423;70424;70425;70426;70427;70428;70429;70430;70431;70432;70433;70434;70435;70436;70437;70438;70439;70440;70441;70442;70443;70444;70445;70446;70447;70448;70449;70450;70451;70452;70453;70454;70455;70456;70457;70458;70459;70460;70461;70462;70463;70464;70465;70466;70467;70468;70469;70470;70471;70472;70473;70474;70475;70476;70477;70478;70479;70480;70481;70482;70483;70484;70485;70486;70487;70488;70489;70490;70491;70492;70493;70494;70495;70496;70497;70498;70499;70500;70501;70502;70503;70504;70505;70506;70507;70508;70509;70510;70511;70512;70513;70514;70515;70516;70517;70518;70519;70520;70521;70522;70523;70524;70525;70526;70527;70528;70529;70530;70531;70532;70533;70534;70535;70536;70537;70538;70539;70540;70541;70542;70543;70544;70545;70546;70547;70548;70549;70550;70551;70552;70553;70554;70555;70556;70557;70558;70559;70560;70561;70562;70563;70564;70565;70566;70567;70568;70569;70570;70571;70572;70573;70574;70575;70576;70577;70578;70579;70580;70581;70582;70583;70584;70585;70586;70587;70588;70589;70590;70591;70592;70593;70594;70595;70596;70597;70598;70599;70600;70601;70602;70603;70604;70605;70606;70607;70608;70609;70610;70611;70612;70613;70614;70615;70616;70617;70618;70619;70620;70621;70622;70623;70624;70625;70626;70627;70628;70629;70630;70631;70632;70633;70634;70635;70636;70637;70638;70639;70640;70641;70642;70643;70644;70645;70646;70647;70648;70649;70650;70651;70652;70653;70654;70655;70656;70657;70658;70659;70660;70661;70662;70663;70664;70665;70666;70667;70668;70669;70670;70671;70672;70673;70674;70675;70676;70677;70678;70679;70680;70681;70682;70683;70684;70685;70686;70687;70688;70689;70690;70691;70692;70693;70694;70695;70696;70697;70698;70699;70700;70701;70702;70703;70704;70705;70706;70707;70708;70709;70710;70711;70712;70713;70714;70715;70716;70717;70718;70719;70720;70721;70722;70723;70724;70725;70726;70727;70728;70729;70730;70731;70732;70733;70734;70735;70736;70737;70738;70739;70740;70741;70742;70743;70744;70745;70746;70747;70748;70749;70750;70751;70752;70753;70754;70755;70756;70757;70758;70759;70760;70761;70762;70763;70764;70765;70766;70767;70768;70769;70770;70771;70772;70773;70774;70775;70776;70777;70778;70779;70780;70781;70782;70783;70784;70785;70786;70787;70788;70789;70790;70791;70792;70793;70794;70795;70796;70797;70798;70799;70800;70801;70802;70803;70804;70805;70806;70807;70808;70809;70810;70811;70812;70813;70814;70815;70816;70817;70818;70819;70820;70821;70822;70823;70824;70825;70826;70827;70828;70829;70830;70831;70832;70833;70834;70835;70836;70837;70838;70839;70840;70841;70842;70843;70844;70845;70846;70847;70848;70849;70850;70851;70852;70853;70854;70855;70856;70857;70858;70859;70860;70861;70862;70863;70864;70865;70866;70867;70868;70869;70870;70871;70872;70873;70874;70875;70876;70877;70878;70879;70880;70881;70882;70883;70884;70885;70886;70887;70888;70889;70890;70891;70892;70893;70894;70895;70896;70897;70898;70899;70900;70901;70902;70903;70904;70905;70906;70907;70908;70909;70910;70911;70912;70913;70914;70915;70916;70917;70918;70919;70920;70921;70922;70923;70924;70925;70926;70927;70928;70929;70930;70931;70932;70933;70934;70935;70936;70937;70938;70939;70940;70941;70942;70943;70944;70945;70946;70947;70948;70949;70950;70951;70952;70953;70954;70955;70956;70957;70958;70959;70960;70961;70962;70963;70964;70965;70966;70967;70968;70969;70970;70971;70972;70973;70974;70975;70976;70977;70978;70979;70980;70981;70982;70983;70984;70985;70986;70987;70988;70989;70990;70991;70992;70993;70994;70995;70996;70997;70998;70999;71000;71001;71002;71003;71004;71005;71006;71007;71008;71009;71010;71011;71012;71013;71014;71015;71016;71017;71018;71019;71020;71021;71022;71023;71024;71025;71026;71027;71028;71029;71030;71031;71032;71033;71034;71035;71036;71037;71038;71039;71040;71041;71042;71043;71044;71045;71046;71047;71048;71049;71050;71051;71052;71053;71054;71055;71056;71057;71058;71059;71060;71061;71062;71063;71064;71065;71066;71067;71068;71069;71070;71071;71072;71073;71074;71075;71076;71077;71078;71079;71080;71081;71082;71083;71084;71085;71086;71087;71088;71089;71090;71091;71092;71093;71094;71095;71096;71097;71098;71099;71100;71101;71102;71103;71104;71105;71106;71107;71108;71109;71110;71111;71112;71113;71114;71115;71116;71117;71118;71119;71120;71121;71122;71123;71124;71125;71126;71127;71128;71129;71130;71131;71132;71133;71134;71135;71136;71137;71138;71139;71140;71141;71142;71143;71144;71145;71146;71147;71148;71149;71150;71151;71152;71153;71154;71155;71156;71157;71158;71159;71160;71161;71162;71163;71164;71165;71166;71167;71168;71169;71170;71171;71172;71173;71174;71175;71176;71177;71178;71179;71180;71181;71182;71183;71184;71185;71186;71187;71188;71189;71190;71191;71192;71193;71194;71195;71196;71197;71198;71199;71200;71201;71202;71203;71204;71205;71206;71207;71208;71209;71210;71211;71212;71213;71214;71215;71216;71217;71218;71219;71220;71221;71222;71223;71224;71225;71226;71227;71228;71229;71230;71231;71232;71233;71234;71235;71236;71237;71238;71239;71240;71241;71242;71243;71244;71245;71246;71247;71248;71249;71250;71251;71252;71253;71254;71255;71256;71257;71258;71259;71260;71261;71262;71263;71264;71265;71266;71267;71268;71269;71270;71271;71272;71273;71274;71275;71276;71277;71278;71279;71280;71281;71282;71283;71284;71285;71286;71287;71288;71289;71290;71291;71292;71293;71294;71295;71296;71297;71298;71299;71300;71301;71302;71303;71304;71305;71306;71307;71308;71309;71310;77356;77357;77358;77359;77360;77361;77362;77363;77364;77365;77366;77367;77368;77369;77370;77371;77372;77373;77374;77375;77376;77377;77378;77379;77380;77381;77382;77383;77384;77385;77386;77387;77388;77389;77390;77391;77392;77393;77394;77395;77396;77397;77398;77399;77400;77401;77402;77403;77404;77405;77406;77407;77408;77409;77410;77411;77412;77413;77414;77415;77416;77417;77418;77419;77420;77421;77422;77423;77424;77425;77426;77427;77428;77429;77430;77431;77432;77433;77434;77435;77436;77437;77438;77439;77440;77441;77442;77443;77444;77445;77446;77847;77848;77849;77850;77851;77852;77853;77854;77855;77856;81505;81506;81507;81508;83636;83637;83638;83639;83640;83641;83642;83643;83644;83645;83646;83647;83648;83649;83650;83651;83652;83653;83654;83655;83656;83657;83658;83659;83660;83661;83662;83663;83664;83665;83666;83667;83668;83669;83670;83671;83672;83673;83674;83675;83676;83677;83678;83679;83680;83681;83682;83683;83684;83685;83686;83687;83688;83689;83690;83691;83692;83693;83694;83695;83696;83697;83698;83699;83700;83701;83702;83703;83704;85744;85745;85746;85747;85748;85749;85750;85751;85752;85753;85754;85755;85756;85757;85758;85759;85760;85761;85762;85763;85764;85765;85766;85767;85768;85769;85770;85771;85772;85773;85774;85775;85776;85777;85778;85779;85780;85781;85782;85783;85784;85785;85786;85787;85788;85789;85790;85791;85792;85793;85794;85795;85796;85797;85798;85799;85800;85801;85802;85803;85804;85805;85806;86165;86166;86167;86168;86169;87892;87893;87894;87895;87896;87897;87898;87899;87900;87901;87902;87903;87904;87905;87906;87907;87908;87909;87910;89434;89435;89436;89437;89438;89439;89440;89441;89442;89443;89444;89445;89446;89447;91532;91533;91534;91535;91536;91537;91538;91539;91540;91541;91542;91543;91544;91545;91546;91547;91548;91549;91550;91551;91552;91553;91554;91555;91556;91557;91558;91559;91560;91561;91562;91563;91564;91565;91566;91567;91568;91569;91570;91571;91572;91573;91574;91575;91576;91577;91578;91579;91580;91581;91582;91583;91584;91585;91586;91587;91588;91589;91590;91591;91592;91593;91594;91595;91596;91597;91598;91599;91600;91601;91602;91603;91604;91605;91606;91607;91608;91609;91610;91611;91612;91613;91614;92169;92170;92171;92172;92173;92174;92175;92176;92177;92178;92179;92180;92181;93142;93143;93144;93145;93146;93147;93148;93149;93150;93151;93152;93153;93154;93155;93156;93157;93158;93159;93160;93161;94213;94214;94215;94216;94271;94272;94273;94274;94275;94276;94277;94278;94279;94280;94281;94282;94283;94284;94285;94286;94287;94288;94289;94290;94291;94292;94293;94294;94295;94296;94297;94298;94299;94300;94301;94302;94303;94304;94305;94306;94307;94308;94309;94310;94311;94312;94313;94314;94315;94316;94317;94318;94319;94320;94321;94322;94323;94324;94325;94326;94327;94328;94329;94330;94331;94332;94333;94334;94335;94336;94337;94338;94339;94340;94341;94342;94343;94344;94345;94346;94347;94348;94349;94350;94351;94352;94353;94354;94355;94362;94363;94364;94365;94366;94367;94368;94369;94370;94371;94372;94373;94374;94375;94376;94377;94378;94379;94380;94381;94382;94383;94384;94385;94386;94387;94388;94389;94390;94391;94392;94393;94394;94395;94396;94397;94398;94399;94400;94401;94402;94403;94404;94405;94406;94407;94408;94409;94410;94411;94412;94413;94414;94415;94416;94417;94418;94546;94547;94548;94549;94550;94551;94552;94553;94554;94555;94556;94557;94558;94559;94560;94561;94562;94563;94564;94565;94566;94567;94568;94569;94570;94571;94572;94573;94574;94575;94576;94577;94578;94579;94580;94581;94582;94583;94584;94585;94586;94587;94588;94589;94590;94591;94592;94593;94594;94595;94596;94597;94598;94599;94600;94601;94602;94603;94604;94605;94606;94607;94608;94609;94610;94611;94612;94613;94614;94615;94616;94617;94618;94619;94620;95614;95615;95616;95617;95618;95619;95620;95621;95622;95623;95624;95625;95626;95627;95628;95629;95630;95631;95632;95633;95634;95635;95636;95637;95638;95639;95640;95641;95642;95643;95644;95645;95646;95647;95648;95649;95650;95651;95652;95653;95654;95655;95656;95657;95658;95659;95660;95661;95662;95663;95664;95665;95666;95667;95668;95669;95670;95671;95672;95673;95674;95675;95676;95677;95678;95679;95680;95681;95682;95683;95684;95685;96319;96320;96321;96322;96323;96324;96325;96326;96327;96328;96329;96330;96331;96332;96333;96334;96335;96336;96337;96338;96339;96340;96341;96342;96343;96344;96345;96346;96347;96348;96349;96350;96351;96352;96353;96354;96355;96356;96357;96358;96359;96360;96361;96362;96363;96364;96365;96366;96367;96368;96369;96370;96371;96372;96373;96374;96375;96376;96377;96378;96379;96380;96381;96382;96383;96384;96385;96386;96387;96388;96389;96390;96391;96392;96393;96394;96395;96396;96397;96398;107048;107049;107050;107051;107052;107053;107054;107055;107056;107057;107058;107059;107060;107061;107062;107063;107064;107065;107066;107067;107068;107069;107070;107071;107072;107073;107074;116138;116139;116140;116141;116142;116143;116144;116145;116146;116147;116148;116149;116150;116151;116152;116153;116154;116155;116156;116157;116158;116159;116160;116161;116162;116163;116164;116165;116166;116167;116168;116169;116170;116171;116172;116173;116174;116175;116176;116177;116178;116179;116180;116181;116182;116183;116184;116185;116186;116187;116188;116189;116190;116191;116192;116193;116194;116195;116196;116197;116198;116199;116200;116201;116202;116203;116204;116205;116206;116207;116208;116209;116210;116211;116212;116213;116214;116215;119987;119988;119989;119990;119991;119992;119993;119994;119995;119996;119997;119998;119999;120000;120001;120002;120003;120004;120005;120006;120007;120008;120009;120010;120011;120012;120013;120014;120015;120016;120017;120018;120019;120020;120021;120022;120023;120024;120025;120026;120027;120028;120029;120030;120031;120032;120033;120034;120035;120036;120037;120038;120039;120040;120041;120042;120043;120044;120045;126544;126545;126546;126547;126548;126549;126550;126551;126552;126553;126554;126555;126556;126557;126558;126559;126560;126561;126562;126563;126564;126565;126566;126567;126568;126569;126570;126571;126572;126573;126574;126575;126576;126577;126578;126579;126580;126581;126582;126583;126584;126585;126586;126587;126588;126589;126590;126591;126592;126593;126594;126595;126596;126597;126598;126599;126600;126601;126602;126603;126604;126605;126606;126607;126608;126609;126610;126611;126612;126613;126614;126615;126616;126617;126618;126619;126620;126621;126622;126623;126624;126625;126626;126627;126628;126629;126630;126631;126632;126633;126634;126635;126636;126637;126638;126639;126640;126641;126642;126643;126644;126645;126646;126647;126648;126649;126650;126651;126652;126653;126654;126655;126656;126657;126658;126659;126660;126661;126662;126663;126664;126665;126666;126667;126668;126669;126670;126671;126672;126673;126674;126675;126676;126677;126678;126679;126680;126681;133294;133295;133296;133297;133298;133299;133300;133301;133302;133303;133304;133305;133306;133307;133308;133309;133310;133311;133312;133313;133314;133315;133316;133317;133318;133319;133320;133321;133322;133323;133324;133325;133326;133327;133328;133329;133330;133331;138901;138902;138903;138904;138905;138906;138907;138908;138909;138910;138911;138912;138913;138914;138915;138916;138917;138918;138919;138920;138921;138922;138923;138924;138925;138926;138927;138928;138929;138930;138931;138932;138933;138934;138935;138936;138937;138938;138939;138940;138941;138942;138943;138944;138945;138946;138947;138948;138949;138950;138951;138952;138953;138954;138955;138956;138957;138958;138959;139059;139060;139061;139062;139063;139064;139065;139066;139301;139302;139303;139304;139305;139306;139307;139308;139309;139310;139311;139312;139313;139314;139315;139316;139317;139318;139319;139320;139321;139322;139323;139324;139325;139326;139327;139328;139329;139330;139331;139332;139333;139334;139335;139336;139337;139338;139339;139372;139373;139374;139375;139376;139377;139378;139379;139380;139381;139382;139383;139384;139385;139386;139387;139388;139389;139390;139391;139392;139393;139394;139395;139396;139397;139398;139399;139400;139401;139402;139403;139404;139405;139406;139407;139408;139409;139410;139411;139412;139413;139414;139415;139416;139417;139418;139419;139420;139421;139422;139423;139424;139425;139426;139427;139428;139429;139430;139431;139432;139433;139434;139435;139436;139437;139438;139439;139440;139441;139442;139443;139444;139445;139446;139447;139530;139531;139532;139533;139534;139535;139536;139537;139538;139539;139540;139541;139542;139543;139544;139545;139546;139547;139548;139549;139550;139551;139552;139553;139554;139555;139556;139557;139558;139559;139560;139561;139562;139563;139564;139565;139566;139567;139568;139569;139570;139571;139572;139573;139574;139575;139576;139577;139578;139579;139580;139581;139582;139583;139584;139585;139586;139587;139588;139589;139590;139591;139592;139593;139594;139595;139596;139597;139598;139599;139600;139601;139602;139603;139604;139605;139606;139607;139608;139609;139610;139611;139612;139613;139614;139615;139616;139617;139618;139619;139620;139621;139622;139623;139624;139625;139626;139627;139628;139629;139630;139631;139632;139633;139634;139635;139636;139637;139638;139639;139640;139641;139767;139768;139769;139770;139771;139772;139773;139774;139775;139776;139777;139778;139779;139780;139781;139782;139783;139784;139785;139786;139787;139788;139789;139790;139791;139792;139793;139794;139795;142772;146387;146388;146389;146390;146391;146392;146393;146394;146395;146396;146397;146398;146399;146400;146401;146402;146403;146404;146405;146406;146407;146408;146409;146410;146411;146412;146413;146414;146415;146416;146417;146418;146419;146420;146421;146422;146423;146424;146425;146426;146427;146428;146429;146430;146431;146432;146433;146434;146435;146436;146437;146438;146439;146440;146441;146442;146443;146444;146445;146446;146447;146448;146449;146450;146451;146452;146453;146454;146455;146456;146457;146458;146459;146460;146461;146462;146463;146464;146465;146466;146467;146468;146469;146470;146471;146472;146473;146474;146475;146476;146597;146598;146599;146600;146601;146602;146603;146604;146605;146606;146607;146608;146609;146610;146611;146612;146613;146614;146615;146616;146617;146618;146619;146695;146696;146697;146698;146699;146700;146701;146702;146703;146704;146705;146706;146707;146708;146709;146710;146711;146712;146713;146714;146715;146716;146717;146718;146719;146720;146721;146722;146723;146724;146725;146756;146757;146758;146825;146826;146827;146828;146829;146830;146831;146832;146833;146834;146835;146836;146837;146838;146839;146840;146841;146842;147025;147026;147027;147028;148482;148483;148484;148485;148486;148487;148488;148489;148490;148491;148492;148493;148494;148495;148496;148497;148498;148499;148500;148501;148502;148503;148504;148505;148506;148507;148508;148509;148510;148511;148512;148513;148514;148515;148516;148517;148518;148519;148520;148521;148522;148523;148524;148525;148526;148527;148528;148529;148530;148531;148532;148533;148534;148535;148536;148537;148538;148539;148540;148541;148542;148543;148544;148545;148546;148547;148548;148549;149843;149844;149845;149846;149847;149848;149849;149850;149851;149852;149853;149854;149855;149856;149857;149858;149859;149860;149861;149862;149863;149864;149865;149866;149867;149868;149869;149870;149871;149872;149873;149874;149875;149876;149877;149878;149879;149880;149881;149882;149883;149884;149885;149886;149887;149888;149889;149890;149891;149892;149893;149894;149895;149896;149897;149898;149899;149900;149901;149902;149903;149904;149905;149906;149907;149908;149909;149910;149911;149912;149913;149914;149915;149916;149917;149918;149919;149920;149921;149922;149923;149924;149925;149926;149927;149928;149929;149930;149931;149932;149933;149934;149935;149936;149937;149938;149939;149940;149941;149942;149943;149944;149945;149946;149947;149948;149949;149950;149951;149952;149953;149954;149955;149956;149957;149958;149959;149960;149961;149962;149963;149964;149965;149966;149967;149968;149969;149970;149971;149972;149973;149974;149975;149976;149977;149978;149979;149980;149981;149982;149983;149984;149985;149986;149987;149988;149989;149990;149991;149992;149993;149994;149995;149996;149997;149998;149999;150000;150001;150002;150003;150004;150005;150006;150007;150008;150009;150010;150011;150012;150013;150014;150015;150016;150017;150018;150019;150020;150021;150022;150023;150024;150025;150026;150027;150028;150029;150030;150031;150032;150033;150034;150035;150036;150037;150038;150039;150040;150041;150042;150043;150044;150045;150046;150047;150048;150049;150050;150051;150052;150053;150054;150055;150056;150057;150058;150059;150060;150061;150062;150063;150064;150065;150066;150067;150068;150069;150070;150071;150072;150073;150074;150075;150076;150077;150078;150079;150080;150081;150082;150083;150084;150085;150086;150087;150088;150089;150090;150091;150092;150093;150094;150095;150096;150097;150098;150099;150100;150101;150102;150103;150104;150105;150106;150107;150108;150109;150110;150111;150112;150113;150114;150115;150116;150117;150118;150119;150120;150121;150122;150123;150124;150125;150126;150127;150128;150129;150130;150131;150132;150133;150134;150135;150136;150137;150138;150139;150140;150141;150142;150143;150144;150145;150146;150147;150148;150149;150150;150151;150152;150153;150154;150155;150156;150157;150158;150159;150160;150161;150162;150163;150164;150165;150166;150167;150168;150169;150170;150171;150172;150173;150174;150175;150176;150177;150178;150179;150180;150181;150182;150183;150184;150185;150186;150187;150188;150189;150190;150191;150192;150193;150194;150195;150196;150197;150198;150199;150200;150201;150202;150203;150204;150205;150206;150207;150208;150209;150210;150211;150212;150213;150214;150215;150216;150217;150218;150219;150220;150221;150222;150223;150224;150225;150226;150227;150228;150229;150230;150231;150232;150233;150234;150235;150236;150237;150238;150239;150240;150241;150242;150243;150244;150245;150246;150247;150248;150249;150250;150251;150252;150253;150254;150255;150256;150257;150258;150259;150260;150261;150262;150263;150264;150265;150266;150267;150268;150269;150270;150271;150272;150273;150274;150275;150276;150277;150278;150279;150280;150281;150282;150283;150284;150285;150286;150287;150288;150289;150290;150291;150292;150293;150294;150295;150296;150297;150298;150299;150300;150301;150302;150303;150304;150305;150306;150307;150308;150309;150310;150311;150312;150313;150314;150315;150316;150317;150318;150319;150320;150321;150322;150323;150324;150325;150326;150327;150328;150329;150330;150331;150332;150333;150334;150335;150336;150337;150338;150339;150340;150341;150342;150343;150344;150345;150346;150347;150348;150349;150350;150351;150352;150353;150354;150355;150356;150357;150358;150359;150360;150361;150362;150363;150364;150365;150366;150367;150368;150369;150370;150371;150372;150373;150374;150375;150376;150377;150378;150379;150380;150381;150382;150383;150384;150385;150386;150387;150388;150389;150390;150391;150392;150393;150394;150395;150396;150397;150398;150399;150400;150401;150402;150403;150404;150405;150406;150407;150408;150409;150410;150411;150412;150413;150414;150415;150416;150417;150418;150419;150420;150421;150422;150423;150424;150425;150426;150427;150428;150429;150430;150431;150432;150433;150434;150435;150436;150437;150438;150439;150440;150441;150442;150443;150444;150445;150446;150447;150448;150449;150450;150451;150452;150453;150454;150455;150456;150457;150458;150459;150460;150461;150462;150463;150464;150465;150466;150467;150468;150469;150470;150471;150472;150473;150474;150475;150476;150477;150478;150479;150480;150481;150482;150483;150484;150485;150486;150487;150488;150489;150490;150491;150492;150493;150494;150495;150496;150497;150498;150499;150500;150501;150502;150503;150504;150505;150506;150507;150508;150509;150510;150511;150512;150513;150514;150515;150516;150517;150518;150519;150520;150521;150522;150523;150524;150525;150526;150527;150528;150529;150530;150531;150532;150533;150534;150535;150536;150537;150538;150539;150540;150541;150542;150543;150544;150545;150546;150547;150548;150549;150550;150551;150552;150553;150554;150555;150556;150557;150558;150559;150560;150561;150562;150563;150564;150565;150566;150567;150568;150569;150570;150571;150572;150573;150574;150575;150576;150577;150578;150579;150580;150581;150582;150583;150584;150585;150586;150587;150588;150589;150590;150591;150592;150593;150594;150595;150596;150597;150598;150599;150600;150601;150602;150603;150604;150605;150606;150607;150608;150609;150610;150611;150612;150613;150614;150615;150616;150617;150618;150619;150620;150621;150622;150623;150624;150625;150626;150627;150628;150629;150630;150631;150632;150633;150634;150635;150636;150637;150638;150639;150640;150641;150642;150643;150644;150645;150646;150647;150648;150649;150650;150651;150652;150653;150654;150655;150656;150657;150658;150659;150660;150661;150662;150663;150664;150665;150666;150667;150668;150669;150670;150671;150672;150673;150674;150675;150676;150677;150678;150679;150680;150681;150682;150683;150684;150685;150686;150687;150688;150689;150690;150691;150692;150693;150694;150695;150696;150697;150698;150699;150700;150701;150702;150703;150704;150705;150706;150707;150708;150709;150710;150711;150712;150713;150714;150715;150716;150717;150718;150719;150720;150721;150722;150723;150724;150725;150726;150727;150728;150729;150730;150731;150732;150733;150734;150735;150736;150737;150738;150739;150740;150741;150742;150743;150744;150745;150746;150747;150748;150749;150750;150751;150752;150753;150754;150755;150756;150757;150758;150759;150760;150761;150762;150763;150764;150765;150766;150767;150768;150769;150770;150771;150772;150773;150774;150775;150776;150777;150778;150779;150780;150781;150782;150783;150784;150785;153048;153049;153050;153051;153052;153053;153054;153055;153056;153057;153058;153059;153060;153061;153062;153063;153064;153065;153066;153067;153068;153069;153070;153071;153072;153073;153074;153075;153076;153077;153078;153079;153080;153081;153082;153083;153084;153085;153086;153087;153088;153089;153090;153091;153092;153093;153094;153095;153096;153097;153098;153099;153100;153101;153102;153103;153104;153105;153106;153107;153108;153109;153110;153111;153112;153113;153114;153115;153116;153117;153118;153119;153120;153121;153122;153123;153124;153125;153126;153127;153128;153129;153130;153131;153132;153133;153134;153135;153136;153137;153138;153139;153140;153141;153142;153143;153144;153145;153146;153147;153148;153149;153150;153151;153152;153153;153154;153155;153156;153502;153503;153504;153505;153506;153507;153508;153509;153510;153511;153512;153513;153514;153515;153516;153517;153518;153519;153520;153521;153522;153523;153524;153525;153526;153527;153528;153529;153530;153531;153532;153533;153534;153535;153536;153537;153538;153539;153540;153541;153542;153543;153544;153545;153546;153547;153548;153549;153550;153551;153552;153553;153554;153555;153556;153557;153558;153559;153560;153561;153562;153563;153564;153565;153566;153567;153568;153569;153570;153571;153572;153573;153574;153575;153576;153577;153578;153579;153580;153581;153582;153583;153584;153585;153586;153587;153588;153589;153590;153591;153592;153593;153594;153595;153596;153597;153598;153599;153600;153601;153602;153603;153604;153605;153606;153607;153608;153609;153610;153611;153612;153613;153614;153615;153616;153617;153618;153619;153620;153621;153622;153623;153624;153625;153626;153627;153628;153629;153630;153631;153632;153633;153634;153635;153636;153637;153638;153639;153640;153641;153642;153643;153644;153645;153646;153647;153648;153649;153650;153651;153652;153653;153654;153655;153656;153657;153658;153659;153660;153661;153662;153663;153664;153665;153666;153667;153668;153669;153670;153671;153672;153673;153674;153675;153676;153677;153678;153679;153680;153681;153682;153683;153684;153685;153686;153687;153688;153689;153690;153691;153692;153693;153694;153695;153696;153697;153698;153699;153700;153701;153702;153703;153704;153705;153706;153707;153708;153709;153740;153741;153742;153743;153744;153745;153746;153747;153748;153749;153750;153751;153752;153753;153754;153755;153756;153757;153758;153759;153760;153761;154643;154644;154645;154646;154647;154648;154649;154650;154651;154652;154653;154654;154655;154656;154657;154658;154659;154660;154661;154662;154663;154664;154665;154666;154667;154668;154669;154670;154671;154672;154673;154674;154675;154676;154677;154678;154679;154680;154681;154682;154683;154684;154685;154686;154687;154688;154689;154690;154691;154692;154693;154694;154695;154696;154697;154698;154699;154700;154701;154702;154703;154704;154705;154706;154707;154708;154709;154710;154711;154712;154713;154714;154715;154716;154717;154718;154719;154720;154721;154722;154723;154724;154771;154772;154773;154774;154775;154776;154777;154778;154779;154780;154781;154782;154783;154784;154785;154786;154787;154788;154789;154790;154791;154792;154793;154794;154795;154796;154797;154798;154799;154800;154801;154802;154803;155179;155180;155181;155182;155183;155184;155185;155186;155187;155188;155189;155190;155191;155192;155193;155194;155195;155196;155197;155198;155199;155200;155201;155202;155203;155204;155205;155206;155207;155208;155209;155210;155211;155212;155213;155214;155215;155216;155217;155218;155219;155220;155221;155222;155223;155224;155225;155226;155227;155228;155229;155230;155231;155232;155233;155234;155235;155236;155237;155238;155239;155240;155241;155242;155243;156484;156485;156486;156487;156488;156489;156490;156491;156492;156493;156494;156495;156496;156497;156498;156499;156500;156501;156502;156503;156504;156505;156506;156507;156508;156509;156510;156511;156512;156513;156514;156515;156516;156517;156518;156519;156520;156521;156522;156791;156792;156793;156794;156795;156796;156797;156798;156799;156800;156801;156802;156803;156804;156805;156806;156807;156808;156809;156810;156811;157245;157246;157247;157248;157249;157250;157251;157252;157253;157254;157255;157256;157257;157258;157259;157260;157261;157262;157263;157264;157265;157266;157267;157268;157269;157270;157271;157272;157273;157274;157275;157276;157277;157278;157279;157280;157281;157282;157283;157284;157285;157286;157287;157288;157289;157290;157291;157292;157293;157294;157295;157296;157297;157298;157299;157300;157322;157323;157324;157325;157326;157327;157328;157329;157330;157331;157332;157333;157334;157335;157336;157337;157338;157339;157340;157341;157342;157343;157344;157345;157346;157347;157348;157349;157350;157351;157352;157353;157354;157355;157356;157357;157358;157359;157360;157361;157362;157363;157364;157365;157366;157367;157368;157369;157370;157371;157372;157373;157374;157375;157376;157377;157378;157379;157380;157381;157382;157383;157384;157385;157386;157387;157388;157389;157390;157391;157392;157393;157394;157395;157396;157397;157398;157399;157400;157401;157402;157403;157404;157405;157406;157407;157408;157409;157410;157411;157412;157413;157414;157415;157416;157417;157418;157419;157420;157421;157422;157423;157674;157675;157676;157677;157678;157679;157680;157681;157682;157683;157684;157685;157686;157687;157688;157689;157690;157691;157692;157693;157694;157695;157696;157697;157698;157699;157700;157701;157702;157703;157704;157705;157706;157707;157708;157709;157710;157711;157712;157713;157714;157715;157716;157717;157718;157719;157720;157721;157722;157723;157724;157725;157726;157727;157728;157729;157730;157731;157732;157733;157734;157735;157736;157737;157738;157739;157740;157741;157742;157743;157744;157745;157746;157954;157955;157956;157957;157958;157959;157960;157961;157962;157963;157964;157965;157966;157967;157968;157969;157970;157971;157972;157973;157974;157975;157976;157977;157978;157979;157980;157981;157982;157983;157984;157985;157986;157987;157988;157989;157990;157991;157992;157993;157994;157995;157996;157997;157998;157999;158000;158001;158002;158003;158004;158005;158006;158007;158008;158009;158010;158011;158012;158013;158014;158015;158016;158017;158018;158019;158020;158021;158022;158023;158024;158025;158026;158146;158147;158148;158149;158150;158151;158152;158153;158154;158155;158156;158157;158158;158159;158160;158161;158162;158163;158164;158165;158166;158167;158168;158169;158170;158171;158172;158173;158174;158175;158176;158177;158178;158179;158180;158181;158182;158183;158184;158185;158186;158187;158188;158189;158190;158191;158192;158193;158194;158195;158196;158197;158198;158199;158200;158201;158202;158203;158204;158205;158206;158207;158208;158209;158210;158211;158212;158213;158214;158215;158216;158217;158218;159090;159091;159092;159093;159094;159095;159096;159097;159098;159099;159100;159101;159102;159103;159104;159105;159106;159107;159108;159109;159110;159111;159112;159113;159114;159115;159116;159117;159118;159119;159120;159121;159122;159123;159124;159125;159126;159127;159128;159129;159130;159131;159132;159133;159134;159135;159136;159137;159138;159139;159140;159141;159142;159143;159144;159145;159146;159147;159148;159149;159150;159151;159152;159153;159154;159155;159156;159157;159158;159159;159160;159161;159162;159163;159164;159165;159166;159167;159168;159169;159170;159171;159172;159173;159174;159175;159176;159177;159178;159179;159180;159181;159182;159183;159184;159185;159186;159187;159188;159189;159190;159191;159192;159193;159194;159195;159196;159197;159198;159199;159200;159201;159202;159203;159204 1001;3201;3416;3434;3435;3436;3437;3438;3649;3782;4383;4384;4387;4480;4493;4497;4500;4507;4508;4934;4990;5147;7950;11528;11543;11599;12043;12404;12414;12422;12424;12429;12432;12444;25080;46310;46567;46652;46921;47011;47025;47087;47107;47691;48472;48588;48819;48859;49022;49086;49144;49273;49278;49390;50819;51489;51730;51735;51757;51773;51897;52203;52209;52292;52374;52732;52785;52794;53216;53237;53575;53579;53655;54646;54658;55796;55818;56022;56103;56132;56146;56197;56241;56258;56264;56274;56292;56297;56372;56381;56435;56469;56475;56482;56500;56538;56549;56690;56790;56990;57056;57158;57178;57200;57285;57366;57404;57513;57541;57628;57630;57728;57751;57807;57902;57949;58107;58150;58193;58320;58376;58472;58527;59293;59506;60332;66803;67232;67271;67336;67338;67386;69133;70099;70164;70304;70390;70527;70622;70654;70749;70816;70830;70867;70907;71038;71059;71151;71186;71300;77436;77848;81507;83656;83687;85792;86169;87905;89443;91541;91546;91573;91585;91592;92169;92177;93159;94213;94214;94216;94277;94338;94354;94374;94552;94597;95628;95685;96364;107055;116202;120038;126545;126591;126609;126681;133317;138928;138958;139059;139338;139434;139580;139634;139770;142772;146392;146410;146426;146428;146429;146473;146609;146619;146711;146725;146758;146832;147027;147028;148524;149846;149953;149995;150047;150117;150130;150172;150244;150287;150318;150345;150416;150483;150618;150672;150699;150785;153099;153140;153509;153517;153589;153617;153624;153644;153658;153751;154670;154706;154787;155203;155224;156484;156791;156811;157257;157344;157701;158019;158025;158210;159192 80;81;82;83;84;85;86;87;88;89;90;91;92;93;94;95;96;97;98;99;100;101;102;103;104;105;106;107;108;109;110;111;112;113;114;115;116;117;118;119;120;121;122;123;124;125;126;127;128;129;130;131;132;133;134;135;136;137;138;139;140;141;142;143;144;145;146;147 182;495;504;573;803;806;870;882;894;934;943;950;963;991;1003;1017;1062;1076;1082;1091;1180;1183;1192;1208;1259;1320;1336;1347;1448;1513;1539;1574;1629;1632;1769;1792;1859;1977;2051;2085;2246;2500;2562;2565;2569;2574;2684;2696;2789;2863;3098;3970;3993;4093;4100;4109;4132;4167;4295;4423;4457;4478;4662;4896;5026;5068;5117;5155

A0A0G2JUB0;Q810F4 A0A0G2JUB0;Q810F4 4;4 4;4 4;4 Protein FAM3C Fam3c tr|A0A0G2JUB0|A0A0G2JUB0_RAT Protein FAM3C OS=Rattus norvegicus OX=10116 GN=Fam3c PE=1 SV=1;sp|Q810F4|FAM3C_RAT Protein FAM3C OS=Rattus norvegicus OX=10116 GN=Fam3c PE=2 SV=1 2 4 4 4 0 1 2 4 2 3 0 1 2 4 2 3 0 1 2 4 2 3 22.9 22.9 22.9 24.734 227 227;227 9.07 14 1 0 6.7869 By MS/MS By MS/MS By MS/MS By MS/MS By MS/MS 0.99759 4.2959 17.268 12 5 Leave out requantified 1.613 4.7702 10.212 12 5 Leave out requantified 1.3987 0.96613 16.5 12 5 Leave out requantified NaN 0.67435 1.2789 1.3198 1.0426 0.94315 NaN 2.729 5.3203 5.9876 4.642 4.1673 NaN NaN NaN 20.072 11.095 21.495 0 1 1 4 2 4 0 1 0 1 1 2 Median Median Median Leave out requantified Median Median NaN 1.3498 1.9407 1.613 1.8333 1.5059 NaN 4.2745 6.5459 4.7326 5.5167 4.8326 NaN NaN NaN 1.754 21.629 6.783 0 1 1 4 2 4 0 1 0 1 1 2 Median Median Median Leave out requantified Median Median NaN 2.0017 1.5054 1.3539 1.7519 1.5878 NaN 1.5906 1.1077 0.92995 1.1978 1.194 NaN NaN NaN 15.682 11.056 23.692 0 1 1 4 2 4 0 1 0 1 1 2 Median Median Median Leave out requantified Median Median 0 5.3 11 22.9 8.8 17.2 32423000 8562400 9693300 14168000 0 0 0 0 710770 213080 181690 316010 1899400 416990 610160 872300 13547000 3448600 4495500 5603100 5934300 1658100 1557300 2718900 10332000 2825700 2848600 4657200 94 5038;6932;9615;10389 True;True;True;True 5301;7297;10200;11009 46081;46082;62277;62278;62279;62280;62281;62282;62283;86679;86680;86681;86682;93697;93698 66415;66416;66417;66418;66419;66420;91022;91023;91024;91025;91026;91027;91028;91029;91030;126028;126029;126030;126031;126032;126033;126034;135960;135961 66419;91027;126030;135960 148;149;150 73;93;147

F1LS48;A0A0G2JUB4;Q5XI22;D3ZKB6;A0A096MJY8 F1LS48;A0A0G2JUB4;Q5XI22;D3ZKB6;A0A096MJY8 2;2;2;1;1 2;2;2;1;1 2;2;2;1;1 Acetyl-CoA acetyltransferase, cytosolic RGD1562948;Acat2 tr|F1LS48|F1LS48_RAT Acetyl-CoA acetyltransferase, cytosolic OS=Rattus norvegicus OX=10116 GN=Acat2 PE=1 SV=2;tr|A0A0G2JUB4|A0A0G2JUB4_RAT Acetyl-CoA acetyltransferase, cytosolic OS=Rattus norvegicus OX=10116 GN=Acat2 PE=1 SV=1;sp|Q5XI22|THIC_RAT Acetyl-Co 5 2 2 2 0 0 0 0 1 1 0 0 0 0 1 1 0 0 0 0 1 1 9.6 9.6 9.6 41.232 397 397;399;397;397;397 7 2 0.0010116 2.4402 By MS/MS By MS/MS 0.58539 3.1088 NaN 1 1 Median NaN NaN NaN 0 0 Median NaN NaN NaN 0 0 Median NaN NaN NaN NaN 0.58539 NaN NaN NaN NaN NaN 3.1088 NaN NaN NaN NaN NaN NaN NaN 0 0 0 0 1 0 0 0 0 0 1 0 Median Median Median Median Median Median NaN NaN NaN NaN NaN NaN NaN NaN NaN NaN NaN NaN NaN NaN NaN NaN NaN NaN 0 0 0 0 0 0 0 0 0 0 0 0 Median Median Median Median Median Median NaN NaN NaN NaN NaN NaN NaN NaN NaN NaN NaN NaN NaN NaN NaN NaN NaN NaN 0 0 0 0 0 0 0 0 0 0 0 0 Median Median Median Median Median Median 0 0 0 0 6.8 2.8 8446300 6187000 2259400 0 0 0 0 0 0 0 0 0 0 0 0 0 0 0 0 0 7577100 5317700 2259400 0 869280 869280 0 0 95 1402;5978 True;True 1484;6306 12596;54294 18028;79926 18028;79926 151;152;153 108;119;122

A0A0G2K9I7;A0A0G2JVM0;A0A0G2JUB9;A0A0G2JUT5;P0C2X9 A0A0G2K9I7;A0A0G2JVM0;A0A0G2JUB9;A0A0G2JUT5;P0C2X9 1;1;1;1;1 1;1;1;1;1 1;1;1;1;1 Delta-1-pyrroline-5-carboxylate dehydrogenase, mitochondrial Aldh4a1 tr|A0A0G2K9I7|A0A0G2K9I7_RAT Taste receptor type 1 member 2 OS=Rattus norvegicus OX=10116 GN=Aldh4a1 PE=1 SV=1;tr|A0A0G2JVM0|A0A0G2JVM0_RAT Taste receptor type 1 member 2 OS=Rattus norvegicus OX=10116 GN=Aldh4a1 PE=1 SV=1;tr|A0A0G2JUB9|A0A0G2JUB9_RAT Taste 5 1 1 1 1 0 1 1 0 0 1 0 1 1 0 0 1 0 1 1 0 0 4 4 4 57.787 528 528;562;563;1463;563 5.33 2 1 0 5.5044 By MS/MS By MS/MS By MS/MS 0.6167 2.2469 80.429 2 2 Median 0.40983 1.0524 27.102 2 2 Median 0.66455 0.50485 49.943 2 2 Median 0.38655 NaN 0.98388 NaN NaN NaN 1.2723 NaN 3.9681 NaN NaN NaN NaN NaN NaN NaN NaN NaN 1 0 1 0 0 0 1 0 1 0 0 0 Median Median Median Median Median Median 0.37549 NaN 0.44731 NaN NaN NaN 0.86889 NaN 1.2747 NaN NaN NaN NaN NaN NaN NaN NaN NaN 1 0 1 0 0 0 1 0 1 0 0 0 Median Median Median Median Median Median 0.97139 NaN 0.45464 NaN NaN NaN 0.71868 NaN 0.35464 NaN NaN NaN NaN NaN NaN NaN NaN NaN 1 0 1 0 0 0 1 0 1 0 0 0 Median Median Median Median Median Median 4 0 4 4 0 0 9187100 6597500 1593300 996330 1277200 921710 120910 234550 0 0 0 0 4013800 1779600 1472400 761780 3896200 3896200 0 0 0 0 0 0 0 0 0 0 96 10999 True 11666 99938;99939;99940 145200;145201;145202 145201

A0A0G2K763;A0A0G2JUD4;A0A0G2K5C9 A0A0G2K763;A0A0G2JUD4;A0A0G2K5C9 1;1;1 1;1;1 1;1;1 tr|A0A0G2K763|A0A0G2K763_RAT Selenoprotein I (Fragment) OS=Rattus norvegicus OX=10116 GN=Selenoi PE=4 SV=1;tr|A0A0G2JUD4|A0A0G2JUD4_RAT Selenoprotein I OS=Rattus norvegicus OX=10116 GN=Selenoi PE=1 SV=1;tr|A0A0G2K5C9|A0A0G2K5C9_RAT Selenoprotein I OS=Rattu 3 1 1 1 1 1 1 1 1 1 1 1 1 1 1 1 1 1 1 1 1 1 13.8 13.8 13.8 13.292 116 116;384;394 2.1 7 7 3 3 0 133.6 By MS/MS By MS/MS By MS/MS By MS/MS By MS/MS By MS/MS 0.29095 0.75655 39.694 19 6 Median 0.31938 1.2031 31.706 19 6 Linear 1.0745 0.83247 12.839 19 6 Median 0.26976 0.30294 0.2938 0.25826 0.30644 0.2717 0.87184 0.94726 0.82119 0.86883 0.6205 0.58292 82.197 20.898 29.466 57.494 49.005 35.713 2 2 5 2 4 4 0 0 4 0 1 1 Median Median Median Median Median Median 0.36489 0.32709 0.36455 0.29973 0.28877 0.28226 0.73909 0.77459 0.83304 0.71663 0.67662 0.83607 36.703 31.334 25.816 44.163 33.722 37.09 2 2 5 2 4 4 0 0 4 0 1 1 Median Median Median Median Median Linear 1.3679 0.99344 1.2328 1.1825 0.98337 1.1611 1.0455 0.81641 0.94915 0.82137 0.77478 0.79563 17.819 8.4703 12.005 10.905 9.9113 4.8497 2 2 5 2 4 4 0 0 4 0 1 1 Median Median Median Median Median Linear 13.8 13.8 13.8 13.8 13.8 13.8 421460000 261230000 81055000 79172000 33587000 20088000 6428300 7070300 58548000 37328000 10635000 10585000 89289000 54181000 17109000 18000000 63494000 39073000 12842000 11580000 100140000 62287000 19337000 18512000 76404000 48274000 14704000 13426000 97 538 True 567;568 4753;4754;4755;4756;4757;4758;4759;4760;4761;4762;4763;4764;4765;4766;4767;4768;4769;4770;4771;4772 6702;6703;6704;6705;6706;6707;6708;6709;6710;6711;6712;6713;6714;6715;6716;6717;6718;6719;6720;6721;6722;6723;6724;6725;6726;6727;6728;6729;6730;6731;6732;6733;6734;6735;6736 6722

A0A0G2JUE4;A0A0G2K9I3;Q5U4E6 A0A0G2JUE4;A0A0G2K9I3;Q5U4E6 32;31;31 32;31;31 32;31;31 Golgin subfamily A member 4 Golga4 tr|A0A0G2JUE4|A0A0G2JUE4_RAT Golgin subfamily A member 4 OS=Rattus norvegicus OX=10116 GN=Golga4 PE=1 SV=1;tr|A0A0G2K9I3|A0A0G2K9I3_RAT Golgin subfamily A member 4 OS=Rattus norvegicus OX=10116 GN=Golga4 PE=1 SV=1;sp|Q5U4E6|GOGA4_RAT Golgin subfamily A mem 3 32 32 32 18 17 22 16 16 18 18 17 22 16 16 18 18 17 22 16 16 18 17.8 17.8 17.8 262.46 2281 2281;2213;2259 2.06 117 3 1 0 234.75 By MS/MS By MS/MS By MS/MS By MS/MS By MS/MS By MS/MS 0.65 1.3829 31.196 107 19 Leave out requantified 0.84612 1.5285 25.888 107 19 Leave out requantified 1.2954 0.98502 15.716 107 19 Leave out requantified 0.64174 0.62647 0.62888 0.67499 0.73955 0.66542 1.4174 1.3893 1.4885 1.3087 1.6322 1.3928 36.865 27.969 38.167 43.295 26.711 44.686 18 16 25 16 13 19 3 2 4 4 1 5 Leave out requantified Leave out requantified Leave out requantified Leave out requantified Leave out requantified Leave out requantified 0.75407 0.82762 0.80964 0.86343 0.98739 0.86487 1.3189 1.4546 1.622 1.5272 2.0787 1.7319 33.706 14.165 39.577 29.586 19.996 32.157 18 16 25 16 13 19 3 2 4 4 1 5 Leave out requantified Leave out requantified Leave out requantified Leave out requantified Leave out requantified Leave out requantified 1.1584 1.2988 1.2422 1.2767 1.2466 1.2821 0.82523 1.0312 0.99442 0.96187 0.96268 0.9992 30.028 17.606 21.381 24.457 17.758 18.443 18 16 25 16 13 19 3 2 4 4 1 5 Leave out requantified Leave out requantified Leave out requantified Leave out requantified Leave out requantified Leave out requantified 9.6 8.5 12.2 9.3 9 10 955920000 395890000 240570000 319460000 144850000 65421000 35430000 43995000 112200000 43615000 29519000 39061000 211980000 89877000 51587000 70519000 170050000 76894000 40316000 52843000 135370000 50268000 33994000 51112000 181470000 69813000 49729000 61926000 98 262;295;617;767;2139;2619;2620;2977;3061;3142;3195;3202;3236;3328;4838;5869;5893;6236;6661;7815;7881;8251;8450;8937;9115;9356;9597;10541;11274;11446;11583;11682 True;True;True;True;True;True;True;True;True;True;True;True;True;True;True;True;True;True;True;True;True;True;True;True;True;True;True;True;True;True;True;True 278;313;651;808;2264;2781;2782;3151;3239;3323;3378;3385;3422;3518;5091;6193;6217;6576;7016;8297;8367;8757;8965;9468;9665;9920;10179;11170;11956;12144;12290;12398 2446;2447;2448;2449;2450;2451;2452;2783;2784;2785;2786;5373;5374;6640;6641;6642;6643;19952;19953;19954;19955;19956;24765;24766;24767;24768;24769;24770;24771;24772;28124;28563;28564;28565;29173;29174;29175;29176;29177;29645;29646;29647;29648;29649;29667;29668;29669;29670;29920;29921;29922;29923;30556;43845;43846;53324;53325;53326;53327;53579;53580;53581;53582;53583;56514;56515;56516;56517;60085;60086;60087;60088;70854;70855;70856;70857;71667;71668;71669;71670;71671;71672;71673;71674;71675;74725;74726;74727;74728;74729;76301;76302;76303;76304;76305;76306;79639;81449;81450;81451;84019;84020;84021;84022;84023;84024;84025;86499;86500;94981;103041;103042;104745;106170;106171;106172;106173;106174;106175;107771;107772 3467;3468;3469;3470;3900;3901;7559;9471;9472;9473;9474;9475;28190;28191;34633;34634;34635;34636;34637;34638;34639;34640;34641;39680;40227;40228;40229;40230;40969;40970;40971;40972;41552;41553;41554;41555;41556;41557;41558;41559;41574;41575;41576;41913;41914;41915;41916;42937;62691;62692;77987;77988;77989;77990;78501;78502;78503;78504;78505;78506;78507;78508;78509;82954;82955;88106;88107;103173;103174;104320;104321;104322;104323;104324;104325;104326;104327;104328;104329;104330;104331;104332;104333;104334;108642;108643;108644;108645;108646;108647;110791;110792;110793;110794;110795;110796;110797;110798;110799;110800;115839;118451;118452;122267;122268;122269;122270;122271;122272;122273;122274;122275;125801;125802;138059;149695;149696;152403;154416;154417;154418;154419;154420;154421;154422;154423;156884 3467;3901;7559;9471;28191;34633;34638;39680;40230;40969;41552;41575;41915;42937;62691;77990;78501;82955;88107;103173;104326;108645;110793;115839;118452;122271;125801;138059;149695;152403;154422;156884 154;155;156;157 739;752;980;1376

A0A0G2JUF8;A0A0G2JWP8;P41516 A0A0G2JUF8;A0A0G2JWP8;P41516 6;6;6 6;6;6 6;6;6 DNA topoisomerase 2-alpha Top2a tr|A0A0G2JUF8|A0A0G2JUF8_RAT DNA topoisomerase 2 OS=Rattus norvegicus OX=10116 GN=Top2a PE=1 SV=1;tr|A0A0G2JWP8|A0A0G2JWP8_RAT DNA topoisomerase 2 OS=Rattus norvegicus OX=10116 GN=Top2a PE=1 SV=1;sp|P41516|TOP2A_RAT DNA topoisomerase 2-alpha OS=Rattus norv 3 6 6 6 1 1 2 1 5 3 1 1 2 1 5 3 1 1 2 1 5 3 5.8 5.8 5.8 172.43 1520 1520;1528;1526 2.8 3 12 0 19.538 By MS/MS By MS/MS By MS/MS By MS/MS By MS/MS By MS/MS 1.0207 2.2009 60.55 10 7 Median 0.91598 1.7714 60.474 10 7 Median 0.79043 0.63291 25.83 10 7 Median 0.75895 1.1834 1.1717 NaN 1.2791 1.7177 1.6918 2.7793 2.4575 NaN 2.3741 3.2855 NaN NaN 48.587 NaN 72.89 107.45 1 1 2 0 4 2 1 0 1 0 4 1 Median Median Median Median Median Median 0.46073 0.69654 1.4366 NaN 1.1051 1.628 0.75691 1.2675 2.3987 NaN 1.8254 2.9565 NaN NaN 50.977 NaN 60.634 64.337 1 1 2 0 4 2 1 0 1 0 4 1 Median Median Median Median Median Median 0.60707 0.67127 1.2234 NaN 0.78366 0.96419 0.47236 0.55902 1.0046 NaN 0.63291 0.75566 NaN NaN 0.29515 NaN 9.6453 27.4 1 1 2 0 4 2 1 0 1 0 4 1 Median Median Median Median Median Median 0.9 0.9 2.3 0.9 5.1 2.6 79773000 24072000 25473000 30228000 7508200 3332500 2394600 1781100 8495200 3070500 2909700 2515000 9883900 3646100 2675200 3562500 0 0 0 0 29803000 8630700 10917000 10256000 24083000 5392400 6576400 12114000 99 1073;2032;5055;7362;8149;12275 True;True;True;True;True;True 1138;2153;5318;7807;8653;13022 9446;18807;46188;66281;66282;66283;73938;73939;73940;73941;73942;73943;113615;113616;113617 13592;26546;66548;96525;96526;96527;96528;107584;107585;107586;107587;107588;107589;107590;107591;165184;165185;165186 13592;26546;66548;96525;107586;165185 158;159 126;493

A0A0G2JUJ9;D4A2Z6 A0A0G2JUJ9;D4A2Z6 13;12 13;12 13;12 Sec63 tr|A0A0G2JUJ9|A0A0G2JUJ9_RAT SEC63 homolog, protein translocation regulator OS=Rattus norvegicus OX=10116 GN=Sec63 PE=1 SV=1;tr|D4A2Z6|D4A2Z6_RAT SEC63 homolog, protein translocation regulator OS=Rattus norvegicus OX=10116 GN=Sec63 PE=1 SV=1 2 13 13 13 4 9 7 8 9 9 4 9 7 8 9 9 4 9 7 8 9 9 25.5 25.5 25.5 87.716 760 760;727 2.94 30 5 51 3 1 0 44.29 By MS/MS By MS/MS By MS/MS By MS/MS By MS/MS By MS/MS 0.54017 1.3645 25.179 74 31 Leave out requantified 0.58483 1.4043 22.01 75 31 Leave out requantified 1.0811 0.82887 21.062 74 31 Leave out requantified 0.54712 0.57899 0.51315 0.51254 0.60387 0.44845 1.9482 1.4863 1.2161 1.4243 2.0009 1.2464 11.834 29.316 25.275 23.562 28.591 16.311 6 10 9 11 19 19 1 3 3 6 11 7 Leave out requantified Leave out requantified Leave out requantified Leave out requantified Leave out requantified Leave out requantified 0.44778 0.52111 0.51808 0.60575 0.65152 0.61803 0.99285 1.1791 1.0026 1.4321 1.5523 1.3815 25.2 17.183 34.006 5.7786 19.232 28.822 6 10 9 11 19 20 1 3 3 6 11 7 Leave out requantified Leave out requantified Leave out requantified Leave out requantified Leave out requantified Leave out requantified 0.90236 0.95809 0.87752 1.4291 1.1452 1.3213 0.66793 0.74654 0.72206 0.99258 0.82443 1.0401 13.558 10.176 11.242 6.8796 14.047 15.555 6 9 9 11 19 20 1 3 3 6 11 7 Leave out requantified Leave out requantified Leave out requantified Leave out requantified Leave out requantified Leave out requantified 6.6 18.8 12.8 13.8 16.8 16.8 531670000 254290000 126790000 150590000 31653000 15374000 8101600 8177600 48436000 24580000 12353000 11503000 48643000 23647000 12703000 12293000 89395000 42739000 19216000 27441000 160080000 74716000 40151000 45216000 153460000 73231000 34264000 45963000 100 1536;1796;2398;3243;3255;4139;5848;6675;6752;8374;9298;9644;9718 True;True;True;True;True;True;True;True;True;True;True;True;True 1622;1898;2553;3429;3441;4369;6171;7030;7107;8886;9860;10231;10308 13691;13692;13693;13694;13695;13696;13697;13698;13699;13700;13701;13702;13703;13704;13705;13706;13707;13708;13709;13710;13711;13712;13713;13714;13715;13716;13717;16127;16128;22561;29939;29940;29941;29942;29943;29944;29945;29946;29947;29948;29949;30024;30025;30026;30027;30028;30029;37227;53131;53132;53133;53134;53135;53136;53137;60152;60153;60154;60155;60156;60732;60733;60734;75674;75675;75676;83440;83441;83442;83443;83444;83445;83446;83447;83448;83449;83450;87116;87766;87767;87768;87769;87770;87771;87772;87773;87774;87775;87776;87777 19581;19582;19583;19584;19585;19586;19587;19588;19589;19590;19591;19592;19593;19594;19595;19596;19597;19598;19599;19600;19601;19602;19603;19604;19605;19606;19607;19608;19609;19610;19611;19612;19613;22884;22885;31626;41933;41934;41935;41936;41937;41938;41939;41940;41941;41942;41943;41944;41945;41946;42052;42053;42054;42055;42056;42057;42058;42059;42060;42061;52519;77675;77676;77677;77678;77679;77680;77681;88184;88185;88186;88187;88188;88189;88190;88953;88954;88955;109990;109991;109992;121479;121480;121481;121482;121483;121484;121485;121486;121487;121488;126751;127673;127674;127675;127676;127677;127678;127679;127680;127681;127682;127683;127684;127685 19597;22884;31626;41934;42055;52519;77681;88188;88955;109991;121488;126751;127684 160;161 372;479

A0A0G2JUL7;B3GNI6;B5DFG5;A0A0U1RRT8;A0A0U1RRW2;G3V9Z6;A0A0G2JVY6;A0A0G2K7G7;B0BNF1 A0A0G2JUL7;B3GNI6 9;9;2;2;1;1;1;1;1 9;9;2;2;1;1;1;1;1 9;9;2;2;1;1;1;1;1 Septin-11 Sept11 tr|A0A0G2JUL7|A0A0G2JUL7_RAT Septin 6 (Predicted), isoform CRA_b OS=Rattus norvegicus OX=10116 GN=Sept11 PE=1 SV=1;sp|B3GNI6|SEP11_RAT Septin-11 OS=Rattus norvegicus OX=10116 GN=Sept11 PE=1 SV=1 9 9 9 9 5 4 4 4 2 7 5 4 4 4 2 7 5 4 4 4 2 7 22.1 22.1 22.1 49.341 429 429;431;427;432;264;429;431;483;442 5.89 4 32 0 100.7 By MS/MS By MS/MS By MS/MS By MS/MS By MS/MS By MS/MS 0.17739 0.91047 22.296 29 23 Leave out requantified 0.3938 1.2533 8.3848 29 23 Leave out requantified 2.0874 1.5585 34.901 29 23 Leave out requantified 0.16436 0.22474 0.21652 0.18487 0.23238 0.25597 0.68868 1.1667 1.0073 0.87727 1.104 1.107 65.364 36.062 12.886 42.635 15.344 25.507 6 4 4 5 3 7 5 4 3 4 2 5 Median Median Median Median Median Median 0.33138 0.28393 0.32812 0.36183 0.41523 0.38504 1.2435 1.0587 1.0296 1.2279 1.2877 1.187 25.301 6.3062 36.289 18.198 6.6444 19.747 6 4 4 5 3 7 5 4 3 4 2 5 Median Median Median Median Median Median 1.855 1.1962 1.6107 2.3202 1.7869 1.4907 1.3489 0.92023 1.2451 1.5052 1.2357 1.1884 59.16 38.792 24.811 43.736 17.678 23.017 6 4 4 5 3 7 5 4 3 4 2 5 Median Median Median Median Median Median 13.3 13.3 9.1 13.3 4.4 21.7 126390000 80033000 16600000 29760000 11697000 7859700 1045000 2792300 12525000 8026200 1520500 2978000 19616000 12744000 3086200 3786100 21459000 13005000 2203700 6249900 8016600 5076900 949810 1989900 53080000 33321000 7794900 11964000 101 26;27;28;1161;2396;2966;5715;9366;9935 True;True;True;True;True;True;True;True;True 28;29;30;1229;2551;3140;6030;9930;9931;10536 237;238;239;240;241;242;243;244;245;10141;22551;22552;22553;22554;22555;22556;28059;28060;28061;28062;28063;28064;28065;28066;51921;84082;84083;84084;84085;84086;84087;84088;84089;84090;84091;89567 335;336;337;338;339;340;341;342;343;14614;31617;31618;31619;31620;31621;31622;39595;39596;39597;39598;39599;39600;39601;39602;39603;75132;122344;122345;122346;122347;122348;122349;122350;122351;122352;122353;122354;122355;122356;130215 335;342;343;14614;31621;39601;75132;122346;130215 162 169

D3ZTJ0;A0A0G2JUM3 D3ZTJ0;A0A0G2JUM3 2;2 2;2 2;2 Protein XRP2 Rp2 tr|D3ZTJ0|D3ZTJ0_RAT Protein XRP2 OS=Rattus norvegicus OX=10116 GN=Rp2 PE=1 SV=3;tr|A0A0G2JUM3|A0A0G2JUM3_RAT RP2, ARL3 GTPase-activating protein OS=Rattus norvegicus OX=10116 GN=Rp2 PE=1 SV=1 2 2 2 2 1 1 1 1 2 1 1 1 1 1 2 1 1 1 1 1 2 1 8.6 8.6 8.6 39.32 348 348;409 3.5 6 3 1 0 5.1816 By MS/MS By MS/MS By MS/MS By MS/MS By MS/MS By MS/MS 0.32569 0.69782 47.518 7 4 Median 0.41172 0.77679 34.246 7 4 Median 1.2835 0.87686 17.309 7 4 Median 0.27789 0.33966 0.31299 0.32443 0.36078 0.34841 0.65211 0.67444 0.6929 1.5602 1.916 1.3884 NaN 4.8192 NaN NaN NaN NaN 1 2 1 1 1 1 0 2 1 0 0 1 Median Median Median Median Median Median 0.41172 0.43131 0.29302 0.37041 0.46784 0.40298 0.7103 0.7573 0.54906 1.2552 1.4133 1.0709 NaN 3.594 NaN NaN NaN NaN 1 2 1 1 1 1 0 2 1 0 0 1 Median Median Median Median Median Median 1.606 1.2698 0.9362 1.2835 1.5128 1.1566 1.2881 1.0788 0.78663 0.87091 0.87686 0.87594 NaN 2.6456 NaN NaN NaN NaN 1 2 1 1 1 1 0 2 1 0 0 1 Median Median Median Median Median Median 2.9 2.9 2.9 2.9 8.6 2.9 29029000 16950000 5042200 7037100 1846800 1074000 314570 458240 7970700 4429100 1343900 2197700 5274300 3376300 963420 934630 3753700 2203400 653620 896740 7553400 4433500 1260200 1859700 2630400 1433800 506520 690040 102 1186;3362 True;True 1255;3552 10367;10368;10369;10370;10371;10372;10373;10374;10375;30818 14912;14913;14914;14915;14916;14917;14918;14919;14920;14921;14922;14923;14924;14925;43287 14912;43287

A0A0G2JUN1 A0A0G2JUN1 1 1 1 tr|A0A0G2JUN1|A0A0G2JUN1_RAT SPATA31 subfamily D, member 3 OS=Rattus norvegicus OX=10116 GN=Spata31d3 PE=4 SV=1 1 1 1 1 1 0 0 1 0 0 1 0 0 1 0 0 1 0 0 1 0 0 1 1 1 146.04 1332 1332 4.5 1 1 1 -2 By MS/MS By matching 0.30908 1.1207 36.343 2 0 Median 0.45852 1.1294 30.008 2 0 Median 1.3189 0.94935 16.149 2 0 Median 0.23374 NaN NaN 0.40871 NaN NaN 0.86676 NaN NaN 1.4492 NaN NaN NaN NaN NaN NaN NaN NaN 1 0 0 1 0 0 0 0 0 0 0 0 Median Median Median Median Median Median 0.3806 NaN NaN 0.5524 NaN NaN 0.91349 NaN NaN 1.3964 NaN NaN NaN NaN NaN NaN NaN NaN 1 0 0 1 0 0 0 0 0 0 0 0 Median Median Median Median Median Median 1.551 NaN NaN 1.1215 NaN NaN 1.0642 NaN NaN 0.8469 NaN NaN NaN NaN NaN NaN NaN NaN 1 0 0 1 0 0 0 0 0 0 0 0 Median Median Median Median Median Median 1 0 0 1 0 0 9142600 5244300 1626600 2271700 4567500 2661200 810630 1095800 0 0 0 0 0 0 0 0 4575000 2583100 815980 1175900 0 0 0 0 0 0 0 0 + 103 7330 True 7765 65974;65975 96028 96028 163 1317

A0A0G2JUN7;R9PXU4;O89049 A0A0G2JUN7;R9PXU4;O89049 1;1;1 1;1;1 1;1;1 Thioredoxin reductase 1, cytoplasmic Txnrd1 tr|A0A0G2JUN7|A0A0G2JUN7_RAT Thioredoxin reductase 1, cytoplasmic OS=Rattus norvegicus OX=10116 GN=Txnrd1 PE=1 SV=1;tr|R9PXU4|R9PXU4_RAT Thioredoxin reductase 1, cytoplasmic OS=Rattus norvegicus OX=10116 GN=Txnrd1 PE=3 SV=2;sp|O89049|TRXR1_RAT Thioredoxin 3 1 1 1 0 0 0 0 0 1 0 0 0 0 0 1 0 0 0 0 0 1 4 4 4 54.498 497 497;498;499 6 1 0.0096534 1.479 By MS/MS NaN NaN NaN 0 0 Median NaN NaN NaN 0 0 Median NaN NaN NaN 0 0 Median NaN NaN NaN NaN NaN NaN NaN NaN NaN NaN NaN NaN NaN NaN NaN NaN NaN NaN 0 0 0 0 0 0 0 0 0 0 0 0 Median Median Median Median Median Median NaN NaN NaN NaN NaN NaN NaN NaN NaN NaN NaN NaN NaN NaN NaN NaN NaN NaN 0 0 0 0 0 0 0 0 0 0 0 0 Median Median Median Median Median Median NaN NaN NaN NaN NaN NaN NaN NaN NaN NaN NaN NaN NaN NaN NaN NaN NaN NaN 0 0 0 0 0 0 0 0 0 0 0 0 Median Median Median Median Median Median 0 0 0 0 0 4 1829400 1829400 0 0 0 0 0 0 0 0 0 0 0 0 0 0 0 0 0 0 0 0 0 0 1829400 1829400 0 0 104 10057 True 10660 90663 131675 131675

A0A0G2JUQ3;Q5FVM3 A0A0G2JUQ3;Q5FVM3 1;1 1;1 1;1 Protein FAM134B Fam134b tr|A0A0G2JUQ3|A0A0G2JUQ3_RAT Reticulophagy regulator 1 OS=Rattus norvegicus OX=10116 GN=LOC103689968 PE=1 SV=1;sp|Q5FVM3|RETR1_RAT Reticulophagy regulator 1 OS=Rattus norvegicus OX=10116 GN=Retreg1 PE=2 SV=1 2 1 1 1 0 1 1 1 1 1 0 1 1 1 1 1 0 1 1 1 1 1 3.5 3.5 3.5 38.076 347 347;480 3 5 2 2 0 10.974 By MS/MS By MS/MS By MS/MS By MS/MS By MS/MS NaN NaN NaN 0 0 Median NaN NaN NaN 0 0 Median NaN NaN NaN 0 0 Median NaN NaN NaN NaN NaN NaN NaN NaN NaN NaN NaN NaN NaN NaN NaN NaN NaN NaN 0 0 0 0 0 0 0 0 0 0 0 0 Median Median Median Median Median Median NaN NaN NaN NaN NaN NaN NaN NaN NaN NaN NaN NaN NaN NaN NaN NaN NaN NaN 0 0 0 0 0 0 0 0 0 0 0 0 Median Median Median Median Median Median NaN NaN NaN NaN NaN NaN NaN NaN NaN NaN NaN NaN NaN NaN NaN NaN NaN NaN 0 0 0 0 0 0 0 0 0 0 0 0 Median Median Median Median Median Median 0 3.5 3.5 3.5 3.5 3.5 0 0 0 0 0 0 0 0 0 0 0 0 0 0 0 0 0 0 0 0 0 0 0 0 0 0 0 0 105 9800 True 10394 88294;88295;88296;88297;88298;88299;88300;88301;88302 128393;128394;128395;128396;128397;128398;128399;128400;128401;128402 128401

A0A0G2JUS4;E9PTG1 A0A0G2JUS4;E9PTG1 3;3 2;2 2;2 Smarca2 tr|A0A0G2JUS4|A0A0G2JUS4_RAT SWI/SNF-related, matrix-associated, actin-dependent regulator of chromatin, subfamily a, member 2 OS=Rattus norvegicus OX=10116 GN=Smarca2 PE=1 SV=1;tr|E9PTG1|E9PTG1_RAT SWI/SNF-related, matrix-associated, actin-dependent regul 2 3 2 2 2 2 1 0 3 2 2 2 1 0 2 2 2 2 1 0 2 2 2.2 1.5 1.5 170.39 1488 1488;1597 1.79 7 3 4 0.001995 2.3465 By MS/MS By MS/MS By MS/MS By MS/MS By MS/MS 1.5698 3.4334 9.9522 11 3 Leave out requantified 1.383 2.4066 7.8659 12 3 Leave out requantified 0.89631 0.72251 5.9355 12 3 Leave out requantified 1.616 1.3446 1.4661 NaN 1.6206 1.3519 4.29 3.024 3.2627 NaN 3.4222 2.5549 30.148 5.266 NaN NaN 18.849 8.9792 2 2 1 0 4 2 1 0 0 0 1 1 Median Median Median Median Median Median 1.4452 1.0454 1.6286 NaN 1.3965 1.3576 2.6625 1.8705 3.0618 NaN 2.4508 2.1505 38.071 1.8397 NaN NaN 7.6997 20.512 3 2 1 0 4 2 1 0 0 0 1 1 Median Median Median Median Median Median 1.072 0.78108 1.1735 NaN 0.79792 1.0718 0.84 0.65669 0.9856 NaN 0.65006 0.83927 25.136 7.5728 NaN NaN 15.527 3.2415 3 2 1 0 4 2 1 0 0 0 1 1 Median Median Median Median Median Median 1.5 1.5 0.6 0 2.2 1.5 80998000 21600000 30302000 29097000 15523000 3335500 5633700 6554300 14174000 4657200 5121100 4395500 5393800 1349100 1902900 2141700 0 0 0 0 32763000 8423400 13165000 11175000 13144000 3834400 4479200 4830300 106 5183;11259;11362 True;False;True 5452;11941;12053 47034;47035;47036;47037;47038;47039;47040;47041;102802;104108;104109;104110;104111;104112;104113 67775;67776;67777;67778;67779;67780;67781;67782;67783;149339;151482;151483;151484 67777;149339;151484

A0A0G2JUV4;A0A0G2JWU3;F1M9A4;D3ZFE9 A0A0G2JUV4;A0A0G2JWU3;F1M9A4;D3ZFE9 2;2;2;2 2;2;2;2 2;2;2;2 Mrpl1 tr|A0A0G2JUV4|A0A0G2JUV4_RAT Mitochondrial ribosomal protein L1 OS=Rattus norvegicus OX=10116 GN=Mrpl1 PE=4 SV=1;tr|A0A0G2JWU3|A0A0G2JWU3_RAT Mitochondrial ribosomal protein L1-like OS=Rattus norvegicus OX=10116 GN=LOC100359687 PE=1 SV=1;tr|F1M9A4|F1M9A4_R 4 2 2 2 2 1 2 2 1 1 2 1 2 2 1 1 2 1 2 2 1 1 9.4 9.4 9.4 33.443 298 298;312;321;328 8.09 1 8 2 0.0029014 2.1499 By MS/MS By MS/MS By MS/MS By MS/MS By MS/MS By MS/MS 0.29632 1.4368 46.989 6 6 Median 0.33775 1.1094 20.653 4 4 Median 1.241 0.88314 17.646 4 4 Median 0.26078 0.2873 0.24236 NaN 0.47916 0.51978 1.4578 1.3615 1.1342 NaN 2.5143 2.3519 NaN NaN NaN NaN NaN 71.74 1 1 1 0 1 2 1 1 1 0 1 2 Median Median Median Median Median Median 0.40979 0.25698 0.29544 NaN NaN 0.38613 1.4076 0.8941 0.99233 NaN NaN 1.2403 NaN NaN NaN NaN NaN NaN 1 1 1 0 0 1 1 1 1 0 0 1 Median Median Median Median Median Median 1.5714 0.89449 1.219 NaN NaN 1.2634 1.0297 0.69754 0.80783 NaN NaN 0.96547 NaN NaN NaN NaN NaN NaN 1 1 1 0 0 1 1 1 1 0 0 1 Median Median Median Median Median Median 9.4 5.4 9.4 9.4 5.4 5.4 12119000 9267100 1326500 1525300 1362200 1004400 145580 212140 2218700 1667700 302580 248430 2274900 1763100 173380 338330 1380300 1380300 0 0 2221200 1755500 221850 243830 2661700 1696000 483140 482540 107 10088;11040 True;True 10692;11710 90867;90868;90869;90870;90871;90872;90873;90874;100467;100468;100469 131954;131955;131956;131957;131958;131959;131960;131961;146030;146031;146032 131959;146032 164 80

F1LZG4;A0A0G2JUV8;P11275 F1LZG4;A0A0G2JUV8;P11275 3;3;3 1;1;1 1;1;1 Calcium/calmodulin-dependent protein kinase type II subunit alpha Camk2a tr|F1LZG4|F1LZG4_RAT Calcium/calmodulin-dependent protein kinase type II subunit alpha OS=Rattus norvegicus OX=10116 GN=Camk2a PE=1 SV=3;tr|A0A0G2JUV8|A0A0G2JUV8_RAT Calcium/calmodulin-dependent protein kinase type II subunit alpha OS=Rattus norvegicus OX= 3 3 1 1 1 1 2 1 1 2 0 0 1 0 0 0 0 0 1 0 0 0 8.4 3.4 3.4 54.042 475 475;475;478 10 1 0.0092756 1.5051 By matching By matching By MS/MS By matching By matching By matching NaN NaN NaN 0 0 Median NaN NaN NaN 0 0 Median NaN NaN NaN 0 0 Median NaN NaN NaN NaN NaN NaN NaN NaN NaN NaN NaN NaN NaN NaN NaN NaN NaN NaN 0 0 0 0 0 0 0 0 0 0 0 0 Median Median Median Median Median Median NaN NaN NaN NaN NaN NaN NaN NaN NaN NaN NaN NaN NaN NaN NaN NaN NaN NaN 0 0 0 0 0 0 0 0 0 0 0 0 Median Median Median Median Median Median NaN NaN NaN NaN NaN NaN NaN NaN NaN NaN NaN NaN NaN NaN NaN NaN NaN NaN 0 0 0 0 0 0 0 0 0 0 0 0 Median Median Median Median Median Median 4 4 8.4 4 4 5.1 0 0 0 0 0 0 0 0 0 0 0 0 0 0 0 0 0 0 0 0 0 0 0 0 0 0 0 0 108 458;459;3343 False;False;True 479;480;3533 4054;4055;4056;4057;4058;4059;4060;4061;4062;4063;4064;4065;4066;4067;4068;4069;30658 5725;5726;5727;5728;5729;5730;5731;5732;5733;5734;5735;5736;5737;5738;5739;5740;5741;5742;5743;5744;5745;5746;5747;5748;5749;5750;5751;43068 5725;5749;43068

A0A0H2UHK5;A0A0G2JYN3;A0A0G2JUW8;P97629 A0A0H2UHK5;A0A0G2JYN3;A0A0G2JUW8;P97629 5;5;5;5 5;5;5;5 5;5;5;5 Leucyl-cystinyl aminopeptidase Lnpep tr|A0A0H2UHK5|A0A0H2UHK5_RAT Aminopeptidase OS=Rattus norvegicus OX=10116 GN=Lnpep PE=1 SV=1;tr|A0A0G2JYN3|A0A0G2JYN3_RAT Leucyl-cystinyl aminopeptidase OS=Rattus norvegicus OX=10116 GN=Lnpep PE=1 SV=1;tr|A0A0G2JUW8|A0A0G2JUW8_RAT Leucyl-cystinyl aminopept 4 5 5 5 0 1 4 2 1 3 0 1 4 2 1 3 0 1 4 2 1 3 5.8 5.8 5.8 103.19 900 900;1011;1024;1025 3 11 0 5.8832 By MS/MS By MS/MS By MS/MS By MS/MS By MS/MS 0.31488 0.62605 28.922 10 8 Median 0.27759 0.46506 27.603 8 6 Median 0.90958 0.71076 47.034 8 6 Median NaN 0.26253 0.21549 0.31488 0.35231 0.36621 NaN 0.4767 0.41676 0.62605 0.88657 0.67141 NaN NaN 34.364 1.5707 NaN 25.105 0 1 3 2 1 3 0 1 2 1 1 3 Median Median Median Median Median Median NaN 0.28415 0.37701 0.18853 0.23246 0.28809 NaN 0.45401 0.61291 0.32338 0.39081 0.51462 NaN NaN 23.734 3.0185 NaN 10.924 0 1 2 2 1 2 0 1 1 1 1 2 Median Median Median Median Median Median NaN 1.0823 1.5953 0.59957 0.65982 0.90958 NaN 0.93719 1.3216 0.46969 0.46307 0.71076 NaN NaN 41.141 4.4271 NaN 11.398 0 1 2 2 1 2 0 1 1 1 1 2 Median Median Median Median Median Median 0 1 5.8 2 2.2 4.7 53537000 35582000 9387200 8567500 0 0 0 0 1923700 1432400 150110 341150 15022000 10215000 2106200 2700600 11093000 7587400 2148900 1356700 4143800 2752000 855670 536170 21355000 13595000 4126400 3633000 109 3033;4017;5901;8739;9873 True;True;True;True;True 3209;4243;6226;9266;10469 28439;28440;36251;36252;53671;53672;78084;78085;78086;78087;88841 40061;40062;51159;51160;78701;78702;113546;113547;113548;113549;129213 40061;51160;78701;113549;129213

F1MAA1;A0A0G2JUX4 F1MAA1;A0A0G2JUX4 3;3 3;3 3;3 Ubiquitin carboxyl-terminal hydrolase Usp47 tr|F1MAA1|F1MAA1_RAT Ubiquitin-specific peptidase 47 OS=Rattus norvegicus OX=10116 GN=Usp47 PE=1 SV=1;tr|A0A0G2JUX4|A0A0G2JUX4_RAT Ubiquitin-specific peptidase 47 OS=Rattus norvegicus OX=10116 GN=Usp47 PE=1 SV=1 2 3 3 3 0 0 2 0 1 2 0 0 2 0 1 2 0 0 2 0 1 2 1.9 1.9 1.9 154.69 1355 1355;1375 3 5 0 4.6292 By MS/MS By MS/MS By MS/MS 0.79056 1.7119 46.127 5 3 Median 0.80466 1.3517 16.864 5 3 Median 1.0267 0.74036 37.391 5 3 Median NaN NaN 0.91659 NaN 0.68449 0.56281 NaN NaN 1.9732 NaN 1.6925 1.065 NaN NaN 3.3457 NaN NaN 67.118 0 0 2 0 1 2 0 0 1 0 0 2 Median Median Median Median Median Median NaN NaN 0.86762 NaN 0.80004 0.72449 NaN NaN 1.403 NaN 1.4386 1.0447 NaN NaN 5.266 NaN NaN 1.4779 0 0 2 0 1 2 0 0 1 0 0 2 Median Median Median Median Median Median NaN NaN 0.94744 NaN 1.22 1.2873 NaN NaN 0.73628 NaN 0.84605 0.99039 NaN NaN 0.78231 NaN NaN 68.643 0 0 2 0 1 2 0 0 1 0 0 2 Median Median Median Median Median Median 0 0 1 0 1 1.9 36444000 14889000 10016000 11539000 0 0 0 0 0 0 0 0 16424000 6976600 4728200 4719000 0 0 0 0 9614100 3389000 2899400 3325700 10406000 4523600 2388300 3494600 110 9412;9959;9960 True;True;True 9979;10560;10561 84800;89868;89869;89870;89871 123488;130629;130630;130631;130632;130633;130634 123488;130629;130632

A0A0G2JV31;O54975 A0A0G2JV31;O54975 2;2 2;2 2;2 Xaa-Pro aminopeptidase 1;Xaa-Pro aminopeptidase 1, N-terminally processed Xpnpep1 tr|A0A0G2JV31|A0A0G2JV31_RAT X-prolyl aminopeptidase (Aminopeptidase P) 1, soluble, isoform CRA_a OS=Rattus norvegicus OX=10116 GN=Xpnpep1 PE=1 SV=1;sp|O54975|XPP1_RAT Xaa-Pro aminopeptidase 1 OS=Rattus norvegicus OX=10116 GN=Xpnpep1 PE=1 SV=1 2 2 2 2 0 1 1 0 1 0 0 1 1 0 1 0 0 1 1 0 1 0 4.8 4.8 4.8 74.667 666 666;623 4.67 1 2 0 3.3486 By MS/MS By MS/MS By MS/MS 0.32523 0.96418 37.076 3 3 Median 0.30981 0.74714 24.986 3 3 Median 1.4129 0.98888 22.352 3 3 Plateau NaN 0.35757 0.32523 NaN 0.17465 NaN NaN 0.98082 0.96418 NaN 0.51175 NaN NaN NaN NaN NaN NaN NaN 0 1 1 0 1 0 0 1 1 0 1 0 Median Median Median Median Median Median NaN 0.30981 0.44641 NaN 0.24676 NaN NaN 0.74714 1.0225 NaN 0.62407 NaN NaN NaN NaN NaN NaN NaN 0 1 1 0 1 0 0 1 1 0 1 0 Median Median Median Median Median Median NaN 0.86643 1.3726 NaN 1.4129 NaN NaN 0.6915 1.0431 NaN 0.98888 NaN NaN NaN NaN NaN NaN NaN 0 1 1 0 1 0 0 1 1 0 1 0 Median Median Median Median Median Median 0 3.3 3.3 0 1.5 0 7638000 5295800 885350 1456800 0 0 0 0 2269400 1567600 287820 413940 2794600 1842700 419060 532770 0 0 0 0 2574100 1885500 178470 510130 0 0 0 0 111 7087;12197 True;True 7480;12937 63729;112866;112867 92896;164156;164157 92896;164156

G3V752;A0A0G2JV51;D4AEB4 G3V752;A0A0G2JV51 9;9;2 9;9;2 9;9;2 Nat10 tr|G3V752|G3V752_RAT RNA cytidine acetyltransferase OS=Rattus norvegicus OX=10116 GN=Nat10 PE=1 SV=1;tr|A0A0G2JV51|A0A0G2JV51_RAT RNA cytidine acetyltransferase OS=Rattus norvegicus OX=10116 GN=Nat10 PE=1 SV=1 3 9 9 9 4 5 8 5 7 7 4 5 8 5 7 7 4 5 8 5 7 7 8.1 8.1 8.1 115.33 1024 1024;1024;909 1 37 0 23.186 By MS/MS By MS/MS By MS/MS By MS/MS By MS/MS By MS/MS 0.38588 1.1135 23.411 36 13 Leave out requantified 0.6661 1.5912 17.646 36 13 Leave out requantified 1.6106 1.2724 17.332 36 13 Leave out requantified 0.32431 0.4426 0.38047 0.3423 0.39917 0.45608 0.91727 1.1674 1.1184 0.92909 1.079 1.3212 15.921 27.042 39.467 50.102 16.529 22.417 4 5 8 5 7 7 3 3 3 2 2 0 Median Median Leave out requantified Leave out requantified Leave out requantified Leave out requantified 0.5994 0.55372 0.6647 0.70908 0.72535 0.65254 1.6134 1.2359 1.3566 1.7996 1.5445 1.7221 31.289 23.989 21.714 47.507 12.338 21.146 4 5 8 5 7 7 3 3 3 2 2 0 Median Median Leave out requantified Leave out requantified Leave out requantified Leave out requantified 1.7896 1.2776 1.6088 2.2493 1.7943 1.3399 1.3167 1.0098 1.3503 1.5534 1.4829 0.95011 31.216 20.36 16.886 26.831 9.7874 15.351 4 5 8 5 7 7 3 3 3 2 2 0 Median Median Leave out requantified Leave out requantified Leave out requantified Leave out requantified 3.4 5.8 7 4 5.2 6.6 399010000 191540000 78571000 128900000 32201000 16589000 5290300 10322000 47027000 23016000 10397000 13614000 81837000 39053000 18318000 24465000 67398000 32591000 10631000 24176000 95861000 45551000 17593000 32717000 74686000 34743000 16341000 23602000 112 505;581;582;2178;2500;6138;9962;10506;10507 True;True;True;True;True;True;True;True;True 531;613;614;2312;2660;6475;10563;11133;11134 4450;4451;4452;4453;4454;5169;5170;5171;5172;5173;5174;5175;5176;5177;5178;5179;20409;20410;20411;23682;23683;23684;23685;55656;89873;89874;94670;94671;94672;94673;94674;94675;94676;94677;94678;94679;94680 6277;6278;6279;6280;6281;7314;7315;7316;7317;7318;7319;7320;7321;7322;7323;7324;7325;7326;7327;7328;28766;28767;28768;28769;28770;33165;33166;33167;33168;81685;130636;130637;137555;137556;137557;137558;137559;137560;137561;137562;137563;137564;137565;137566;137567;137568;137569;137570 6280;7318;7324;28768;33165;81685;130636;137562;137568 165;166;167 918;926;958

A0A0G2JV54 A0A0G2JV54 3 1 1 tr|A0A0G2JV54|A0A0G2JV54_RAT Polypyrimidine tract-binding protein 3 OS=Rattus norvegicus OX=10116 GN=Ptbp3 PE=1 SV=1 1 3 1 1 1 2 0 0 2 0 0 0 0 0 1 0 0 0 0 0 1 0 5.2 2.5 2.5 59.809 554 554 1 1 1 -2 By matching By matching By MS/MS 0.66051 1.0999 NaN 1 1 Median 0.5866 0.70816 NaN 1 1 Median 0.8881 0.75219 NaN 1 1 Median NaN NaN NaN NaN 0.66051 NaN NaN NaN NaN NaN 1.0999 NaN NaN NaN NaN NaN NaN NaN 0 0 0 0 1 0 0 0 0 0 1 0 Median Median Median Median Median Median NaN NaN NaN NaN 0.5866 NaN NaN NaN NaN NaN 0.70816 NaN NaN NaN NaN NaN NaN NaN 0 0 0 0 1 0 0 0 0 0 1 0 Median Median Median Median Median Median NaN NaN NaN NaN 0.8881 NaN NaN NaN NaN NaN 0.75219 NaN NaN NaN NaN NaN NaN NaN 0 0 0 0 1 0 0 0 0 0 1 0 Median Median Median Median Median Median 1.3 2.7 0 0 3.8 0 1175200 596620 296500 282120 0 0 0 0 0 0 0 0 0 0 0 0 0 0 0 0 1175200 596620 296500 282120 0 0 0 0 + 113 6757;6963;11819 False;True;False 7112;7337;12544 60765;60766;60767;62669;109548 88999;89000;91518;159564 88999;91518;159564 168 1

A0A0G2K5Q3;A0A0G2JV69 A0A0G2K5Q3;A0A0G2JV69 2;2 2;2 2;2 tr|A0A0G2K5Q3|A0A0G2K5Q3_RAT Mediator complex subunit 12-like (Fragment) OS=Rattus norvegicus OX=10116 GN=Med12l PE=4 SV=1;tr|A0A0G2JV69|A0A0G2JV69_RAT Mediator complex subunit 12-like (Fragment) OS=Rattus norvegicus OX=10116 GN=Med12l PE=4 SV=1 2 2 2 2 1 1 1 1 1 2 1 1 1 1 1 2 1 1 1 1 1 2 1.7 1.7 1.7 131.39 1177 1177;1746 2.28 6 4 5 3 0.0024765 2.307 By MS/MS By MS/MS By MS/MS By MS/MS By MS/MS By MS/MS 0.17019 0.52919 50.98 18 6 Median 0.26612 0.83928 30.606 18 6 Plateau 1.5546 1.1703 35.44 18 6 Median 0.16894 0.21408 0.17019 0.14408 0.18904 0.50054 0.62905 0.65744 0.5086 0.34935 0.6643 2.604 24.024 4.0077 32.338 19.527 33.302 101.56 2 2 4 5 2 3 0 0 0 1 2 3 Median Median Median Median Median Plateau 0.26934 0.22589 0.35452 0.32484 0.22979 0.29916 0.64311 0.66746 1.197 0.95701 0.62033 0.86073 3.3298 18.443 42.986 35.801 43.115 37.699 2 2 4 5 2 3 0 0 0 1 2 3 Median Median Linear Plateau Median Median 1.5763 1.0173 1.5546 2.0824 1.2156 1.2517 1.0682 0.79572 1.1982 1.3874 0.92613 0.88564 29.653 2.1715 9.5725 15.031 9.5982 66.061 2 2 4 5 2 3 0 0 0 1 2 3 Median Median Median Median Median Median 0.8 0.8 0.8 0.8 0.8 1.7 670780000 437200000 105670000 127910000 97638000 65415000 13427000 18796000 91469000 62209000 15167000 14093000 123430000 83152000 15938000 24340000 171010000 114950000 18483000 37580000 33973000 23166000 4772900 6034400 153260000 88313000 37881000 27062000 114 5002;5091 True;True 5262;5355 45671;46343;46344;46345;46346;46347;46348;46349;46350;46351;46352;46353;46354;46355;46356;46357;46358;46359 65813;66751;66752;66753;66754;66755;66756;66757;66758;66759;66760;66761;66762;66763;66764;66765;66766;66767;66768;66769;66770 65813;66759

A0A0G2JV81;A0A0G2K9H8;P84082;P84079;P61206;A0A0G2K4Q4 A0A0G2JV81;A0A0G2K9H8;P84082;P84079;P61206;A0A0G2K4Q4 3;3;3;3;3;2 3;3;3;3;3;2 2;2;2;2;2;1 ADP-ribosylation factor 2;ADP-ribosylation factor 1;ADP-ribosylation factor 3 Arf2;Arf1;Arf3 tr|A0A0G2JV81|A0A0G2JV81_RAT FKBP prolyl isomerase 11 OS=Rattus norvegicus OX=10116 GN=Arf3 PE=3 SV=1;tr|A0A0G2K9H8|A0A0G2K9H8_RAT ADP-ribosylation factor 3 OS=Rattus norvegicus OX=10116 GN=Arf3 PE=3 SV=1;sp|P84082|ARF2_RAT ADP-ribosylation factor 2 OS=Rat 6 3 3 2 1 1 1 2 2 1 1 1 1 2 2 1 1 1 1 1 2 1 27.6 27.6 16 20.568 181 181;187;181;181;181;180 10.5 1 7 6 1 0 36.333 By MS/MS By MS/MS By MS/MS By MS/MS By MS/MS By MS/MS 0.30639 1.5979 30.15 10 3 Median 0.39844 1.2153 20.442 10 3 Median 1.2689 0.87557 20.879 10 3 Median 0.29065 NaN 0.35195 0.40008 0.36875 0.3022 1.7287 NaN 1.6686 2.0522 1.7598 1.3487 NaN NaN 38.82 28.678 62.028 12.851 1 0 2 3 2 2 0 0 1 2 0 0 Median Median Median Median Median Median 0.31415 NaN 0.44369 0.4104 0.40564 0.34601 1.0304 NaN 1.4883 1.3159 1.2153 0.9458 NaN NaN 35.529 3.3298 0.26902 0.061915 1 0 2 3 2 2 0 0 1 2 0 0 Median Median Median Median Median Median 1.1051 NaN 1.3019 1.0258 1.4889 1.1885 0.73028 NaN 0.91926 0.70768 1.0212 0.84464 NaN NaN 7.1245 34.441 21.519 10.688 1 0 2 3 2 2 0 0 1 2 0 0 Median Median Median Median Median Median 7.7 7.7 7.7 19.3 16 7.7 30146000 18266000 5036000 6843600 1222300 795450 178510 248370 0 0 0 0 2562900 1486300 429510 647120 13276000 8235800 1952000 3088000 6169000 3462000 1231800 1475200 6915800 4286800 1244100 1384900 115 6162;7546;8044 True;True;True 6499;8004;8539 56012;67900;67901;67902;67903;67904;67905;67906;67907;67908;67909;67910;67911;67912;73097 82272;98771;98772;98773;98774;98775;98776;98777;98778;98779;98780;98781;98782;98783;98784;98785;98786;98787;98788;98789;98790;106298 82272;98778;106298 169 134

A0A0G2JV84;Q7TP13 A0A0G2JV84;Q7TP13 4;2 4;2 4;2 Gpt2 tr|A0A0G2JV84|A0A0G2JV84_RAT Glutamic--pyruvic transaminase 2 OS=Rattus norvegicus OX=10116 GN=Gpt2 PE=1 SV=1;tr|Q7TP13|Q7TP13_RAT Cc2-5 OS=Rattus norvegicus OX=10116 GN=Gpt2 PE=1 SV=1 2 4 4 4 3 2 4 2 0 2 3 2 4 2 0 2 3 2 4 2 0 2 10.7 10.7 10.7 60.288 544 544;789 5.65 6 11 0 4.1214 By MS/MS By MS/MS By MS/MS By MS/MS By MS/MS 0.2474 1.0221 13.786 16 10 Leave out requantified 0.34256 1.02 2.8128 16 10 Leave out requantified 1.4318 1.0856 13.56 16 10 Leave out requantified 0.33362 0.44646 0.23437 0.32378 NaN 0.23003 1.2239 1.6494 1.0053 1.3686 NaN 0.95282 33.898 45.584 45.506 36.131 NaN 19.529 4 2 6 2 0 2 2 1 4 2 0 1 Median Median Median Median Median Median 0.35135 0.31618 0.34144 0.43062 NaN 0.35178 0.96189 0.94566 1.0397 1.1277 NaN 1.0224 11.684 16.882 20.458 0.035727 NaN 12.697 4 2 6 2 0 2 2 1 4 2 0 1 Median Median Median Median Median Median 1.0021 0.72541 1.4533 1.33 NaN 1.4628 0.73081 0.56383 1.126 0.92947 NaN 1.1647 28.794 73.834 31.607 35.13 NaN 12.729 4 2 6 2 0 2 2 1 4 2 0 1 Median Median Median Median Median Median 6.4 4 10.7 4.8 0 4 55639000 34420000 8165600 13054000 8722500 5534800 1352500 1835200 5153900 3120300 995840 1037700 24832000 15391000 3425500 6015400 3558500 2003800 589550 965150 0 0 0 0 13372000 8369600 1802100 3200300 116 468;1447;3352;11442 True;True;True;True 489;490;1530;3542;12139 4101;4102;4103;4104;4105;4106;4107;4108;12942;12943;12944;12945;30751;30752;30753;30754;104724 5788;5789;5790;5791;5792;5793;5794;5795;5796;18525;18526;18527;18528;18529;43199;43200;43201;152377 5790;18525;43199;152377 170 99

A0A0G2JV87;D3ZL86 A0A0G2JV87;D3ZL86 3;3 3;3 3;3 Heatr1 tr|A0A0G2JV87|A0A0G2JV87_RAT HEAT repeat-containing protein 1 OS=Rattus norvegicus OX=10116 GN=Heatr1 PE=1 SV=1;tr|D3ZL86|D3ZL86_RAT HEAT repeat-containing protein 1 OS=Rattus norvegicus OX=10116 GN=Heatr1 PE=1 SV=2 2 3 3 3 0 0 0 0 2 1 0 0 0 0 2 1 0 0 0 0 2 1 2.3 2.3 2.3 200.69 1790 1790;2143 1 3 0.00053677 2.8742 By MS/MS By MS/MS 0.48567 1.0985 33.272 3 3 Plateau 0.5931 1.0329 82.647 3 3 Median 2.0647 1.7778 52.096 3 3 Median NaN NaN NaN NaN 0.4198 0.28725 NaN NaN NaN NaN 0.87919 0.65592 NaN NaN NaN NaN 40.521 NaN 0 0 0 0 2 1 0 0 0 0 2 1 Median Median Median Median Median Median NaN NaN NaN NaN 0.71403 0.5931 NaN NaN NaN NaN 1.2558 1.0329 NaN NaN NaN NaN 115.79 NaN 0 0 0 0 2 1 0 0 0 0 2 1 Median Median Median Median Median Median NaN NaN NaN NaN 1.7009 2.0647 NaN NaN NaN NaN 1.445 1.7778 NaN NaN NaN NaN 71.705 NaN 0 0 0 0 2 1 0 0 0 0 2 1 Median Median Median Median Median Median 0 0 0 0 1.7 0.7 13985000 7431700 1974100 4579300 0 0 0 0 0 0 0 0 0 0 0 0 0 0 0 0 12153000 6295000 1684800 4173200 1832000 1136700 289280 406090 117 7343;9401;9729 True;True;True 7781;9967;10319 66054;84499;87807 96140;122941;127717 96140;122941;127717 171;172 664;680

A0A0G2JVA4;D3ZQR7;G3V9I0;Q811A3 A0A0G2JVA4;D3ZQR7;G3V9I0;Q811A3 5;5;4;4 5;5;4;4 5;5;4;4 Procollagen-lysine,2-oxoglutarate 5-dioxygenase 2 Plod2 tr|A0A0G2JVA4|A0A0G2JVA4_RAT Procollagen-lysine,2-oxoglutarate 5-dioxygenase 2 OS=Rattus norvegicus OX=10116 GN=Plod2 PE=4 SV=1;tr|D3ZQR7|D3ZQR7_RAT Procollagen-lysine,2-oxoglutarate 5-dioxygenase 2 OS=Rattus norvegicus OX=10116 GN=Plod2 PE=4 SV=1;tr|G3V9I 4 5 5 5 2 3 4 3 3 3 2 3 4 3 3 3 2 3 4 3 3 3 6.9 6.9 6.9 86.483 754 754;758;737;737 3.71 3 4 19 2 0 22.221 By MS/MS By MS/MS By MS/MS By MS/MS By MS/MS By MS/MS 0.54019 1.9676 37.583 24 8 Leave out requantified 0.62236 1.5337 31.108 23 7 Leave out requantified 1.1119 0.83327 14.719 23 7 Leave out requantified 0.48518 0.54595 0.57178 0.52631 0.54618 0.55847 1.7418 2.1243 1.7701 2.0814 2.8625 1.8767 2.3094 12.179 45.799 20.208 82.698 19.611 2 3 7 3 4 5 0 0 3 1 2 2 Median Leave out requantified Leave out requantified Median Linear Leave out requantified 0.46436 0.52162 0.54555 0.67784 0.6703 0.7342 1.096 1.6372 1.2873 1.7705 1.6268 1.8791 5.7359 13.961 28.059 16.229 81.56 14.29 2 3 7 3 4 4 0 0 3 1 2 1 Median Leave out requantified Leave out requantified Plateau Median Leave out requantified 0.90664 0.97485 0.93719 1.2913 1.1949 1.3132 0.62718 0.76661 0.73439 0.9183 0.90777 1.0145 13.479 10.056 13.824 44.915 12.841 11.172 2 3 7 3 4 4 0 0 3 1 2 1 Median Leave out requantified Leave out requantified Median Median Leave out requantified 2.9 3.8 4.9 4.9 4.9 4.9 270260000 127830000 65286000 77145000 8304300 4301600 2207300 1795400 42438000 20446000 11256000 10736000 40958000 19714000 11608000 9636800 55604000 26459000 12247000 16898000 58530000 26914000 12905000 18711000 64428000 29998000 15062000 19369000 118 2363;4776;5483;6436;12136 True;True;True;True;True 2518;5028;5786;6783;12873 22286;22287;22288;43379;43380;50198;50199;50200;50201;50202;50203;50204;50205;50206;50207;50208;50209;50210;50211;50212;58111;58112;58113;58114;58115;58116;58117;112402 31169;31170;31171;31172;61998;61999;62000;72290;72291;72292;72293;72294;72295;72296;72297;72298;72299;72300;72301;72302;72303;72304;72305;72306;72307;72308;72309;72310;72311;72312;72313;72314;85215;85216;85217;85218;85219;85220;85221;85222;85223;163447 31171;62000;72307;85222;163447 173 512

B5DF60;A0A0G2JVA7;Q6VV72 B5DF60;A0A0G2JVA7;Q6VV72 1;1;1 1;1;1 1;1;1 Eukaryotic translation initiation factor 1A Eif1ax;Eif1a tr|B5DF60|B5DF60_RAT Eukaryotic translation initiation factor 1A, X-linked OS=Rattus norvegicus OX=10116 GN=Eif1ax PE=1 SV=1;tr|A0A0G2JVA7|A0A0G2JVA7_RAT Eukaryotic translation initiation factor 1A OS=Rattus norvegicus OX=10116 GN=Eif1a PE=1 SV=1;sp|Q6VV72 3 1 1 1 0 0 1 1 1 1 0 0 1 1 1 1 0 0 1 1 1 1 6.9 6.9 6.9 16.46 144 144;144;144 10.8 2 6 0.0092552 1.4987 By MS/MS By MS/MS By MS/MS By MS/MS 0.33569 1.6312 26.005 6 1 Median 0.55213 1.5662 23.586 6 1 Median 1.7694 1.2279 19.418 6 1 Median NaN NaN 0.50333 0.34008 0.33136 0.29765 NaN NaN 2.6813 1.5874 1.6761 1.4075 NaN NaN NaN 15.614 NaN NaN 0 0 1 3 1 1 0 0 0 1 0 0 Median Median Median Median Median Median NaN NaN 0.66254 0.51923 0.43409 0.58713 NaN NaN 2.2716 1.5731 1.3438 1.5592 NaN NaN NaN 25.001 NaN NaN 0 0 1 3 1 1 0 0 0 1 0 0 Median Median Median Median Median Median NaN NaN 1.3026 1.804 1.3507 1.8258 NaN NaN 0.93011 1.2445 0.9155 1.3019 NaN NaN NaN 11.362 NaN NaN 0 0 1 3 1 1 0 0 0 1 0 0 Median Median Median Median Median Median 0 0 6.9 6.9 6.9 6.9 15959000 7938500 3314700 4705300 0 0 0 0 0 0 0 0 2261100 892460 830440 538180 7214700 3406000 1375200 2433500 2927400 1747700 505330 674440 3555300 1892400 603730 1059200 119 12189 True 12929 112828;112829;112830;112831;112832;112833;112834;112835 164105;164106;164107;164108;164109;164110;164111;164112;164113;164114 164112

A0A0G2K4U8;A0A0G2JVB6;G3V7P1 A0A0G2K4U8;A0A0G2JVB6;G3V7P1 1;1;1 1;1;1 1;1;1 Syntaxin-12 Stx12 tr|A0A0G2K4U8|A0A0G2K4U8_RAT Syntaxin-12 OS=Rattus norvegicus OX=10116 GN=Stx12 PE=1 SV=1;tr|A0A0G2JVB6|A0A0G2JVB6_RAT Syntaxin-12 OS=Rattus norvegicus OX=10116 GN=Stx12 PE=1 SV=1;sp|G3V7P1|STX12_RAT Syntaxin-12 OS=Rattus norvegicus OX=10116 GN=Stx12 PE=1 3 1 1 1 0 1 0 0 1 1 0 1 0 0 1 1 0 1 0 0 1 1 6.6 6.6 6.6 31.106 272 272;285;274 8 3 0 4.1502 By MS/MS By MS/MS By matching 0.53826 2.8244 40.119 3 1 Median 0.7531 2.6017 11.299 3 1 Median 1.367 0.96266 33.347 3 1 Median NaN 0.76372 NaN NaN 0.53826 0.34221 NaN 3.5576 NaN NaN 2.8244 1.6293 NaN NaN NaN NaN NaN NaN 0 1 0 0 1 1 0 1 0 0 0 0 Median Median Median Median Median Median NaN 0.7531 NaN NaN 0.67661 0.80812 NaN 2.6017 NaN NaN 2.1604 2.6499 NaN NaN NaN NaN NaN NaN 0 1 0 0 1 1 0 1 0 0 0 0 Median Median Median Median Median Median NaN 0.98609 NaN NaN 1.367 1.962 NaN 0.77098 NaN NaN 0.96266 1.4852 NaN NaN NaN NaN NaN NaN 0 1 0 0 1 1 0 1 0 0 0 0 Median Median Median Median Median Median 0 6.6 0 0 6.6 6.6 10247000 4783000 2259800 3204200 0 0 0 0 1495500 571300 342480 581730 0 0 0 0 0 0 0 0 5477000 2631100 1324200 1521700 3274600 1580700 593110 1100800 120 3002 True 3176 28269;28270;28271 39865;39866;39867 39866

D4A1Y0;A0A0G2JVC2 D4A1Y0;A0A0G2JVC2 3;3 3;3 3;3 Scyl2 tr|D4A1Y0|D4A1Y0_RAT SCY1-like pseudokinase 2 OS=Rattus norvegicus OX=10116 GN=Scyl2 PE=1 SV=1;tr|A0A0G2JVC2|A0A0G2JVC2_RAT SCY1-like pseudokinase 2 OS=Rattus norvegicus OX=10116 GN=Scyl2 PE=1 SV=1 2 3 3 3 0 0 0 0 0 3 0 0 0 0 0 3 0 0 0 0 0 3 5.8 5.8 5.8 103.36 930 930;934 4 3 0 3.5902 By MS/MS 0.48491 1.1144 35.213 3 1 Median 1.1486 2.2622 21.437 3 1 Median 2.3414 1.846 36.737 3 1 Median NaN NaN NaN NaN NaN 0.48491 NaN NaN NaN NaN NaN 1.1144 NaN NaN NaN NaN NaN 35.213 0 0 0 0 0 3 0 0 0 0 0 1 Median Median Median Median Median Median NaN NaN NaN NaN NaN 1.1486 NaN NaN NaN NaN NaN 2.2622 NaN NaN NaN NaN NaN 21.437 0 0 0 0 0 3 0 0 0 0 0 1 Median Median Median Median Median Median NaN NaN NaN NaN NaN 2.3414 NaN NaN NaN NaN NaN 1.846 NaN NaN NaN NaN NaN 36.737 0 0 0 0 0 3 0 0 0 0 0 1 Median Median Median Median Median Median 0 0 0 0 0 5.8 18468000 7594100 3150800 7723600 0 0 0 0 0 0 0 0 0 0 0 0 0 0 0 0 0 0 0 0 18468000 7594100 3150800 7723600 121 5358;6389;8602 True;True;True 5641;6735;9124 48520;57752;77362 69872;84694;112512 69872;84694;112512 174;175 756;757

A0A0G2JVG3;P11980;M0RD14 A0A0G2JVG3;P11980;M0RD14 14;13;12 14;13;12 14;13;12 Pyruvate kinase PKM;Pyruvate kinase Pkm tr|A0A0G2JVG3|A0A0G2JVG3_RAT Pyruvate kinase OS=Rattus norvegicus OX=10116 GN=Pkm PE=1 SV=1;sp|P11980|KPYM_RAT Pyruvate kinase PKM OS=Rattus norvegicus OX=10116 GN=Pkm PE=1 SV=3;tr|M0RD14|M0RD14_RAT Pyruvate kinase OS=Rattus norvegicus OX=10116 PE=3 SV=2 3 14 14 14 10 11 12 10 11 11 10 11 12 10 11 11 10 11 12 10 11 11 33.7 33.7 33.7 53.193 489 489;531;486 3.66 52 18 5 4 49 33 4 2 1 0 109.46 By MS/MS By MS/MS By MS/MS By MS/MS By MS/MS By MS/MS 0.21133 0.8074 24.365 130 72 Leave out requantified 0.32434 0.91803 13.754 130 72 Leave out requantified 1.5177 1.1687 17.685 130 72 Leave out requantified 0.17 0.20968 0.20427 0.18213 0.1921 0.20053 0.85327 0.84901 0.90949 0.82154 0.68373 0.71686 52.229 8.0794 26.778 35.688 48.285 32.389 13 16 24 22 24 31 10 8 13 13 15 13 Leave out requantified Leave out requantified Leave out requantified Leave out requantified Leave out requantified Leave out requantified 0.37078 0.29699 0.30046 0.32784 0.34263 0.32647 1.2252 1.0644 0.89506 0.92829 0.91599 0.83513 22.05 21.386 23.191 16.975 8.9751 19.175 13 16 24 22 24 31 10 8 13 13 15 13 Leave out requantified Leave out requantified Leave out requantified Leave out requantified Leave out requantified Leave out requantified 2.074 1.4903 1.5636 1.6642 1.6756 1.5269 1.5274 1.1311 1.2263 1.1932 1.2117 1.1396 12.516 21.609 13.46 25.575 50.478 25.019 13 16 24 22 24 31 10 8 13 13 15 13 Leave out requantified Leave out requantified Leave out requantified Leave out requantified Leave out requantified Leave out requantified 25.6 26.4 29 24.1 28.6 27.8 1717200000 1118800000 239370000 359000000 136240000 92885000 13778000 29579000 213510000 142350000 29411000 41746000 289680000 185080000 47993000 56608000 340430000 210830000 42753000 86842000 325100000 212920000 44582000 67600000 412240000 274750000 60855000 76627000 122 1582;1585;3685;3974;4405;4573;4694;4695;5525;5526;5671;5672;7926;9203 True;True;True;True;True;True;True;True;True;True;True;True;True;True 1671;1674;3888;3889;4199;4645;4821;4946;4947;5830;5831;5984;5985;8414;9761 14075;14076;14077;14078;14079;14102;14103;14104;33338;33339;33340;33341;33342;33343;33344;33345;33346;33347;33348;33349;33350;35940;35941;35942;35943;35944;35945;35946;35947;35948;35949;35950;35951;35952;35953;39671;39672;39673;39674;39675;39676;39677;39678;39679;41421;41422;41423;41424;41425;41426;41427;41428;41429;41430;41431;41432;41433;42520;42521;42522;42523;42524;42525;42526;42527;42528;42529;42530;42531;42532;42533;42534;42535;42536;42537;42538;42539;42540;42541;42542;42543;42544;42545;42546;42547;42548;42549;42550;42551;42552;42553;42554;42555;42556;42557;42558;42559;42560;42561;42562;42563;42564;42565;42566;50619;50620;50621;50622;50623;50624;50625;50626;50627;50628;50629;50630;50631;50632;50633;50634;50635;50636;50637;51661;51662;51663;51664;51665;51666;51667;51668;51669;51670;51671;51672;51673;51674;51675;51676;51677;51678;51679;51680;51681;51682;51683;51684;51685;51686;51687;51688;51689;51690;51691;51692;51693;72005;72006;72007;72008;72009;72010;72011;72012;72013;72014;82423;82424 20073;20074;20075;20076;20113;20114;20115;46674;46675;46676;46677;46678;46679;46680;46681;46682;46683;46684;46685;46686;46687;46688;46689;46690;46691;50673;50674;50675;50676;50677;50678;50679;50680;50681;50682;50683;50684;50685;50686;50687;50688;50689;50690;50691;50692;50693;56202;56203;56204;56205;56206;56207;56208;56209;56210;59317;59318;59319;59320;59321;59322;59323;59324;59325;59326;59327;59328;59329;59330;59331;60810;60811;60812;60813;60814;60815;60816;60817;60818;60819;60820;60821;60822;60823;60824;60825;60826;60827;60828;60829;60830;60831;60832;60833;60834;60835;60836;60837;60838;60839;60840;60841;60842;60843;60844;60845;60846;60847;60848;60849;60850;60851;60852;60853;60854;60855;60856;60857;60858;60859;60860;60861;60862;60863;60864;60865;60866;72880;72881;72882;72883;72884;72885;72886;72887;72888;72889;72890;72891;72892;72893;72894;72895;72896;72897;72898;72899;72900;72901;74671;74672;74673;74674;74675;74676;74677;74678;74679;74680;74681;74682;74683;74684;74685;74686;74687;74688;74689;74690;74691;74692;74693;74694;74695;74696;74697;74698;74699;74700;74701;74702;74703;74704;74705;74706;74707;74708;74709;74710;74711;74712;74713;74714;74715;74716;74717;104777;104778;104779;104780;104781;104782;104783;104784;104785;104786;104787;104788;104789;104790;104791;119798;119799 20073;20114;46674;50683;56205;59327;60832;60860;72882;72900;74684;74717;104779;119799 176;177 69;239

A0A0G2JVH4;A0A140TAG5;Q3KR86 A0A0G2JVH4;A0A140TAG5;Q3KR86 20;17;17 20;17;17 20;17;17 MICOS complex subunit Mic60 Immt tr|A0A0G2JVH4|A0A0G2JVH4_RAT MICOS complex subunit MIC60 OS=Rattus norvegicus OX=10116 GN=Immt PE=1 SV=1;tr|A0A140TAG5|A0A140TAG5_RAT MICOS complex subunit MIC60 OS=Rattus norvegicus OX=10116 GN=Immt PE=1 SV=1;sp|Q3KR86|MIC60_RAT MICOS complex subunit Mic6 3 20 20 20 14 17 15 17 14 17 14 17 15 17 14 17 14 17 15 17 14 17 31.3 31.3 31.3 86.229 776 776;608;609 3.66 30 11 13 128 44 2 0 264.41 By MS/MS By MS/MS By MS/MS By MS/MS By MS/MS By MS/MS 0.2325 0.84716 16.233 205 111 Leave out requantified 0.27633 0.71953 11.048 203 108 Leave out requantified 1.2305 0.9035 7.7339 203 108 Leave out requantified 0.21274 0.22873 0.1994 0.23549 0.24285 0.21079 0.79391 0.92194 0.7643 0.83417 0.85263 0.82507 27.385 23.267 21.581 16.273 22.038 17.27 25 33 41 30 33 43 17 22 25 14 11 22 Leave out requantified Leave out requantified Leave out requantified Leave out requantified Leave out requantified Leave out requantified 0.26291 0.24582 0.26093 0.28761 0.292 0.28008 0.76386 0.73049 0.7512 0.77366 0.72891 0.71752 22.098 13.473 13.412 15.558 8.2727 6.6748 25 31 40 30 33 44 17 20 24 14 11 22 Leave out requantified Leave out requantified Leave out requantified Leave out requantified Leave out requantified Leave out requantified 1.3287 1.052 1.271 1.2152 1.1625 1.2274 0.92444 0.81483 0.965 0.84739 0.83525 0.92922 15.934 30.636 19.738 6.5966 12.273 10.655 25 31 40 30 33 44 17 20 24 14 11 22 Leave out requantified Leave out requantified Leave out requantified Leave out requantified Leave out requantified Leave out requantified 24 29.6 26.3 28 24.4 26.7 4595700000 3209900000 627700000 758080000 298220000 199590000 43348000 55279000 736140000 526780000 101190000 108160000 837430000 587760000 111340000 138330000 759000000 518150000 107680000 133160000 953820000 680100000 126170000 147550000 1011100000 697540000 137970000 175600000 123 555;1404;2957;5833;5876;6031;6134;6222;6425;8063;8125;8218;8495;8550;8974;9371;10128;10129;10369;10387 True;True;True;True;True;True;True;True;True;True;True;True;True;True;True;True;True;True;True;True 585;1486;3131;6156;6200;6365;6470;6471;6562;6772;8559;8627;8723;9013;9070;9508;9937;10735;10736;10737;10989;11007 4949;4950;4951;4952;4953;4954;4955;4956;12613;12614;12615;12616;12617;12618;12619;12620;12621;12622;12623;12624;12625;12626;12627;27998;27999;28000;28001;28002;28003;28004;28005;28006;28007;28008;28009;28010;28011;28012;28013;28014;28015;28016;53019;53020;53021;53022;53023;53024;53025;53026;53491;53492;53493;53494;53495;53496;53497;53498;53499;53500;53501;53502;53503;53504;53505;53506;53507;54788;55608;55609;55610;55611;55612;55613;55614;55615;55616;55617;55618;55619;55620;55621;55622;55623;55624;55625;55626;55627;55628;55629;55630;55631;55632;55633;55634;55635;55636;56370;56371;56372;56373;56374;56375;56376;56377;56378;56379;56380;58061;58062;73168;73169;73170;73171;73172;73173;73661;73662;73663;73664;73665;73666;74555;74556;74557;74558;74559;76564;76565;76566;76567;76568;76569;76570;76571;76572;76573;76574;76575;76576;76577;76578;76579;76580;76581;76582;76583;76584;76585;76586;76587;76956;79992;79993;79994;79995;79996;79997;79998;79999;80000;80001;80002;80003;80004;80005;80006;80007;80008;84122;84123;84124;84125;84126;84127;84128;84129;84130;84131;84132;84133;84134;84135;84136;84137;84138;84139;84140;84141;84142;84143;84144;84145;84146;84147;91110;91111;91112;91113;91114;91115;91116;91117;91118;91119;91120;91121;91122;91123;91124;91125;91126;91127;91128;91129;91130;91131;91132;91133;93549;93550;93551;93552;93553;93554;93555;93684;93685 7007;7008;7009;7010;7011;7012;7013;7014;7015;7016;7017;18045;18046;18047;18048;18049;18050;18051;18052;18053;18054;18055;18056;18057;39523;39524;39525;39526;39527;39528;39529;39530;39531;39532;39533;39534;39535;39536;39537;39538;39539;39540;39541;39542;39543;39544;39545;39546;39547;77468;77469;77470;77471;77472;77473;77474;77475;77476;77477;77478;77479;77480;78344;78345;78346;78347;78348;78349;78350;78351;78352;78353;78354;78355;78356;78357;78358;78359;78360;78361;78362;78363;78364;78365;78366;78367;78368;78369;78370;78371;80615;80616;81623;81624;81625;81626;81627;81628;81629;81630;81631;81632;81633;81634;81635;81636;81637;81638;81639;81640;81641;81642;81643;81644;81645;81646;81647;81648;81649;81650;81651;81652;81653;81654;81655;81656;81657;81658;81659;81660;82769;82770;82771;82772;82773;82774;82775;82776;82777;82778;82779;82780;82781;82782;82783;82784;85140;85141;106385;106386;106387;106388;106389;106390;106391;107199;107200;107201;107202;107203;107204;107205;107206;107207;107208;107209;108437;108438;108439;108440;108441;111165;111166;111167;111168;111169;111170;111171;111172;111173;111174;111175;111176;111177;111178;111179;111180;111181;111182;111183;111184;111185;111186;111187;111188;111189;111190;111191;111192;111193;111194;111195;111196;111197;111198;111199;111200;111201;111202;111203;111204;111205;111206;111207;111208;111772;116461;116462;116463;116464;116465;116466;116467;116468;116469;116470;116471;116472;116473;116474;116475;116476;116477;116478;116479;116480;116481;116482;116483;122389;122390;122391;122392;122393;122394;122395;122396;122397;122398;122399;122400;122401;122402;122403;122404;122405;122406;122407;122408;122409;122410;122411;122412;122413;122414;122415;122416;122417;132268;132269;132270;132271;132272;132273;132274;132275;132276;132277;132278;132279;132280;132281;132282;132283;132284;132285;132286;132287;132288;132289;132290;132291;132292;132293;132294;132295;132296;135794;135795;135796;135797;135798;135799;135943 7014;18051;39524;77473;78367;80615;81649;82777;85141;106390;107201;108438;111207;111772;116476;122407;132269;132294;135797;135943 178;179;180;181 88;201;244;318

G3V6Z3;A0A0G2JVI5;Q8VH46 G3V6Z3;A0A0G2JVI5;Q8VH46 6;6;6 6;6;6 6;6;6 Actin filament-associated protein 1 Afap1 tr|G3V6Z3|G3V6Z3_RAT Actin filament-associated protein 1 OS=Rattus norvegicus OX=10116 GN=Afap1 PE=1 SV=1;tr|A0A0G2JVI5|A0A0G2JVI5_RAT Actin filament-associated protein 1 OS=Rattus norvegicus OX=10116 GN=Afap1 PE=1 SV=1;sp|Q8VH46|AFAP1_RAT Actin filament-a 3 6 6 6 2 0 2 4 2 4 2 0 2 4 2 4 2 0 2 4 2 4 8.1 8.1 8.1 80.746 731 731;744;731 4 16 0 8.3231 By MS/MS By MS/MS By MS/MS By MS/MS By MS/MS 1.2307 3.9898 25.601 14 6 Leave out requantified 2.6364 5.0385 32.833 15 6 Leave out requantified 1.9584 1.3646 6.4153 15 6 Leave out requantified 1.5291 NaN 0.98585 1.2106 1.2398 1.3979 5.3161 NaN 2.4393 3.1933 4.2523 4.0206 4.0625 NaN 24.051 39.015 31.293 10.639 2 0 2 4 3 3 1 0 2 1 1 1 Median Median Median Leave out requantified Plateau Median 1.9894 NaN 1.5641 2.6736 2.7618 2.984 4.4029 NaN 3.0116 4.3625 6.8142 6.3573 1.387 NaN 2.3489 23.375 67.674 46.798 2 0 2 4 3 4 1 0 2 1 1 1 Median Median Median Leave out requantified Plateau Leave out requantified 1.2406 NaN 1.5865 1.7582 1.9584 2.2178 0.88697 NaN 1.3088 1.2337 1.4475 1.5726 3.1977 NaN 26.348 12.346 39.349 20.387 2 0 2 4 3 4 1 0 2 1 1 1 Median Median Median Leave out requantified Plateau Leave out requantified 3.6 0 3.3 5.1 3.6 7 81146000 17382000 18416000 45348000 3697600 766340 868820 2062500 0 0 0 0 6737300 1611900 1852800 3272600 23792000 5807400 5312900 12672000 18360000 4597100 4082200 9680700 28559000 4599500 6299000 17661000 124 1468;3193;5450;8707;10692;10693 True;True;True;True;True;True 1552;3376;5750;9232;11336;11337 13124;29641;49929;49930;49931;49932;49933;49934;49935;77932;77933;96627;96628;96629;96630;96631 18766;41548;71914;71915;71916;71917;71918;71919;71920;113334;113335;113336;113337;140460;140461;140462;140463;140464;140465;140466;140467;140468 18766;41548;71917;113335;140460;140464

A0A0G2JVL6;F7EXQ7 A0A0G2JVL6;F7EXQ7 3;2 3;2 3;2 tr|A0A0G2JVL6|A0A0G2JVL6_RAT NADH dehydrogenase [ubiquinone] 1 alpha subcomplex subunit 8 OS=Rattus norvegicus OX=10116 GN=Ndufa8 PE=1 SV=1;tr|F7EXQ7|F7EXQ7_RAT NADH dehydrogenase [ubiquinone] 1 alpha subcomplex subunit 8 OS=Rattus norvegicus OX=10116 GN=N 2 3 3 3 0 0 0 2 2 1 0 0 0 2 2 1 0 0 0 2 2 1 22.1 22.1 22.1 19.965 172 172;190 9.88 1 7 0 4.7187 By MS/MS By MS/MS By MS/MS 0.21207 0.95278 23.992 5 1 Leave out requantified 0.28974 0.89818 9.1561 5 1 Leave out requantified 1.6102 1.0967 4.4453 5 1 Leave out requantified NaN NaN NaN 0.24173 0.20525 0.16436 NaN NaN NaN 1.1313 0.93754 0.69232 NaN NaN NaN 25.43 0.54211 NaN 0 0 0 2 2 1 0 0 0 1 0 0 Median Median Median Median Median Median NaN NaN NaN 0.30436 0.28233 0.29379 NaN NaN NaN 1.0799 0.8645 0.82658 NaN NaN NaN 3.4937 1.157 NaN 0 0 0 2 2 1 0 0 0 1 0 0 Median Median Median Median Median Median NaN NaN NaN 1.5174 1.6867 1.5792 NaN NaN NaN 0.99848 1.1429 1.1185 NaN NaN NaN 1.1565 0.28731 NaN 0 0 0 2 2 1 0 0 0 1 0 0 Median Median Median Median Median Median 0 0 0 18 14.5 4.1 35271000 23373000 4482600 7414800 0 0 0 0 0 0 0 0 0 0 0 0 18263000 12033000 2570200 3659400 12683000 8330600 1540000 2812800 4324700 3009600 372420 942620 125 6215;7966;11743 True;True;True 6555;8456;8457;12464 56338;72567;72568;72569;72570;72571;108453;108454 82712;105559;105560;105561;105562;105563;105564;105565;105566;157953 82712;105560;157953

A0A0G2JVM2;A0A0G2K0A8 A0A0G2JVM2 21;10 21;10 21;10 tr|A0A0G2JVM2|A0A0G2JVM2_RAT Uncharacterized protein OS=Rattus norvegicus OX=10116 PE=1 SV=1 2 21 21 21 16 16 18 15 17 17 16 16 18 15 17 17 16 16 18 15 17 17 22.3 22.3 22.3 153.47 1377 1377;780 2.46 1 114 61 14 0 163.68 By MS/MS By MS/MS By MS/MS By MS/MS By MS/MS By MS/MS 0.5537 1.322 22.725 179 50 Leave out requantified 0.70685 1.4046 13.399 179 49 Leave out requantified 1.2778 0.92172 14.258 176 49 Leave out requantified 0.55401 0.53275 0.54698 0.53777 0.58087 0.4967 1.3218 1.5299 1.3521 1.3105 1.4132 1.1968 25.256 20.003 21.699 19.443 29.333 38.584 27 23 33 30 32 34 8 2 11 14 8 7 Leave out requantified Leave out requantified Leave out requantified Leave out requantified Leave out requantified Leave out requantified 0.66334 0.65575 0.64607 0.78471 0.74022 0.78052 1.2694 1.3322 1.3299 1.3994 1.7002 1.6874 18.234 17.989 29.502 34.579 31.055 26.551 27 23 32 30 32 35 8 2 10 14 8 7 Leave out requantified Leave out requantified Leave out requantified Leave out requantified Leave out requantified Leave out requantified 1.1191 1.1314 1.1372 1.408 1.2378 1.5632 0.75799 0.90663 0.86612 1.0473 0.96054 1.1828 19.136 15.703 14.311 15.121 14.364 24.765 27 22 32 30 32 33 8 2 10 14 8 7 Leave out requantified Leave out requantified Leave out requantified Leave out requantified Leave out requantified Leave out requantified 16.8 16.7 19 14.2 18.9 18.7 2948600000 1342100000 699460000 907050000 361650000 175110000 86446000 100090000 419240000 190200000 105750000 123290000 448390000 207220000 111610000 129570000 342400000 155240000 72739000 114420000 627450000 281070000 146290000 200090000 749450000 333230000 176630000 239590000 126 1821;2203;2382;2461;2462;2662;2844;4878;5742;5760;6364;7131;7752;8284;8297;9302;9457;11243;11348;11887;12066 True;True;True;True;True;True;True;True;True;True;True;True;True;True;True;True;True;True;True;True;True 1929;2345;2537;2621;2622;2826;3015;5135;6059;6079;6710;7530;8232;8793;8807;9864;10026;11924;12038;12616;12801 16634;16635;16636;20664;20665;20666;20667;20668;20669;20670;20671;20672;20673;20674;20675;22355;22356;22357;22358;22359;22360;22361;22362;22363;22364;22365;22366;22367;23252;23253;23254;23255;23256;23257;23258;23259;23260;23261;23262;23263;23264;23265;25122;25123;25124;25125;25126;25127;25128;25129;25130;25131;25132;25133;25134;25135;25136;25137;25138;26816;26817;26818;26819;26820;26821;26822;26823;26824;26825;26826;26827;26828;26829;26830;26831;44453;44454;44455;44456;44457;44458;44459;44460;44461;44462;52186;52187;52188;52399;52400;52401;52402;52403;52404;52405;57519;57520;57521;57522;57523;57524;57525;64068;64069;64070;64071;64072;64073;64074;70141;70142;70143;70144;70145;70146;70147;70148;70149;70150;70151;70152;70153;70154;70155;70156;70157;70158;74954;74955;74956;74957;75063;75064;75065;75066;75067;75068;75069;75070;83461;85205;85206;85207;85208;85209;85210;85211;102650;102651;102652;102653;102654;102655;102656;102657;102658;102659;102660;102661;103993;103994;103995;103996;103997;103998;103999;104000;104001;104002;104003;104004;104005;104006;110082;110083;110084;110085;110086;110087;110088;110089;110090;110091;110092;110093;110094;110095;110096;110097;111618 23538;23539;23540;29059;29060;29061;29062;29063;29064;29065;29066;29067;29068;29069;29070;29071;29072;29073;29074;29075;29076;29077;29078;29079;29080;29081;29082;29083;29084;31258;31259;31260;31261;31262;31263;31264;31265;31266;31267;31268;31269;31270;31271;31272;31273;31274;31275;31276;31277;31278;31279;31280;31281;31282;31283;31284;31285;31286;32557;32558;32559;32560;32561;32562;32563;32564;32565;32566;32567;32568;32569;32570;32571;32572;32573;32574;32575;32576;32577;32578;32579;32580;35157;35158;35159;35160;35161;35162;35163;35164;35165;35166;35167;35168;35169;35170;35171;35172;37672;37673;37674;37675;37676;37677;37678;37679;37680;37681;37682;37683;37684;37685;37686;37687;37688;37689;37690;37691;37692;37693;37694;37695;37696;37697;37698;37699;37700;63730;63731;63732;63733;63734;63735;63736;63737;63738;63739;63740;63741;63742;63743;63744;63745;63746;75665;75666;75667;76092;76093;76094;76095;76096;76097;76098;76099;76100;76101;76102;76103;76104;84323;84324;84325;84326;93388;93389;93390;93391;93392;93393;93394;102144;102145;102146;102147;102148;102149;102150;102151;102152;102153;102154;102155;102156;102157;102158;102159;102160;102161;102162;102163;102164;102165;102166;102167;102168;102169;109006;109007;109008;109160;109161;109162;109163;109164;109165;109166;109167;121500;124059;124060;124061;124062;124063;149098;149099;149100;149101;149102;149103;149104;149105;149106;149107;149108;149109;149110;149111;149112;149113;149114;149115;149116;149117;149118;149119;151316;151317;151318;151319;151320;151321;151322;151323;151324;151325;151326;151327;151328;151329;151330;151331;160302;160303;160304;160305;160306;160307;160308;160309;160310;160311;160312;160313;160314;160315;160316;160317;160318;160319;160320;160321;160322;160323;162346 23540;29073;31273;32558;32568;35163;37689;63744;75665;76092;84323;93393;102145;109006;109161;121500;124063;149098;151318;160318;162346 182;183;184;185;186;187 401;439;796;929;930;1104

A0A0G2JVN4;M0RDN9;Q6J1Y9 A0A0G2JVN4;M0RDN9;Q6J1Y9 2;2;2 2;2;2 2;2;2 Ubiquitin carboxyl-terminal hydrolase;Ubiquitin carboxyl-terminal hydrolase 19 Usp19 tr|A0A0G2JVN4|A0A0G2JVN4_RAT RCG25629, isoform CRA_a OS=Rattus norvegicus OX=10116 GN=Usp19 PE=1 SV=1;tr|M0RDN9|M0RDN9_RAT Ubiquitin carboxyl-terminal hydrolase 19 OS=Rattus norvegicus OX=10116 GN=Usp19 PE=1 SV=1;sp|Q6J1Y9|UBP19_RAT Ubiquitin carboxyl-term 3 2 2 2 0 0 0 0 1 1 0 0 0 0 1 1 0 0 0 0 1 1 4.2 4.2 4.2 146.2 1322 1322;1357;1357 3 2 0 11.176 By MS/MS By MS/MS 1.3075 2.5314 11.167 2 1 Median 1.6556 2.5931 12.328 2 1 Median 1.1808 0.93525 14.811 2 1 Median NaN NaN NaN NaN 1.1679 1.4639 NaN NaN NaN NaN 2.3392 2.7394 NaN NaN NaN NaN NaN NaN 0 0 0 0 1 1 0 0 0 0 1 0 Median Median Median Median Median Median NaN NaN NaN NaN 1.5172 1.8066 NaN NaN NaN NaN 2.3767 2.8294 NaN NaN NaN NaN NaN NaN 0 0 0 0 1 1 0 0 0 0 1 0 Median Median Median Median Median Median NaN NaN NaN NaN 1.2991 1.0733 NaN NaN NaN NaN 1.0385 0.84225 NaN NaN NaN NaN NaN NaN 0 0 0 0 1 1 0 0 0 0 1 0 Median Median Median Median Median Median 0 0 0 0 2.5 1.7 11704000 3066600 3631000 5006100 0 0 0 0 0 0 0 0 0 0 0 0 0 0 0 0 5855400 1105800 1485700 3264000 5848200 1960800 2145300 1742100 127 8952;11047 True;True 9483;11717 79723;100559 115962;146130 115962;146130

D4A0B4;A0A0G2JVW3;E9PTK9;A0A0G2K916 D4A0B4;A0A0G2JVW3;E9PTK9;A0A0G2K916 3;3;2;2 3;3;2;2 3;3;2;2 Ankrd17;Ankhd1 tr|D4A0B4|D4A0B4_RAT Ankyrin repeat domain 17 OS=Rattus norvegicus OX=10116 GN=Ankrd17 PE=1 SV=1;tr|A0A0G2JVW3|A0A0G2JVW3_RAT Ankyrin repeat domain 17 OS=Rattus norvegicus OX=10116 GN=Ankrd17 PE=1 SV=1;tr|E9PTK9|E9PTK9_RAT Eukaryotic translation initiation 4 3 3 3 2 1 3 0 1 0 2 1 3 0 1 0 2 1 3 0 1 0 2.8 2.8 2.8 250.02 2358 2358;2524;2523;2615 2.29 10 4 0 5.8827 By MS/MS By MS/MS By MS/MS By MS/MS 1.4379 3.015 22.622 12 11 Median 1.4393 2.3924 26.562 12 11 Median 0.99287 0.85687 28.489 12 11 Median 1.113 1.2735 1.585 NaN 1.8014 NaN 2.0259 3.0294 3.1198 NaN 3.6996 NaN 2.3125 9.2238 20.32 NaN NaN NaN 2 2 7 0 1 0 2 2 6 0 1 0 Median Median Median Median Median Median 1.2373 1.7771 2.0491 NaN 0.9971 NaN 1.8505 3.2415 3.3904 NaN 1.8902 NaN 19.851 12.97 24.191 NaN NaN NaN 2 2 7 0 1 0 2 2 6 0 1 0 Median Median Linear Median Median Median 1.1117 1.3954 0.92691 NaN 0.55352 NaN 0.91992 1.1621 0.79942 NaN 0.4469 NaN 22.109 13.814 18.576 NaN NaN NaN 2 2 7 0 1 0 2 2 6 0 1 0 Median Median Median Median Median Median 2 1 2.8 0 1 0 57317000 11512000 22567000 23238000 5783600 1139400 1717600 2926600 17690000 3404500 7133400 7152500 26281000 5831700 9407900 11041000 0 0 0 0 7561600 1136000 4307900 2117800 0 0 0 0 128 3973;7806;10290 True;True;True 4198;8287;10907 35933;35934;35935;35936;35937;35938;35939;70692;92649;92650;92651;92652;92653;92654 50666;50667;50668;50669;50670;50671;50672;102981;134507;134508;134509;134510;134511;134512;134513;134514 50666;102981;134512 188 1201

A0A0G2JVW5;E9PSZ5 A0A0G2JVW5 4;1 4;1 4;1 tr|A0A0G2JVW5|A0A0G2JVW5_RAT E3 ubiquitin-protein ligase HUWE1 OS=Rattus norvegicus OX=10116 GN=Huwe1 PE=1 SV=1 2 4 4 4 3 3 3 2 4 4 3 3 3 2 4 4 3 3 3 2 4 4 1.3 1.3 1.3 447.65 4062 4062;914 1.41 20 3 4 0 35.928 By MS/MS By MS/MS By MS/MS By MS/MS By MS/MS By MS/MS 0.52592 1.3102 3.532 22 8 Leave out requantified 0.53692 1.1061 24.819 21 7 Leave out requantified 1.051 0.84328 4.7633 21 7 Leave out requantified 0.41911 0.52383 0.60163 0.92257 0.5799 0.58882 1.1443 1.2561 1.9472 1.6352 1.2666 1.3199 26.642 3.5876 30.11 79.229 26.739 12.028 3 3 3 2 6 5 0 0 0 2 3 3 Leave out requantified Leave out requantified Leave out requantified Median Median Median 0.49108 0.53478 0.53692 0.45475 0.59764 0.46318 0.93747 1.2776 1.118 0.92479 1.1565 0.84727 58.783 31.138 34.536 NaN 31.995 30.055 3 3 3 1 6 5 0 0 0 1 3 3 Leave out requantified Leave out requantified Leave out requantified Median Median Median 1.1639 1.0586 0.87998 0.33524 0.87079 0.81995 0.908 0.84328 0.72561 0.25788 0.72087 0.6433 5.9967 4.5818 25.075 NaN 22.677 17.386 3 3 3 1 6 5 0 0 0 1 3 3 Leave out requantified Leave out requantified Leave out requantified Median Median Median 0.9 0.9 0.9 0.7 1.3 1.3 113160000 52284000 30585000 30290000 14454000 6687300 2611000 5155200 15931000 6973400 4374900 4582200 18821000 8224300 5520600 5075900 6645000 2796800 2567300 1280900 32506000 16025000 8785400 7695400 24804000 11578000 6725800 6500200 129 4366;10455;10641;11164 True;True;True;True 4606;11080;11283;11837 39341;39342;39343;39344;39345;39346;94311;94312;94313;94314;94315;94316;94317;94318;96108;96109;101763;101764;101765;101766;101767;101768;101769;101770;101771;101772;101773 55685;55686;55687;55688;55689;55690;55691;55692;137096;137097;137098;137099;137100;137101;137102;137103;137104;139731;139732;147826;147827;147828;147829;147830;147831;147832;147833;147834;147835;147836;147837;147838 55686;137102;139732;147836

F1LZX9;A0A0G2JVZ6 F1LZX9;A0A0G2JVZ6 7;7 7;7 7;7 Itgav tr|F1LZX9|F1LZX9_RAT Integrin subunit alpha V OS=Rattus norvegicus OX=10116 GN=Itgav PE=1 SV=3;tr|A0A0G2JVZ6|A0A0G2JVZ6_RAT Integrin subunit alpha V OS=Rattus norvegicus OX=10116 GN=Itgav PE=1 SV=1 2 7 7 7 5 7 4 5 3 5 5 7 4 5 3 5 5 7 4 5 3 5 7.4 7.4 7.4 115.58 1047 1047;1052 4.02 4 21 9 6 2 0 17.106 By MS/MS By MS/MS By MS/MS By MS/MS By MS/MS By MS/MS 0.69914 1.7556 44.359 39 6 Leave out requantified 0.72313 1.2999 40.351 39 6 Leave out requantified 1.1189 0.79115 17.791 39 6 Leave out requantified 0.78445 0.72564 0.79131 0.64346 0.72336 0.67772 2.4607 2.1351 2.1945 1.4053 2.2787 1.469 80.589 51.392 43.066 38.062 23.836 25.219 6 7 6 7 6 7 0 0 0 3 0 3 Leave out requantified Leave out requantified Leave out requantified Leave out requantified Leave out requantified Leave out requantified 0.87675 0.77514 0.81599 0.57919 0.75847 0.55873 1.7292 1.9003 1.6758 0.96303 1.5372 1.1885 47.419 20.243 36.304 30.248 30.933 26.927 6 7 6 7 6 7 0 0 0 3 0 3 Leave out requantified Leave out requantified Leave out requantified Leave out requantified Leave out requantified Leave out requantified 1.1461 1.145 1.0764 0.95168 1.148 1.0146 0.77814 0.93993 0.80865 0.68363 0.79451 0.75547 27.148 18.539 27.288 12.133 23.817 19.491 6 7 6 7 6 7 0 0 0 3 0 3 Leave out requantified Leave out requantified Leave out requantified Leave out requantified Leave out requantified Leave out requantified 6.3 7.4 5.1 4.7 3.6 5.3 322230000 139890000 81333000 101000000 23187000 9032700 5902100 8251900 58428000 30109000 12326000 15993000 69037000 28883000 17165000 22989000 38787000 17201000 9762700 11824000 69365000 24640000 20637000 24087000 63422000 30023000 15539000 17860000 130 615;5522;5711;8043;9428;9767;10840 True;True;True;True;True;True;True 649;5826;6026;8538;9995;10358;11493 5361;5362;5363;5364;5365;5366;5367;50591;50592;50593;51888;51889;51890;51891;51892;51893;51894;51895;51896;51897;51898;51899;51900;51901;73093;73094;73095;73096;84891;84892;88024;88025;88026;88027;88028;88029;88030;88031;88032;98083;98084;98085 7537;7538;7539;7540;7541;7542;7543;7544;7545;7546;7547;7548;7549;7550;7551;7552;7553;72848;75066;75067;75068;75069;75070;75071;75072;75073;75074;75075;75076;75077;75078;75079;75080;75081;75082;75083;75084;75085;75086;75087;75088;75089;75090;75091;75092;75093;75094;75095;75096;75097;75098;75099;106297;123606;123607;123608;123609;123610;128048;128049;128050;128051;128052;128053;128054;128055;128056;128057;142489;142490;142491;142492;142493;142494;142495;142496 7553;72848;75085;106297;123606;128054;142491 189 938

A0A0G2JW28;D3ZCH7;G3V9D7;Q62847 A0A0G2JW28;D3ZCH7;G3V9D7;Q62847 1;1;1;1 1;1;1;1 1;1;1;1 Gamma-adducin Add3 tr|A0A0G2JW28|A0A0G2JW28_RAT Gamma-adducin OS=Rattus norvegicus OX=10116 GN=Add3 PE=1 SV=1;tr|D3ZCH7|D3ZCH7_RAT Adducin 3 (Gamma), isoform CRA_b OS=Rattus norvegicus OX=10116 GN=Add3 PE=1 SV=2;tr|G3V9D7|G3V9D7_RAT Adducin 3 (Gamma), isoform CRA_a OS=Rattus 4 1 1 1 1 0 1 0 0 0 1 0 1 0 0 0 1 0 1 0 0 0 4.2 4.2 4.2 74.963 670 670;673;705;705 1 2 0 6.6782 By matching By MS/MS 1.3501 4.6099 69.602 2 0 Median 1.4259 4.0467 32.359 2 0 Median 1.0283 0.73017 18.743 2 0 Median 1.1914 NaN 1.5299 NaN NaN NaN 2.818 NaN 7.5411 NaN NaN NaN NaN NaN NaN NaN NaN NaN 1 0 1 0 0 0 0 0 0 0 0 0 Median Median Median Median Median Median 1.5553 NaN 1.3073 NaN NaN NaN 5.0871 NaN 3.219 NaN NaN NaN NaN NaN NaN NaN NaN NaN 1 0 1 0 0 0 0 0 0 0 0 0 Median Median Median Median Median Median 1.2339 NaN 0.85705 NaN NaN NaN 0.83366 NaN 0.63954 NaN NaN NaN NaN NaN NaN NaN NaN NaN 1 0 1 0 0 0 0 0 0 0 0 0 Median Median Median Median Median Median 4.2 0 4.2 0 0 0 16570000 4032800 6510000 6026800 4442800 1060400 1586500 1795900 0 0 0 0 12127000 2972500 4923500 4230900 0 0 0 0 0 0 0 0 0 0 0 0 131 11746 True 12467 108492;108493 158027;158028;158029 158027

A0A0G2JW64;A0A0G2K0C5 A0A0G2JW64;A0A0G2K0C5 2;2 2;2 2;2 tr|A0A0G2JW64|A0A0G2JW64_RAT Cell division cycle 40 OS=Rattus norvegicus OX=10116 GN=Cdc40 PE=1 SV=1;tr|A0A0G2K0C5|A0A0G2K0C5_RAT Cell division cycle 40 OS=Rattus norvegicus OX=10116 GN=Cdc40 PE=1 SV=1 2 2 2 2 0 1 0 0 1 0 0 1 0 0 1 0 0 1 0 0 1 0 4.9 4.9 4.9 51.323 447 447;579 3 1 1 0.0081781 1.6844 By MS/MS By MS/MS 1.0387 2.6744 114.86 2 0 Median 1.3512 2.9635 37.773 2 0 Median 1.3179 1.0171 77.302 2 0 Median NaN 0.62157 NaN NaN 1.7356 NaN NaN 1.1871 NaN NaN 6.0249 NaN NaN NaN NaN NaN NaN NaN 0 1 0 0 1 0 0 0 0 0 0 0 Median Median Median Median Median Median NaN 1.2719 NaN NaN 1.4355 NaN NaN 2.2689 NaN NaN 3.8708 NaN NaN NaN NaN NaN NaN NaN 0 1 0 0 1 0 0 0 0 0 0 0 Median Median Median Median Median Median NaN 2.0568 NaN NaN 0.8444 NaN NaN 1.7569 NaN NaN 0.58881 NaN NaN NaN NaN NaN NaN NaN 0 1 0 0 1 0 0 0 0 0 0 0 Median Median Median Median Median Median 0 2.2 0 0 2.7 0 7966000 2104900 2575400 3285700 0 0 0 0 2788600 1084000 687090 1017500 0 0 0 0 0 0 0 0 5177400 1021000 1888300 2268200 0 0 0 0 132 7757;12131 True;True 8237;12868 70190;112370 102209;163416 102209;163416

A0A0G2JW69;CON__Q8VED5;CON__P05787;Q10758;CON__Q9H552;G3V712;CON__Q9DCV7;CON__Q3KNV1;CON__P08729;D3ZSY5;A0A0G2K0I4;Q6IG12;CON__H-INV:HIT000016045 A0A0G2JW69;CON__Q8VED5;CON__P05787;Q10758;CON__Q9H552;G3V712;CON__Q9DCV7;CON__Q3KNV1;CON__P08729;D3ZSY5;A0A0G2K0I4;Q6IG12 6;6;5;5;4;3;3;3;3;3;3;3;2 1;1;0;0;0;0;0;0;0;0;0;0;0 1;1;0;0;0;0;0;0;0;0;0;0;0 Keratin, type II cytoskeletal 8;Keratin, type II cytoskeletal 7 Krt8;Krt7;Krt84 tr|A0A0G2JW69|A0A0G2JW69_RAT Keratin 79 (Fragment) OS=Rattus norvegicus OX=10116 GN=Krt79 PE=1 SV=1;;;sp|Q10758|K2C8_RAT Keratin, type II cytoskeletal 8 OS=Rattus norvegicus OX=10116 GN=Krt8 PE=1 SV=3;;tr|G3V712|G3V712_RAT Keratin complex 2, basic, gene 7, 13 6 1 1 3 3 3 3 5 5 0 0 0 0 0 1 0 0 0 0 0 1 8.7 2.5 2.5 52.768 484 484;531;483;483;499;457;457;469;469;557;608;457;99 12 1 0 6.5639 By matching By matching By matching By matching By matching By MS/MS 0.078564 0.29491 NaN 1 1 Median 0.077576 0.24235 NaN 1 1 Median 0.98742 0.71549 NaN 1 1 Median NaN NaN NaN NaN NaN 0.078564 NaN NaN NaN NaN NaN 0.29491 NaN NaN NaN NaN NaN NaN 0 0 0 0 0 1 0 0 0 0 0 1 Median Median Median Median Median Median NaN NaN NaN NaN NaN 0.077576 NaN NaN NaN NaN NaN 0.24235 NaN NaN NaN NaN NaN NaN 0 0 0 0 0 1 0 0 0 0 0 1 Median Median Median Median Median Median NaN NaN NaN NaN NaN 0.98742 NaN NaN NaN NaN NaN 0.71549 NaN NaN NaN NaN NaN NaN 0 0 0 0 0 1 0 0 0 0 0 1 Median Median Median Median Median Median 4.8 4.8 4.8 4.8 6.2 7.6 4789100 4201600 473480 114000 0 0 0 0 0 0 0 0 0 0 0 0 0 0 0 0 0 0 0 0 4789100 4201600 473480 114000 + 133 3622;7572;9720;10488;11651;12052 False;False;True;False;False;False 3824;8031;10310;11115;12360;12361;12787 32878;32879;32880;32881;32882;32883;32884;32885;32886;32887;32888;32889;32890;32891;32892;32893;32894;32895;32896;32897;32898;32899;32900;32901;32902;32903;32904;32905;32906;32907;32908;32909;32910;32911;32912;32913;32914;32915;32916;32917;32918;32919;32920;32921;32922;32923;32924;32925;32926;32927;32928;32929;32930;32931;32932;32933;32934;32935;32936;68168;68169;68170;87782;94563;94564;107374;107375;107376;107377;107378;107379;107380;107381;107382;107383;107384;107385;107386;107387;107388;107389;107390;107391;107392;107393;107394;107395;107396;107397;107398;107399;107400;107401;107402;107403;107404;107405;107406;107407;107408;107409;107410;107411;107412;107413;107414;107415;107416;107417;107418;107419;107420;107421;107422;107423;107424;107425;107426;107427;107428;107429;107430;107431;107432;107433;107434;107435;107436;107437;107438;107439;107440;107441;107442;107443;107444;107445;107446;107447;107448;107449;107450;107451;107452;107453;107454;107455;107456;107457;107458;107459;107460;107461;107462;107463;111518;111519;111520;111521;111522;111523;111524;111525;111526;111527;111528;111529;111530;111531;111532;111533;111534;111535;111536;111537;111538;111539;111540;111541;111542;111543;111544;111545;111546;111547;111548;111549;111550;111551;111552 46086;46087;46088;46089;46090;46091;46092;46093;46094;46095;46096;46097;46098;46099;46100;46101;46102;46103;46104;46105;46106;46107;46108;46109;46110;46111;46112;46113;46114;46115;46116;46117;46118;46119;46120;46121;46122;46123;46124;46125;46126;46127;46128;46129;46130;46131;46132;46133;46134;46135;46136;46137;46138;46139;46140;46141;46142;46143;46144;99255;99256;99257;127690;137392;137393;156291;156292;156293;156294;156295;156296;156297;156298;156299;156300;156301;156302;156303;156304;156305;156306;156307;156308;156309;156310;156311;156312;156313;156314;156315;156316;156317;156318;156319;156320;156321;156322;156323;156324;156325;156326;156327;156328;156329;156330;156331;156332;156333;156334;156335;156336;156337;156338;156339;156340;156341;156342;156343;156344;156345;156346;156347;156348;156349;156350;156351;156352;156353;156354;156355;156356;156357;156358;156359;156360;156361;156362;156363;156364;156365;156366;156367;156368;156369;156370;156371;156372;156373;156374;156375;156376;156377;156378;156379;156380;156381;156382;156383;156384;156385;156386;156387;156388;156389;156390;156391;156392;156393;156394;156395;156396;156397;156398;156399;156400;156401;156402;156403;156404;156405;156406;156407;156408;156409;156410;156411;156412;156413;156414;156415;156416;156417;156418;156419;156420;156421;156422;156423;156424;156425;156426;156427;156428;156429;156430;156431;156432;156433;156434;156435;156436;162208;162209;162210;162211;162212;162213;162214;162215;162216;162217;162218;162219;162220;162221;162222;162223;162224;162225;162226;162227;162228;162229;162230;162231;162232;162233;162234;162235;162236;162237;162238;162239;162240;162241;162242 46098;99257;127690;137392;156297;162234

A0A0G2JW88 A0A0G2JW88 36 36 3 tr|A0A0G2JW88|A0A0G2JW88_RAT Microtubule-associated protein OS=Rattus norvegicus OX=10116 GN=Map4 PE=1 SV=1 1 36 36 3 27 31 33 27 26 28 27 31 33 27 26 28 3 3 3 3 3 3 19.6 19.6 2.1 233.39 2201 2201 2.8 15 104 189 47 7 0 323.31 By MS/MS By MS/MS By MS/MS By MS/MS By MS/MS By MS/MS 0.32617 0.86928 25.001 324 130 Leave out requantified 0.36314 0.75258 24.749 320 125 Leave out requantified 1.0882 0.82034 27.797 319 125 Leave out requantified 0.26034 0.35546 0.33789 0.33978 0.38107 0.31531 0.76793 1.0378 0.81114 0.84641 0.93276 1.0441 44.824 24.02 23.704 24.854 38.188 32.599 59 61 62 47 46 49 27 27 28 18 16 14 Leave out requantified Leave out requantified Leave out requantified Leave out requantified Leave out requantified Leave out requantified 0.46653 0.42232 0.40482 0.36069 0.29233 0.32806 0.98564 0.92769 0.7284 0.66159 0.59613 0.76896 16.727 25.12 22.892 16.002 29.206 16.963 60 61 61 46 45 47 27 27 27 17 15 12 Leave out requantified Leave out requantified Leave out requantified Leave out requantified Leave out requantified Leave out requantified 1.7875 1.1322 1.1697 1.1029 0.79587 1.0662 1.2168 0.89617 0.8877 0.78272 0.58623 0.78136 25.448 19.932 18.89 32.636 28.438 16.342 60 61 61 45 45 47 27 27 27 17 15 12 Leave out requantified Leave out requantified Leave out requantified Leave out requantified Leave out requantified Leave out requantified 17.8 18.6 18.5 15.7 15.9 17.4 5916400000 3420600000 1213200000 1282500000 732390000 423690000 117340000 191370000 833730000 468410000 173990000 191330000 1055300000 611830000 203160000 240300000 651760000 393290000 126500000 131970000 1130600000 655370000 271230000 204030000 1512600000 868050000 321020000 323500000 134 169;188;672;738;739;1357;1409;1410;2174;2175;2409;2493;2502;2503;3315;3534;3535;4959;5319;5552;5839;5904;7371;7856;7857;8304;8761;8853;9915;10214;11038;11195;11285;11286;11784;11785 True;True;True;True;True;True;True;True;True;True;True;True;True;True;True;True;True;True;True;True;True;True;True;True;True;True;True;True;True;True;True;True;True;True;True;True 176;196;708;776;777;1435;1492;1493;2306;2307;2308;2564;2565;2653;2662;2663;3505;3733;3734;5218;5602;5860;6162;6229;7816;7817;8342;8343;8814;9289;9383;10513;10826;11708;11871;11967;11968;12506;12507 1480;1481;1482;1483;1484;1485;1486;1487;1488;1489;1490;1491;1643;1644;1645;1646;1647;1648;1649;1650;1651;1652;1653;1654;1655;1656;1657;1658;1659;1660;1661;1662;1663;1664;1665;1666;1667;1668;1669;1670;5800;5801;6437;6438;6439;6440;6441;6442;6443;6444;6445;6446;6447;6448;6449;6450;6451;6452;12057;12058;12059;12060;12061;12062;12063;12064;12065;12066;12067;12068;12069;12070;12071;12072;12073;12074;12075;12076;12077;12078;12079;12080;12081;12082;12083;12084;12085;12086;12087;12088;12089;12090;12091;12092;12093;12685;12686;12687;12688;12689;12690;12691;12692;12693;12694;12695;12696;12697;12698;20385;20386;20387;20388;20389;20390;20391;20392;20393;20394;20395;20396;20397;20398;20399;22709;22710;22711;22712;22713;22714;22715;22716;22717;22718;22719;22720;22721;22722;22723;22724;22725;22726;22727;22728;22729;22730;22731;22732;23578;23579;23580;23581;23582;23583;23584;23585;23586;23587;23588;23589;23590;23591;23592;23687;23688;23689;23690;23691;23692;23693;23694;30413;30414;30415;30416;30417;30418;30419;30420;30421;30422;30423;32142;32143;32144;32145;32146;32147;32148;45369;45370;45371;45372;45373;45374;45375;48354;48355;48356;48357;48358;50889;50890;50891;53060;53061;53062;53063;53064;53065;53066;53067;53068;53069;53070;53682;53683;53684;53685;53686;53687;53688;53689;53690;53691;53692;53693;53694;53695;53696;53697;53698;53699;53700;53701;53702;53703;53704;53705;66318;66319;66320;66321;66322;66323;66324;66325;66326;66327;66328;66329;66330;66331;66332;66333;66334;66335;66336;66337;66338;66339;66340;71507;71508;71509;71510;71511;71512;71513;71514;71515;71516;71517;71518;71519;75124;75125;75126;75127;75128;75129;75130;75131;75132;75133;75134;75135;75136;75137;75138;75139;75140;78165;78166;79072;79073;79074;79075;79076;79077;79078;89232;89233;89234;89235;89236;89237;89238;89239;89240;89241;89242;89243;89244;91954;100455;100456;100457;100458;100459;100460;102221;102222;102223;102224;102225;102226;102227;102228;102229;103125;103126;103127;103128;103129;103130;103131;103132;103133;108859;108860;108861;108862;108863;108864;108865;108866;108867;108868;108869;108870;108871;108872;108873;108874;108875;108876;108877;108878;108879;108880;108881 2035;2036;2037;2038;2039;2040;2041;2042;2043;2044;2045;2046;2243;2244;2245;2246;2247;2248;2249;2250;2251;2252;2253;2254;2255;2256;2257;2258;2259;2260;2261;2262;2263;2264;2265;2266;2267;2268;2269;2270;2271;2272;2273;2274;2275;2276;2277;2278;2279;2280;2281;2282;2283;2284;2285;2286;8181;8182;8183;9189;9190;9191;9192;9193;9194;9195;9196;9197;9198;9199;9200;9201;9202;9203;9204;9205;9206;9207;9208;9209;9210;9211;9212;9213;9214;9215;9216;9217;17284;17285;17286;17287;17288;17289;17290;17291;17292;17293;17294;17295;17296;17297;17298;17299;17300;17301;17302;17303;17304;17305;17306;17307;17308;17309;17310;17311;17312;17313;17314;17315;17316;17317;17318;17319;17320;17321;17322;17323;17324;17325;17326;17327;17328;17329;17330;17331;17332;17333;17334;17335;17336;17337;17338;17339;18149;18150;18151;18152;18153;18154;18155;18156;18157;18158;18159;18160;18161;18162;18163;18164;18165;18166;18167;18168;28731;28732;28733;28734;28735;28736;28737;28738;28739;28740;28741;28742;28743;28744;28745;28746;28747;28748;28749;28750;28751;31848;31849;31850;31851;31852;31853;31854;31855;31856;31857;31858;31859;31860;31861;31862;31863;31864;31865;31866;31867;31868;31869;31870;31871;31872;31873;31874;31875;31876;31877;31878;31879;31880;31881;33022;33023;33024;33025;33026;33027;33028;33029;33030;33031;33032;33033;33034;33035;33036;33037;33038;33039;33040;33041;33042;33043;33044;33045;33046;33047;33170;33171;33172;33173;33174;33175;33176;33177;33178;33179;33180;33181;42704;42705;42706;42707;42708;42709;42710;42711;42712;42713;42714;42715;45028;45029;45030;45031;45032;45033;45034;45035;45036;45037;45038;45039;45040;65345;65346;65347;65348;65349;65350;65351;65352;65353;65354;69635;73387;73388;73389;73390;77543;77544;77545;77546;77547;77548;77549;77550;77551;77552;77553;77554;77555;77556;77557;77558;78716;78717;78718;78719;78720;78721;78722;78723;78724;78725;78726;78727;78728;78729;78730;78731;78732;78733;78734;78735;78736;78737;78738;78739;78740;78741;78742;78743;78744;78745;78746;78747;78748;78749;78750;78751;78752;78753;96575;96576;96577;96578;96579;96580;96581;96582;96583;96584;96585;96586;96587;96588;96589;96590;96591;96592;96593;96594;96595;96596;96597;96598;96599;96600;96601;96602;96603;96604;96605;96606;96607;96608;96609;104107;104108;104109;104110;104111;104112;104113;104114;104115;104116;104117;104118;104119;104120;104121;104122;104123;104124;104125;104126;104127;104128;104129;104130;104131;104132;109253;109254;109255;109256;109257;109258;109259;109260;109261;109262;109263;109264;109265;109266;109267;109268;109269;109270;109271;109272;109273;109274;109275;109276;109277;109278;109279;109280;109281;109282;109283;113653;113654;114997;114998;114999;115000;115001;115002;115003;115004;115005;115006;115007;115008;115009;129743;129744;129745;129746;129747;129748;129749;129750;129751;129752;129753;129754;129755;129756;129757;129758;129759;129760;129761;129762;129763;129764;133541;146019;146020;148469;148470;148471;148472;148473;148474;148475;148476;148477;148478;148479;148480;148481;149803;149804;149805;149806;149807;149808;149809;149810;149811;149812;149813;149814;149815;149816;158541;158542;158543;158544;158545;158546;158547;158548;158549;158550;158551;158552;158553;158554;158555;158556;158557;158558;158559;158560;158561;158562;158563;158564;158565;158566;158567;158568;158569;158570;158571;158572;158573;158574;158575;158576;158577;158578;158579;158580;158581;158582 2043;2270;8183;9190;9215;17329;18153;18160;28737;28750;31850;33037;33173;33181;42713;45033;45037;65353;69635;73387;77550;78720;96589;104110;104132;109255;113653;114998;129755;133541;146019;148469;149808;149815;158553;158572 190;191;192;193;194;195;196;197;198 243;244;322;349;449;457;477;545;1789

F7FKI5;D4A5G8;A0A0G2JW90;P26284 F7FKI5;D4A5G8;A0A0G2JW90;P26284 3;3;3;3 3;3;3;3 3;3;3;3 Pyruvate dehydrogenase E1 component subunit alpha;Pyruvate dehydrogenase E1 component subunit alpha, somatic form, mitochondrial Pdha1;Pdha1l1 tr|F7FKI5|F7FKI5_RAT Pyruvate dehydrogenase E1 component subunit alpha OS=Rattus norvegicus OX=10116 GN=Pdha1 PE=1 SV=2;tr|D4A5G8|D4A5G8_RAT Pyruvate dehydrogenase E1 component subunit alpha OS=Rattus norvegicus OX=10116 PE=1 SV=3;tr|A0A0G2JW90|A0A0G2JW90_ 4 3 3 3 2 1 0 0 1 3 2 1 0 0 1 3 2 1 0 0 1 3 8.5 8.5 8.5 43.113 389 389;390;390;390 6.86 1 6 0 5.045 By MS/MS By MS/MS By MS/MS By MS/MS 0.26782 1.3431 42.608 6 3 Median 0.37503 0.96139 45.086 5 2 Median 1.4813 1.025 35.101 5 2 Median 0.41371 0.26007 NaN NaN NaN 0.27581 1.8826 1.3338 NaN NaN NaN 1.3525 57.314 NaN NaN NaN NaN 44.175 2 1 0 0 0 3 2 0 0 0 0 1 Median Median Median Median Median Median 0.29108 0.57934 NaN NaN NaN 0.37503 0.91728 2.0588 NaN NaN NaN 0.96139 NaN NaN NaN NaN NaN 38.285 1 1 0 0 0 3 1 0 0 0 0 1 Median Median Median Median Median Median 1.2117 2.1076 NaN NaN NaN 1.7517 0.87454 1.5478 NaN NaN NaN 1.1463 NaN NaN NaN NaN NaN 34.842 1 1 0 0 0 3 1 0 0 0 0 1 Median Median Median Median Median Plateau 6.4 2.1 0 0 3.9 8.5 24719000 18109000 2835500 3774200 1484100 1028900 217530 237640 3142000 1731700 493440 916790 0 0 0 0 0 0 0 0 5543000 5543000 0 0 14550000 9805400 2124500 2619700 135 2309;3586;9044 True;True;True 2463;3786;9589 21834;21835;21836;32605;32606;80805;80806 30634;30635;30636;30637;45697;45698;117575;117576 30637;45698;117575 199 323

D3ZKE6;A0A0G2JWA5;P0C219 D3ZKE6;A0A0G2JWA5;P0C219 13;13;12 13;13;12 1;1;1 Sarcolemmal membrane-associated protein Slmap tr|D3ZKE6|D3ZKE6_RAT Sarcolemma associated protein (Predicted) OS=Rattus norvegicus OX=10116 GN=Slmap PE=1 SV=1;tr|A0A0G2JWA5|A0A0G2JWA5_RAT Sarcolemmal membrane-associated protein OS=Rattus norvegicus OX=10116 GN=Slmap PE=1 SV=1;sp|P0C219|SLMAP_RAT Sarcol 3 13 13 1 7 4 3 8 7 8 7 4 3 8 7 8 1 0 0 1 1 1 19.3 19.3 1.7 90.559 787 787;808;858 5.94 23 1 5 25 9 0 24.398 By MS/MS By MS/MS By MS/MS By MS/MS By MS/MS By MS/MS 0.35766 1.1475 24.565 54 27 Leave out requantified 0.42989 1.0516 15.695 54 27 Leave out requantified 1.1341 0.84687 14.687 54 27 Leave out requantified 0.34337 0.21908 0.41249 0.37163 0.47289 0.5349 1.0545 1.1084 1.3161 1.0429 1.4229 1.198 29.456 39.906 36.595 18.291 9.7165 26.334 11 7 4 11 9 12 3 4 1 7 4 8 Leave out requantified Median Leave out requantified Leave out requantified Leave out requantified Leave out requantified 0.38691 0.29576 0.46411 0.42806 0.61481 0.54033 0.9741 1.0314 1.1373 1.032 1.3663 1.2477 29.581 17.326 19.942 27.905 11.108 6.9733 11 7 4 11 9 12 3 4 1 7 4 8 Leave out requantified Median Leave out requantified Leave out requantified Leave out requantified Leave out requantified 1.1907 1.2317 1.1706 1.2242 1.4034 1.2104 0.87387 0.91768 0.94935 0.9436 1.0375 1.0325 30.104 23.353 12.604 43.642 28.135 44.165 11 7 4 11 9 12 3 4 1 7 4 8 Leave out requantified Median Leave out requantified Leave out requantified Leave out requantified Leave out requantified 10.4 6.5 4.7 14 12.7 11.6 303490000 176150000 50990000 76349000 23434000 13391000 4128100 5915700 32665000 20427000 5503100 6734800 21976000 11513000 5056500 5406900 66715000 38448000 12963000 15304000 98798000 56824000 14144000 27830000 59904000 35551000 9194400 15158000 136 1491;1492;1555;2976;3076;3077;3145;3210;6311;6414;7770;9772;10688 True;True;True;True;True;True;True;True;True;True;True;True;True 1576;1577;1643;3150;3254;3255;3326;3393;6654;6761;8250;10364;11332 13290;13291;13292;13293;13826;13827;13828;13829;13830;13831;28123;28758;28759;28760;28761;28762;28763;28764;28765;28766;28767;29191;29192;29193;29194;29195;29196;29197;29198;29744;57049;58006;58007;58008;58009;58010;58011;58012;58013;58014;58015;58016;58017;58018;70380;88061;88062;88063;88064;96588;96589;96590;96591;96592;96593;96594;96595;96596;96597;96598;96599;96600;96601 18997;18998;18999;19000;19761;19762;19763;19764;19765;19766;19767;19768;19769;39679;40436;40437;40438;40439;40440;40441;40442;40443;40993;40994;40995;40996;40997;40998;40999;41000;41679;83630;85081;85082;85083;85084;85085;85086;85087;85088;85089;85090;85091;85092;85093;85094;85095;102505;128087;128088;128089;128090;140419;140420;140421;140422;140423;140424;140425;140426;140427;140428;140429;140430;140431;140432;140433;140434 18997;19000;19761;39679;40436;40443;40995;41679;83630;85086;102505;128087;140424 200;201 429;565

A0A0G2JWB6;A0A0G2KAH2 A0A0G2JWB6;A0A0G2KAH2 3;2 3;2 3;2 tr|A0A0G2JWB6|A0A0G2JWB6_RAT Peroxidasin OS=Rattus norvegicus OX=10116 GN=Pxdn PE=4 SV=1;tr|A0A0G2KAH2|A0A0G2KAH2_RAT Peroxidasin OS=Rattus norvegicus OX=10116 GN=Pxdn PE=4 SV=1 2 3 3 3 0 1 1 2 1 0 0 1 1 2 1 0 0 1 1 2 1 0 2.2 2.2 2.2 165.21 1475 1475;1237 2.4 3 2 0.00056022 3.1851 By MS/MS By MS/MS By MS/MS By MS/MS 2.1602 4.0785 72.825 5 2 Median 3.1156 5.3015 44.933 5 2 Median 1.2145 0.98087 23.382 5 2 Median NaN 2.1602 2.7125 1.5583 6.3071 NaN NaN 4.0785 5.7876 2.7628 15.939 NaN NaN NaN NaN 11.049 NaN NaN 0 1 1 2 1 0 0 0 0 1 1 0 Median Median Median Median Median Median NaN 2.3524 3.1156 3.0018 7.66 NaN NaN 4.098 5.327 4.9012 12.671 NaN NaN NaN NaN 11.103 NaN NaN 0 1 1 2 1 0 0 0 0 1 1 0 Median Median Median Median Median Median NaN 1.1827 1.1686 1.7371 1.2145 NaN NaN 0.98087 0.95261 1.377 0.8548 NaN NaN NaN NaN 14.53 NaN NaN 0 1 1 2 1 0 0 0 0 1 1 0 Median Median Median Median Median Median 0 0.7 0.7 1.2 0.9 0 24618000 4048000 7758600 12811000 0 0 0 0 4981800 990770 1778500 2212500 6347200 938220 2190500 3218500 9903400 1945400 2624000 5334000 3385300 173600 1165700 2046000 0 0 0 0 137 1927;2751;5697 True;True;True 2041;2921;6012 17636;17637;17638;26113;51823 24811;24812;24813;36585;74962;74963 24811;36585;74963

Q5XI19;A0A0G2JWC7 Q5XI19;A0A0G2JWC7 4;4 4;4 4;4 Fermt2 tr|Q5XI19|Q5XI19_RAT Fermitin family homolog 2 (Drosophila) OS=Rattus norvegicus OX=10116 GN=Fermt2 PE=1 SV=1;tr|A0A0G2JWC7|A0A0G2JWC7_RAT Fermitin family member 2 OS=Rattus norvegicus OX=10116 GN=Fermt2 PE=1 SV=1 2 4 4 4 2 4 3 1 3 4 2 4 3 1 3 4 2 4 3 1 3 4 12.2 12.2 12.2 77.875 680 680;695 1.15 23 4 0 26.09 By MS/MS By MS/MS By MS/MS By MS/MS By MS/MS By MS/MS 0.36332 1.1988 23.445 23 10 Leave out requantified 0.64176 1.6982 44.718 23 10 Leave out requantified 1.5694 1.097 11.599 23 10 Leave out requantified 0.39507 0.36332 0.34969 0.41747 0.3309 0.38051 1.0365 1.0413 1.6349 1.6282 0.99466 1.0691 NaN 14.892 70.696 NaN 23.842 0.31507 1 4 5 1 6 6 1 1 2 0 4 2 Median Leave out requantified Median Median Median Leave out requantified 0.82604 0.55539 0.53191 0.76764 0.6459 0.65184 2.8206 1.9085 1.0499 2.0222 1.5596 1.5354 NaN 55.66 22.852 NaN 18.7 9.6364 1 4 5 1 6 6 1 1 2 0 4 2 Median Leave out requantified Median Median Median Leave out requantified 2.0909 1.247 1.3702 1.7732 1.8911 1.7281 1.4107 0.99008 1.016 1.097 1.4757 1.2341 NaN 13.323 28.155 NaN 12.949 11.03 1 4 5 1 6 6 1 1 2 0 4 2 Median Leave out requantified Median Median Median Leave out requantified 6.5 12.2 9.3 1.5 9.3 12.2 188980000 90185000 35675000 63124000 9036500 4304900 1710900 3020800 33817000 14797000 5674800 13345000 30604000 14470000 7413800 8719500 10848000 4749500 2555000 3543500 65031000 32196000 10238000 22597000 39647000 19667000 8082500 11898000 138 5736;6381;7054;10755 True;True;True;True 6052;6053;6727;7440;11400 52142;52143;52144;52145;52146;52147;52148;52149;52150;52151;57670;57671;63456;63457;63458;63459;63460;63461;63462;63463;63464;97215;97216;97217;97218;97219;97220 75589;75590;75591;75592;75593;75594;75595;75596;75597;75598;75599;75600;75601;75602;75603;75604;75605;75606;75607;75608;75609;75610;75611;75612;75613;84564;84565;92547;92548;92549;92550;92551;92552;92553;92554;92555;92556;92557;92558;92559;92560;92561;92562;141268;141269;141270;141271;141272;141273;141274;141275 75601;84565;92548;141275 202;203 172;236

A0A0G2JWD6;D3ZIF5;D4AE00 A0A0G2JWD6;D3ZIF5 8;5;1 8;5;1 8;5;1 Ap3b1 tr|A0A0G2JWD6|A0A0G2JWD6_RAT AP-3 complex subunit beta OS=Rattus norvegicus OX=10116 GN=Ap3b1 PE=1 SV=1;tr|D3ZIF5|D3ZIF5_RAT Adaptor-related protein complex 3 subunit beta 1 OS=Rattus norvegicus OX=10116 GN=Ap3b1 PE=1 SV=1 3 8 8 8 7 5 3 3 4 3 7 5 3 3 4 3 7 5 3 3 4 3 8.5 8.5 8.5 121.5 1096 1096;794;1082 3.47 8 1 15 8 0 11.571 By MS/MS By MS/MS By MS/MS By MS/MS By MS/MS By MS/MS 0.53066 1.7102 19.883 32 6 Leave out requantified 0.50095 1.1374 18.585 32 6 Leave out requantified 0.91276 0.70941 18.758 32 6 Leave out requantified 0.4467 0.6181 0.58298 0.51676 0.61054 0.48871 1.3779 1.9076 2.073 1.2379 1.8421 1.4774 19.829 10.049 26.051 5.2323 47.786 44.367 8 6 7 3 5 3 3 1 1 1 0 0 Leave out requantified Leave out requantified Leave out requantified Median Leave out requantified Leave out requantified 0.48389 0.49526 0.59763 0.50569 0.44134 0.65143 1.1222 1.2323 1.5611 1.0125 1.0263 1.5403 20.825 16.749 27.065 5.9203 39.628 24.347 8 6 7 3 5 3 3 1 1 1 0 0 Leave out requantified Leave out requantified Leave out requantified Plateau Leave out requantified Leave out requantified 1.147 0.8111 1.0523 0.90367 0.82094 0.93776 0.82935 0.63857 0.85366 0.69741 0.60617 0.7077 15.632 9.9146 4.1396 6.211 9.4711 9.4074 8 6 7 3 5 3 3 1 1 1 0 0 Leave out requantified Leave out requantified Leave out requantified Median Leave out requantified Leave out requantified 5.8 6.4 4.4 2.8 5.7 2.6 209290000 101790000 54173000 53328000 28948000 14578000 6399700 7970800 38557000 18691000 10474000 9391100 54625000 26433000 13055000 15137000 19234000 9288200 5133100 4812200 37498000 18364000 10957000 8177300 30427000 14432000 8154700 7839600 139 1221;2644;3560;6543;6891;7394;8649;9161 True;True;True;True;True;True;True;True 1292;2807;3759;6895;7249;7842;9172;9716 10824;10825;10826;10827;24974;24975;24976;24977;32351;58832;61985;66600;66601;66602;66603;66604;66605;66606;66607;66608;66609;77601;77602;77603;77604;77605;77606;77607;82048;82049;82050;82051 15537;15538;15539;15540;15541;15542;34968;34969;45339;86189;90634;96976;96977;96978;96979;96980;96981;96982;96983;96984;96985;112858;112859;112860;112861;112862;112863;119305;119306;119307 15540;34968;45339;86189;90634;96977;112859;119305
[truncated: 5,104,096 more chars]
